# Identification of Real MicroRNA Precursors with a Pseudo Structure Status Composition Approach

**DOI:** 10.1371/journal.pone.0121501

**Published:** 2015-03-30

**Authors:** Bin Liu, Longyun Fang, Fule Liu, Xiaolong Wang, Junjie Chen, Kuo-Chen Chou

**Affiliations:** 1 School of Computer Science and Technology, Harbin Institute of Technology Shenzhen Graduate School, Shenzhen, Guangdong, China; 2 Key Laboratory of Network Oriented Intelligent Computation, Harbin Institute of Technology Shenzhen Graduate School, Shenzhen, Guangdong, China; 3 Gordon Life Science Institute, Belmont, Massachusetts, United States of America; 4 Center of Excellence in Genomic Medicine Research (CEGMR), King Abdulaziz University, Jeddah, Saudi Arabia; Sabanci University, TURKEY

## Abstract

Containing about 22 nucleotides, a micro RNA (abbreviated miRNA) is a small non-coding RNA molecule, functioning in transcriptional and post-transcriptional regulation of gene expression. The human genome may encode over 1000 miRNAs. Albeit poorly characterized, miRNAs are widely deemed as important regulators of biological processes. Aberrant expression of miRNAs has been observed in many cancers and other disease states, indicating they are deeply implicated with these diseases, particularly in carcinogenesis. Therefore, it is important for both basic research and miRNA-based therapy to discriminate the real pre-miRNAs from the false ones (such as hairpin sequences with similar stem-loops). Particularly, with the avalanche of RNA sequences generated in the postgenomic age, it is highly desired to develop computational sequence-based methods in this regard. Here two new predictors, called “iMcRNA-PseSSC” and “iMcRNA-ExPseSSC”, were proposed for identifying the human pre-microRNAs by incorporating the global or long-range structure-order information using a way quite similar to the pseudo amino acid composition approach. Rigorous cross-validations on a much larger and more stringent newly constructed benchmark dataset showed that the two new predictors (accessible at http://bioinformatics.hitsz.edu.cn/iMcRNA/) outperformed or were highly comparable with the best existing predictors in this area.

## Introduction

**Fig 1:**
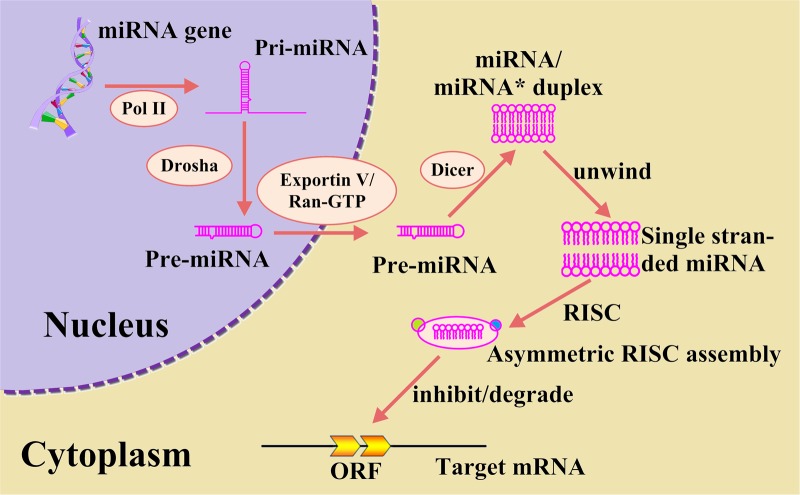
An illustration to show biogenesis of miRNAs and model of miRNA-mediated translational repression or mRNA degradation. MiRNA genes are transcribed by RNA polymerase II [2,90], resulting in the primary transcripts termed as pri-miRNAs, which are typically 60–70 nucleotides. The pri-miRNAs are processed by the enzyme Drosha to release the hairpin-shaped intermediates (pre-miRNAs) [3], followed by being exported into the cytoplasm by Exportin V and Ran-GTP cofactor [4–6], and then cleaved by the enzyme Dicer to yield miRNA/miRNA* duplexes [7–11].

MicroRNAs (miRNAs) are small single-strand, non-coding RNAs about 22 nucleotides (nt) in length, which play important roles in gene regulation by targeting messenger RNAs (mRNAs) for cleavage or translational repression. The miRNAs are also involved in many important biological processes, such as affecting stability, translation of mRNAs and negatively regulating gene expression in post-transcriptional processes. In animals, the biogenesis of miRNA is shown in Fig. 1, and can be divided into the following steps: (i) The genes of miRNA are transcribed by RNA polymerase II [1,2], resulting in the primary transcripts termed as pri-miRNAs, which are typically 60–70 nt. (ii) The pre-miRNAs are processed by the enzyme Drosha to release the hairpin-shaped intermediates (pre-miRNAs) [3]. (iii) The pre-miRNAs are then exported into the cytoplasm by Exportin V and Ran-GTP cofactor [4–6] and cleaved by the enzyme Dicer to yield miRNA/miRNA* duplexes [7–11].

Owing to the difficulty of systematically detecting miRNAs from a genome by existing experiment techniques, computational methods have been indispensable tools in miRNA studies [12]. Various computational methods have been proposed to predict pre-miRNAs. Most of these methods employed the machine learning techniques to build their prediction models, which treated this problem as a binary classification task to discriminate the real pre-miRNAs from false pre-miRNAs. These methods are different in the feature selections and machine learning algorithms or operation engines. The machine learning algorithms usually used in this field include Support Vector Machine (SVM) [11,13–20], Random Forest (RF) [21–23], Hidden Markov Model (HMM) [24], Covariant Discrimination (CD) [25] or Naive Bayes (NB) [26], and Linear Genetic Programming (LGP) [27].

The secondary structure is an important feature used in the computational methods, because most of the pre-miRNAs have the characteristic of stem-loop hairpin structures [16]. Mir-abela [28] is an SVM-based method trained with 16 statistic features computed from the entire hairpin structure. Triplet-SVM [16] employed a SVM classifier to train 32 local triplet sequence-structure features. Later, MiPred [21] improved Triplet-SVM [16] by employing the Random Forest classifier trained with the local triplet sequence-structure features, minimum of free energy (MFE), and *P*-values. MiRFinder [29] is a high-throughput pre-miRNA prediction method, which consists of two steps: a search for hairpin candidates and exclusion of the non-robust structures based on the analysis of 18 parameters by the SVM.

All these computational methods could yield quite encouraging results, and each of them did play a role in simulating the development of pre-miRNA identification. However, further work is needed due to the following reasons: (i) The datasets constructed in those methods were too small to reflect the statistical profile of human pre-miRNAs. Most of these methods were trained and tested with a dataset containing only several hundreds of human pre-miRNA samples or pseudo pre-miRNA samples. (ii) No cutoff threshold was imposed to rigorously exclude the redundant samples or those with high sequence similarity with others in a same benchmark dataset. (iii) Most of these methods only consider the local structure or sequence order information of RNA sequences, and all the global or long range structure or sequence order effects were ignored.

In this study, we attempted to improve the accuracy for human pre-miRNA identification from the above three aspects; especially, we focused on how to incorporate the global structure-order effects into the predictor. However, it is difficult to incorporate this kind of information into a statistical predictor because the RNA sequences have different lengths with extremely large number of possible structure patterns. To overcome this difficulty, is it possible to find an approximate way to take the structure-order effects into account?

Actually, similar problems were also encountered in computational proteomics and genomics. To incorporate the long-range or global sequence order information for protein/peptide sequences, the pseudo amino acid composition [30,31] or Chou’s PseAAC [32] was proposed. Ever since the concept of PseAAC was proposed in 2001[30], it has been penetrating into almost all the fields of protein attribute predictions (see, e.g., [33–44], as well as a Wikipedia article at http://en.wikipedia.org/wiki/Pseudo_amino_acid_composition and a long list of papers cited in [45]) and some fields of drug development and biomedicine [46]. Recently, the concept of PseAAC has also been further extended to the field of genomics by using different modes of pseudo K-tuple nucleotide composition or PseKNC [47–49] to predict the recombination spots of DNA [19,50], the nucleosome positions [20], sigma-54 promoters [51], and DNA methylation sites [52]. For more information about this, see a recent review [53].

Encouraged by the successes of PseAAC and PseKNC approaches in the fields of proteomics and genomics, we proposed a feature vector called “pseudo structure status composition (PseSSC)” to represent RNA sequences by incorporating the structure-order effects so as to improve the prediction quality in identifying human pre-miRNA. The detailed approach is elaborated as follows.

As pointed out in a comprehensive review [54] and carried out in a series of recent publications (see, e.g., [19,20,50,55–57]), to develop a really useful statistical predictor or model for a biological system, one needs to engage the following procedures: (i) construct or select a valid benchmark dataset to train and test the predictor; (ii) formulate the statistical samples with an effective mathematical expression that can truly reflect their intrinsic correlation with the target to be predicted; (iii) introduce or develop a powerful algorithm (or engine) to operate the prediction; (iv) properly perform cross-validation tests to objectively evaluate the anticipated accuracy of the model; (v) establish a user-friendly web-server for the predictor that is accessible to the public. Below, let us describe how to deal with these procedures one-by-one.

## Materials and Method

### 1. Benchmark Dataset

The pre-miRNAs or positive samples were downloaded from the latest version (release 20: June 2013) of miRNABase [58,59], which contained 1,872 experiment-confirmed sapiens pre-miRNA entries. The false pre-miRNAs or negative samples were taken from the data constructed by Xue et al. [16], which contained 8,489 false pre-miRNA samples. These false pre-miRNAs are similar to the real pre-miRNAs according to the following widely accepted characteristics [16]: (i) the RNA length ranges from 51 nt to 137 nt; (ii) a minimum of 18 base pairings on the stem of the hairpin structure; (iii) a maximum of-15 kal/mol free energy of the secondary structure.

To get rid of the redundancy and avoid homology bias, the CD-HIT software [60] with the cutoff threshold set at 80% (note that the most stringent cutoff threshold for DNA sequences by CD-HIT is 75%) was used to winnow those samples which had ≥80% sequence identity to any other in a same subset. After such a screening procedure, we obtained 1,612 human pre-miRNAs, which formed the positive dataset in the current study.

To avoid imbalance problem caused by different number of positive and negative samples, we randomly picked 1,612 samples from the 8,489 false pre-miRNAs to form the negative dataset. Again, none of the samples included had ≥80% sequence identity to any other in a same subset.

As pointed out by a review [25], there is no need to separate a benchmark dataset into a training dataset and a testing dataset for validating a prediction method if it is tested by the jackknife or subsampling (K-fold) cross-validation because the outcome thus obtained is actually from a combination of many different independent dataset tests. Therefore, the benchmark dataset 𝕊 can be formulated as

$$S=S_{}^{+}\cup S_{}^{-}$$ (1)

where the subset 𝕊^+^contains 1,612 human pre-miRNAs, the subset 𝕊^-^ contains 1,612 false pre-miRNAs, and the symbol represents the “union” in the set theory.

The detailed sequences are given in S1 Dataset that is not only the largest but also most stringent benchmark dataset in this area.

### 2. Pseudo Structure Status Composition (PseSSC)

Suppose a RNA sequence **R** with *L* nucleobases (nitrogenous bases or nucleic acid residues); i.e.,

$$\text{R}={\text{B}}_{1}{\text{B}}_{2}{\text{B}}_{3}{\text{B}}_{4}{\text{B}}_{5}\ldots {\text{B}}_{L}$$ (2)

where B_1_ denotes the nucleobase at sequence position 1, B_2_ denotes the base at position 2, and so forth. They can be any of the four nucleobases; i.e.,

$${\text{B}}_{i}^{}\in \left\{\text{adenine}\left(\text{A}\right)\text{, cytosine}\left(\text{C}\right)\text{, guanine}\left(\text{G}\right)\text{, uracil}\left(\text{U}\right)\right\}\;i=\text{1, 2,}\cdots \text{,}\;L$$ (3)

**Fig 2:**
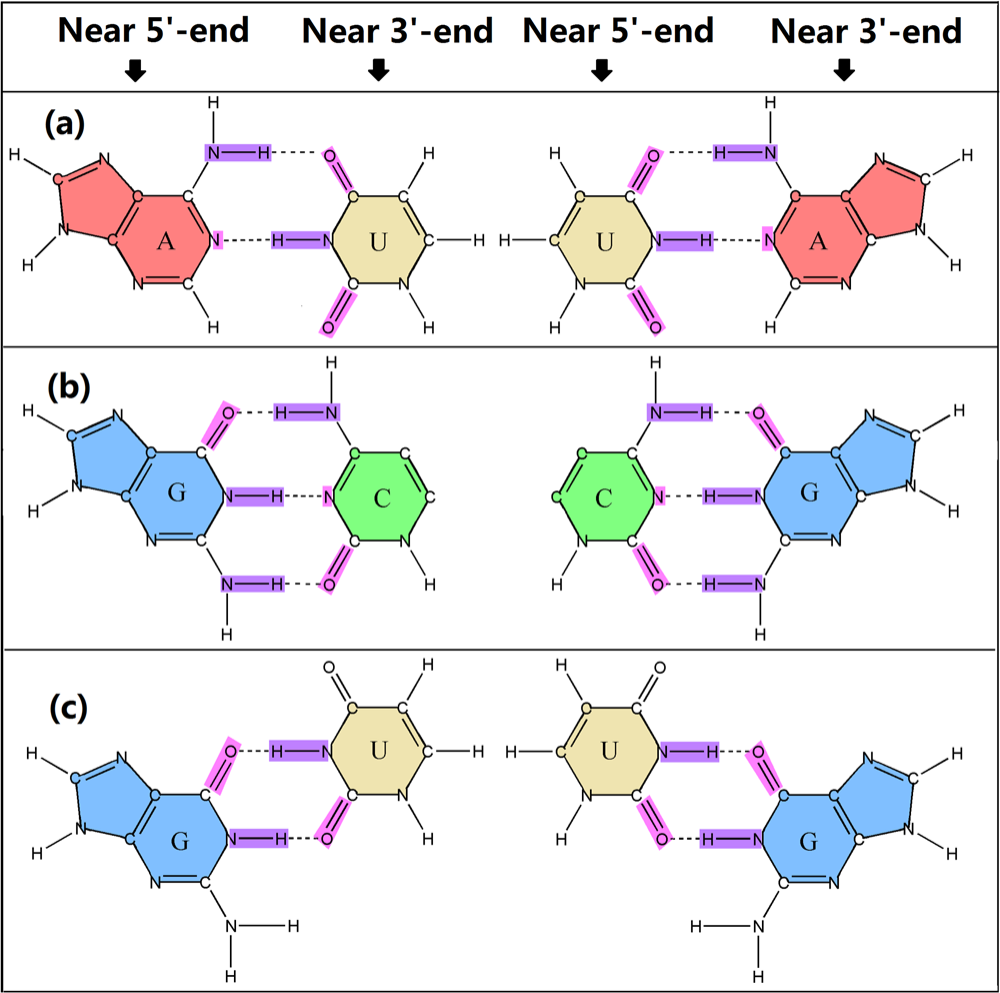
Illustration to show the 6 structure statuses of paired nucleic acid residues. Note that the nucleotide near 5’ end is different with the one near 3’end: (a) the base pairs A-U or U-A has 2 hydrogen bonds; (b) the base pair G-C or C-G has 3 hydrogen bonds; and (c) the wobble base pair G-U or U-G has 2 weaker hydrogen bonds. See the main text for further explanation.

If the RNA sequence is formulated according to its secondary structure derived from the Vienna RNA software package (released 2.1.6) [61], we have

$$\text{R}=\Psi_{1}^{}\Psi_{2}^{}\Psi_{3}^{}\Psi_{4}^{}\Psi_{5}^{}\cdots \Psi_{L}^{}$$ (4)

where Ψ_1_ denotes the structure status of B_1_, Ψ_2_ the structure status of B_2_, and so forth. They can be any of the 10 structure statuses; i.e.,

$$\Psi_{i}^{}\in \left\{\text{A, C, G, U, A-U, U-A, G-C, C-G, G-U, U-G}\right\}\;i=\text{1, 2,}\cdots \text{, }L$$ (5)

where A, C, G, U represent the structure statuses of the four unpaired nucleobases, while A-U, U-A, G-C, C-G, G-U, U-G represent the structure statuses of the six paired bases. Note that A-U means the base A located near the 5’-end paired with its complementary base U near the 5’-end. Therefore, A-U and U-A represent two different structure statuses. The same is true for G-C, C-G, G-U, U-G. Therefore, we have additional six different structure statuses of the paired bases in RNA (Fig. 2).

**Fig 3:**
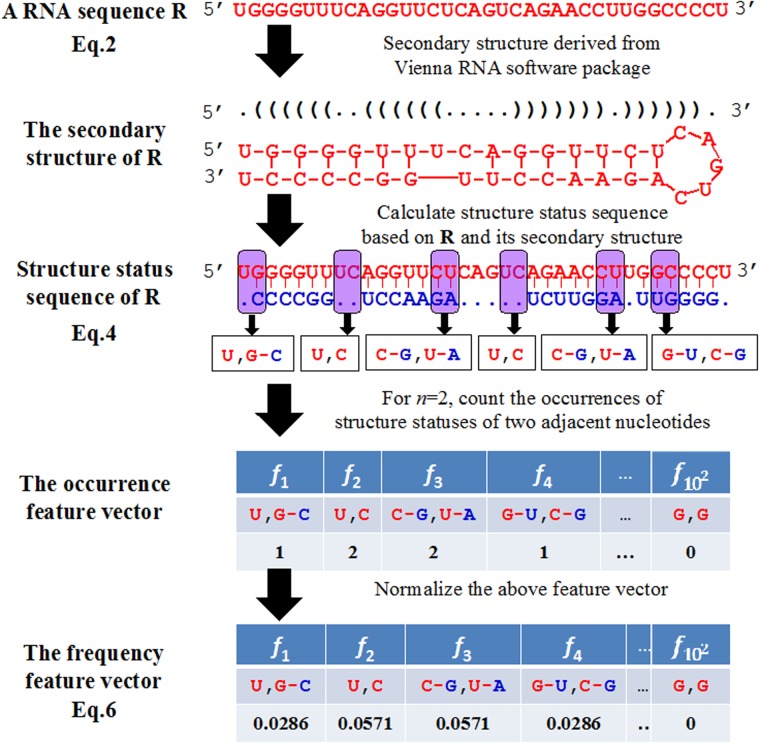
A flowchart to show the process of generating the feature vector for a RNA sequence by its structure status composition. Given a RNA sequence R (cf. Equation 2), its secondary structure sequence was derived from Vienna RNA software package, as formulated in Equation 4. According to the definition in that package, there are two types of status for each of the nucleotides: unpaired or paired. The former is denoted by a dot “.” and the latter by the symbol “(“or “)”. The left bracket “(“stands for a nucleotide near the 5'-end while the right bracket for the one near the 3'-end. Since the number of different structure elements in the RNA sequence thus obtained is 10 (cf. Equation 5), its *n*-tuple element composition will contain 10^*n*^components (cf. Equation 6). For simplicity, however, shown here is only for the case of *n* = 2; i.e., the 2-tuple element composition that contains 10^2^ = 100 components formed by different pairs of the most contiguous secondary structure status elements.

Based on the ten structure statuses, if the RNA sequence is represented by the structure statuses of its *n* adjacent nucleotides, or the so-called “*n*-tuple nucleobase composition” [47], the corresponding feature vector will contain 10^*n*^ components as given by (cf. Fig. 3)

$$R={\left[\begin{array}{cccccc} f_{\text{1}}^{} & f_{2}^{} & f_{3}^{} & f_{4}^{} & \cdots & f_{{\text{10}}^{n}}^{} \end{array}\right]}^{T}$$ (6)

where *f*_*i*_ = (*i* = 1,2,…,10^*n*^) represents the normalized occurrence frequency of the structure status combination of *n* adjacent nucleobases. As indicated by the above equation, with the increase of *n*, the structure-order information within a local or short-range scope could be incorporated, but none of the global or long-range structure information would be reflected.

Stimulated by the PseAAC approach [30,31] in computational proteomics, here we are to propose a novel feature vector called the pseudo structure status composition (PseSSC) to incorporate the global or long-range structure-order information so as to improve the prediction quality in identifying the pre-miRNAs. The detailed procedures are described as follows.

**Fig 4:**
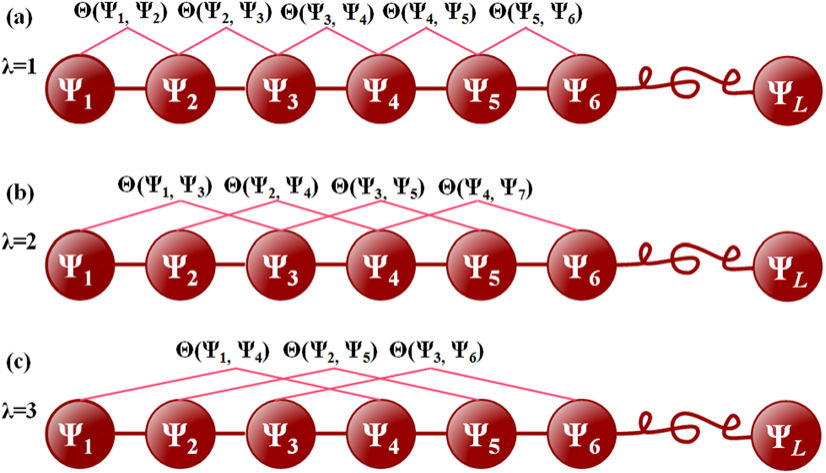
A schematic illustration to show the correlation of structure statuses along a RNA sequence. (a) The first-tier correlation reflects the structure-order mode between all the most contiguous nucleotides. (b) The 2nd-tier correlation reflects the structure-order mode between all the second-most contiguous nucleotides. (c) The 3rd-tier correlation reflects the structure-order mode between all the third-most contiguous nucleotides. As we can see, the global or long-range sequence order information of RNA can thus be approximately and indirectly incorporated into the current prediction model as done by the PseAAC approach for proteins [30].

In a way parallel to the formulation in [30], the global structure-order information for the RNA structure status sequence of Equation 4 can be reflected by a series correlation factors as given by

$$\left\{ \begin{array}{l} \theta_{1}=\frac{1}{L-1}\overset{L-1}{\underset{i=1}{\sum }}\Theta (\Psi_{i},\Psi_{i+1})\\ \theta_{2}=\frac{1}{L-2}\overset{L-2}{\underset{i=1}{\sum }}\Theta (\Psi_{i},\Psi_{i+2})\\ \theta_{3}=\frac{1}{L-3}\overset{L-3}{\underset{i=1}{\sum }}\Theta (\Psi_{i},\Psi_{i+3})\text{ (}\lambda <L\text{)}\\ \ldots \ldots \\ \theta_{\lambda }=\frac{1}{L-\lambda }\overset{L-\lambda }{\underset{i=1}{\sum }}\Theta (\Psi_{i},\Psi_{i+\lambda }) \end{array}\right.$$ (7)

where λ is an integer, representing the highest counted rank (or tier) of the structural correlation along a RNA chain; θ_1_ is the first-tier correlation factor reflecting the structure-order information between all the most contiguous bases along a RNA chain (Fig. 4a); θ_2_ the second-tier correlation factor between all the second most contiguous nucleobases (Fig. 4b); θ_3_ the third-tier correlation factor between all the third most contiguous nucleobases (Fig. 4c); and so forth. In Equation 8 the correlation function is given by

$$\Theta \left(\Psi_{i},\Psi_{j}\right)={\left[F(\Psi_{i})-F(\Psi_{j})\right]}^{2}$$ (8)

where *F*(Ψ_*i*_) is the free energy of the structure status Ψi of the nucleobase at position *i*, and *F*(Ψ_*j*_) is the free energy of the structure status Ψ_*j*_ of the nucleobase at position *j*. As mentioned above, if we distinguish the nucleobase near 5’ end and 3’ end, there are 6 different structure statuses for the paired nucleobases (Fig. 2). For the base pairs A-U and U-A, since they have 2 hydrogen bonds, their free energy values could be set as-2 kcal/mol; for the base pairs G-C or C-G, they have 3 hydrogen bonds (Fig. 2b) and hence their free energy values were set as-3 kcal/mol; for the wobble base pairs G-U and U-G (Fig. 2c), their free energy values were set as-1 kcal/mol; for the four unpaired nucleobases, their free energy values were each set as 0 kcal/mol.

After incorporating the correlation factors, the original Equation 6 for the *n*-tuple nucleobase composition of RNA is augmented to

$$R={\left[\begin{array}{cccccccc} f_{\text{1}}^{*} & f_{2}^{*} & f_{3}^{\text{ *}} & \cdots & f_{{\text{10}}^{n}}^{*} & f_{{\text{10}}^{n}+1}^{*} & \cdots & f_{{\text{10}}^{n}+\lambda }^{*} \end{array}\right]}^{T}$$ (9)

where

$$f_{}^{*}=\{\begin{array}{ll} \frac{f_{u}^{}}{\sum_{i=1}^{10^{n}}f_{i}^{}+w\sum_{j=1}^{\lambda }\theta_{j}^{}} & \left(1\leq u\leq 10_{}^{n}\right)\\ \frac{w\theta_{u-10_{}^{n}}^{}}{\sum_{i=1}^{10^{n}}f_{i}^{}+w\sum_{j=1}^{\lambda }\theta_{j}^{}} & \left(10_{}^{n}+1\leq u\leq 10_{}^{n}+\lambda \right) \end{array}$$ (10)

where *f*_*i*_ = (*i* = 1,2,…,10^*n*^) are the same as in Equation 6, θ_*j*_ the *j*-tier sequence correlation factor computed according to Equations 7–8 for the RNA sequence, and *w* is the weight factor used to adjust the effect of the correlation factors.

As shown in Equations 9 and 10, the first 10^*n*^components reflect the effect of the *n-*tuple structure status composition, whereas the components from 10^*n*^+1 to 10^*n*^+λ reflect the effect of structure order. A vector formed with such 10^*n*^+λ components is called pseudo structure status composition or PseSSC for the RNA sequence with *L* nucleobases.

Finally, the PseSSC vector of Equation 9 was further augmented to

$$\tilde{R}={\left[\begin{array}{cccccccccccc} f_{\text{1}}^{*} & f_{2}^{*} & \cdots & f_{{\text{10}}^{n}}^{*} & f_{{\text{10}}^{n}+1}^{*} & \cdots & f_{{\text{10}}^{n}+\lambda }^{*} & a & b & c_{1} & \cdots & c_{64} \end{array}\right]}^{T}$$ (11)

where $\tilde{R}$ is the augmented PseSSC, *a* is the minimum of free energy (MFE) derived from the Vienna RNAsoftware package (released 2.1.6) [61], *b* the *P*-value of randomization test feature calculated by using the Monte Carlo randomization test [62], and c_*i*_(*i* = 1,2,…,64) the occurrence frequencies of the tri-nucleobases in the RNA sequence. A feature vector formed with such 10^*n*^+λ+66 components is called extended pseudo structure status composition or ExPseSSC for the RNA sequence with *L* nucleobases.

### 3. Support Vector Machine

Support Vector Machine (SVM) is a class of supervised learning algorithms first introduced by Vapnik [63]. Given a set of labelled training vectors (positive and negative input samples), SVM learns a linear decision boundary from both positive and negative training samples to discriminate between the unseen protein sequences. A key feature of SVM is that it needs fixed length of the input vector. The proteins in the training set and test set were transformed into fixed-dimension feature vectors following the process introduced above, and then the training vectors were input into SVM to construct the classifier. The SVM gives a predicted class for each sample in the test set.

In the current study, the LIBSVM algorithm [64] was employed, which is a type of software for SVM classification and regression. The kernel function was set as Radial Basis Function (RBF), which is defined as

$$K\left(X_{i},X_{j}\right)=\exp\left(-\gamma {\left\|X_{i}-X_{j}\right\|}^{2}\right)$$ (12)

The two parameters *C* and γ were optimized on the benchmark dataset by adopting the grid tool provided by LIBSVM [64], and their actual values in this study will be given later. For a brief formulation of SVM and how it works, see the paper [65]; for more details about SVM, see a monograph [66].

Finally, we obtain two predictors, one is based on Equation 9, and the other based on Equation 11, as formulated below

$$\{\begin{array}{ll} \text{iMcRNA-PseSSC,} & \text{if use }R\text{ of Eq.9 to represent RNA samples }\\ \text{iMcRNA-ExPseSSC,} & \text{if use }\tilde{R}\text{ of Eq.11 to represent RNA samples} \end{array}$$ (13)

where “i” stands for “identifying”, “McRNA” for “microRNA”, “Pse” for “pseudo”, “SS” for “structure status”, “C” for “composition”, and “Ex” for extended.

### 4. Cross Validation

In examining the accuracy of a statistical predictor, it is very important to choose an objective method to perform the test. In literature, the following three cross-validation methods are often used to examine the quality of a predictor and its effectiveness in practical application: independent dataset test, subsampling or K-fold (such as 5-fold, 7-fold, or 10-fold) crossover test, and jackknife test. However, as elucidated by a penetrating analysis in [54], considerable arbitrariness exists in the independent dataset test. Also, as demonstrated by Eqs.28–32 of [54], the subsampling (or K-fold crossover validation) test cannot avoid arbitrariness either. Only the jackknife test is the least arbitrary that can always yield a unique result for a given benchmark dataset. Therefore, the jackknife test has been widely recognized and increasingly utilized by investigators to examine the quality of various predictors (see, e.g., [20,41,57,67–72]). Accordingly, in this study we also use the jackknife test to evaluate the accuracy of the current predictor. During the jackknife test, each of the samples in the benchmark dataset is in turn singled out as an independent test sample and all the rule-parameters are calculated without including the sample being identified. Although the jackknife test may take more computational time, it is worthwhile because it will yield a unique outcome for a given benchmark dataset.

### 5. Metrics for Measuring Prediction Quality

After choosing the cross validation method, the next important thing is how to quantitatively measure the prediction quality. To introduce a more intuitive and easier-to-understand method for scoring the prediction quality, the following set of metrics based on the formulation used by Chou [73] in predicting signal peptides was adopted. According to the formulation, the sensitivity Sn, specificity Sp, overall accuracy Acc, and Matthews correlation coefficient MCC can be respectively expressed as [19,20,50]

$$\left\{ \begin{array}{l} Sn=1-\frac{N_{-}^{+}}{N^{+}},\;0\leq \text{Sn}\leq 1\\ \text{Sp}=1-\frac{N_{+}^{-}}{N^{-}},\;0\leq \text{Sp}\leq 1\\ \text{Acc}=1-\frac{N_{-}^{+}\;+\;N_{+}^{-}}{N^{+}\;-\;N^{-}},\;0\leq \text{Acc}\leq 1\\ \text{MCC}=\frac{1-\left(\frac{N_{-}^{+}}{N^{+}}+\frac{N_{+}^{-}}{N^{-}}\right)}{\sqrt{\left(1+\frac{N_{+}^{-}\;+\;N_{-}^{+}}{N^{+}}\right)\left(1+\frac{N_{+}^{-}\;-\;N_{-}^{+}}{N^{-}}\right)}},\;-1\leq \text{MCC}\leq 1 \end{array}\right.$$ (14)

where *N* ^*+*^is the total number of the pre-miRNAs investigated whereas $N_{-}^{+}$ the number of the pre-miRNAs incorrectly predicted as false pre-miRNAs; *N* ^-^ the total number of the false pre-miRNAs investigated whereas $N_{+}^{-}$ the number of the false pre-miRNAs incorrectly predicted as the real pre-miRNAs.

According to Equation 14 we can easily see the following. When $N_{-}^{+}=0$ meaning none of the pre-miRNAs was mispredicted to be a false pre-miRNAs, we have the sensitivity Sn = 1; while $N_{-}^{+}=N_{}^{+}$ meaning that all the real pre-miRNAs were mispredicted to be the false pre-miRNAs, we have the sensitivity Sn = 0. Likewise, when $N_{+}^{-}=0$ meaning none of the false pre-miRNAs was mispredicted, we have the specificity Sp = 1; while $N_{+}^{-}=N_{}^{-}$ meaning all the false pre-miRNAs were incorrectly predicted as real pre-miRNAs, we have the specificity Sp = 0. When $N_{-}^{+}=N_{+}^{-}=0$ meaning that none of the pre-miRNAs in the dataset 𝕊^+^ and none of the false pre-miRNAs in 𝕊^-^ was incorrectly predicted, we have the overall accuracy Acc = 1; while $N_{-}^{+}=N_{}^{+}$ and $N_{+}^{-}=N_{}^{-}$ meaning that all the real pre-miRNAs in the dataset 𝕊^+^ and all the false pre-miRNAs in 𝕊^-^ were mispredicted, we have the overall accuracy Acc = 0. The Matthews correlation coefficient (MCC) is usually used for measuring the quality of binary (two-class) classifications. When $N_{-}^{+}=N_{+}^{-}=0$ meaning that none of the real pre-miRNAs in the dataset 𝕊^+^ and none of the false pre-miRNAs in 𝕊^-^ was mispredicted, we have MCC = 1; when $N_{-}^{+}=N_{}^{+}/2$ and $N_{+}^{-}=N^{-}/2$ we have MCC = 0 meaning no better than random prediction; when $N_{-}^{+}=N_{}^{+}$ and $N_{+}^{-}=N_{}^{-}$ we have MCC = -1 meaning total disagreement between prediction and observation. As we can see from the above discussion, it is much more intuitive and easier to understand when using Equation 14 to examine a predictor for its four metrics, particularly for its Mathew’s correlation coefficient. It is instructive to point out that the metrics as defined in Equation 14 are valid for single label systems; for multi-label systems, a set of more complicated metrics should be used as given in [74].

## Results and Discussion

### 1. Performance of iMcRNA-PseSSC and iMcRNA-ExPseSSC

As we can see from Equation 9–11, both the iMcRNA-PseSSC and iMcRNA-ExPseSSC predictors contain three uncertain parameters, namely *n*,λ, and *w*, where *n* reflects the local or short-range structure-order effect, λ reflects the global or long-range structure-order effect, and 0077 is the factor to adjust the weight between the local and global effects. Generally speaking, the greater the *n* is, the more local structure-order information is incorporated. And the greater the λ is, the more global structure-order information is taken into account. However, if *n* or λ is too large, it would reduce the cluster-tolerant capacity [75] and cause the “overfitting” or “high dimension disaster” [76] problem, so as to reduce the prediction accuracy. Accordingly, in the current study, their optimal values were determined within the ranges as defined below

$$\left\{ \begin{array}{l} 1\leq n\leq 4\text{ with step }\Delta =1\\ 1\leq \lambda \leq 20\text{ with step }\Delta =1\\ 0\leq w\leq 1\text{ with step }\Delta =0.1 \end{array}\right.$$ (15)

It can be seen from Equation 15 that, to determine the optimal values for the three parameters, 4×20×10 = 800 different combination cases need to be considered. To reduce the computational time, we adopted the 5-fold cross-validation approach on the benchmark dataset. The final optimal values for the three parameters along with the two parameters *C* and γ in SVM (see Equation 12) were defined by the highest overall accuracy after trying all the 800 combination cases for each of the two predictors in Equation 13, as given by

$$\{\begin{array}{ll} n=2,\lambda =13,w=0.5,C=8,\gamma =2_{}^{-5} & \text{for iMcRNA-PseSSC}\\ n=1,\lambda =17,w=0.2,C=128,\gamma =2_{}^{-7} & \text{for iMcRNA-ExPseSSC} \end{array}$$ (16)

Thus, the parameters in Equation 16 were used to perform the rigorous jackknife test on the benchmark dataset to calculate the metrics defined in Equation 14.

The results thus obtained by the two new predictors are given in Table 1, from which we can see that the overall accuracy (Acc) achieved by iMcRNA-PseSSC was 85.76% with the Matthews correlation coefficient (MCC) equal to 0.72. The corresponding rates achieved by iMcRNA-ExPseSSC were even better; i.e., 89.86% and 0.80 for Acc and MCC, respectively. It is not surprising because the additional features counted in Equation 11 play a complementary role to the feature in Equation 9. In other words, all these features are complementary with each other: PseSSC is a structure-based feature reflecting the global or long-range structure-order effects; MFE and *P*-value are for the secondary structure state of minimum free energy; and tri-nucleobase composition is for the local or short-range sequence order information [47].

**Table 1:** Comparison of different predictors by the jackknife tests on a same benchmark dataset (S1 Dataset).

Method	Acc (%)	MCC	Sn (%)	Sp (%)
iMcRNA-PseSSC^a^	85.76	0.72	88.36	83.50
iMcRNA-ExPseSSC^b^	89.86	0.80	89.93	89.78
Triplet-SVM^c^	81.85	0.64	78.47	85.20
MiPred^d^	87.30	0.75	84.00	90.60

^a^The parameters used: *n* = 2, λ = 13, *w* = 0.5, *C* = 8, and γ = 2. γ = 2^-5^.

^b^The parameters used: *n* = 1, λ = 17, *w* = 0.2, *C* = 128, and γ = 2^-7^.

^c^Results obtained by in-house implementation from [16].

^d^Results obtained by in-house implementation from [21].

### 2. Comparison with Other Methods

We have also made a comparison of the current iMcRNA-PseSSC and iMcRNA-ExPseSSC (Equation 13) with Triplet-SVM [16] and MiPred [21], two of the best existing predictors in this area. As mentioned in the Introduction section, the accuracy rates by the two predictors as originally reported [16,21] were based on small benchmark datasets without removing high similarity or redundant RNA sequences, and hence the rates thus obtained might be over-estimated.

For instance, Triplet-SVM [16] was trained with 163 human pre-miRNAs and 168 false pre-miRNAs, and tested with only 30 human pre-miRNAs and 1,000 false pre-miRNAs. Also, MiPred [21] was trained using the same dataset as used by Triplet-SVM [16] and tested with 263 human pre-miRNAs and 265 false pre-miRNAs. In contrast, the current predictors iMcRNA-PseSSC and iMcRNA-ExPseSSC were trained and tested on a much larger and more stringent benchmark dataset that contained 1,612 human pre-miRNAs and 1,612 false pre-miRNAs in which none had more than 80% pairwise sequence identity to any other.

If using the larger and more stringent benchmark dataset (S1 Dataset) to examine the two predictors via the rigorous jackknife tests, we obtained the corresponding results as given in Table 1

**Fig 5:**
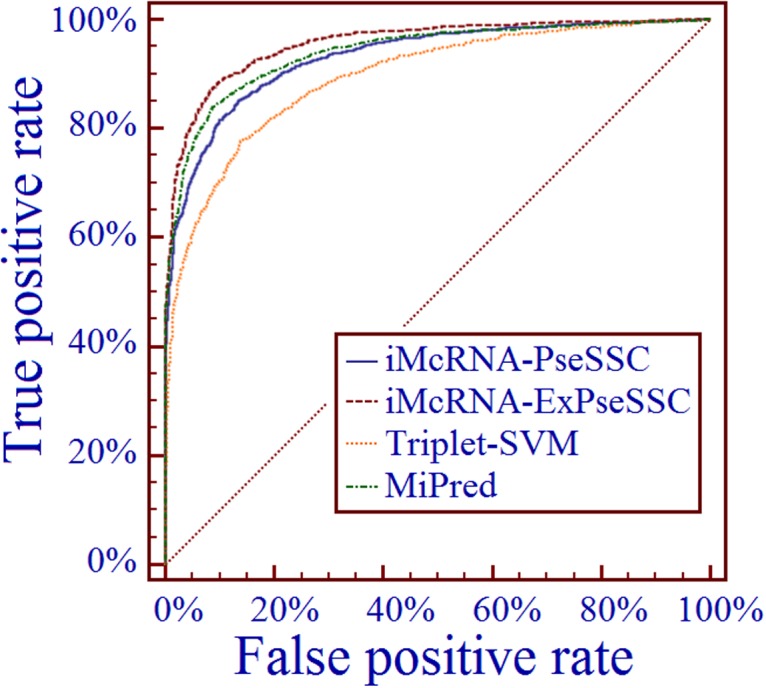
A graphical illustration to show the performance of different methods by means of the receiver operating characteristic (ROC) curves. The areas under the ROC curves, or AUC are 0.93, 0.96, 0.90, and 0.94 for iMcRNA-PseSSC, iMcRNA-ExPseSSC, Triplet-SVM, and MiPred, respectively. See section “Comparison with Other Methods” for further explanation.

Furthermore, to provide a graphic illustration to show the performances of the four predictors, the corresponding ROC (receiver operating characteristic) curves were drawn in Fig. 5, where the horizontal coordinate X is for the false positive rate or 1-Sp, and the vertical coordinate Y is for the true positive rate or Sn. The best possible predictor should yield a point with the coordinate (0, 1) meaning 0 false positive rate (or 100% specificity), and 100% true positive rate or sensitivity Sn. Therefore, the (0,1) point is also called a perfect classification. A completely random guess would give a point along a diagonal from the point (0,0) to (1,1). The area under the ROC curve is called AUC, which is often used to indicate the performance quality of a binary classification predictor: the larger the area, the better the prediction quality is.

From Table 1 and Fig. 5 we can clearly observe the following. (i) The predictor iMcRNA-PseSSC outperformed Triplet-SVM [16] and was highly comparable with MiPred [21], meaning that the prediction quality can be enhanced to the level of the existing best predictor by only taking into account the long-range or global secondary structure sequence order information. (ii) The predictor iMcRNA-ExPseSSC outperformed all its counterparts, meaning that the prediction quality can be further enhanced by combing the aforementioned long-range information with the local features as used in the existing predictors [16,21].

### 3. Discriminant Visualization and Interpretation

**Fig 6:**
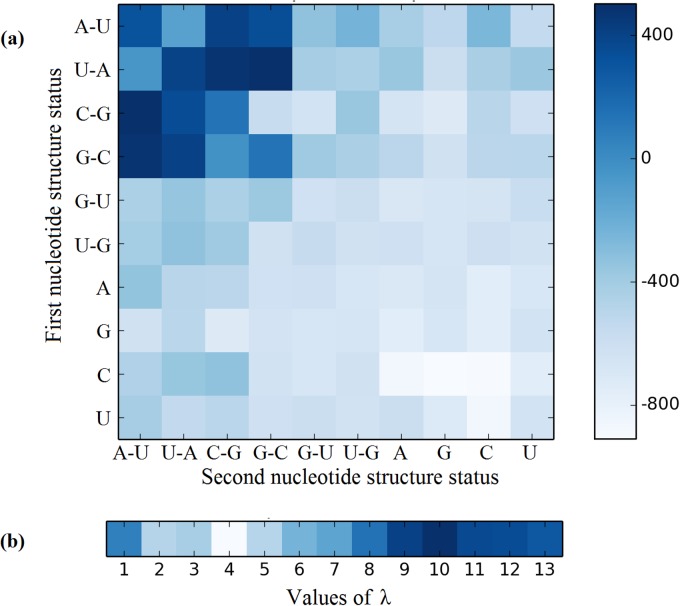
Visualizing the discriminative power with a heat map. (a) The discriminative power of the 100 local structure status compositions. The structure statuses marked on the vertical and horizontal axes indicate the first structure status and the second structure status in the local structure status compositions. (b) The discriminative power of the 13 features incorporating the structure-order effect. The λ values are marked on horizontal axis.

Why was the current approach able to enhance the success rates so remarkably? To address this problem, we are to carry out a graphical analysis. It can provide an intuitive picture or useful insights for helping understand varieties of complicated relations, as demonstrated by many previous studies on a series of important biological topics, such as using graphical rules to study enzyme-catalyzed reactions [77,78], inhibition of HIV-1 reverse transcriptase [79], and drug metabolism systems [80]; using the “cellular automaton image” [81] to study hepatitis B viral infections [82] and HBV virus gene missense mutation [83]; and using wenxiang diagram or graph [84,85] to study protein-protein interactions [86,87]. Here, we used the heat map [88] to present an intuitive analysis. Similar to the approach in [89], we calculated the discriminant weight vector in the feature space of iMcRNA-PseSSC. The results thus obtained are illustrated in Fig. 6a, where the darker the spot is, the more discriminative power the corresponding structure status has. Thus, according to the degree of dark colour in the subfigure, we can see that the statuses of the four structures (A-U, U-A, C-G, G-C) are more important than the others in identifying human microRNA precursors because they have stronger discriminative power. Moreover, the discriminative powers of the 13 features incorporating the structure-order effects are shown in Fig. 6b, from which we can see that the discriminative power for miRNAs tends to be stronger with the increasing λ in value, indicating that the long-range or global structure-order effect do have considerable impacts upon the discrimination. That is the main reason why iMcRNA-PseSSC can remarkably outperform its counterparts.

### 4. Web-Server Guide

We have also established a web-server for the two predictors as formulated in Equation 13. Furthermore, for the convenience of the vast majority of experimental scientists, below let us give a step-by-step guide on how to use the web-server to get their desired results without the need to follow the complicated mathematic equations.

**Fig 7:**
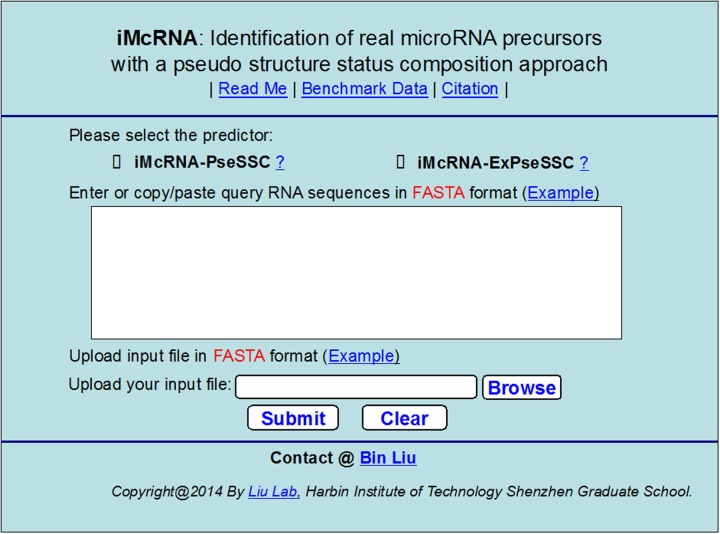
A semi-screenshot to show the top page of the web-server iMcRNA. Its website address is at http://bioinformatics.hitsz.edu.cn/iMcRNA/.

**Step 1**. Open the web-server by clicking the link at http://bioinformatics.hitsz.edu.cn/iMcRNA/ and you will see its top page as shown in Fig. 7. Click on the Read Me button to see a brief introduction about the server that contains two predictors: iMcRNA-PseSSC and iMcRNA-ExPseSSC.

**Step 2**. Check the open circle right in front of iMcRNA-PseSSC or iMcRNA-ExPseSSC to choose which of the two predictors you are to use for prediction.

**Step 3**. You can directly enter the query RNA sequences into the input box at the center of Fig. 7, or use the Browse button to upload them via a file. All the input sequences should be in the FASTA format. A sequence in FASTA format consists of a single initial line beginning with the symbol “>” in the first column, followed by lines of sequence data in which nucleotides are represented using single-letter codes. Except for the mandatory symbol “>”, all the other characters in the single initial line are optional and only used for the purpose of identification and description. The sequence ends if another line starting with the symbol “>” appears; this indicates the start of another sequence. Example sequences in FASTA format can be seen by clicking on the Example button.

**Fig 8:**
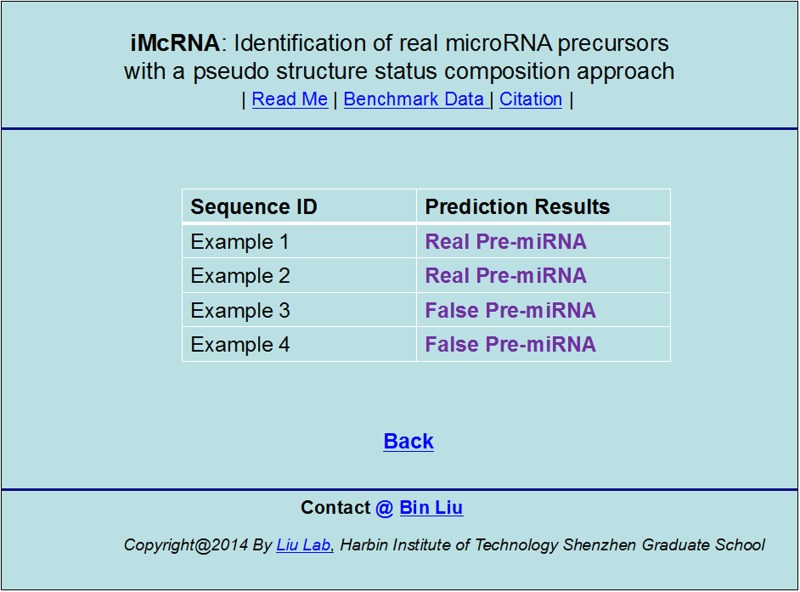
A semi-screenshot to show the output obtained by the web-server. See the text for further explanation.

**Step 4**. Click on the Submit button to see the predicted result. For example, if you use the four query RNA sequences in the Example window as the input and select iMcRNA-PseSSC for prediction, after clicking the Submit button, you will see on your screen (Fig. 8) that the predicted results for the 1^st^ and 2^nd^ query RNA sequences are “**Real Pre-miRNA**”, and that for the 3^rd^ and 4th ones are “**False Pre-miRNA**”. **All these predicted results are fully consistent with the experimental observations**. It takes about 2 seconds for the above computation before the predicted result appears on your computer screen. If you select iMcRNA-ExPseSSC, however, for the same prediction, it may take about 20 seconds because more calculations are needed although the overall success rates thus obtained are generally higher than those by the iMcRNA-PseSSC predictor.

## Conclusion

Based on the concept of pseudo amino acid composition [30] or Chou’s PseAAC [32], two new predictors named iMcRNA-PseSSC and iMcRNA-ExPseSSC were proposed for identifying the human pre-micrRNAs by incorporating the global or long-range structure-order information. It was observed via the rigorous cross-validation on a larger and more stringent newly constructed benchmark dataset that the two new predictors outperformed or were highly comparable with the best existing predictor in this area. The two predictors are publically accessible via a web-server at http://bioinformatics.hitsz.edu.cn/iMcRNA/, by which users can easily get their desired results without the need to follow the complicated mathematical equations, which were presented in this paper just for the integrity of their development process.

It is instructive to point out that although the current two predictors were established for identifying the human pre-micrRNAs, they can be easily used to identify the pre-micrRNAs in any other organism as well if a corresponding benchmark dataset is available.

## Supporting Information

S1 Dataset The benchmark dataset. It contains 3,224 human pre-miRNAs, of which 1,612 are real pre-miRNAs and 1,612 are false pre-miRNAs. None of the sequences included has ≥80% pairwise sequence identity with any other.
(DOC)
