# Supplementary material for: Identification of Real MicroRNA Precursors with a Pseudo Structure Status Composition Approach
Source: PLoS One. 2015 Mar 30;10(3):e0121501. doi: 10.1371/journal.pone.0121501 (PMC4378912; doi:10.1371/journal.pone.0121501)
Supplement: S1 Dataset — It contains 3,224 human pre-miRNAs, of which 1,612 are real pre-miRNAs and 1,612 are false pre-miRNAs. None of the sequences included has ≥80% pairwise sequence identity with any other. (DOC) [file pone.0121501.s001.doc]

**S1 Dataset**. **The benchmark dataset**. It contains 3,224 human pre-miRNAs, of which 1,612 are real pre-miRNAs and 1,612 are false pre-miRNAs. None of the sequences included has $\geq80\%$ pairwise sequence identity with any other.

(1) 1612 real human pre-miRNAs

>hsa-let-7c MI0000064

GCAUCCGGGUUGAGGUAGUAGGUUGUAUGGUUUAGAGUUACACCCUGGGAGUUAACUGUACAACCUUCUAGCUUUCCUUGGAGC

>hsa-let-7d MI0000065

CCUAGGAAGAGGUAGUAGGUUGCAUAGUUUUAGGGCAGGGAUUUUGCCCACAAGGAGGUAACUAUACGACCUGCUGCCUUUCUUAGG

>hsa-let-7e MI0000066

CCCGGGCUGAGGUAGGAGGUUGUAUAGUUGAGGAGGACACCCAAGGAGAUCACUAUACGGCCUCCUAGCUUUCCCCAGG

>hsa-let-7f-1 MI0000067

UCAGAGUGAGGUAGUAGAUUGUAUAGUUGUGGGGUAGUGAUUUUACCCUGUUCAGGAGAUAACUAUACAAUCUAUUGCCUUCCCUGA

>hsa-let-7f-2 MI0000068

UGUGGGAUGAGGUAGUAGAUUGUAUAGUUUUAGGGUCAUACCCCAUCUUGGAGAUAACUAUACAGUCUACUGUCUUUCCCACG

>hsa-let-7g MI0000433

AGGCUGAGGUAGUAGUUUGUACAGUUUGAGGGUCUAUGAUACCACCCGGUACAGGAGAUAACUGUACAGGCCACUGCCUUGCCA

>hsa-let-7i MI0000434

CUGGCUGAGGUAGUAGUUUGUGCUGUUGGUCGGGUUGUGACAUUGCCCGCUGUGGAGAUAACUGCGCAAGCUACUGCCUUGCUA

>hsa-mir-1-2 MI0000437

ACCUACUCAGAGUACAUACUUCUUUAUGUACCCAUAUGAACAUACAAUGCUAUGGAAUGUAAAGAAGUAUGUAUUUUUGGUAGGC

>hsa-mir-7-1 MI0000263

UUGGAUGUUGGCCUAGUUCUGUGUGGAAGACUAGUGAUUUUGUUGUUUUUAGAUAACUAAAUCGACAACAAAUCACAGUCUGCCAUAUGGCACAGGCCAUGCCUCUACAG

>hsa-mir-7-2 MI0000264

CUGGAUACAGAGUGGACCGGCUGGCCCCAUCUGGAAGACUAGUGAUUUUGUUGUUGUCUUACUGCGCUCAACAACAAAUCCCAGUCUACCUAAUGGUGCCAGCCAUCGCA

>hsa-mir-7-3 MI0000265

AGAUUAGAGUGGCUGUGGUCUAGUGCUGUGUGGAAGACUAGUGAUUUUGUUGUUCUGAUGUACUACGACAACAAGUCACAGCCGGCCUCAUAGCGCAGACUCCCUUCGAC

>hsa-mir-9-1 MI0000466

CGGGGUUGGUUGUUAUCUUUGGUUAUCUAGCUGUAUGAGUGGUGUGGAGUCUUCAUAAAGCUAGAUAACCGAAAGUAAAAAUAACCCCA

>hsa-mir-9-3 MI0000468

GGAGGCCCGUUUCUCUCUUUGGUUAUCUAGCUGUAUGAGUGCCACAGAGCCGUCAUAAAGCUAGAUAACCGAAAGUAGAAAUGAUUCUCA

>hsa-mir-10a MI0000266

GAUCUGUCUGUCUUCUGUAUAUACCCUGUAGAUCCGAAUUUGUGUAAGGAAUUUUGUGGUCACAAAUUCGUAUCUAGGGGAAUAUGUAGUUGACAUAAACACUCCGCUCU

>hsa-mir-10b MI0000267

CCAGAGGUUGUAACGUUGUCUAUAUAUACCCUGUAGAACCGAAUUUGUGUGGUAUCCGUAUAGUCACAGAUUCGAUUCUAGGGGAAUAUAUGGUCGAUGCAAAAACUUCA

>hsa-mir-15a MI0000069

CCUUGGAGUAAAGUAGCAGCACAUAAUGGUUUGUGGAUUUUGAAAAGGUGCAGGCCAUAUUGUGCUGCCUCAAAAAUACAAGG

>hsa-mir-15b MI0000438

UUGAGGCCUUAAAGUACUGUAGCAGCACAUCAUGGUUUACAUGCUACAGUCAAGAUGCGAAUCAUUAUUUGCUGCUCUAGAAAUUUAAGGAAAUUCAU

>hsa-mir-16-1 MI0000070

GUCAGCAGUGCCUUAGCAGCACGUAAAUAUUGGCGUUAAGAUUCUAAAAUUAUCUCCAGUAUUAACUGUGCUGCUGAAGUAAGGUUGAC

>hsa-mir-16-2 MI0000115

GUUCCACUCUAGCAGCACGUAAAUAUUGGCGUAGUGAAAUAUAUAUUAAACACCAAUAUUACUGUGCUGCUUUAGUGUGAC

>hsa-mir-17 MI0000071

GUCAGAAUAAUGUCAAAGUGCUUACAGUGCAGGUAGUGAUAUGUGCAUCUACUGCAGUGAAGGCACUUGUAGCAUUAUGGUGAC

>hsa-mir-18a MI0000072

UGUUCUAAGGUGCAUCUAGUGCAGAUAGUGAAGUAGAUUAGCAUCUACUGCCCUAAGUGCUCCUUCUGGCA

>hsa-mir-19a MI0000073

GCAGUCCUCUGUUAGUUUUGCAUAGUUGCACUACAAGAAGAAUGUAGUUGUGCAAAUCUAUGCAAAACUGAUGGUGGCCUGC

>hsa-mir-19b-2 MI0000075

ACAUUGCUACUUACAAUUAGUUUUGCAGGUUUGCAUUUCAGCGUAUAUAUGUAUAUGUGGCUGUGCAAAUCCAUGCAAAACUGAUUGUGAUAAUGU

>hsa-mir-20a MI0000076

GUAGCACUAAAGUGCUUAUAGUGCAGGUAGUGUUUAGUUAUCUACUGCAUUAUGAGCACUUAAAGUACUGC

>hsa-mir-20b MI0001519

AGUACCAAAGUGCUCAUAGUGCAGGUAGUUUUGGCAUGACUCUACUGUAGUAUGGGCACUUCCAGUACU

>hsa-mir-21 MI0000077

UGUCGGGUAGCUUAUCAGACUGAUGUUGACUGUUGAAUCUCAUGGCAACACCAGUCGAUGGGCUGUCUGACA

>hsa-mir-22 MI0000078

GGCUGAGCCGCAGUAGUUCUUCAGUGGCAAGCUUUAUGUCCUGACCCAGCUAAAGCUGCCAGUUGAAGAACUGUUGCCCUCUGCC

>hsa-mir-23a MI0000079

GGCCGGCUGGGGUUCCUGGGGAUGGGAUUUGCUUCCUGUCACAAAUCACAUUGCCAGGGAUUUCCAACCGACC

>hsa-mir-23b MI0000439

CUCAGGUGCUCUGGCUGCUUGGGUUCCUGGCAUGCUGAUUUGUGACUUAAGAUUAAAAUCACAUUGCCAGGGAUUACCACGCAACCACGACCUUGGC

>hsa-mir-23c MI0016010

AGUGACUUUCCAGGUGUCACACAGUGAGUGGCAUAAUCAGAGUACAAUUUGAGUCAUGCCCAUACAUCACAUUGCCAGUGAUUACCCAAGGAAAGUGACG

>hsa-mir-24-2 MI0000081

CUCUGCCUCCCGUGCCUACUGAGCUGAAACACAGUUGGUUUGUGUACACUGGCUCAGUUCAGCAGGAACAGGG

>hsa-mir-25 MI0000082

GGCCAGUGUUGAGAGGCGGAGACUUGGGCAAUUGCUGGACGCUGCCCUGGGCAUUGCACUUGUCUCGGUCUGACAGUGCCGGCC

>hsa-mir-26a-1 MI0000083

GUGGCCUCGUUCAAGUAAUCCAGGAUAGGCUGUGCAGGUCCCAAUGGGCCUAUUCUUGGUUACUUGCACGGGGACGC

>hsa-mir-26a-2 MI0000750

GGCUGUGGCUGGAUUCAAGUAAUCCAGGAUAGGCUGUUUCCAUCUGUGAGGCCUAUUCUUGAUUACUUGUUUCUGGAGGCAGCU

>hsa-mir-26b MI0000084

CCGGGACCCAGUUCAAGUAAUUCAGGAUAGGUUGUGUGCUGUCCAGCCUGUUCUCCAUUACUUGGCUCGGGGACCGG

>hsa-mir-27a MI0000085

CUGAGGAGCAGGGCUUAGCUGCUUGUGAGCAGGGUCCACACCAAGUCGUGUUCACAGUGGCUAAGUUCCGCCCCCCAG

>hsa-mir-27b MI0000440

ACCUCUCUAACAAGGUGCAGAGCUUAGCUGAUUGGUGAACAGUGAUUGGUUUCCGCUUUGUUCACAGUGGCUAAGUUCUGCACCUGAAGAGAAGGUG

>hsa-mir-28 MI0000086

GGUCCUUGCCCUCAAGGAGCUCACAGUCUAUUGAGUUACCUUUCUGACUUUCCCACUAGAUUGUGAGCUCCUGGAGGGCAGGCACU

>hsa-mir-29b-1 MI0000105

CUUCAGGAAGCUGGUUUCAUAUGGUGGUUUAGAUUUAAAUAGUGAUUGUCUAGCACCAUUUGAAAUCAGUGUUCUUGGGGG

>hsa-mir-29c MI0000735

AUCUCUUACACAGGCUGACCGAUUUCUCCUGGUGUUCAGAGUCUGUUUUUGUCUAGCACCAUUUGAAAUCGGUUAUGAUGUAGGGGGA

>hsa-mir-30b MI0000441

ACCAAGUUUCAGUUCAUGUAAACAUCCUACACUCAGCUGUAAUACAUGGAUUGGCUGGGAGGUGGAUGUUUACUUCAGCUGACUUGGA

>hsa-mir-30c-1 MI0000736

ACCAUGCUGUAGUGUGUGUAAACAUCCUACACUCUCAGCUGUGAGCUCAAGGUGGCUGGGAGAGGGUUGUUUACUCCUUCUGCCAUGGA

>hsa-mir-30d MI0000255

GUUGUUGUAAACAUCCCCGACUGGAAGCUGUAAGACACAGCUAAGCUUUCAGUCAGAUGUUUGCUGCUAC

>hsa-mir-30e MI0000749

GGGCAGUCUUUGCUACUGUAAACAUCCUUGACUGGAAGCUGUAAGGUGUUCAGAGGAGCUUUCAGUCGGAUGUUUACAGCGGCAGGCUGCCA

>hsa-mir-31 MI0000089

GGAGAGGAGGCAAGAUGCUGGCAUAGCUGUUGAACUGGGAACCUGCUAUGCCAACAUAUUGCCAUCUUUCC

>hsa-mir-32 MI0000090

GGAGAUAUUGCACAUUACUAAGUUGCAUGUUGUCACGGCCUCAAUGCAAUUUAGUGUGUGUGAUAUUUUC

>hsa-mir-33a MI0000091

CUGUGGUGCAUUGUAGUUGCAUUGCAUGUUCUGGUGGUACCCAUGCAAUGUUUCCACAGUGCAUCACAG

>hsa-mir-33b MI0003646

GCGGGCGGCCCCGCGGUGCAUUGCUGUUGCAUUGCACGUGUGUGAGGCGGGUGCAGUGCCUCGGCAGUGCAGCCCGGAGCCGGCCCCUGGCACCAC

>hsa-mir-34a MI0000268

GGCCAGCUGUGAGUGUUUCUUUGGCAGUGUCUUAGCUGGUUGUUGUGAGCAAUAGUAAGGAAGCAAUCAGCAAGUAUACUGCCCUAGAAGUGCUGCACGUUGUGGGGCCC

>hsa-mir-34b MI0000742

GUGCUCGGUUUGUAGGCAGUGUCAUUAGCUGAUUGUACUGUGGUGGUUACAAUCACUAACUCCACUGCCAUCAAAACAAGGCAC

>hsa-mir-34c MI0000743

AGUCUAGUUACUAGGCAGUGUAGUUAGCUGAUUGCUAAUAGUACCAAUCACUAACCACACGGCCAGGUAAAAAGAUU

>hsa-mir-92a-1 MI0000093

CUUUCUACACAGGUUGGGAUCGGUUGCAAUGCUGUGUUUCUGUAUGGUAUUGCACUUGUCCCGGCCUGUUGAGUUUGG

>hsa-mir-92a-2 MI0000094

UCAUCCCUGGGUGGGGAUUUGUUGCAUUACUUGUGUUCUAUAUAAAGUAUUGCACUUGUCCCGGCCUGUGGAAGA

>hsa-mir-92b MI0003560

CGGGCCCCGGGCGGGCGGGAGGGACGGGACGCGGUGCAGUGUUGUUUUUUCCCCCGCCAAUAUUGCACUCGUCCCGGCCUCCGGCCCCCCCGGCCC

>hsa-mir-93 MI0000095

CUGGGGGCUCCAAAGUGCUGUUCGUGCAGGUAGUGUGAUUACCCAACCUACUGCUGAGCUAGCACUUCCCGAGCCCCCGG

>hsa-mir-95 MI0000097

AACACAGUGGGCACUCAAUAAAUGUCUGUUGAAUUGAAAUGCGUUACAUUCAACGGGUAUUUAUUGAGCACCCACUCUGUG

>hsa-mir-96 MI0000098

UGGCCGAUUUUGGCACUAGCACAUUUUUGCUUGUGUCUCUCCGCUCUGAGCAAUCAUGUGCAGUGCCAAUAUGGGAAA

>hsa-mir-98 MI0000100

AGGAUUCUGCUCAUGCCAGGGUGAGGUAGUAAGUUGUAUUGUUGUGGGGUAGGGAUAUUAGGCCCCAAUUAGAAGAUAACUAUACAACUUACUACUUUCCCUGGUGUGUGGCAUAUUCA

>hsa-mir-99a MI0000101

CCCAUUGGCAUAAACCCGUAGAUCCGAUCUUGUGGUGAAGUGGACCGCACAAGCUCGCUUCUAUGGGUCUGUGUCAGUGUG

>hsa-mir-99b MI0000746

GGCACCCACCCGUAGAACCGACCUUGCGGGGCCUUCGCCGCACACAAGCUCGUGUCUGUGGGUCCGUGUC

>hsa-mir-100 MI0000102

CCUGUUGCCACAAACCCGUAGAUCCGAACUUGUGGUAUUAGUCCGCACAAGCUUGUAUCUAUAGGUAUGUGUCUGUUAGG

>hsa-mir-101-1 MI0000103

UGCCCUGGCUCAGUUAUCACAGUGCUGAUGCUGUCUAUUCUAAAGGUACAGUACUGUGAUAACUGAAGGAUGGCA

>hsa-mir-101-2 MI0000739

ACUGUCCUUUUUCGGUUAUCAUGGUACCGAUGCUGUAUAUCUGAAAGGUACAGUACUGUGAUAACUGAAGAAUGGUGGU

>hsa-mir-103b-1 MI0007261

UCAUAGCCCUGUACAAUGCUGCUUGAUCCAUAUGCAACAAGGCAGCACUGUAAAGAAGCCGA

>hsa-mir-105-1 MI0000111

UGUGCAUCGUGGUCAAAUGCUCAGACUCCUGUGGUGGCUGCUCAUGCACCACGGAUGUUUGAGCAUGUGCUACGGUGUCUA

>hsa-mir-106a MI0000113

CCUUGGCCAUGUAAAAGUGCUUACAGUGCAGGUAGCUUUUUGAGAUCUACUGCAAUGUAAGCACUUCUUACAUUACCAUGG

>hsa-mir-106b MI0000734

CCUGCCGGGGCUAAAGUGCUGACAGUGCAGAUAGUGGUCCUCUCCGUGCUACCGCACUGUGGGUACUUGCUGCUCCAGCAGG

>hsa-mir-107 MI0000114

CUCUCUGCUUUCAGCUUCUUUACAGUGUUGCCUUGUGGCAUGGAGUUCAAGCAGCAUUGUACAGGGCUAUCAAAGCACAGA

>hsa-mir-122 MI0000442

CCUUAGCAGAGCUGUGGAGUGUGACAAUGGUGUUUGUGUCUAAACUAUCAAACGCCAUUAUCACACUAAAUAGCUACUGCUAGGC

>hsa-mir-124-2 MI0000444

AUCAAGAUUAGAGGCUCUGCUCUCCGUGUUCACAGCGGACCUUGAUUUAAUGUCAUACAAUUAAGGCACGCGGUGAAUGCCAAGAGCGGAGCCUACGGCUGCACUUGAA

>hsa-mir-125a MI0000469

UGCCAGUCUCUAGGUCCCUGAGACCCUUUAACCUGUGAGGACAUCCAGGGUCACAGGUGAGGUUCUUGGGAGCCUGGCGUCUGGCC

>hsa-mir-125b-1 MI0000446

UGCGCUCCUCUCAGUCCCUGAGACCCUAACUUGUGAUGUUUACCGUUUAAAUCCACGGGUUAGGCUCUUGGGAGCUGCGAGUCGUGCU

>hsa-mir-125b-2 MI0000470

ACCAGACUUUUCCUAGUCCCUGAGACCCUAACUUGUGAGGUAUUUUAGUAACAUCACAAGUCAGGCUCUUGGGACCUAGGCGGAGGGGA

>hsa-mir-126 MI0000471

CGCUGGCGACGGGACAUUAUUACUUUUGGUACGCGCUGUGACACUUCAAACUCGUACCGUGAGUAAUAAUGCGCCGUCCACGGCA

>hsa-mir-127 MI0000472

UGUGAUCACUGUCUCCAGCCUGCUGAAGCUCAGAGGGCUCUGAUUCAGAAAGAUCAUCGGAUCCGUCUGAGCUUGGCUGGUCGGAAGUCUCAUCAUC

>hsa-mir-128-1 MI0000447

UGAGCUGUUGGAUUCGGGGCCGUAGCACUGUCUGAGAGGUUUACAUUUCUCACAGUGAACCGGUCUCUUUUUCAGCUGCUUC

>hsa-mir-128-2 MI0000727

UGUGCAGUGGGAAGGGGGGCCGAUACACUGUACGAGAGUGAGUAGCAGGUCUCACAGUGAACCGGUCUCUUUCCCUACUGUGUC

>hsa-mir-129-2 MI0000473

UGCCCUUCGCGAAUCUUUUUGCGGUCUGGGCUUGCUGUACAUAACUCAAUAGCCGGAAGCCCUUACCCCAAAAAGCAUUUGCGGAGGGCG

>hsa-mir-130a MI0000448

UGCUGCUGGCCAGAGCUCUUUUCACAUUGUGCUACUGUCUGCACCUGUCACUAGCAGUGCAAUGUUAAAAGGGCAUUGGCCGUGUAGUG

>hsa-mir-130b MI0000748

GGCCUGCCCGACACUCUUUCCCUGUUGCACUACUAUAGGCCGCUGGGAAGCAGUGCAAUGAUGAAAGGGCAUCGGUCAGGUC

>hsa-mir-132 MI0000449

CCGCCCCCGCGUCUCCAGGGCAACCGUGGCUUUCGAUUGUUACUGUGGGAACUGGAGGUAACAGUCUACAGCCAUGGUCGCCCCGCAGCACGCCCACGCGC

>hsa-mir-133a-2 MI0000451

GGGAGCCAAAUGCUUUGCUAGAGCUGGUAAAAUGGAACCAAAUCGACUGUCCAAUGGAUUUGGUCCCCUUCAACCAGCUGUAGCUGUGCAUUGAUGGCGCCG

>hsa-mir-133b MI0000822

CCUCAGAAGAAAGAUGCCCCCUGCUCUGGCUGGUCAAACGGAACCAAGUCCGUCUUCCUGAGAGGUUUGGUCCCCUUCAACCAGCUACAGCAGGGCUGGCAAUGCCCAGUCCUUGGAGA

>hsa-mir-134 MI0000474

CAGGGUGUGUGACUGGUUGACCAGAGGGGCAUGCACUGUGUUCACCCUGUGGGCCACCUAGUCACCAACCCUC

>hsa-mir-135a-1 MI0000452

AGGCCUCGCUGUUCUCUAUGGCUUUUUAUUCCUAUGUGAUUCUACUGCUCACUCAUAUAGGGAUUGGAGCCGUGGCGCACGGCGGGGACA

>hsa-mir-135a-2 MI0000453

AGAUAAAUUCACUCUAGUGCUUUAUGGCUUUUUAUUCCUAUGUGAUAGUAAUAAAGUCUCAUGUAGGGAUGGAAGCCAUGAAAUACAUUGUGAAAAAUCA

>hsa-mir-135b MI0000810

CACUCUGCUGUGGCCUAUGGCUUUUCAUUCCUAUGUGAUUGCUGUCCCAAACUCAUGUAGGGCUAAAAGCCAUGGGCUACAGUGAGGGGCGAGCUCC

>hsa-mir-136 MI0000475

UGAGCCCUCGGAGGACUCCAUUUGUUUUGAUGAUGGAUUCUUAUGCUCCAUCAUCGUCUCAAAUGAGUCUUCAGAGGGUUCU

>hsa-mir-137 MI0000454

GGUCCUCUGACUCUCUUCGGUGACGGGUAUUCUUGGGUGGAUAAUACGGAUUACGUUGUUAUUGCUUAAGAAUACGCGUAGUCGAGGAGAGUACCAGCGGCA

>hsa-mir-138-1 MI0000476

CCCUGGCAUGGUGUGGUGGGGCAGCUGGUGUUGUGAAUCAGGCCGUUGCCAAUCAGAGAACGGCUACUUCACAACACCAGGGCCACACCACACUACAGG

>hsa-mir-138-2 MI0000455

CGUUGCUGCAGCUGGUGUUGUGAAUCAGGCCGACGAGCAGCGCAUCCUCUUACCCGGCUAUUUCACGACACCAGGGUUGCAUCA

>hsa-mir-139 MI0000261

GUGUAUUCUACAGUGCACGUGUCUCCAGUGUGGCUCGGAGGCUGGAGACGCGGCCCUGUUGGAGUAAC

>hsa-mir-140 MI0000456

UGUGUCUCUCUCUGUGUCCUGCCAGUGGUUUUACCCUAUGGUAGGUUACGUCAUGCUGUUCUACCACAGGGUAGAACCACGGACAGGAUACCGGGGCACC

>hsa-mir-141 MI0000457

CGGCCGGCCCUGGGUCCAUCUUCCAGUACAGUGUUGGAUGGUCUAAUUGUGAAGCUCCUAACACUGUCUGGUAAAGAUGGCUCCCGGGUGGGUUC

>hsa-mir-142 MI0000458

GACAGUGCAGUCACCCAUAAAGUAGAAAGCACUACUAACAGCACUGGAGGGUGUAGUGUUUCCUACUUUAUGGAUGAGUGUACUGUG

>hsa-mir-143 MI0000459

GCGCAGCGCCCUGUCUCCCAGCCUGAGGUGCAGUGCUGCAUCUCUGGUCAGUUGGGAGUCUGAGAUGAAGCACUGUAGCUCAGGAAGAGAGAAGUUGUUCUGCAGC

>hsa-mir-144 MI0000460

UGGGGCCCUGGCUGGGAUAUCAUCAUAUACUGUAAGUUUGCGAUGAGACACUACAGUAUAGAUGAUGUACUAGUCCGGGCACCCCC

>hsa-mir-145 MI0000461

CACCUUGUCCUCACGGUCCAGUUUUCCCAGGAAUCCCUUAGAUGCUAAGAUGGGGAUUCCUGGAAAUACUGUUCUUGAGGUCAUGGUU

>hsa-mir-146a MI0000477

CCGAUGUGUAUCCUCAGCUUUGAGAACUGAAUUCCAUGGGUUGUGUCAGUGUCAGACCUCUGAAAUUCAGUUCUUCAGCUGGGAUAUCUCUGUCAUCGU

>hsa-mir-146b MI0003129

CCUGGCACUGAGAACUGAAUUCCAUAGGCUGUGAGCUCUAGCAAUGCCCUGUGGACUCAGUUCUGGUGCCCGG

>hsa-mir-147b MI0005544

UAUAAAUCUAGUGGAAACAUUUCUGCACAAACUAGAUUCUGGACACCAGUGUGCGGAAAUGCUUCUGCUACAUUUUUAGG

>hsa-mir-148a MI0000253

GAGGCAAAGUUCUGAGACACUCCGACUCUGAGUAUGAUAGAAGUCAGUGCACUACAGAACUUUGUCUC

>hsa-mir-148b MI0000811

CAAGCACGAUUAGCAUUUGAGGUGAAGUUCUGUUAUACACUCAGGCUGUGGCUCUCUGAAAGUCAGUGCAUCACAGAACUUUGUCUCGAAAGCUUUCUA

>hsa-mir-149 MI0000478

GCCGGCGCCCGAGCUCUGGCUCCGUGUCUUCACUCCCGUGCUUGUCCGAGGAGGGAGGGAGGGACGGGGGCUGUGCUGGGGCAGCUGGA

>hsa-mir-150 MI0000479

CUCCCCAUGGCCCUGUCUCCCAACCCUUGUACCAGUGCUGGGCUCAGACCCUGGUACAGGCCUGGGGGACAGGGACCUGGGGAC

>hsa-mir-151a MI0000809

UUUCCUGCCCUCGAGGAGCUCACAGUCUAGUAUGUCUCAUCCCCUACUAGACUGAAGCUCCUUGAGGACAGGGAUGGUCAUACUCACCUC

>hsa-mir-151b MI0003772

ACCUCUGAUGUGUCAGUCUCUCUUCAGGGCUCCCGAGACACAGAAACAGACACCUGCCCUCGAGGAGCUCACAGUCUAGACAAACAAACCCAGGGU

>hsa-mir-152 MI0000462

UGUCCCCCCCGGCCCAGGUUCUGUGAUACACUCCGACUCGGGCUCUGGAGCAGUCAGUGCAUGACAGAACUUGGGCCCGGAAGGACC

>hsa-mir-153-1 MI0000463

CUCACAGCUGCCAGUGUCAUUUUUGUGAUCUGCAGCUAGUAUUCUCACUCCAGUUGCAUAGUCACAAAAGUGAUCAUUGGCAGGUGUGGC

>hsa-mir-154 MI0000480

GUGGUACUUGAAGAUAGGUUAUCCGUGUUGCCUUCGCUUUAUUUGUGACGAAUCAUACACGGUUGACCUAUUUUUCAGUACCAA

>hsa-mir-155 MI0000681

CUGUUAAUGCUAAUCGUGAUAGGGGUUUUUGCCUCCAACUGACUCCUACAUAUUAGCAUUAACAG

>hsa-mir-181a-1 MI0000289

UGAGUUUUGAGGUUGCUUCAGUGAACAUUCAACGCUGUCGGUGAGUUUGGAAUUAAAAUCAAAACCAUCGACCGUUGAUUGUACCCUAUGGCUAACCAUCAUCUACUCCA

>hsa-mir-181a-2 MI0000269

AGAAGGGCUAUCAGGCCAGCCUUCAGAGGACUCCAAGGAACAUUCAACGCUGUCGGUGAGUUUGGGAUUUGAAAAAACCACUGACCGUUGACUGUACCUUGGGGUCCUUA

>hsa-mir-181b-1 MI0000270

CCUGUGCAGAGAUUAUUUUUUAAAAGGUCACAAUCAACAUUCAUUGCUGUCGGUGGGUUGAACUGUGUGGACAAGCUCACUGAACAAUGAAUGCAACUGUGGCCCCGCUU

>hsa-mir-181b-2 MI0000683

CUGAUGGCUGCACUCAACAUUCAUUGCUGUCGGUGGGUUUGAGUCUGAAUCAACUCACUGAUCAAUGAAUGCAAACUGCGGACCAAACA

>hsa-mir-181c MI0000271

CGGAAAAUUUGCCAAGGGUUUGGGGGAACAUUCAACCUGUCGGUGAGUUUGGGCAGCUCAGGCAAACCAUCGACCGUUGAGUGGACCCUGAGGCCUGGAAUUGCCAUCCU

>hsa-mir-181d MI0003139

GUCCCCUCCCCUAGGCCACAGCCGAGGUCACAAUCAACAUUCAUUGUUGUCGGUGGGUUGUGAGGACUGAGGCCAGACCCACCGGGGGAUGAAUGUCACUGUGGCUGGGCCAGACACGGCUUAAGGGGAAUGGGGAC

>hsa-mir-182 MI0000272

GAGCUGCUUGCCUCCCCCCGUUUUUGGCAAUGGUAGAACUCACACUGGUGAGGUAACAGGAUCCGGUGGUUCUAGACUUGCCAACUAUGGGGCGAGGACUCAGCCGGCAC

>hsa-mir-183 MI0000273

CCGCAGAGUGUGACUCCUGUUCUGUGUAUGGCACUGGUAGAAUUCACUGUGAACAGUCUCAGUCAGUGAAUUACCGAAGGGCCAUAAACAGAGCAGAGACAGAUCCACGA

>hsa-mir-184 MI0000481

CCAGUCACGUCCCCUUAUCACUUUUCCAGCCCAGCUUUGUGACUGUAAGUGUUGGACGGAGAACUGAUAAGGGUAGGUGAUUGA

>hsa-mir-185 MI0000482

AGGGGGCGAGGGAUUGGAGAGAAAGGCAGUUCCUGAUGGUCCCCUCCCCAGGGGCUGGCUUUCCUCUGGUCCUUCCCUCCCA

>hsa-mir-186 MI0000483

UGCUUGUAACUUUCCAAAGAAUUCUCCUUUUGGGCUUUCUGGUUUUAUUUUAAGCCCAAAGGUGAAUUUUUUGGGAAGUUUGAGCU

>hsa-mir-187 MI0000274

GGUCGGGCUCACCAUGACACAGUGUGAGACCUCGGGCUACAACACAGGACCCGGGCGCUGCUCUGACCCCUCGUGUCUUGUGUUGCAGCCGGAGGGACGCAGGUCCGCA

>hsa-mir-188 MI0000484

UGCUCCCUCUCUCACAUCCCUUGCAUGGUGGAGGGUGAGCUUUCUGAAAACCCCUCCCACAUGCAGGGUUUGCAGGAUGGCGAGCC

>hsa-mir-190a MI0000486

UGCAGGCCUCUGUGUGAUAUGUUUGAUAUAUUAGGUUGUUAUUUAAUCCAACUAUAUAUCAAACAUAUUCCUACAGUGUCUUGCC

>hsa-mir-190b MI0005545

UGCUUCUGUGUGAUAUGUUUGAUAUUGGGUUGUUUAAUUAGGAACCAACUAAAUGUCAAACAUAUUCUUACAGCAGCAG

>hsa-mir-191 MI0000465

CGGCUGGACAGCGGGCAACGGAAUCCCAAAAGCAGCUGUUGUCUCCAGAGCAUUCCAGCUGCGCUUGGAUUUCGUCCCCUGCUCUCCUGCCU

>hsa-mir-192 MI0000234

GCCGAGACCGAGUGCACAGGGCUCUGACCUAUGAAUUGACAGCCAGUGCUCUCGUCUCCCCUCUGGCUGCCAAUUCCAUAGGUCACAGGUAUGUUCGCCUCAAUGCCAGC

>hsa-mir-193a MI0000487

CGAGGAUGGGAGCUGAGGGCUGGGUCUUUGCGGGCGAGAUGAGGGUGUCGGAUCAACUGGCCUACAAAGUCCCAGUUCUCGGCCCCCG

>hsa-mir-193b MI0003137

GUGGUCUCAGAAUCGGGGUUUUGAGGGCGAGAUGAGUUUAUGUUUUAUCCAACUGGCCCUCAAAGUCCCGCUUUUGGGGUCAU

>hsa-mir-194-1 MI0000488

AUGGUGUUAUCAAGUGUAACAGCAACUCCAUGUGGACUGUGUACCAAUUUCCAGUGGAGAUGCUGUUACUUUUGAUGGUUACCAA

>hsa-mir-194-2 MI0000732

UGGUUCCCGCCCCCUGUAACAGCAACUCCAUGUGGAAGUGCCCACUGGUUCCAGUGGGGCUGCUGUUAUCUGGGGCGAGGGCCAG

>hsa-mir-195 MI0000489

AGCUUCCCUGGCUCUAGCAGCACAGAAAUAUUGGCACAGGGAAGCGAGUCUGCCAAUAUUGGCUGUGCUGCUCCAGGCAGGGUGGUG

>hsa-mir-196a-1 MI0000238

GUGAAUUAGGUAGUUUCAUGUUGUUGGGCCUGGGUUUCUGAACACAACAACAUUAAACCACCCGAUUCAC

>hsa-mir-196a-2 MI0000279

UGCUCGCUCAGCUGAUCUGUGGCUUAGGUAGUUUCAUGUUGUUGGGAUUGAGUUUUGAACUCGGCAACAAGAAACUGCCUGAGUUACAUCAGUCGGUUUUCGUCGAGGGC

>hsa-mir-196b MI0001150

ACUGGUCGGUGAUUUAGGUAGUUUCCUGUUGUUGGGAUCCACCUUUCUCUCGACAGCACGACACUGCCUUCAUUACUUCAGUUG

>hsa-mir-197 MI0000239

GGCUGUGCCGGGUAGAGAGGGCAGUGGGAGGUAAGAGCUCUUCACCCUUCACCACCUUCUCCACCCAGCAUGGCC

>hsa-mir-198 MI0000240

UCAUUGGUCCAGAGGGGAGAUAGGUUCCUGUGAUUUUUCCUUCUUCUCUAUAGAAUAAAUGA

>hsa-mir-199a-2 MI0000281

AGGAAGCUUCUGGAGAUCCUGCUCCGUCGCCCCAGUGUUCAGACUACCUGUUCAGGACAAUGCCGUUGUACAGUAGUCUGCACAUUGGUUAGACUGGGCAAGGGAGAGCA

>hsa-mir-199b MI0000282

CCAGAGGACACCUCCACUCCGUCUACCCAGUGUUUAGACUAUCUGUUCAGGACUCCCAAAUUGUACAGUAGUCUGCACAUUGGUUAGGCUGGGCUGGGUUAGACCCUCGG

>hsa-mir-200a MI0000737

CCGGGCCCCUGUGAGCAUCUUACCGGACAGUGCUGGAUUUCCCAGCUUGACUCUAACACUGUCUGGUAACGAUGUUCAAAGGUGACCCGC

>hsa-mir-200b MI0000342

CCAGCUCGGGCAGCCGUGGCCAUCUUACUGGGCAGCAUUGGAUGGAGUCAGGUCUCUAAUACUGCCUGGUAAUGAUGACGGCGGAGCCCUGCACG

>hsa-mir-200c MI0000650

CCCUCGUCUUACCCAGCAGUGUUUGGGUGCGGUUGGGAGUCUCUAAUACUGCCGGGUAAUGAUGGAGG

>hsa-mir-202 MI0003130

CGCCUCAGAGCCGCCCGCCGUUCCUUUUUCCUAUGCAUAUACUUCUUUGAGGAUCUGGCCUAAAGAGGUAUAGGGCAUGGGAAAACGGGGCGGUCGGGUCCUCCCCAGCG

>hsa-mir-203a MI0000283

GUGUUGGGGACUCGCGCGCUGGGUCCAGUGGUUCUUAACAGUUCAACAGUUCUGUAGCGCAAUUGUGAAAUGUUUAGGACCACUAGACCCGGCGGGCGCGGCGACAGCGA

>hsa-mir-203b MI0017343

GCGCCCGCCGGGUCUAGUGGUCCUAAACAUUUCACAAUUGCGCUACAGAACUGUUGAACUGUUAAGAACCACUGGACCCAGCGCGC

>hsa-mir-204 MI0000284

GGCUACAGUCUUUCUUCAUGUGACUCGUGGACUUCCCUUUGUCAUCCUAUGCCUGAGAAUAUAUGAAGGAGGCUGGGAAGGCAAAGGGACGUUCAAUUGUCAUCACUGGC

>hsa-mir-205 MI0000285

AAAGAUCCUCAGACAAUCCAUGUGCUUCUCUUGUCCUUCAUUCCACCGGAGUCUGUCUCAUACCCAACCAGAUUUCAGUGGAGUGAAGUUCAGGAGGCAUGGAGCUGACA

>hsa-mir-206 MI0000490

UGCUUCCCGAGGCCACAUGCUUCUUUAUAUCCCCAUAUGGAUUACUUUGCUAUGGAAUGUAAGGAAGUGUGUGGUUUCGGCAAGUG

>hsa-mir-208a MI0000251

UGACGGGCGAGCUUUUGGCCCGGGUUAUACCUGAUGCUCACGUAUAAGACGAGCAAAAAGCUUGUUGGUCA

>hsa-mir-208b MI0005570

CCUCUCAGGGAAGCUUUUUGCUCGAAUUAUGUUUCUGAUCCGAAUAUAAGACGAACAAAAGGUUUGUCUGAGGGCAG

>hsa-mir-210 MI0000286

ACCCGGCAGUGCCUCCAGGCGCAGGGCAGCCCCUGCCCACCGCACACUGCGCUGCCCCAGACCCACUGUGCGUGUGACAGCGGCUGAUCUGUGCCUGGGCAGCGCGACCC

>hsa-mir-211 MI0000287

UCACCUGGCCAUGUGACUUGUGGGCUUCCCUUUGUCAUCCUUCGCCUAGGGCUCUGAGCAGGGCAGGGACAGCAAAGGGGUGCUCAGUUGUCACUUCCCACAGCACGGAG

>hsa-mir-212 MI0000288

CGGGGCACCCCGCCCGGACAGCGCGCCGGCACCUUGGCUCUAGACUGCUUACUGCCCGGGCCGCCCUCAGUAACAGUCUCCAGUCACGGCCACCGACGCCUGGCCCCGCC

>hsa-mir-214 MI0000290

GGCCUGGCUGGACAGAGUUGUCAUGUGUCUGCCUGUCUACACUUGCUGUGCAGAACAUCCGCUCACCUGUACAGCAGGCACAGACAGGCAGUCACAUGACAACCCAGCCU

>hsa-mir-215 MI0000291

AUCAUUCAGAAAUGGUAUACAGGAAAAUGACCUAUGAAUUGACAGACAAUAUAGCUGAGUUUGUCUGUCAUUUCUUUAGGCCAAUAUUCUGUAUGACUGUGCUACUUCAA

>hsa-mir-216a MI0000292

GAUGGCUGUGAGUUGGCUUAAUCUCAGCUGGCAACUGUGAGAUGUUCAUACAAUCCCUCACAGUGGUCUCUGGGAUUAUGCUAAACAGAGCAAUUUCCUAGCCCUCACGA

>hsa-mir-216b MI0005569

GCAGACUGGAAAAUCUCUGCAGGCAAAUGUGAUGUCACUGAGGAAAUCACACACUUACCCGUAGAGAUUCUACAGUCUGACA

>hsa-mir-217 MI0000293

AGUAUAAUUAUUACAUAGUUUUUGAUGUCGCAGAUACUGCAUCAGGAACUGAUUGGAUAAGAAUCAGUCACCAUCAGUUCCUAAUGCAUUGCCUUCAGCAUCUAAACAAG

>hsa-mir-218-1 MI0000294

GUGAUAAUGUAGCGAGAUUUUCUGUUGUGCUUGAUCUAACCAUGUGGUUGCGAGGUAUGAGUAAAACAUGGUUCCGUCAAGCACCAUGGAACGUCACGCAGCUUUCUACA

>hsa-mir-218-2 MI0000295

GACCAGUCGCUGCGGGGCUUUCCUUUGUGCUUGAUCUAACCAUGUGGUGGAACGAUGGAAACGGAACAUGGUUCUGUCAAGCACCGCGGAAAGCACCGUGCUCUCCUGCA

>hsa-mir-219a-1 MI0000296

CCGCCCCGGGCCGCGGCUCCUGAUUGUCCAAACGCAAUUCUCGAGUCUAUGGCUCCGGCCGAGAGUUGAGUCUGGACGUCCCGAGCCGCCGCCCCCAAACCUCGAGCGGG

>hsa-mir-219a-2 MI0000740

ACUCAGGGGCUUCGCCACUGAUUGUCCAAACGCAAUUCUUGUACGAGUCUGCGGCCAACCGAGAAUUGUGGCUGGACAUCUGUGGCUGAGCUCCGGG

>hsa-mir-219b MI0017299

GGAGCUCAGCCACAGAUGUCCAGCCACAAUUCUCGGUUGGCCGCAGACUCGUACAAGAAUUGCGUUUGGACAAUCAGUGGCGAAGCCC

>hsa-mir-221 MI0000298

UGAACAUCCAGGUCUGGGGCAUGAACCUGGCAUACAAUGUAGAUUUCUGUGUUCGUUAGGCAACAGCUACAUUGUCUGCUGGGUUUCAGGCUACCUGGAAACAUGUUCUC

>hsa-mir-222 MI0000299

GCUGCUGGAAGGUGUAGGUACCCUCAAUGGCUCAGUAGCCAGUGUAGAUCCUGUCUUUCGUAAUCAGCAGCUACAUCUGGCUACUGGGUCUCUGAUGGCAUCUUCUAGCU

>hsa-mir-223 MI0000300

CCUGGCCUCCUGCAGUGCCACGCUCCGUGUAUUUGACAAGCUGAGUUGGACACUCCAUGUGGUAGAGUGUCAGUUUGUCAAAUACCCCAAGUGCGGCACAUGCUUACCAG

>hsa-mir-224 MI0000301

GGGCUUUCAAGUCACUAGUGGUUCCGUUUAGUAGAUGAUUGUGCAUUGUUUCAAAAUGGUGCCCUAGUGACUACAAAGCCC

>hsa-mir-296 MI0000747

AGGACCCUUCCAGAGGGCCCCCCCUCAAUCCUGUUGUGCCUAAUUCAGAGGGUUGGGUGGAGGCUCUCCUGAAGGGCUCU

>hsa-mir-297 MI0005775

UGUAUGUAUGUGUGCAUGUGCAUGUAUGUGUAUAUACAUAUAUAUGUAUUAUGUACUCAUAUAUCA

>hsa-mir-298 MI0005523

UCAGGUCUUCAGCAGAAGCAGGGAGGUUCUCCCAGUGGUUUUCCUUGACUGUGAGGAACUAGCCUGCUGCUUUGCUCAGGAGUGAGCU

>hsa-mir-299 MI0000744

AAGAAAUGGUUUACCGUCCCACAUACAUUUUGAAUAUGUAUGUGGGAUGGUAAACCGCUUCUU

>hsa-mir-300 MI0005525

UGCUACUUGAAGAGAGGUAAUCCUUCACGCAUUUGCUUUACUUGCAAUGAUUAUACAAGGGCAGACUCUCUCUGGGGAGCAAA

>hsa-mir-301a MI0000745

ACUGCUAACGAAUGCUCUGACUUUAUUGCACUACUGUACUUUACAGCUAGCAGUGCAAUAGUAUUGUCAAAGCAUCUGAAAGCAGG

>hsa-mir-301b MI0005568

GCCGCAGGUGCUCUGACGAGGUUGCACUACUGUGCUCUGAGAAGCAGUGCAAUGAUAUUGUCAAAGCAUCUGGGACCA

>hsa-mir-302a MI0000738

CCACCACUUAAACGUGGAUGUACUUGCUUUGAAACUAAAGAAGUAAGUGCUUCCAUGUUUUGGUGAUGG

>hsa-mir-302b MI0000772

GCUCCCUUCAACUUUAACAUGGAAGUGCUUUCUGUGACUUUAAAAGUAAGUGCUUCCAUGUUUUAGUAGGAGU

>hsa-mir-302c MI0000773

CCUUUGCUUUAACAUGGGGGUACCUGCUGUGUGAAACAAAAGUAAGUGCUUCCAUGUUUCAGUGGAGG

>hsa-mir-302e MI0006417

UUGGGUAAGUGCUUCCAUGCUUCAGUUUCCUUACUGGUAAGAUGGAUGUAGUAAUAGCACCUACCUUAUAGA

>hsa-mir-302f MI0006418

UCUGUGUAAACCUGGCAAUUUUCACUUAAUUGCUUCCAUGUUUAUAAAAGA

>hsa-mir-320a MI0000542

GCUUCGCUCCCCUCCGCCUUCUCUUCCCGGUUCUUCCCGGAGUCGGGAAAAGCUGGGUUGAGAGGGCGAAAAAGGAUGAGGU

>hsa-mir-320b-1 MI0003776

AAUUAAUCCCUCUCUUUCUAGUUCUUCCUAGAGUGAGGAAAAGCUGGGUUGAGAGGGCAAACAAAUUAACUAAUUAAUU

>hsa-mir-320b-2 MI0003839

UGUUAUUUUUUGUCUUCUACCUAAGAAUUCUGUCUCUUAGGCUUUCUCUUCCCAGAUUUCCCAAAGUUGGGAAAAGCUGGGUUGAGAGGGCAAAAGGAAAAAAAAAGAAUUCUGUCUCUGACAUAAUUAGAUAGGGAA

>hsa-mir-320c-1 MI0003778

UUUGCAUUAAAAAUGAGGCCUUCUCUUCCCAGUUCUUCCCAGAGUCAGGAAAAGCUGGGUUGAGAGGGUAGAAAAAAAAUGAUGUAGG

>hsa-mir-323a MI0000807

UUGGUACUUGGAGAGAGGUGGUCCGUGGCGCGUUCGCUUUAUUUAUGGCGCACAUUACACGGUCGACCUCUUUGCAGUAUCUAAUC

>hsa-mir-324 MI0000813

CUGACUAUGCCUCCCCGCAUCCCCUAGGGCAUUGGUGUAAAGCUGGAGACCCACUGCCCCAGGUGCUGCUGGGGGUUGUAGUC

>hsa-mir-325 MI0000824

AUACAGUGCUUGGUUCCUAGUAGGUGUCCAGUAAGUGUUUGUGACAUAAUUUGUUUAUUGAGGACCUCCUAUCAAUCAAGCACUGUGCUAGGCUCUGG

>hsa-mir-326 MI0000808

CUCAUCUGUCUGUUGGGCUGGAGGCAGGGCCUUUGUGAAGGCGGGUGGUGCUCAGAUCGCCUCUGGGCCCUUCCUCCAGCCCCGAGGCGGAUUCA

>hsa-mir-328 MI0000804

UGGAGUGGGGGGGCAGGAGGGGCUCAGGGAGAAAGUGCAUACAGCCCCUGGCCCUCUCUGCCCUUCCGUCCCCUG

>hsa-mir-329-2 MI0001726

GUGGUACCUGAAGAGAGGUUUUCUGGGUUUCUGUUUCUUUAUUGAGGACGAAACACACCUGGUUAACCUCUUUUCCAGUAUCAA

>hsa-mir-330 MI0000803

CUUUGGCGAUCACUGCCUCUCUGGGCCUGUGUCUUAGGCUCUGCAAGAUCAACCGAGCAAAGCACACGGCCUGCAGAGAGGCAGCGCUCUGCCC

>hsa-mir-331 MI0000812

GAGUUUGGUUUUGUUUGGGUUUGUUCUAGGUAUGGUCCCAGGGAUCCCAGAUCAAACCAGGCCCCUGGGCCUAUCCUAGAACCAACCUAAGCUC

>hsa-mir-335 MI0000816

UGUUUUGAGCGGGGGUCAAGAGCAAUAACGAAAAAUGUUUGUCAUAAACCGUUUUUCAUUAUUGCUCCUGACCUCCUCUCAUUUGCUAUAUUCA

>hsa-mir-337 MI0000806

GUAGUCAGUAGUUGGGGGGUGGGAACGGCUUCAUACAGGAGUUGAUGCACAGUUAUCCAGCUCCUAUAUGAUGCCUUUCUUCAUCCCCUUCAA

>hsa-mir-338 MI0000814

UCUCCAACAAUAUCCUGGUGCUGAGUGAUGACUCAGGCGACUCCAGCAUCAGUGAUUUUGUUGAAGA

>hsa-mir-339 MI0000815

CGGGGCGGCCGCUCUCCCUGUCCUCCAGGAGCUCACGUGUGCCUGCCUGUGAGCGCCUCGACGACAGAGCCGGCGCCUGCCCCAGUGUCUGCGC

>hsa-mir-340 MI0000802

UUGUACCUGGUGUGAUUAUAAAGCAAUGAGACUGAUUGUCAUAUGUCGUUUGUGGGAUCCGUCUCAGUUACUUUAUAGCCAUACCUGGUAUCUUA

>hsa-mir-342 MI0000805

GAAACUGGGCUCAAGGUGAGGGGUGCUAUCUGUGAUUGAGGGACAUGGUUAAUGGAAUUGUCUCACACAGAAAUCGCACCCGUCACCUUGGCCUACUUA

>hsa-mir-345 MI0000825

ACCCAAACCCUAGGUCUGCUGACUCCUAGUCCAGGGCUCGUGAUGGCUGGUGGGCCCUGAACGAGGGGUCUGGAGGCCUGGGUUUGAAUAUCGACAGC

>hsa-mir-346 MI0000826

GGUCUCUGUGUUGGGCGUCUGUCUGCCCGCAUGCCUGCCUCUCUGUUGCUCUGAAGGAGGCAGGGGCUGGGCCUGCAGCUGCCUGGGCAGAGCGG

>hsa-mir-361 MI0000760

GGAGCUUAUCAGAAUCUCCAGGGGUACUUUAUAAUUUCAAAAAGUCCCCCAGGUGUGAUUCUGAUUUGCUUC

>hsa-mir-362 MI0000762

CUUGAAUCCUUGGAACCUAGGUGUGAGUGCUAUUUCAGUGCAACACACCUAUUCAAGGAUUCAAA

>hsa-mir-363 MI0000764

UGUUGUCGGGUGGAUCACGAUGCAAUUUUGAUGAGUAUCAUAGGAGAAAAAUUGCACGGUAUCCAUCUGUAAACC

>hsa-mir-365b MI0000769

AGAGUGUUCAAGGACAGCAAGAAAAAUGAGGGACUUUCAGGGGCAGCUGUGUUUUCUGACUCAGUCAUAAUGCCCCUAAAAAUCCUUAUUGUUCUUGCAGUGUGCAUCGGG

>hsa-mir-367 MI0000775

CCAUUACUGUUGCUAAUAUGCAACUCUGUUGAAUAUAAAUUGGAAUUGCACUUUAGCAAUGGUGAUGG

>hsa-mir-369 MI0000777

UUGAAGGGAGAUCGACCGUGUUAUAUUCGCUUUAUUGACUUCGAAUAAUACAUGGUUGAUCUUUUCUCAG

>hsa-mir-370 MI0000778

AGACAGAGAAGCCAGGUCACGUCUCUGCAGUUACACAGCUCACGAGUGCCUGCUGGGGUGGAACCUGGUCUGUCU

>hsa-mir-371a MI0000779

GUGGCACUCAAACUGUGGGGGCACUUUCUGCUCUCUGGUGAAAGUGCCGCCAUCUUUUGAGUGUUAC

>hsa-mir-371b MI0017393

GGUAACACUCAAAAGAUGGCGGCACUUUCACCAGAGAGCAGAAAGUGCCCCCACAGUUUGAGUGCC

>hsa-mir-372 MI0000780

GUGGGCCUCAAAUGUGGAGCACUAUUCUGAUGUCCAAGUGGAAAGUGCUGCGACAUUUGAGCGUCAC

>hsa-mir-373 MI0000781

GGGAUACUCAAAAUGGGGGCGCUUUCCUUUUUGUCUGUACUGGGAAGUGCUUCGAUUUUGGGGUGUCCC

>hsa-mir-374a MI0000782

UACAUCGGCCAUUAUAAUACAACCUGAUAAGUGUUAUAGCACUUAUCAGAUUGUAUUGUAAUUGUCUGUGUA

>hsa-mir-374b MI0005566

ACUCGGAUGGAUAUAAUACAACCUGCUAAGUGUCCUAGCACUUAGCAGGUUGUAUUAUCAUUGUCCGUGUCU

>hsa-mir-374c MI0016684

ACACGGACAAUGAUAAUACAACCUGCUAAGUGCUAGGACACUUAGCAGGUUGUAUUAUAUCCAUCCGAGU

>hsa-mir-375 MI0000783

CCCCGCGACGAGCCCCUCGCACAAACCGGACCUGAGCGUUUUGUUCGUUCGGCUCGCGUGAGGC

>hsa-mir-376b MI0002466

CAGUCCUUCUUUGGUAUUUAAAACGUGGAUAUUCCUUCUAUGUUUACGUGAUUCCUGGUUAAUCAUAGAGGAAAAUCCAUGUUUUCAGUAUCAAAUGCUG

>hsa-mir-377 MI0000785

UUGAGCAGAGGUUGCCCUUGGUGAAUUCGCUUUAUUUAUGUUGAAUCACACAAAGGCAACUUUUGUUUG

>hsa-mir-378a MI0000786

AGGGCUCCUGACUCCAGGUCCUGUGUGUUACCUAGAAAUAGCACUGGACUUGGAGUCAGAAGGCCU

>hsa-mir-378b MI0014154

GGUCAUUGAGUCUUCAAGGCUAGUGGAAAGAGCACUGGACUUGGAGGCAGAAAGACC

>hsa-mir-378c MI0015825

GGAGGCCAUCACUGGACUUGGAGUCAGAAGAGUGGAGUCGGGUCAGACUUCAACUCUGACUUUGAAGGUGGUGAGUGCCUC

>hsa-mir-378d-1 MI0016749

ACUGUUUCUGUCCUUGUUCUUGUUGUUAUUACUGGACUUGGAGUCAGAAACAGG

>hsa-mir-378d-2 MI0003840

GAAUGGUUACAAGGAGAGAACACUGGACUUGGAGUCAGAAAACUUUCAUCCAAGUCAUUCCCUGCUCUAAGUCCCAUUUCUGUUCCAUGAGAUUGUUU

>hsa-mir-378e MI0016750

CUGACUCCAGUGUCCAGGCCAGGGGCAGACAGUGGACAGAGAACAGUGCCCAAGACCACUGGACUUGGAGUCAGGACAU

>hsa-mir-378f MI0016756

GUCAGGUCCUGGACUCCCAUAGUUUUCAGGCUGCUAAACAACAGAACGAGCACUGGACUUGGAGCCAGAAGUCUUGGG

>hsa-mir-378h MI0016808

ACAGGAACACUGGACUUGGUGUCAGAUGGGAUGAGCCCUGGCUCUGUUUCCUAGCAGCAAUCUGAUCUUGAGCUAGUCACUGG

>hsa-mir-378i MI0016902

GGGAGCACUGGACUAGGAGUCAGAAGGUGGAGUUCUGGGUGCUGUUUUCCCACUCUUGGGCCCUGGGCAUGUUCUG

>hsa-mir-378j MI0021273

AUGCAGUGAGUCGGGGAGGAACUGGAUUUGGAGCCAGAAGAACUGGUUCUAAUAUCUACUUCCCUGUGUAGAGUUGGGAUUUGGGAUUAUAUGAGUUAAUAUACACCAA

>hsa-mir-379 MI0000787

AGAGAUGGUAGACUAUGGAACGUAGGCGUUAUGAUUUCUGACCUAUGUAACAUGGUCCACUAACUCU

>hsa-mir-380 MI0000788

AAGAUGGUUGACCAUAGAACAUGCGCUAUCUCUGUGUCGUAUGUAAUAUGGUCCACAUCUU

>hsa-mir-381 MI0000789

UACUUAAAGCGAGGUUGCCCUUUGUAUAUUCGGUUUAUUGACAUGGAAUAUACAAGGGCAAGCUCUCUGUGAGUA

>hsa-mir-382 MI0000790

UACUUGAAGAGAAGUUGUUCGUGGUGGAUUCGCUUUACUUAUGACGAAUCAUUCACGGACAACACUUUUUUCAGUA

>hsa-mir-383 MI0000791

CUCCUCAGAUCAGAAGGUGAUUGUGGCUUUGGGUGGAUAUUAAUCAGCCACAGCACUGCCUGGUCAGAAAGAG

>hsa-mir-384 MI0001145

UGUUAAAUCAGGAAUUUUAAACAAUUCCUAGACAAUAUGUAUAAUGUUCAUAAGUCAUUCCUAGAAAUUGUUCAUAAUGCCUGUAACA

>hsa-mir-409 MI0001735

UGGUACUCGGGGAGAGGUUACCCGAGCAACUUUGCAUCUGGACGACGAAUGUUGCUCGGUGAACCCCUUUUCGGUAUCA

>hsa-mir-410 MI0002465

GGUACCUGAGAAGAGGUUGUCUGUGAUGAGUUCGCUUUUAUUAAUGACGAAUAUAACACAGAUGGCCUGUUUUCAGUACC

>hsa-mir-411 MI0003675

UGGUACUUGGAGAGAUAGUAGACCGUAUAGCGUACGCUUUAUCUGUGACGUAUGUAACACGGUCCACUAACCCUCAGUAUCAAAUCCAUCCCCGAG

>hsa-mir-412 MI0002464

CUGGGGUACGGGGAUGGAUGGUCGACCAGUUGGAAAGUAAUUGUUUCUAAUGUACUUCACCUGGUCCACUAGCCGUCCGUAUCCGCUGCAG

>hsa-mir-422a MI0001444

GAGAGAAGCACUGGACUUAGGGUCAGAAGGCCUGAGUCUCUCUGCUGCAGAUGGGCUCUCUGUCCCUGAGCCAAGCUUUGUCCUCCCUGG

>hsa-mir-423 MI0001445

AUAAAGGAAGUUAGGCUGAGGGGCAGAGAGCGAGACUUUUCUAUUUUCCAAAAGCUCGGUCUGAGGCCCCUCAGUCUUGCUUCCUAACCCGCGC

>hsa-mir-424 MI0001446

CGAGGGGAUACAGCAGCAAUUCAUGUUUUGAAGUGUUCUAAAUGGUUCAAAACGUGAGGCGCUGCUAUACCCCCUCGUGGGGAAGGUAGAAGGUGGGG

>hsa-mir-425 MI0001448

GAAAGCGCUUUGGAAUGACACGAUCACUCCCGUUGAGUGGGCACCCGAGAAGCCAUCGGGAAUGUCGUGUCCGCCCAGUGCUCUUUC

>hsa-mir-429 MI0001641

CGCCGGCCGAUGGGCGUCUUACCAGACAUGGUUAGACCUGGCCCUCUGUCUAAUACUGUCUGGUAAAACCGUCCAUCCGCUGC

>hsa-mir-431 MI0001721

UCCUGCUUGUCCUGCGAGGUGUCUUGCAGGCCGUCAUGCAGGCCACACUGACGGUAACGUUGCAGGUCGUCUUGCAGGGCUUCUCGCAAGACGACAUCCUCAUCACCAACGACG

>hsa-mir-432 MI0003133

UGACUCCUCCAGGUCUUGGAGUAGGUCAUUGGGUGGAUCCUCUAUUUCCUUACGUGGGCCACUGGAUGGCUCCUCCAUGUCUUGGAGUAGAUCA

>hsa-mir-433 MI0001723

CCGGGGAGAAGUACGGUGAGCCUGUCAUUAUUCAGAGAGGCUAGAUCCUCUGUGUUGAGAAGGAUCAUGAUGGGCUCCUCGGUGUUCUCCAGG

>hsa-mir-448 MI0001637

GCCGGGAGGUUGAACAUCCUGCAUAGUGCUGCCAGGAAAUCCCUAUUUCAUAUAAGAGGGGGCUGGCUGGUUGCAUAUGUAGGAUGUCCCAUCUCCCAGCCCACUUCGUCA

>hsa-mir-449a MI0001648

CUGUGUGUGAUGAGCUGGCAGUGUAUUGUUAGCUGGUUGAAUAUGUGAAUGGCAUCGGCUAACAUGCAACUGCUGUCUUAUUGCAUAUACA

>hsa-mir-449b MI0003673

UGACCUGAAUCAGGUAGGCAGUGUAUUGUUAGCUGGCUGCUUGGGUCAAGUCAGCAGCCACAACUACCCUGCCACUUGCUUCUGGAUAAAUUCUUCU

>hsa-mir-449c MI0003823

GCUGGGAUGUGUCAGGUAGGCAGUGUAUUGCUAGCGGCUGUUAAUGAUUUUAACAGUUGCUAGUUGCACUCCUCUCUGUUGCAUUCAGAAGC

>hsa-mir-450a-2 MI0003187

CCAAAGAAAGAUGCUAAACUAUUUUUGCGAUGUGUUCCUAAUAUGUAAUAUAAAUGUAUUGGGGACAUUUUGCAUUCAUAGUUUUGUAUCAAUAAUAUGG

>hsa-mir-451a MI0001729

CUUGGGAAUGGCAAGGAAACCGUUACCAUUACUGAGUUUAGUAAUGGUAAUGGUUCUCUUGCUAUACCCAGA

>hsa-mir-451b MI0017360

UGGGUAUAGCAAGAGAACCAUUACCAUUACUAAACUCAGUAAUGGUAACGGUUUCCUUGCCAUUCCCA

>hsa-mir-452 MI0001733

GCUAAGCACUUACAACUGUUUGCAGAGGAAACUGAGACUUUGUAACUAUGUCUCAGUCUCAUCUGCAAAGAAGUAAGUGCUUUGC

>hsa-mir-454 MI0003820

UCUGUUUAUCACCAGAUCCUAGAACCCUAUCAAUAUUGUCUCUGCUGUGUAAAUAGUUCUGAGUAGUGCAAUAUUGCUUAUAGGGUUUUGGUGUUUGGAAAGAACAAUGGGCAGG

>hsa-mir-455 MI0003513

UCCCUGGCGUGAGGGUAUGUGCCUUUGGACUACAUCGUGGAAGCCAGCACCAUGCAGUCCAUGGGCAUAUACACUUGCCUCAAGGCCUAUGUCAUC

>hsa-mir-466 MI0014157

GUGUGUGUAUAUGUGUGUUGCAUGUGUGUAUAUGUGUGUAUAUAUGUACACAUACACAUACACGCAACACACAUAUAUACAUGC

>hsa-mir-483 MI0002467

GAGGGGGAAGACGGGAGGAAAGAAGGGAGUGGUUCCAUCACGCCUCCUCACUCCUCUCCUCCCGUCUUCUCCUCUC

>hsa-mir-484 MI0002468

AGCCUCGUCAGGCUCAGUCCCCUCCCGAUAAACCCCUAAAUAGGGACUUUCCCGGGGGGUGACCCUGGCUUUUUUGGCG

>hsa-mir-485 MI0002469

ACUUGGAGAGAGGCUGGCCGUGAUGAAUUCGAUUCAUCAAAGCGAGUCAUACACGGCUCUCCUCUCUUUUAGU

>hsa-mir-486 MI0002470

GCAUCCUGUACUGAGCUGCCCCGAGGCCCUUCAUGCUGCCCAGCUCGGGGCAGCUCAGUACAGGAUAC

>hsa-mir-487b MI0003530

UUGGUACUUGGAGAGUGGUUAUCCCUGUCCUGUUCGUUUUGCUCAUGUCGAAUCGUACAGGGUCAUCCACUUUUUCAGUAUCAA

>hsa-mir-488 MI0003123

GAGAAUCAUCUCUCCCAGAUAAUGGCACUCUCAAACAAGUUUCCAAAUUGUUUGAAAGGCUAUUUCUUGGUCAGAUGACUCUC

>hsa-mir-489 MI0003124

GUGGCAGCUUGGUGGUCGUAUGUGUGACGCCAUUUACUUGAACCUUUAGGAGUGACAUCACAUAUACGGCAGCUAAACUGCUAC

>hsa-mir-490 MI0003125

UGGAGGCCUUGCUGGUUUGGAAAGUUCAUUGUUCGACACCAUGGAUCUCCAGGUGGGUCAAGUUUAGAGAUGCACCAACCUGGAGGACUCCAUGCUGUUGAGCUGUUCACAAGCAGCGGACACUUCCA

>hsa-mir-491 MI0003126

UUGACUUAGCUGGGUAGUGGGGAACCCUUCCAUGAGGAGUAGAACACUCCUUAUGCAAGAUUCCCUUCUACCUGGCUGGGUUGG

>hsa-mir-492 MI0003131

CAACUACAGCCACUACUACAGGACCAUCGAGGACCUGCGGGACAAGAUUCUUGGUGCCACCAUUGAGAACGCCAGGAUUGUCCUGCAGAUCAACAAUGCUCAACUGGCUGCAGAUG

>hsa-mir-493 MI0003132

CUGGCCUCCAGGGCUUUGUACAUGGUAGGCUUUCAUUCAUUCGUUUGCACAUUCGGUGAAGGUCUACUGUGUGCCAGGCCCUGUGCCAG

>hsa-mir-494 MI0003134

GAUACUCGAAGGAGAGGUUGUCCGUGUUGUCUUCUCUUUAUUUAUGAUGAAACAUACACGGGAAACCUCUUUUUUAGUAUC

>hsa-mir-495 MI0003135

UGGUACCUGAAAAGAAGUUGCCCAUGUUAUUUUCGCUUUAUAUGUGACGAAACAAACAUGGUGCACUUCUUUUUCGGUAUCA

>hsa-mir-496 MI0003136

CCCAAGUCAGGUACUCGAAUGGAGGUUGUCCAUGGUGUGUUCAUUUUAUUUAUGAUGAGUAUUACAUGGCCAAUCUCCUUUCGGUACUCAAUUCUUCUUGGG

>hsa-mir-497 MI0003138

CCACCCCGGUCCUGCUCCCGCCCCAGCAGCACACUGUGGUUUGUACGGCACUGUGGCCACGUCCAAACCACACUGUGGUGUUAGAGCGAGGGUGGGGGAGGCACCGCCGAGG

>hsa-mir-498 MI0003142

AACCCUCCUUGGGAAGUGAAGCUCAGGCUGUGAUUUCAAGCCAGGGGGCGUUUUUCUAUAACUGGAUGAAAAGCACCUCCAGAGCUUGAAGCUCACAGUUUGAGAGCAAUCGUCUAAGGAAGUU

>hsa-mir-499a MI0003183

GCCCUGUCCCCUGUGCCUUGGGCGGGCGGCUGUUAAGACUUGCAGUGAUGUUUAACUCCUCUCCACGUGAACAUCACAGCAAGUCUGUGCUGCUUCCCGUCCCUACGCUGCCUGGGCAGGGU

>hsa-mir-499b MI0017396

GGAAGCAGCACAGACUUGCUGUGAUGUUCACGUGGAGAGGAGUUAAACAUCACUGCAAGUCUUAACAGCCGCC

>hsa-mir-502 MI0003186

UGCUCCCCCUCUCUAAUCCUUGCUAUCUGGGUGCUAGUGCUGGCUCAAUGCAAUGCACCUGGGCAAGGAUUCAGAGAGGGGGAGCU

>hsa-mir-503 MI0003188

UGCCCUAGCAGCGGGAACAGUUCUGCAGUGAGCGAUCGGUGCUCUGGGGUAUUGUUUCCGCUGCCAGGGUA

>hsa-mir-504 MI0003189

GCUGCUGUUGGGAGACCCUGGUCUGCACUCUAUCUGUAUUCUUACUGAAGGGAGUGCAGGGCAGGGUUUCCCAUACAGAGGGC

>hsa-mir-505 MI0003190

GAUGCACCCAGUGGGGGAGCCAGGAAGUAUUGAUGUUUCUGCCAGUUUAGCGUCAACACUUGCUGGUUUCCUCUCUGGAGCAUC

>hsa-mir-506 MI0003193

GCCACCACCAUCAGCCAUACUAUGUGUAGUGCCUUAUUCAGGAAGGUGUUACUUAAUAGAUUAAUAUUUGUAAGGCACCCUUCUGAGUAGAGUAAUGUGCAACAUGGACAACAUUUGUGGUGGC

>hsa-mir-507 MI0003194

GUGCUGUGUGUAGUGCUUCACUUCAAGAAGUGCCAUGCAUGUGUCUAGAAAUAUGUUUUGCACCUUUUGGAGUGAAAUAAUGCACAACAGAUAC

>hsa-mir-508 MI0003195

CCACCUUCAGCUGAGUGUAGUGCCCUACUCCAGAGGGCGUCACUCAUGUAAACUAAAACAUGAUUGUAGCCUUUUGGAGUAGAGUAAUACACAUCACGUAACGCAUAUUUGGUGG

>hsa-mir-510 MI0003197

GUGGUGUCCUACUCAGGAGAGUGGCAAUCACAUGUAAUUAGGUGUGAUUGAAACCUCUAAGAGUGGAGUAACAC

>hsa-mir-511 MI0003127

CAAUAGACACCCAUCGUGUCUUUUGCUCUGCAGUCAGUAAAUAUUUUUUUGUGAAUGUGUAGCAAAAGACAGAAUGGUGGUCCAUUG

>hsa-mir-512-2 MI0003141

GGUACUUCUCAGUCUGUGGCACUCAGCCUUGAGGGCACUUUCUGGUGCCAGAAUGAAAGUGCUGUCAUAGCUGAGGUCCAAUGACUGAGGCGAGCACC

>hsa-mir-513a-1 MI0003191

GGGAUGCCACAUUCAGCCAUUCAGCGUACAGUGCCUUUCACAGGGAGGUGUCAUUUAUGUGAACUAAAAUAUAAAUUUCACCUUUCUGAGAAGGGUAAUGUACAGCAUGCACUGCAUAUGUGGUGUCCC

>hsa-mir-514a-1 MI0003198

AACAUGUUGUCUGUGGUACCCUACUCUGGAGAGUGACAAUCAUGUAUAAUUAAAUUUGAUUGACACUUCUGUGAGUAGAGUAACGCAUGACACGUACG

>hsa-mir-518c MI0003159

GCGAGAAGAUCUCAUGCUGUGACUCUCUGGAGGGAAGCACUUUCUGUUGUCUGAAAGAAAACAAAGCGCUUCUCUUUAGAGUGUUACGGUUUGAGAAAAGC

>hsa-mir-519d MI0003162

UCCCAUGCUGUGACCCUCCAAAGGGAAGCGCUUUCUGUUUGUUUUCUCUUAAACAAAGUGCCUCCCUUUAGAGUGUUACCGUUUGGGA

>hsa-mir-532 MI0003205

CGACUUGCUUUCUCUCCUCCAUGCCUUGAGUGUAGGACCGUUGGCAUCUUAAUUACCCUCCCACACCCAAGGCUUGCAAAAAAGCGAGCCU

>hsa-mir-541 MI0005539

ACGUCAGGGAAAGGAUUCUGCUGUCGGUCCCACUCCAAAGUUCACAGAAUGGGUGGUGGGCACAGAAUCUGGACUCUGCUUGUG

>hsa-mir-542 MI0003686

CAGAUCUCAGACAUCUCGGGGAUCAUCAUGUCACGAGAUACCAGUGUGCACUUGUGACAGAUUGAUAACUGAAAGGUCUGGGAGCCACUCAUCUUCA

>hsa-mir-544a MI0003515

AUUUUCAUCACCUAGGGAUCUUGUUAAAAAGCAGAUUCUGAUUCAGGGACCAAGAUUCUGCAUUUUUAGCAAGUUCUCAAGUGAUGCUAAU

>hsa-mir-544b MI0014159

GGAAUUUUGUUAAAAUGCAGAAUCCAUUUCUGUAGCUCUGAGACUAGACCUGAGGUUGUGCAUUUCUAACAAAGUGCC

>hsa-mir-545 MI0003516

CCCAGCCUGGCACAUUAGUAGGCCUCAGUAAAUGUUUAUUAGAUGAAUAAAUGAAUGACUCAUCAGCAAACAUUUAUUGUGUGCCUGCUAAAGUGAGCUCCACAGG

>hsa-mir-548a-1 MI0003593

UGCAGGGAGGUAUUAAGUUGGUGCAAAAGUAAUUGUGAUUUUUGCCAUUAAAAGUAACGACAAAACUGGCAAUUACUUUUGCACCAAACCUGGUAUU

>hsa-mir-548a-3 MI0003612

CCUAGAAUGUUAUUAGGUCGGUGCAAAAGUAAUUGCGAGUUUUACCAUUACUUUCAAUGGCAAAACUGGCAAUUACUUUUGCACCAACGUAAUACUU

>hsa-mir-548al MI0016851

GGUCGGUGCAAAAGUAAUUGCUGUUUUUGCCAUUAAAAAUAAUGGCAUUAAAAGUAAUGGCAAAAACGGCAAUGACUUUUGUACCAAUCUAAUAUCU

>hsa-mir-548ao MI0017871

AACUAUUCUUAGGUUGAUGCAGAAGUAACUACGGUUUUUGCAGUUGAAAGUAAUGGCAAAGACCGUGACUACUUUUGCAACAGCCUAAUAGUUUCU

>hsa-mir-548b MI0003596

CAGACUAUAUAUUUAGGUUGGCGCAAAAGUAAUUGUGGUUUUGGCCUUUAUUUUCAAUGGCAAGAACCUCAGUUGCUUUUGUGCCAACCUAAUACUU

>hsa-mir-548d-1 MI0003668

AAACAAGUUAUAUUAGGUUGGUGCAAAAGUAAUUGUGGUUUUUGCCUGUAAAAGUAAUGGCAAAAACCACAGUUUCUUUUGCACCAGACUAAUAAAG

>hsa-mir-548f-2 MI0006375

UAAUAACUAUUAGGUUGGUGCGAACAUAAUUGCAGUUUUUAUCAUUACUUUUAAUGGCAAAAACUGUAAUUACUUUUGCACCAACCUAAUAUUUUAGU

>hsa-mir-548h-3 MI0006413

UCUGAUUCUGCAUGUAUUAGGUUGGUGCAAAAGUAAUCGCGGUUUUUGUCAUUGAAAGUAAUAGCAAAAACUGCAAUUACUUUUGCACCAACCUAAAAGUAGUCACUGUCUUCAGAUA

>hsa-mir-548h-4 MI0006414

GCUAUUAGGUUGGUGCAAAAGUAAUCGCGGUUUUUGUCAUUACUUUAAUUACUUUACGUUUCAUUAAUGACAAAAACCGCAAUUACUUUUGCACCAACCUAAUACUUGCUA

>hsa-mir-548i-1 MI0006421

CAGAUGGCUCUGAAGUUUGCACCCUAUUAGGUUGGUGCAAAAGUAAUUGCGGAUUUUGCCAUUAAAAGUAAUGGCAAAAAUAGCAAUUAUUUUUGUACCAGCCUAGUAUCUUUUCUCCUUCUACCAAACUUUGUCCCUGAGCCAUCUCA

>hsa-mir-548j MI0006345

GGGCAGCCAGUGAAUAGUUAGCUGGUGCAAAAGUAAUUGCGGUCUUUGGUAUUACUUUCAGUGGCAAAAACUGCAUUACUUUUGCACCAGCCUACUAGAACGCUGAGUUCAG

>hsa-mir-548k MI0006354

CUUUUCUCAAGUAUUGCUGUUAGGUUGGUGCAAAAGUACUUGCGGAUUUUGCUUUACUUUUAAUGGCAAAAACCGCAAUUAUUUUUGCUUCAACCUAAUAUGAUGCAAAAUUGGCU

>hsa-mir-548o MI0006402

UGGUGAAAAUGUGUUGAUUGUAAUGGUUCCUAUUCUGAUCAAUAAACAUGGUUUGAGCCUAGUUACAAUGAUCUAAAAUUCACGGUCCAAAACUGCAGUUACUUUUGCACCAAC

>hsa-mir-548q MI0010637

AUAUUAGGCUGGUGCAAAAGUAAUGGCGGUUUUUGCCAUUACUUUUCAUUUUUACCAUUAAAAGUAAUGGCAAAAAGCAUGAUUACUUUUUCACCAACCU

>hsa-mir-548y MI0016595

GCCUAAACUAUUAGGUUGGUGCAAAAGUAAUCACUGUUUUUGCCAUUACUCUCAGUGGCAAAAACCGUGAUUACUUUUGCACCAACCUAGUAACACCUUCACUGUGGGGG

>hsa-mir-549a MI0003679

AGACAUGCAACUCAAGAAUAUAUUGAGAGCUCAUCCAUAGUUGUCACUGUCUCAAAUCAGUGACAACUAUGGAUGAGCUCUUAAUAUAUCCCAGGC

>hsa-mir-550a-1 MI0003600

UGAUGCUUUGCUGGCUGGUGCAGUGCCUGAGGGAGUAAGAGCCCUGUUGUUGUAAGAUAGUGUCUUACUCCCUCAGGCACAUCUCCAACAAGUCUCU

>hsa-mir-550b-1 MI0016686

AGAGACUUGUUGGAGAUGUGCCUGAGGGAGUAAGACACUAUCUUACAACAACAGGGCUCUUACUCCCUCAGGCACUGCACCAGCCAGCAAAGCAUCA

>hsa-mir-551a MI0003556

GGGGACUGCCGGGUGACCCUGGAAAUCCAGAGUGGGUGGGGCCAGUCUGACCGUUUCUAGGCGACCCACUCUUGGUUUCCAGGGUUGCCCUGGAAA

>hsa-mir-551b MI0003575

AGAUGUGCUCUCCUGGCCCAUGAAAUCAAGCGUGGGUGAGACCUGGUGCAGAACGGGAAGGCGACCCAUACUUGGUUUCAGAGGCUGUGAGAAUAA

>hsa-mir-552 MI0003557

AACCAUUCAAAUAUACCACAGUUUGUUUAACCUUUUGCCUGUUGGUUGAAGAUGCCUUUCAACAGGUGACUGGUUAGACAAACUGUGGUAUAUACA

>hsa-mir-553 MI0003558

CUUCAAUUUUAUUUUAAAACGGUGAGAUUUUGUUUUGUCUGAGAAAAUCUCGCUGUUUUAGACUGAGG

>hsa-mir-554 MI0003559

ACCUGAGUAACCUUUGCUAGUCCUGACUCAGCCAGUACUGGUCUUAGACUGGUGAUGGGUCAGGGUUCAUAUUUUGGCAUCUCUCUCUGGGCAUCU

>hsa-mir-555 MI0003561

GGAGUGAACUCAGAUGUGGAGCACUACCUUUGUGAGCAGUGUGACCCAAGGCCUGUGGACAGGGUAAGCUGAACCUCUGAUAAAACUCUGAUCUAU

>hsa-mir-556 MI0003562

GAUAGUAAUAAGAAAGAUGAGCUCAUUGUAAUAUGAGCUUCAUUUAUACAUUUCAUAUUACCAUUAGCUCAUCUUUUUUAUUACUACCUUCAACA

>hsa-mir-557 MI0003563

AGAAUGGGCAAAUGAACAGUAAAUUUGGAGGCCUGGGGCCCUCCCUGCUGCUGGAGAAGUGUUUGCACGGGUGGGCCUUGUCUUUGAAAGGAGGUGGA

>hsa-mir-558 MI0003564

GUGUGUGUGUGUGUGUGUGGUUAUUUUGGUAUAGUAGCUCUAGACUCUAUUAUAGUUUCCUGAGCUGCUGUACCAAAAUACCACAAACGGGCUG

>hsa-mir-559 MI0003565

GCUCCAGUAACAUCUUAAAGUAAAUAUGCACCAAAAUUACUUUUGGUAAAUACAGUUUUGGUGCAUAUUUACUUUAGGAUGUUACUGGAGCUCCCA

>hsa-mir-561 MI0003567

CUUCAUCCACCAGUCCUCCAGGAACAUCAAGGAUCUUAAACUUUGCCAGAGCUACAAAGGCAAAGUUUAAGAUCCUUGAAGUUCCUGGGGGAACCAU

>hsa-mir-562 MI0003568

AGUGAAAUUGCUAGGUCAUAUGGUCAGUCUACUUUUAGAGUAAUUGUGAAACUGUUUUUCAAAGUAGCUGUACCAUUUGCACUCCCUGUGGCAAU

>hsa-mir-563 MI0003569

AGCAAAGAAGUGUGUUGCCCUCUAGGAAAUGUGUGUUGCUCUGAUGUAAUUAGGUUGACAUACGUUUCCCUGGUAGCCA

>hsa-mir-564 MI0003570

CGGGCAGCGGGUGCCAGGCACGGUGUCAGCAGGCAACAUGGCCGAGAGGCCGGGGCCUCCGGGCGGCGCCGUGUCCGCGACCGCGUACCCUGAC

>hsa-mir-566 MI0003572

GCUAGGCGUGGUGGCGGGCGCCUGUGAUCCCAACUACUCAGGAGGCUGGGGCAGCAGAAUCGCUUGAACCCGGGAGGCGAAGGUUGCAGUGAGC

>hsa-mir-567 MI0003573

GGAUUCUUAUAGGACAGUAUGUUCUUCCAGGACAGAACAUUCUUUGCUAUUUUGUACUGGAAGAACAUGCAAAACUAAAAAAAAAAAAAGUUAUUGCU

>hsa-mir-568 MI0003574

GAUAUACACUAUAUUAUGUAUAAAUGUAUACACACUUCCUAUAUGUAUCCACAUAUAUAUAGUGUAUAUAUUAUACAUGUAUAGGUGUGUAUAUG

>hsa-mir-569 MI0003576

GGUAUUGUUAGAUUAAUUUUGUGGGACAUUAACAACAGCAUCAGAAGCAACAUCAGCUUUAGUUAAUGAAUCCUGGAAAGUUAAGUGACUUUAUUU

>hsa-mir-571 MI0003578

CCUCAGUAAGACCAAGCUCAGUGUGCCAUUUCCUUGUCUGUAGCCAUGUCUAUGGGCUCUUGAGUUGGCCAUCUGAGUGAGGGCCUGCUUAUUCUA

>hsa-mir-572 MI0003579

GUCGAGGCCGUGGCCCGGAAGUGGUCGGGGCCGCUGCGGGCGGAAGGGCGCCUGUGCUUCGUCCGCUCGGCGGUGGCCCAGCCAGGCCCGCGGGA

>hsa-mir-573 MI0003580

UUUAGCGGUUUCUCCCUGAAGUGAUGUGUAACUGAUCAGGAUCUACUCAUGUCGUCUUUGGUAAAGUUAUGUCGCUUGUCAGGGUGAGGAGAGUUUUUG

>hsa-mir-574 MI0003581

GGGACCUGCGUGGGUGCGGGCGUGUGAGUGUGUGUGUGUGAGUGUGUGUCGCUCCGGGUCCACGCUCAUGCACACACCCACACGCCCACACUCAGG

>hsa-mir-575 MI0003582

AAUUCAGCCCUGCCACUGGCUUAUGUCAUGACCUUGGGCUACUCAGGCUGUCUGCACAAUGAGCCAGUUGGACAGGAGCAGUGCCACUCAACUC

>hsa-mir-576 MI0003583

UACAAUCCAACGAGGAUUCUAAUUUCUCCACGUCUUUGGUAAUAAGGUUUGGCAAAGAUGUGGAAAAAUUGGAAUCCUCAUUCGAUUGGUUAUAACCA

>hsa-mir-577 MI0003584

UGGGGGAGUGAAGAGUAGAUAAAAUAUUGGUACCUGAUGAAUCUGAGGCCAGGUUUCAAUACUUUAUCUGCUCUUCAUUUCCCCAUAUCUACUUAC

>hsa-mir-578 MI0003585

AGAUAAAUCUAUAGACAAAAUACAAUCCCGGACAACAAGAAGCUCCUAUAGCUCCUGUAGCUUCUUGUGCUCUAGGAUUGUAUUUUGUUUAUAUAU

>hsa-mir-579 MI0003586

CAUAUUAGGUUAAUGCAAAAGUAAUCGCGGUUUGUGCCAGAUGACGAUUUGAAUUAAUAAAUUCAUUUGGUAUAAACCGCGAUUAUUUUUGCAUCAAC

>hsa-mir-580 MI0003587

AUAAAAUUUCCAAUUGGAACCUAAUGAUUCAUCAGACUCAGAUAUUUAAGUUAACAGUAUUUGAGAAUGAUGAAUCAUUAGGUUCCGGUCAGAAAUU

>hsa-mir-581 MI0003588

GUUAUGUGAAGGUAUUCUUGUGUUCUCUAGAUCAGUGCUUUUAGAAAAUUUGUGUGAUCUAAAGAACACAAAGAAUACCUACACAGAACCACCUGC

>hsa-mir-582 MI0003589

AUCUGUGCUCUUUGAUUACAGUUGUUCAACCAGUUACUAAUCUAACUAAUUGUAACUGGUUGAACAACUGAACCCAAAGGGUGCAAAGUAGAAACAUU

>hsa-mir-583 MI0003590

AACUCACACAUUAACCAAAGAGGAAGGUCCCAUUACUGCAGGGAUCUUAGCAGUACUGGGACCUACCUCUUUGGU

>hsa-mir-584 MI0003591

UAGGGUGACCAGCCAUUAUGGUUUGCCUGGGACUGAGGAAUUUGCUGGGAUAUGUCAGUUCCAGGCCAACCAGGCUGGUUGGUCUCCCUGAAGCAAC

>hsa-mir-585 MI0003592

UGGGGUGUCUGUGCUAUGGCAGCCCUAGCACACAGAUACGCCCAGAGAAAGCCUGAACGUUGGGCGUAUCUGUAUGCUAGGGCUGCUGUAACAA

>hsa-mir-586 MI0003594

AUGGGGUAAAACCAUUAUGCAUUGUAUUUUUAGGUCCCAAUACAUGUGGGCCCUAAAAAUACAAUGCAUAAUGGUUUUUCACUCUUUAUCUUCUUAU

>hsa-mir-587 MI0003595

CUCCUAUGCACCCUCUUUCCAUAGGUGAUGAGUCACAGGGCUCAGGGAAUGUGUCUGCACCUGUGACUCAUCACCAGUGGAAAGCCCAUCCCAUAU

>hsa-mir-588 MI0003597

AGCUUAGGUACCAAUUUGGCCACAAUGGGUUAGAACACUAUUCCAUUGUGUUCUUACCCACCAUGGCCAAAAUUGGGCCUAAG

>hsa-mir-589 MI0003599

UCCAGCCUGUGCCCAGCAGCCCCUGAGAACCACGUCUGCUCUGAGCUGGGUACUGCCUGUUCAGAACAAAUGCCGGUUCCCAGACGCUGCCAGCUGGCC

>hsa-mir-590 MI0003602

UAGCCAGUCAGAAAUGAGCUUAUUCAUAAAAGUGCAGUAUGGUGAAGUCAAUCUGUAAUUUUAUGUAUAAGCUAGUCUCUGAUUGAAACAUGCAGCA

>hsa-mir-591 MI0003603

UCUUAUCAAUGAGGUAGACCAUGGGUUCUCAUUGUAAUAGUGUAGAAUGUUGGUUAACUGUGGACUCCCUGGCUCUGUCUCAAAUCUACUGAUUC

>hsa-mir-592 MI0003604

UAUUAUGCCAUGACAUUGUGUCAAUAUGCGAUGAUGUGUUGUGAUGGCACAGCGUCAUCACGUGGUGACGCAACAUCAUGACGUAAGACGUCACAAC

>hsa-mir-593 MI0003605

CCCCCAGAAUCUGUCAGGCACCAGCCAGGCAUUGCUCAGCCCGUUUCCCUCUGGGGGAGCAAGGAGUGGUGCUGGGUUUGUCUCUGCUGGGGUUUCUCCU

>hsa-mir-595 MI0003607

ACGGAAGCCUGCACGCAUUUAACACCAGCACGCUCAAUGUAGUCUUGUAAGGAACAGGUUGAAGUGUGCCGUGGUGUGUCUGGAGGAAGCGCCUGU

>hsa-mir-596 MI0003608

AGCACGGCCUCUCCGAAGCCUGCCCGGCUCCUCGGGAACCUGCCUCCCGCAUGGCAGCUGCUGCCCUUCGGAGGCCG

>hsa-mir-597 MI0003609

UACUUACUCUACGUGUGUGUCACUCGAUGACCACUGUGAAGACAGUAAAAUGUACAGUGGUUCUCUUGUGGCUCAAGCGUAAUGUAGAGUACUGGUC

>hsa-mir-598 MI0003610

GCUUGAUGAUGCUGCUGAUGCUGGCGGUGAUCCCGAUGGUGUGAGCUGGAAAUGGGGUGCUACGUCAUCGUUGUCAUCGUCAUCAUCAUCAUCCGAG

>hsa-mir-599 MI0003611

AAAGACAUGCUGUCCACAGUGUGUUUGAUAAGCUGACAUGGGACAGGGAUUCUUUUCACUGUUGUGUCAGUUUAUCAAACCCAUACUUGGAUGAC

>hsa-mir-600 MI0003613

AAGUCACGUGCUGUGGCUCCAGCUUCAUAGGAAGGCUCUUGUCUGUCAGGCAGUGGAGUUACUUACAGACAAGAGCCUUGCUCAGGCCAGCCCUGCCC

>hsa-mir-601 MI0003614

UGCAUGAGUUCGUCUUGGUCUAGGAUUGUUGGAGGAGUCAGAAAAACUACCCCAGGGAUCCUGAAGUCCUUUGGGUGGA

>hsa-mir-602 MI0003615

UUCUCACCCCCGCCUGACACGGGCGACAGCUGCGGCCCGCUGUGUUCACUCGGGCCGAGUGCGUCUCCUGUCAGGCAAGGGAGAGCAGAGCCCCCCUG

>hsa-mir-604 MI0003617

AGAGCAUCGUGCUUGACCUUCCACGCUCUCGUGUCCACUAGCAGGCAGGUUUUCUGACACAGGCUGCGGAAUUCAGGACAGUGCAUCAUGGAGA

>hsa-mir-605 MI0003618

GCCCUAGCUUGGUUCUAAAUCCCAUGGUGCCUUCUCCUUGGGAAAAACAGAGAAGGCACUAUGAGAUUUAGAAUCAAGUUAGG

>hsa-mir-606 MI0003619

UGUAUCCUUGGUUUUUAGUAGUUUUACUAUGAUGAGGUGUGCCAUCCACCCCAUCAUAGUAAACUACUGAAAAUCAAAGAUACAAGUGCCUGACCA

>hsa-mir-607 MI0003620

UUGCCUAAAGUCACACAGGUUAUAGAUCUGGAUUGGAACCCAGGGAGCCAGACUGCCUGGGUUCAAAUCCAGAUCUAUAACUUGUGUGACUUUGGG

>hsa-mir-608 MI0003621

GGGCCAAGGUGGGCCAGGGGUGGUGUUGGGACAGCUCCGUUUAAAAAGGCAUCUCCAAGAGCUUCCAUCAAAGGCUGCCUCUUGGUGCAGCACAGGUAGA

>hsa-mir-609 MI0003622

UGCUCGGCUGUUCCUAGGGUGUUUCUCUCAUCUCUGGUCUAUAAUGGGUUAAAUAGUAGAGAUGAGGGCAACACCCUAGGAACAGCAGAGGAACC

>hsa-mir-610 MI0003623

UCUAUUUGUCUUAGGUGAGCUAAAUGUGUGCUGGGACACAUUUGAGCCAAAUGUCCCAGCACACAUUUAGCUCACAUAAGAAAAAUGGACUCUAGU

>hsa-mir-611 MI0003624

AAAAUGGUGAGAGCGUUGAGGGGAGUUCCAGACGGAGAUGCGAGGACCCCUCGGGGUCUGACCCACA

>hsa-mir-612 MI0003625

UCCCAUCUGGACCCUGCUGGGCAGGGCUUCUGAGCUCCUUAGCACUAGCAGGAGGGGCUCCAGGGGCCCUCCCUCCAUGGCAGCCAGGACAGGACUCUCA

>hsa-mir-613 MI0003626

GGUGAGUGCGUUUCCAAGUGUGAAGGGACCCUUCCUGUAGUGUCUUAUAUACAAUACAGUAGGAAUGUUCCUUCUUUGCCACUCAUACACCUUUA

>hsa-mir-614 MI0003627

UCUAAGAAACGCAGUGGUCUCUGAAGCCUGCAGGGGCAGGCCAGCCCUGCACUGAACGCCUGUUCUUGCCAGGUGGCAGAAGGUUGCUGC

>hsa-mir-615 MI0003628

CUCGGGAGGGGCGGGAGGGGGGUCCCCGGUGCUCGGAUCUCGAGGGUGCUUAUUGUUCGGUCCGAGCCUGGGUCUCCCUCUUCCCCCCAACCCCCC

>hsa-mir-616 MI0003629

UUAGGUAAUUCCUCCACUCAAAACCCUUCAGUGACUUCCAUGACAUGAAAUAGGAAGUCAUUGGAGGGUUUGAGCAGAGGAAUGACCUGUUUUAAAA

>hsa-mir-617 MI0003631

CAUCAUAAGGAGCCUAGACUUCCCAUUUGAAGGUGGCCAUUUCCUACCACCUUCAAAUGGUAAGUCCAGGCUCCUUCUGAUUCAAUAAAUGAGGAGC

>hsa-mir-618 MI0003632

CUCUUGUUCACAGCCAAACUCUACUUGUCCUUCUGAGUGUAAUUACGUACAUGCAGUAGCUCAGGAGACAAGCAGGUUUACCCUGUGGAUGAGUCUGA

>hsa-mir-619 MI0003633

CGCCCACCUCAGCCUCCCAAAAUGCUGGGAUUACAGGCAUGAGCCACUGCGGUCGACCAUGACCUGGACAUGUUUGUGCCCAGUACUGUCAGUUUGCAG

>hsa-mir-620 MI0003634

AUAUAUAUCUAUAUCUAGCUCCGUAUAUAUAUAUAUAUAUAUAUAGAUAUCUCCAUAUAUAUGGAGAUAGAUAUAGAAAUAAAACAAGCAAAGAA

>hsa-mir-621 MI0003635

UAGAUUGAGGAAGGGGCUGAGUGGUAGGCGGUGCUGCUGUGCUCUGAUGAAGACCCAUGUGGCUAGCAACAGCGCUUACCUUUUGUCUCUGGGUCC

>hsa-mir-622 MI0003636

AGAGAAGCUGGACAAGUACUGGUCUCAGCAGAUUGAGGAGAGCACCACAGUGGUCAUCACACAGUCUGCUGAGGUUGGAGCUGCUGAGAUGACACU

>hsa-mir-623 MI0003637

GUACACAGUAGAAGCAUCCCUUGCAGGGGCUGUUGGGUUGCAUCCUAAGCUGUGCUGGAGCUUCCCGAUGUACUCUGUAGAUGUCUUUGCACCUUCUG

>hsa-mir-624 MI0003638

AAUGCUGUUUCAAGGUAGUACCAGUACCUUGUGUUCAGUGGAACCAAGGUAAACACAAGGUAUUGGUAUUACCUUGAGAUAGCAUUACACCUAAGUG

>hsa-mir-625 MI0003639

AGGGUAGAGGGAUGAGGGGGAAAGUUCUAUAGUCCUGUAAUUAGAUCUCAGGACUAUAGAACUUUCCCCCUCAUCCCUCUGCCCU

>hsa-mir-626 MI0003640

ACUGAUAUAUUUGUCUUAUUUGAGAGCUGAGGAGUAUUUUUAUGCAAUCUGAAUGAUCUCAGCUGUCUGAAAAUGUCUUCAAUUUUAAAGGCUU

>hsa-mir-627 MI0003641

UACUUAUUACUGGUAGUGAGUCUCUAAGAAAAGAGGAGGUGGUUGUUUUCCUCCUCUUUUCUUUGAGACUCACUACCAAUAAUAAGAAAUACUACUA

>hsa-mir-628 MI0003642

AUAGCUGUUGUGUCACUUCCUCAUGCUGACAUAUUUACUAGAGGGUAAAAUUAAUAACCUUCUAGUAAGAGUGGCAGUCGAAGGGAAGGGCUCAU

>hsa-mir-629 MI0003643

UCCCUUUCCCAGGGGAGGGGCUGGGUUUACGUUGGGAGAACUUUUACGGUGAACCAGGAGGUUCUCCCAACGUAAGCCCAGCCCCUCCCCUCUGCCU

>hsa-mir-630 MI0003644

AACUUAACAUCAUGCUACCUCUUUGUAUCAUAUUUUGUUAUUCUGGUCACAGAAUGACCUAGUAUUCUGUACCAGGGAAGGUAGUUCUUAACUAUAU

>hsa-mir-631 MI0003645

GUGGGGAGCCUGGUUAGACCUGGCCCAGACCUCAGCUACACAAGCUGAUGGACUGAGUCAGGGGCCACACUCUCC

>hsa-mir-632 MI0003647

CGCCUCCUACCGCAGUGCUUGACGGGAGGCGGAGCGGGGAACGAGGCCGUCGGCCAUUUUGUGUCUGCUUCCUGUGGGACGUGGUGGUAGCCGU

>hsa-mir-633 MI0003648

AACCUCUCUUAGCCUCUGUUUCUUUAUUGCGGUAGAUACUAUUAACCUAAAAUGAGAAGGCUAAUAGUAUCUACCACAAUAAAAUUGUUGUGAGGAUA

>hsa-mir-634 MI0003649

AAACCCACACCACUGCAUUUUGGCCAUCGAGGGUUGGGGCUUGGUGUCAUGCCCCAAGAUAACCAGCACCCCAACUUUGGACAGCAUGGAUUAGUCU

>hsa-mir-635 MI0003650

CAGAGAGGAGCUGCCACUUGGGCACUGAAACAAUGUCCAUUAGGCUUUGUUAUGGAAACUUCUCCUGAUCAUUGUUUUGUGUCCAUUGAGCUUCCAAU

>hsa-mir-636 MI0003651

UGGCGGCCUGGGCGGGAGCGCGCGGGCGGGGCCGGCCCCGCUGCCUGGAAUUAACCCCGCUGUGCUUGCUCGUCCCGCCCGCAGCCCUAGGCGGCGUCG

>hsa-mir-637 MI0003652

UGGCUAAGGUGUUGGCUCGGGCUCCCCACUGCAGUUACCCUCCCCUCGGCGUUACUGAGCACUGGGGGCUUUCGGGCUCUGCGUCUGCACAGAUACUUC

>hsa-mir-638 MI0003653

GUGAGCGGGCGCGGCAGGGAUCGCGGGCGGGUGGCGGCCUAGGGCGCGGAGGGCGGACCGGGAAUGGCGCGCCGUGCGCCGCCGGCGUAACUGCGGCGCU

>hsa-mir-639 MI0003654

UGGCCGACGGGGCGCGCGCGGCCUGGAGGGGCGGGGCGGACGCAGAGCCGCGUUUAGUCUAUCGCUGCGGUUGCGAGCGCUGUAGGGAGCCUGUGCUG

>hsa-mir-640 MI0003655

GUGACCCUGGGCAAGUUCCUGAAGAUCAGACACAUCAGAUCCCUUAUCUGUAAAAUGGGCAUGAUCCAGGAACCUGCCUCUACGGUUGCCUUGGGG

>hsa-mir-641 MI0003656

UGGGUGAAAGGAAGGAAAGACAUAGGAUAGAGUCACCUCUGUCCUCUGUCCUCUACCUAUAGAGGUGACUGUCCUAUGUCUUUCCUUCCUCUUACCCCU

>hsa-mir-642a MI0003657

AUCUGAGUUGGGAGGGUCCCUCUCCAAAUGUGUCUUGGGGUGGGGGAUCAAGACACAUUUGGAGAGGGAACCUCCCAACUCGGCCUCUGCCAUCAUU

>hsa-mir-643 MI0003658

ACCAAGUGAUAUUCAUUGUCUACCUGAGCUAGAAUACAAGUAGUUGGCGUCUUCAGAGACACUUGUAUGCUAGCUCAGGUAGAUAUUGAAUGAAAAA

>hsa-mir-644a MI0003659

UUUUUUUUUAGUAUUUUUCCAUCAGUGUUCAUAAGGAAUGUUGCUCUGUAGUUUUCUUAUAGUGUGGCUUUCUUAGAGCAAAGAUGGUUCCCUA

>hsa-mir-645 MI0003660

CAGUUCCUAACAGGCCUCAGACCAGUACCGGUCUGUGGCCUGGGGGUUGAGGACCCCUGCUCUAGGCUGGUACUGCUGAUGCUUAAAAAGAGAG

>hsa-mir-646 MI0003661

GAUCAGGAGUCUGCCAGUGGAGUCAGCACACCUGCUUUUCACCUGUGAUCCCAGGAGAGGAAGCAGCUGCCUCUGAGGCCUCAGGCUCAGUGGC

>hsa-mir-647 MI0003662

AGGAAGUGUUGGCCUGUGGCUGCACUCACUUCCUUCAGCCCCAGGAAGCCUUGGUCGGGGGCAGGAGGGAGGGUCAGGCAGGGCUGGGGGCCUGAC

>hsa-mir-648 MI0003663

AUCACAGACACCUCCAAGUGUGCAGGGCACUGGUGGGGGCCGGGGCAGGCCCAGCGAAAGUGCAGGACCUGGCACUUAGUCGGAAGUGAGGGUG

>hsa-mir-649 MI0003664

GGCCUAGCCAAAUACUGUAUUUUUGAUCGACAUUUGGUUGAAAAAUAUCUAUGUAUUAGUAAACCUGUGUUGUUCAAGAGUCCACUGUGUUUUGCUG

>hsa-mir-650 MI0003665

CAGUGCUGGGGUCUCAGGAGGCAGCGCUCUCAGGACGUCACCACCAUGGCCUGGGCUCUGCUCCUCCUCACCCUCCUCACUCAGGGCACAGGUGAU

>hsa-mir-651 MI0003666

AAUCUAUCACUGCUUUUUAGGAUAAGCUUGACUUUUGUUCAAAUAAAAAUGCAAAAGGAAAGUGUAUCCUAAAAGGCAAUGACAGUUUAAUGUGUUU

>hsa-mir-652 MI0003667

ACGAAUGGCUAUGCACUGCACAACCCUAGGAGAGGGUGCCAUUCACAUAGACUAUAAUUGAAUGGCGCCACUAGGGUUGUGCAGUGCACAACCUACAC

>hsa-mir-653 MI0003674

UUCAUUCCUUCAGUGUUGAAACAAUCUCUACUGAACCAGCUUCAAACAAGUUCACUGGAGUUUGUUUCAAUAUUGCAAGAAUGAUAAGAUGGAAGC

>hsa-mir-654 MI0003676

GGGUAAGUGGAAAGAUGGUGGGCCGCAGAACAUGUGCUGAGUUCGUGCCAUAUGUCUGCUGACCAUCACCUUUAGAAGCCC

>hsa-mir-655 MI0003677

AACUAUGCAAGGAUAUUUGAGGAGAGGUUAUCCGUGUUAUGUUCGCUUCAUUCAUCAUGAAUAAUACAUGGUUAACCUCUUUUUGAAUAUCAGACUC

>hsa-mir-656 MI0003678

CUGAAAUAGGUUGCCUGUGAGGUGUUCACUUUCUAUAUGAUGAAUAUUAUACAGUCAACCUCUUUCCGAUAUCGAAUC

>hsa-mir-657 MI0003681

GUGUAGUAGAGCUAGGAGGAGAGGGUCCUGGAGAAGCGUGGACCGGUCCGGGUGGGUUCCGGCAGGUUCUCACCCUCUCUAGGCCCCAUUCUCCUCUG

>hsa-mir-658 MI0003682

GCUCGGUUGCCGUGGUUGCGGGCCCUGCCCGCCCGCCAGCUCGCUGACAGCACGACUCAGGGCGGAGGGAAGUAGGUCCGUUGGUCGGUCGGGAACGAGG

>hsa-mir-659 MI0003683

UACCGACCCUCGAUUUGGUUCAGGACCUUCCCUGAACCAAGGAAGAGUCACAGUCUCUUCCUUGGUUCAGGGAGGGUCCCCAACAAUGUCCUCAUGG

>hsa-mir-660 MI0003684

CUGCUCCUUCUCCCAUACCCAUUGCAUAUCGGAGUUGUGAAUUCUCAAAACACCUCCUGUGUGCAUGGAUUACAGGAGGGUGAGCCUUGUCAUCGUG

>hsa-mir-661 MI0003669

GGAGAGGCUGUGCUGUGGGGCAGGCGCAGGCCUGAGCCCUGGUUUCGGGCUGCCUGGGUCUCUGGCCUGCGCGUGACUUUGGGGUGGCU

>hsa-mir-662 MI0003670

GCUGUUGAGGCUGCGCAGCCAGGCCCUGACGGUGGGGUGGCUGCGGGCCUUCUGAAGGUCUCCCACGUUGUGGCCCAGCAGCGCAGUCACGUUGC

>hsa-mir-663b MI0006336

GGUGCCGAGGGCCGUCCGGCAUCCUAGGCGGGUCGCUGCGGUACCUCCCUCCUGUCUGUGGCGGUGGGAUCCCGUGGCCGUGUUUUCCUGGUGGCCCGGCCGUGCCUGAGGUUUC

>hsa-mir-664a MI0006442

GAACAUUGAAACUGGCUAGGGAAAAUGAUUGGAUAGAAACUAUUAUUCUAUUCAUUUAUCCCCAGCCUACAAAAUGAAAAAA

>hsa-mir-665 MI0005563

UCUCCUCGAGGGGUCUCUGCCUCUACCCAGGACUCUUUCAUGACCAGGAGGCUGAGGCCCCUCACAGGCGGC

>hsa-mir-668 MI0003761

GGUAAGUGCGCCUCGGGUGAGCAUGCACUUAAUGUGGGUGUAUGUCACUCGGCUCGGCCCACUACC

>hsa-mir-670 MI0003933

GUUUAGGGGUGGACCUGAUGUCCCUGAGUGUAUGUGGUGAACCUGAAUUUGCCUUGGGUUUCCUCAUAUUCAUUCAGGAGUGUCAGUUGCCCCUUCAC

>hsa-mir-671 MI0003760

GCAGGUGAACUGGCAGGCCAGGAAGAGGAGGAAGCCCUGGAGGGGCUGGAGGUGAUGGAUGUUUUCCUCCGGUUCUCAGGGCUCCACCUCUUUCGGGCCGUAGAGCCAGGGCUGGUGC

>hsa-mir-675 MI0005416

CCCAGGGUCUGGUGCGGAGAGGGCCCACAGUGGACUUGGUGACGCUGUAUGCCCUCACCGCUCAGCCCCUGGG

>hsa-mir-676 MI0016436

GCAUGACUCUUCAACCUCAGGACUUGCAGAAUUAAUGGAAUGCUGUCCUAAGGUUGUUGAGUUGUGC

>hsa-mir-708 MI0005543

AACUGCCCUCAAGGAGCUUACAAUCUAGCUGGGGGUAAAUGACUUGCACAUGAACACAACUAGACUGUGAGCUUCUAGAGGGCAGGGA

>hsa-mir-711 MI0012488

ACUGACUUUGAGUCUCUCCUCAGGGUGCUGCAGGCAAAGCUGGGGACCCAGGGAGAGACGUAAGUGAGGGGAGAUG

>hsa-mir-718 MI0012489

GGCCGCGGCGCGCAAGAUGGCGGCGGGCCCGGGCACCGCCCCUUCCGCCCCGCCGGGCGUCGCACGAGGC

>hsa-mir-744 MI0005559

UUGGGCAAGGUGCGGGGCUAGGGCUAACAGCAGUCUUACUGAAGGUUUCCUGGAAACCACGCACAUGCUGUUGCCACUAACCUCAACCUUACUCGGUC

>hsa-mir-758 MI0003757

GCCUGGAUACAUGAGAUGGUUGACCAGAGAGCACACGCUUUAUUUGUGCCGUUUGUGACCUGGUCCACUAACCCUCAGUAUCUAAUGC

>hsa-mir-759 MI0004065

UAAUAAAUUAAAUGCCUAAACUGGCAGAGUGCAAACAAUUUUGACUCAGAUCUAAAUGUUUGCACUGGCUGUUUAAACAUUUAAUUUGUUA

>hsa-mir-760 MI0005567

GGCGCGUCGCCCCCCUCAGUCCACCAGAGCCCGGAUACCUCAGAAAUUCGGCUCUGGGUCUGUGGGGAGCGAAAUGCAAC

>hsa-mir-761 MI0003941

GGAGGAGCAGCAGGGUGAAACUGACACAGUUCUGGUGAGUUUCACUUUGCUGCUCCUCC

>hsa-mir-762 MI0003892

GGCCCGGCUCCGGGUCUCGGCCCGUACAGUCCGGCCGGCCAUGCUGGCGGGGCUGGGGCCGGGGCCGAGCCCGCGGCGGGGCC

>hsa-mir-764 MI0003944

AAUCUAGGAGGCAGGUGCUCACUUGUCCUCCUCCAUGCUUGGAAAAUGCAGGGAGGAGGCCAUAGUGGCAACUGUUACCAUGAUU

>hsa-mir-765 MI0005116

UUUAGGCGCUGAUGAAAGUGGAGUUCAGUAGACAGCCCUUUUCAAGCCCUACGAGAAACUGGGGUUUCUGGAGGAGAAGGAAGGUGAUGAAGGAUCUGUUCUCGUGAGCCUGAA

>hsa-mir-766 MI0003836

GCAUCCUCAGGACCUGGGCUUGGGUGGUAGGAGGAAUUGGUGCUGGUCUUUCAUUUUGGAUUUGACUCCAGCCCCACAGCCUCAGCCACCCCAGCCAAUUGUCAUAGGAGC

>hsa-mir-767 MI0003763

GCUUUUAUAUUGUAGGUUUUUGCUCAUGCACCAUGGUUGUCUGAGCAUGCAGCAUGCUUGUCUGCUCAUACCCCAUGGUUUCUGAGCAGGAACCUUCAUUGUCUACUGC

>hsa-mir-769 MI0003834

GCCUUGGUGCUGAUUCCUGGGCUCUGACCUGAGACCUCUGGGUUCUGAGCUGUGAUGUUGCUCUCGAGCUGGGAUCUCCGGGGUCUUGGUUCAGGGCCGGGGCCUCUGGGUUCCAAGC

>hsa-mir-770 MI0005118

AGGAGCCACCUUCCGAGCCUCCAGUACCACGUGUCAGGGCCACAUGAGCUGGGCCUCGUGGGCCUGAUGUGGUGCUGGGGCCUCAGGGGUCUGCUCUU

>hsa-mir-802 MI0003906

GUUCUGUUAUUUGCAGUCAGUAACAAAGAUUCAUCCUUGUGUCCAUCAUGCAACAAGGAGAAUCUUUGUCACUUAGUGUAAUUAAUAGCUGGAC

>hsa-mir-873 MI0005564

GUGUGCAUUUGCAGGAACUUGUGAGUCUCCUAUUGAAAAUGAACAGGAGACUGAUGAGUUCCCGGGAACACCCACAA

>hsa-mir-874 MI0005532

UUAGCCCUGCGGCCCCACGCACCAGGGUAAGAGAGACUCUCGCUUCCUGCCCUGGCCCGAGGGACCGACUGGCUGGGC

>hsa-mir-875 MI0005541

UUAGUGGUACUAUACCUCAGUUUUAUCAGGUGUUCUUAAAAUCACCUGGAAACACUGAGGUUGUGUCUCACUGAAC

>hsa-mir-876 MI0005542

UGAAGUGCUGUGGAUUUCUUUGUGAAUCACCAUAUCUAAGCUAAUGUGGUGGUGGUUUACAAAGUAAUUCAUAGUGCUUCA

>hsa-mir-877 MI0005561

GUAGAGGAGAUGGCGCAGGGGACACGGGCAAAGACUUGGGGGUUCCUGGGACCCUCAGACGUGUGUCCUCUUCUCCCUCCUCCCAG

>hsa-mir-885 MI0005560

CCGCACUCUCUCCAUUACACUACCCUGCCUCUUCUCCAUGAGAGGCAGCGGGGUGUAGUGGAUAGAGCACGGGU

>hsa-mir-887 MI0005562

GUGCAGAUCCUUGGGAGCCCUGUUAGACUCUGGAUUUUACACUUGGAGUGAACGGGCGCCAUCCCGAGGCUUUGCACAG

>hsa-mir-888 MI0005537

GGCAGUGCUCUACUCAAAAAGCUGUCAGUCACUUAGAUUACAUGUGACUGACACCUCUUUGGGUGAAGGAAGGCUCA

>hsa-mir-889 MI0005540

GUGCUUAAAGAAUGGCUGUCCGUAGUAUGGUCUCUAUAUUUAUGAUGAUUAAUAUCGGACAACCAUUGUUUUAGUAUCC

>hsa-mir-890 MI0005533

GGAAGUGCCCUACUUGGAAAGGCAUCAGUUGCUUAGAUUACAUGUAACUAUUCCCUUUCUGAGUAGAGUAAGUCUUA

>hsa-mir-891a MI0005524

CCUUAAUCCUUGCAACGAACCUGAGCCACUGAUUCAGUAAAAUACUCAGUGGCACAUGUUUGUUGUGAGGGUCAAAAGA

>hsa-mir-892b MI0005538

UGCAAUGCCCUACUCAGAAAGGUGCCAUUUAUGUAGAUUUUAUGUCACUGGCUCCUUUCUGGGUAGAGCAAGGCUCA

>hsa-mir-920 MI0005712

GUAGUUGUUCUACAGAAGACCUGGAUGUGUAGGAGCUAAGACACACUCCAGGGGAGCUGUGGAAGCAGUAACACG

>hsa-mir-921 MI0005713

ACUAGUGAGGGACAGAACCAGGAUUCAGACUCAGGUCCAUGGGCCUGGAUCACUGG

>hsa-mir-922 MI0005714

AUGGCGUUUUCCCUCUCCCUGUCCUGGACUGGGGUCAGACUGUGCCCCGAGGAGAAGCAGCAGAGAAUAGGACUACGUCAU

>hsa-mir-924 MI0005716

AAUAGAGUCUUGUGAUGUCUUGCUUAAGGGCCAUCCAACCUAGAGUCUACAAC

>hsa-mir-933 MI0005755

ACUUGGGUCAGUUCAGAGGUCCUCGGGGCGCGCGUCGAGUCAGCCGUGUGCGCAGGGAGACCUCUCCCACCCACAGU

>hsa-mir-934 MI0005756

AGAAAUAAGGCUUCUGUCUACUACUGGAGACACUGGUAGUAUAAAACCCAGAGUCUCCAGUAAUGGACGGGAGCCUUAUUUCU

>hsa-mir-935 MI0005757

GGCGGGGGCGCGGGCGGCAGUGGCGGGAGCGGCCCCUCGGCCAUCCUCCGUCUGCCCAGUUACCGCUUCCGCUACCGCCGCCGCUCCCGCU

>hsa-mir-936 MI0005758

UCAAGGCCACUGGGACAGUAGAGGGAGGAAUCGCAGAAAUCACUCCAGGAGCAACUGAGAGACCUUGCUUCUACUUUACCAGGUCCUGCUGGCCCAGA

>hsa-mir-937 MI0005759

AGCACUGCCCCCGGUGAGUCAGGGUGGGGCUGGCCCCCUGCUUCGUGCCCAUCCGCGCUCUGACUCUCUGCCCACCUGCAGGAGCU

>hsa-mir-938 MI0005760

GAAGGUGUACCAUGUGCCCUUAAAGGUGAACCCAGUGCACCUUCAUGAACCGUGGUACACCUUUAAGAACUUGGUAUGCCUUC

>hsa-mir-939 MI0005761

UGUGGGCAGGGCCCUGGGGAGCUGAGGCUCUGGGGGUGGCCGGGGCUGACCCUGGGCCUCUGCUCCCCAGUGUCUGACCGCG

>hsa-mir-940 MI0005762

GUGAGGUGUGGGCCCGGCCCCAGGAGCGGGGCCUGGGCAGCCCCGUGUGUUGAGGAAGGAAGGCAGGGCCCCCGCUCCCCGGGCCUGACCCCAC

>hsa-mir-941-1 MI0005763

UGUGGACAUGUGCCCAGGGCCCGGGACAGCGCCACGGAAGAGGACGCACCCGGCUGUGUGCACAUGUGCCCA

>hsa-mir-942 MI0005767

AUUAGGAGAGUAUCUUCUCUGUUUUGGCCAUGUGUGUACUCACAGCCCCUCACACAUGGCCGAAACAGAGAAGUUACUUUCCUAAU

>hsa-mir-943 MI0005768

GGGACGUUCUGAGCUCGGGGUGGGGGACGUUUGCCGGUCACUGCUGCUGGCGCCCUGACUGUUGCCGUCCUCCAGCCCCACUCAAAGGCAUCCC

>hsa-mir-944 MI0005769

GUUCCAGACACAUCUCAUCUGAUAUACAAUAUUUUCUUAAAUUGUAUAAAGAGAAAUUAUUGUACAUCGGAUGAGCUGUGUCUGGGAU

>hsa-mir-1178 MI0006271

GCGUUGGCUGGCAGAGGAAGGGAAGGGUCCAGGGUCAGCUGAGCAUGCCCUCAGGUUGCUCACUGUUCUUCCCUAGAAUGUCAGGUGAUGU

>hsa-mir-1179 MI0006272

GGCUGGAAAGGAAGAAGCAUUCUUUCAUUGGUUGGUGUGUAUUGCCUUGUCAACCAAUAAGAGGAUGCCAUUUAUCCUUUUCUGACUAGCU

>hsa-mir-1180 MI0006273

GCUGCUGGACCCACCCGGCCGGGAAUAGUGCUCCUGGUUGUUUCCGGCUCGCGUGGGUGUGUCGGCGGC

>hsa-mir-1181 MI0006274

UCCACUGCUGCCGCCGUCGCCGCCACCCGAGCCGGAGCGGGCUGGGCCGCCAAGGCAAGAUGGUGGACUACAGCGUGUGGG

>hsa-mir-1182 MI0006275

GGGACUUGUCACUGCCUGUCUCCUCCCUCUCCAGCAGCGACUGGAUUCUGGAGUCCAUCUAGAGGGUCUUGGGAGGGAUGUGACUGUUGGGAAGCCC

>hsa-mir-1183 MI0006276

AUUAUUCAAAUGCUCGGAGACACAGAACAUUAGAGAAGACAGGAGUUCACUGUAGGUGAUGGUGAGAGUGGGCAUGGAGCAGGAGUGCC

>hsa-mir-1184-1 MI0006277

CUUGCAGAACGAGGUGAAGGAGGUGGUUCUGCUCAGCAGUCAACAGUGGCCACAUCUCCACCUGCAGCGACUUGAUGGCUUCCGUGUCCUUUUCGUGGG

>hsa-mir-1185-1 MI0003844

UUUGGUACUUGAAGAGAGGAUACCCUUUGUAUGUUCACUUGAUUAAUGGCGAAUAUACAGGGGGAGACUCUUAUUUGCGUAUCAAA

>hsa-mir-1193 MI0014205

GUAGCUGAGGGGAUGGUAGACCGGUGACGUGCACUUCAUUUACGAUGUAGGUCACCCGUUUGACUAUCCACCAGCGCC

>hsa-mir-1197 MI0006656

ACUUCCUGGUAUUUGAAGAUGCGGUUGACCAUGGUGUGUACGCUUUAUUUGUGACGUAGGACACAUGGUCUACUUCUUCUCAAUAUCA

>hsa-mir-1199 MI0020340

AGCCUGCGCCGGAGCCGGGGCCUGAGCCCGGGCCGCGCAGGCCGUGAACUCGUCGAGCUGCGCGUGCGGCCGGUGCUCAACCUGCCGGGUCCUGGCCCCGCGCUCCCGCGCGCCCUGGA

>hsa-mir-1200 MI0006332

UGCUACUUCUCCUGAGCCAUUCUGAGCCUCAAUCACUUGCCAGAGAGAUUGGUUCAGGAAUUUGUCAGGGAUAGCC

>hsa-mir-1202 MI0006334

CCUGCUGCAGAGGUGCCAGCUGCAGUGGGGGAGGCACUGCCAGGGCUGCCCACUCUGCUUAGCCAGCAGGUGCCAAGAACAGG

>hsa-mir-1203 MI0006335

UCCUCCCCGGAGCCAGGAUGCAGCUCAAGCCACAGCAGGGUGUUUAGCGCUCUUCAGUGGCUCCAGAUUGUGGCGCUGGUGCAGG

>hsa-mir-1204 MI0006337

ACCUCGUGGCCUGGUCUCCAUUAUUUGAGAUGAGUUACAUCUUGGAGGUGAGGACGUGCCUCGUGGU

>hsa-mir-1205 MI0006338

GAAGGCCUCUGCAGGGUUUGCUUUGAGGUACUUCCUUCCUGUCAACCCUGUUCUGGAGUCUGU

>hsa-mir-1206 MI0006339

CAGUGUUCAUGUAGAUGUUUAAGCUCUUGCAGUAGGUUUUUGCAAGCUAGUGAACGCUG

>hsa-mir-1207 MI0006340

GCAGGGCUGGCAGGGAGGCUGGGAGGGGCUGGCUGGGUCUGGUAGUGGGCAUCAGCUGGCCCUCAUUUCUUAAGACAGCACUUCUGU

>hsa-mir-1208 MI0006341

CACCGGCAGAAUCACUGUUCAGACAGGCGGAGACGGGUCUUUCUCGCCCUCUGAUGAGUCACCACUGUGGUGG

>hsa-mir-1224 MI0003764

GUGAGGACUCGGGAGGUGGAGGGUGGUGCCGCCGGGGCCGGGCGCUGUUUCAGCUCGCUUCUCCCCCCACCUCCUCUCUCCUCAG

>hsa-mir-1225 MI0006311

GUGGGUACGGCCCAGUGGGGGGGAGAGGGACACGCCCUGGGCUCUGCCCAGGGUGCAGCCGGACUGACUGAGCCCCUGUGCCGCCCCCAG

>hsa-mir-1226 MI0006313

GUGAGGGCAUGCAGGCCUGGAUGGGGCAGCUGGGAUGGUCCAAAAGGGUGGCCUCACCAGCCCUGUGUUCCCUAG

>hsa-mir-1227 MI0006316

GUGGGGCCAGGCGGUGGUGGGCACUGCUGGGGUGGGCACAGCAGCCAUGCAGAGCGGGCAUUUGACCCCGUGCCACCCUUUUCCCCAG

>hsa-mir-1228 MI0006318

GUGGGCGGGGGCAGGUGUGUGGUGGGUGGUGGCCUGCGGUGAGCAGGGCCCUCACACCUGCCUCGCCCCCCAG

>hsa-mir-1229 MI0006319

GUGGGUAGGGUUUGGGGGAGAGCGUGGGCUGGGGUUCAGGGACACCCUCUCACCACUGCCCUCCCACAG

>hsa-mir-1231 MI0006321

GUCAGUGUCUGGGCGGACAGCUGCAGGAAAGGGAAGACCAAGGCUUGCUGUCUGUCCAGUCUGCCACCCUACCCUGUCUGUUCUUGCCACAG

>hsa-mir-1233-1 MI0006323

GUGAGUGGGAGGCCAGGGCACGGCAGGGGGAGCUGCAGGGCUAUGGGAGGGGCCCCAGCGUCUGAGCCCUGUCCUCCCGCAG

>hsa-mir-1234 MI0006324

GUGAGUGUGGGGUGGCUGGGGGGGGGGGGGGGGGGCCGGGGACGGCUUGGGCCUGCCUAGUCGGCCUGACCACCCACCCCACAG

>hsa-mir-1236 MI0006326

GUGAGUGACAGGGGAAAUGGGGAUGGACUGGAAGUGGGCAGCAUGGAGCUGACCUUCAUCAUGGCUUGGCCAACAUAAUGCCUCUUCCCCUUGUCUCUCCAG

>hsa-mir-1237 MI0006327

GUGGGAGGGCCCAGGCGCGGGCAGGGGUGGGGGUGGCAGAGCGCUGUCCCGGGGGCGGGGCCGAAGCGCGGCGACCGUAACUCCUUCUGCUCCGUCCCCCAG

>hsa-mir-1238 MI0006328

GUGAGUGGGAGCCCCAGUGUGUGGUUGGGGCCAUGGCGGGUGGGCAGCCCAGCCUCUGAGCCUUCCUCGUCUGUCUGCCCCAG

>hsa-mir-1243 MI0006373

CUAAAACUGGAUCAAUUAUAGGAGUGAAAUAAAGGUCCAUCUCCUGCCUAUUUAUUACUUUGCUUUGGUAAUAAAUCUAUUUUUAAAAGAACC

>hsa-mir-1244-1 MI0006379

AUCUUAUUCCGAGCAUUCCAGUAACUUUUUUGUGUAUGUACUUAGCUGUACUAUAAGUAGUUGGUUUGUAUGAGAUGGUUAAAAA

>hsa-mir-1245a MI0006380

AUUUAUGUAUAGGCCUUUAGAUCAUCUGAUGUUGAAUACUCUUUAAGUGAUCUAAAGGCCUACAUAUAAA

>hsa-mir-1245b MI0017431

UUUAUAUGUAGGCCUUUAGAUCACUUAAAGAGUAUUCAACAUCAGAUGAUCUAAAGGCCUAUACAUAAA

>hsa-mir-1246 MI0006381

UGUAUCCUUGAAUGGAUUUUUGGAGCAGGAGUGGACACCUGACCCAAAGGAAAUCAAUCCAUAGGCUAGCAAU

>hsa-mir-1247 MI0006382

CCGCUUGCCUCGCCCAGCGCAGCCCCGGCCGCUGGGCGCACCCGUCCCGUUCGUCCCCGGACGUUGCUCUCUACCCCGGGAACGUCGAGACUGGAGCGCCCGAACUGAGCCACCUUCGCGGACCCCGAGAGCGGCG

>hsa-mir-1248 MI0006383

UUUACCUUCUUGUAUAAGCACUGUGCUAAAAUUGCAGACACUAGGACCAUGUCUUGGUUUUUGCAAUAAUGCUAGCAGAGUACACACAAGAAGAAAAGUAACAGCA

>hsa-mir-1249 MI0006384

GGGAGGAGGGAGGAGAUGGGCCAAGUUCCCUCUGGCUGGAACGCCCUUCCCCCCCUUCUUCACCUG

>hsa-mir-1250 MI0006385

CUGUCCCGCUGGCCUGGCAGGUGACGGUGCUGGAUGUGGCCUUUUUGCCUUUUCUAAAGGCCACAUUUUCCAGCCCAUUCAACCUUCCAGAGCCCUCUGAAGUGGCCACAGGC

>hsa-mir-1251 MI0006386

GUGGACUCUAGCUGCCAAAGGCGCUUCUCCUUCUGAACAGAGCGCUUUGCUCAGCCAGUGUAGACAUGGC

>hsa-mir-1252 MI0006434

AGAAAGAAGGAAAUUGAAUUCAUUUAGAAAAGAGAAUUCCAAAUGAGCUUAAUUUCCUUUUUUCU

>hsa-mir-1253 MI0006387

AGCAGCAAGAGAUAGAAUCCAAAAGAGAAGAAGAUCAGCCUGCAGAUGUGGACUGCUAAAUGCAGGCUGAUCUUCUCCCCUUUGGGAUUCUCUUAUGAGAAGCCA

>hsa-mir-1254-1 MI0006388

GGUGGGAGGAUUGCUUGAGCCUGGAAGCUGGAGCCUGCAGUGAACUAUCAUUGUGCCACUGUACUCCAGCCUAGGCAACAAAAUGAAAUCCUGUCUA

>hsa-mir-1255a MI0006389

AUUGGAAAUCCUUUGAGUUGCUUCUCAAGGAUGAGCAAAGAAAGUAGAUUUUUUAGAUUCUAAAGAAACUAUCUUCUUUGCUCAUCCUUGAGAAGCAACUCCUUAUCCAUUAA

>hsa-mir-1255b-1 MI0006435

UACGGAUGAGCAAAGAAAGUGGUUUCUUAAAAUGGAAUCUACUCUUUGUGAAGAUGCUGUGAA

>hsa-mir-1255b-2 MI0006436

UCUUACGGAUGAGCAAAGAAAGUGGUUUGCGCCUCAAGAAACCACUUUCUUUGCUCAUCCAUAAGGA

>hsa-mir-1256 MI0006390

AGUCAGCCUGUUGAAGCUUUGAAGCUUUGAUGCCAGGCAUUGACUUCUCACUAGCUGUGAAAGUCCUAGCUAAAGAGAAGUCAAUGCAUGACAUCUUGUUUCAAUAGAUGGCUGUUUCA

>hsa-mir-1257 MI0006391

GCCCUGGGCUUGUGCUUGGGGAGUGAAUGAUGGGUUCUGACCCCCAUGCACCCCUGUGGGCCCCUGGCAUCACUGGCCCCAUCCUUCACCCCUGCCAACCACGCUUGCCCUGUGCCU

>hsa-mir-1258 MI0006392

CUGUGGCUUCCACGACCUAAUCCUAACUCCUGCGAGUCCCUGGAGUUAGGAUUAGGUCGUGGAAGCCACAGGA

>hsa-mir-1260a MI0006394

ACCUUUCCAGCUCAUCCCACCUCUGCCACCAAAACACUCAUCGCGGGGUCAGAGGGAGUGCCAAAAAAGGUAA

>hsa-mir-1260b MI0014197

UCUCCGUUUAUCCCACCACUGCCACCAUUAUUGCUACUGUUCAGCAGGUGCUGCUGGUGGUGAUGGUGAUAGUCUGGUGGGGGCGGUGG

>hsa-mir-1261 MI0006396

UGCUAUGGAUAAGGCUUUGGCUUAUGGGGAUAUUGUGGUUGAUCUGUUCUAUCCAGAUGACUGAAACUUUCUCCAUAGCAGC

>hsa-mir-1262 MI0006397

AUCUACAAUGGUGAUGGGUGAAUUUGUAGAAGGAUGAAAGUCAAAGAAUCCUUCUGGGAACUAAUUUUUGGCCUUCAACAAGAAUUGUGAUAU

>hsa-mir-1263 MI0006398

CUACCCCAAAAUAUGGUACCCUGGCAUACUGAGUAUUUUAAUACUGGCAUACUCAGUAUGCCAUGUUGCCAUAUUUUGGGGUAGCA

>hsa-mir-1264 MI0003758

AGGUCCUCAAUAAGUAUUUGUUGAAAGAAUAAAUAAACCAACAAGUCUUAUUUGAGCACCUGUUAUGUG

>hsa-mir-1265 MI0006401

AUGGUUUGGGACUCAGGAUGUGGUCAAGUGUUGUUAAGGCAUGUUCAGGAACAAUACUUGACCACAUUUUGAAUUCCAAACCAUAU

>hsa-mir-1266 MI0006403

ACAGGUAGUGUCCCUCAGGGCUGUAGAACAGGGCUGGGAUUACUAAAGCCCUGUUCUAUGCCCUGAGGGACACUGAGCAUGUCA

>hsa-mir-1267 MI0006404

CUCCCAAAUCUCCUGUUGAAGUGUAAUCCCCACCUCCAGCAUUGGGGAUUACAUUUCAACAUGAGAUUUGGAUGAGGA

>hsa-mir-1268a MI0006405

UAGCCGGGCGUGGUGGUGGGGGCCUGUGGUCCCAGCUACUUUGGAGGCUGAG

>hsa-mir-1268b MI0016748

ACCCGGGCGUGGUGGUGGGGGUGGGUGCCUGUAAUUCCAGCUAGUUGGGA

>hsa-mir-1269a MI0006406

UGGAUUGCCUAGACCAGGGAAGCCAGUUGGCAUGGCUCAGUCCAAGUCUGACCACCUGAGGAAUGCCUGGACUGAGCCGUGCUACUGGCUUCCCUGGUCUCCAGC

>hsa-mir-1269b MI0016888

UGAGGUUUCUGGACUGAGCCAUGCUACUGGCUUCUCUGGUUCUCCAGCUUACAGAUGGCUUAUCAUGGGACCUCU

>hsa-mir-1270-1 MI0006407

CACAGAGUUAUACUGGAGAUAUGGAAGAGCUGUGUUGGGUAUAAGUAACAGGCUUUUCUUUAUCUUCUAUGUGGCUCUUUGCA

>hsa-mir-1271 MI0003814

CACCCAGAUCAGUGCUUGGCACCUAGCAAGCACUCAGUAAAUAUUUGUUGAGUGCCUGCUAUGUGCCAGGCAUUGUGCUGAGGGCU

>hsa-mir-1272 MI0006408

CCAGAUCAGAUCUGGGUGCGAUGAUGAUGGCAGCAAAUUCUGAAAACGUGCUCAGUGUCUUUAUAACAGGAAAGCCGUAAACUUAGAAAUGUAGGCUGCAGCUCGUGUGCUCUGUGGUCUGGGCUGGUA

>hsa-mir-1273a MI0006409

UGAGGCAGGAGAAUUGCUUGAACCCGGGUGGUGGAGGUUGCAGUGAGCCAAGAUUGCGCCACUGCACUCCAGCCUGGGCGACAAAGCAAGACUCUUUCUUGGA

>hsa-mir-1273c MI0014171

UGCAGCCUGGGCGACAAAACGAGACCCUGUCUUUUUUUUUUUCUGAGACAGAGUCUCGUUCUGUUGCCCAAGCUGGA

>hsa-mir-1273f MI0018002

AGGUGGGAGGAUUGCUUGAGCCUGGGAGAUGGAGGUUGCAGUGAGCUGAGAUCACGCAACUGCACCCCCAGCCUGGGCCAUAGAGUCAGUCCUUGUCUC

>hsa-mir-1273g MI0018003

GAGGUGGGAGGAUUGCUUGAGUCAGGGUGGUUGAGGCUGCAGUAAGUUGUGAUCAUACCACUGCACUCCAGCCUGAGUGACAGAGCAAGACCUUGUCUCA

>hsa-mir-1273h MI0025512

UACUUGGGUGACUAAGGCAGGAUUGCUUGAGCCUGGGAGGUCAAGGCUGCAGUGUCGUGGUCACAGCUUGCUGCAGACUCGACCUCCCAGGCUUAAGCAAUCCUCCUGCUCGAGUG

>hsa-mir-1275 MI0006415

CCUCUGUGAGAAAGGGUGUGGGGGAGAGGCUGUCUUGUGUCUGUAAGUAUGCCAAACUUAUUUUCCCCAAGGCAGAGGGA

>hsa-mir-1276 MI0006416

CCCCAGCUAGGUAAAGAGCCCUGUGGAGACACCUGGAUUCAGAGAACAUGUCUCCACUGAGCACUUGGGCCUUGAUGGCGGCU

>hsa-mir-1277 MI0006419

ACCUCCCAAAUAUAUAUAUAUAUGUACGUAUGUGUAUAUAAAUGUAUACGUAGAUAUAUAUGUAUUUUUGGUGGGUUU

>hsa-mir-1278 MI0006425

AUUUGCUCAUAGAUGAUAUGCAUAGUACUCCCAGAACUCAUUAAGUUGGUAGUACUGUGCAUAUCAUCUAUGAGCGAAUAG

>hsa-mir-1279 MI0006426

AUAUUCACAAAAAUUCAUAUUGCUUCUUUCUAAUGCCAAGAAAGAAGAGUAUAAGAACUUCC

>hsa-mir-1282 MI0006429

CCUUCUUCUCGUUUGCCUUUUUCUGCUUCUGCUGCAUGAUCUCCGAGUCCCUGGGGGUAGAGAUGAUGGGGCACUGGGAGGUACCAGAGGGCAAAAAGGAC

>hsa-mir-1284 MI0006431

AUUUUGAUAUAUAAGCCAGUUUAAUGUUUUCUAUACAGACCCUGGCUUUUCUUAAAUUUUAUAUAUUGGAAAGCCCAUGUUUGUAUUGGAAACUGCUGGUUUCUUUCAUACUGAAAAUCU

>hsa-mir-1285-1 MI0006346

UGUAGAGAUAGGAUCUCACUUUGUUGCCCAGGCUGGUCUCAAACUCCUGGUCUGGGCAACAAAGUGAGACCUUAUCUCUACAAG

>hsa-mir-1285-2 MI0006347

UUUGGGAGGCCGAGGCUGGUGCAUCACUUGAGCCCAGCAAUUUGAGACCAAUCUGGGCAACAAAGUGAGACCUCCGUCUCUACAAAGA

>hsa-mir-1286 MI0006348

UGUCCUCUGGGGACUCAGCUUGCUCUGGCUGCUGGAUUGAAUUAGCUGCAGGACCAAGAUGAGCCCUUGGUGGAGACA

>hsa-mir-1287 MI0006349

GUUGUGCUGUCCAGGUGCUGGAUCAGUGGUUCGAGUCUGAGCCUUUAAAAGCCACUCUAGCCACAGAUGCAGUGAUUGGAGCCAUGACAA

>hsa-mir-1288 MI0006432

GAGGGUGUUGAUCAGCAGAUCAGGACUGUAACUCACCAUAGUGGUGGACUGCCCUGAUCUGGAGACCACUGCCUU

>hsa-mir-1289-1 MI0006350

UUCUCAAUUUUUAGUAGGAAUUAAAAACAAAACUGGUAAAUGCAGACUCUUGGUUUCCACCCCCAGAGAAUCCCUAAACCGGGGGUGGAGUCCAGGAAUCUGCAUUUUAGAAAGUACCCAGGGUGAUUCUGAUAAUUGGGAACA

>hsa-mir-1289-2 MI0006351

CCACGGUCCUAGUUAAAAAGGCACAUUCCUAGACCCUGCCUCAGAACUACUGAACAGAGUCACUGGGUGUGGAGUCCAGGAAUCUGCAUUUUUACCCCUAUCGCCCCCGCC

>hsa-mir-1290 MI0006352

GAGCGUCACGUUGACACUCAAAAAGUUUCAGAUUUUGGAACAUUUCGGAUUUUGGAUUUUUGGAUCAGGGAUGCUCAA

>hsa-mir-1291 MI0006353

GGUAGAAUUCCAGUGGCCCUGACUGAAGACCAGCAGUUGUACUGUGGCUGUUGGUUUCAAGCAGAGGCCUAAAGGACUGUCUUCCUG

>hsa-mir-1292 MI0006433

CCUGGGAACGGGUUCCGGCAGACGCUGAGGUUGCGUUGACGCUCGCGCCCCGGCUCCCGUUCCAGG

>hsa-mir-1293 MI0006355

AGGUUGUUCUGGGUGGUCUGGAGAUUUGUGCAGCUUGUACCUGCACAAAUCUCCGGACCACUUAGUCUUUA

>hsa-mir-1294 MI0006356

CACCUAAUGUGUGCCAAGAUCUGUUCAUUUAUGAUCUCACCGAGUCCUGUGAGGUUGGCAUUGUUGUCUGGCAUUGUCUGAUAUACAACAGUGCCAACCUCACAGGACUCAGUGAGGUGAAACUGAGGAUUAGGAAGGUGUA

>hsa-mir-1295a MI0006357

AGGACAUUUUGCCCAGAUCCGUGGCCUAUUCAGAAAUGUGGCCUGUGAUUAGGCCGCAGAUCUGGGUGAAAUGUCCUCC

>hsa-mir-1295b MI0019146

CACCCAGAUCUGCGGCCUAAUCACAGGCCACAUUUCUGAAUAGGCCACGGAUCUGGGCAA

>hsa-mir-1296 MI0003780

ACCUACCUAACUGGGUUAGGGCCCUGGCUCCAUCUCCUUUAGGAAAACCUUCUGUGGGGAGUGGGGCUUCGACCCUAACCCAGGUGGGCUGU

>hsa-mir-1297 MI0006358

UGUUUAUCUCUAGGGUUGAUCUAUUAGAAUUACUUAUCUGAGCCAAAGUAAUUCAAGUAAUUCAGGUGUAGUGAAAC

>hsa-mir-1298 MI0003938

AGACGAGGAGUUAAGAGUUCAUUCGGCUGUCCAGAUGUAUCCAAGUACCCUGUGUUAUUUGGCAAUAAAUACAUCUGGGCAACUGACUGAACUUUUCACUUUUCAUGACUCA

>hsa-mir-1299 MI0006359

CCUCAUGGCAGUGUUCUGGAAUCCUACGUGAGGGACAAUCAUUCAGACCCACGUAGCAGUGUUCUGGAAUUCUGUGUGAGGGA

>hsa-mir-1301 MI0003815

GGAUUGUGGGGGGUCGCUCUAGGCACCGCAGCACUGUGCUGGGGAUGUUGCAGCUGCCUGGGAGUGACUUCACACAGUCCUC

>hsa-mir-1302-1 MI0006362

CAGAAAGCCCAGUUAAAUUUGAAUUUCAAGUAAACAAUGAAUAAUUGUGUAUGUAAGAAUAUCCCAUACAAUAUUUGGGACAUACUUAUGCUAAAAAUUAUUCCUUGCUUAUCUGAAAUUCAAAUGUAACUAGGAUUCCUGUA

>hsa-mir-1302-10 MI0015979

GGAUGCCCAGCUAGUUUGAAUUUUAGAUAAACAACGAAUAAUUUCGUAGCAUAAAUAUGUCCCAAGCUUAGUUUGGGACAUACUUAUGCUAAAAAACAUUAUUGGUUGUUUAUCUGAGAUUCAGAAUUAAGCAUUUUA

>hsa-mir-1302-4 MI0006365

AAUGCAGAAGCACAGCUAAAAUUUGAAUUUCAGAUAAACAAAUUUUUCUUAGAAUAAGUAUGUCUCCAUGCAACAUUUGGGACAUACUUAUGCUAAAAUAUUAUUUGUGUUUCAUCUGAAAUUCAAAUUCAACUGGACAUCCUGUAUUUU

>hsa-mir-1302-5 MI0006366

UGCCCGGCCUCCCAUUAAAUUGGUUUUUCAGACAAAUCACAAAUUUGUUUAGGUAUAAGUAUAUCCCAUGUAAUCUUUGGGACAUACUUAUGCUAAAAUAAUUGUUCCUUGUUGAUUGGAAAUUUUAAUUUUAAUUAGGUGUCCUGUAUU

>hsa-mir-1302-6 MI0006367

AACAAAUAAUUUGGUAAUAUAUGUAUGGCCCACACAAUAUUUAGGACAACAAUAUUUGGGACAUACUUAUGCUAAAAAAGUAUUUGUUGA

>hsa-mir-1302-8 MI0006369

CCCAUUUAAACUUGAAUUUCAUAUAAACACCGUAAUUUUCAGCAUUAGUGUAUCACAUGCAGUAUUUGGGACAUACUUAUGCUAAAAAAUUAGGUGGUGUUGAUCUGAAAUUCCAGUGUAGAUGGGCA

>hsa-mir-1303 MI0006370

GGCUGGGCAACAUAGCGAGACCUCAACUCUACAAUUUUUUUUUUUUUAAAUUUUAGAGACGGGGUCUUGCUCUGUUGCCAGGCUUU

>hsa-mir-1304 MI0006371

AAACACUUGAGCCCAGCGGUUUGAGGCUACAGUGAGAUGUGAUCCUGCCACAUCUCACUGUAGCCUCGAACCCCUGGGCUCAAGUGAUUCA

>hsa-mir-1305 MI0006372

AAGAUCCUGCUGUUUCUACCAUUAGUUUUGAAUGUUUAUUGUAAAGAUACUUUUCAACUCUAAUGGGAGAGACAGCAGGAUUCUCC

>hsa-mir-1306 MI0006443

GUGAGCAGUCUCCACCACCUCCCCUGCAAACGUCCAGUGGUGCAGAGGUAAUGGACGUUGGCUCUGGUGGUGAUGGACAGUCCGA

>hsa-mir-1307 MI0006444

CAUCAAGACCCAGCUGAGUCACUGUCACUGCCUACCAAUCUCGACCGGACCUCGACCGGCUCGUCUGUGUUGCCAAUCGACUCGGCGUGGCGUCGGUCGUGGUAGAUAGGCGGUCAUGCAUACGAAUUUUCAGCUCUUGUUCUGGUGAC

>hsa-mir-1321 MI0006652

ACAUUAUGAAGCAAGUAUUAUUAUCCCUGUUUUACAAAUAAGGAAAUAAACUCAGGGAGGUGAAUGUGAUCAAAGAUAG

>hsa-mir-1322 MI0006653

AGUAUCAUGAAUUAGAAACCUACUUAUUACAUAGUUUACAUAAGAAGCGUGAUGAUGCUGCUGAUGCUGUA

>hsa-mir-1323 MI0003786

ACUGAGGUCCUCAAAACUGAGGGGCAUUUUCUGUGGUUUGAAAGGAAAGUGCACCCAGUUUUGGGGAUGUCAA

>hsa-mir-1324 MI0006657

CCUGAAGAGGUGCAUGAAGCCUGGUCCUGCCCUCACUGGGAACCCCCUUCCCUCUGGGUACCAGACAGAAUUCUAUGCACUUUCCUGGAGGCUCCA

>hsa-mir-1343 MI0017320

GCUGGCGUCGGUGCUGGGGAGCGGCCCCCGGGUGGGCCUCUGCUCUGGCCCCUCCUGGGGCCCGCACUCUCGCUCUGGGCCCGC

>hsa-mir-1468 MI0003782

GGUGGGUGGUUUCUCCGUUUGCCUGUUUCGCUGAUGUGCAUUCAACUCAUUCUCAGCAAAAUAAGCAAAUGGAAAAUUCGUCCAUC

>hsa-mir-1470 MI0007075

GCCCUCCGCCCGUGCACCCCGGGGCAGGAGACCCCGCGGGACGCGCCGAGGUAGGGGGGAC

>hsa-mir-1471 MI0007076

GCCCGCGUGUGGAGCCAGGUGUAGAGGCGGAGCACAGCUGGCUCUAAUUUGAGGGGC

>hsa-mir-1537 MI0007258

ACAGCUGUAAUUAGUCAGUUUUCUGUCCUGUCCACACAGAAAACCGUCUAGUUACAGUUGU

>hsa-mir-1538 MI0007259

GGGAACAGCAGCAACAUGGGCCUCGCUUCCUGCCGGCGCGGCCCGGGCUGCUGCUGUUCCU

>hsa-mir-1539 MI0007260

GGCUCUGCGGCCUGCAGGUAGCGCGAAAGUCCUGCGCGUCCCAGAUGCCC

>hsa-mir-1587 MI0016905

UUUGGGCUGGGCUGGGUUGGGCAGUUCUUCUGCUGGACUCACCUGUGACCAGC

>hsa-mir-1825 MI0008193

AGAGACUGGGGUGCUGGGCUCCCCUAGACUAGGACUCCAGUGCCCUCCUCUCC

>hsa-mir-1827 MI0008195

UCAGCAGCACAGCCUUCAGCCUAAAGCAAUGAGAAGCCUCUGAAAGGCUGAGGCAGUAGAUUGAAU

>hsa-mir-1908 MI0008329

CGGGAAUGCCGCGGCGGGGACGGCGAUUGGUCCGUAUGUGUGGUGCCACCGGCCGCCGGCUCCGCCCCGGCCCCCGCCCC

>hsa-mir-1909 MI0008330

CAUCCAGGACAAUGGUGAGUGCCGGUGCCUGCCCUGGGGCCGUCCCUGCGCAGGGGCCGGGUGCUCACCGCAUCUGCCCC

>hsa-mir-1910 MI0008331

UGUCCCUUCAGCCAGUCCUGUGCCUGCCGCCUUUGUGCUGUCCUUGGAGGGAGGCAGAAGCAGGAUGACAAUGAGGGCAA

>hsa-mir-1911 MI0008332

UCGGCAUCUGCUGAGUACCGCCAUGUCUGUUGGGCAUCCACAGUCUCCCACCAGGCAUUGUGGUCUCCGCUGACGCUUUG

>hsa-mir-1912 MI0008333

CUCUAGGAUGUGCUCAUUGCAUGGGCUGUGUAUAGUAUUAUUCAAUACCCAGAGCAUGCAGUGUGAACAUAAUAGAGAUU

>hsa-mir-1913 MI0008334

ACCUCUACCUCCCGGCAGAGGAGGCUGCAGAGGCUGGCUUUCCAAAACUCUGCCCCCUCCGCUGCUGCCAAGUGGCUGGU

>hsa-mir-1914 MI0008335

CGUGUGAGCCCGCCCUGUGCCCGGCCCACUUCUGCUUCCUCUUAGCGCAGGAGGGGUCCCGCACUGGGAGGGGCCCUCAC

>hsa-mir-1915 MI0008336

UGAGAGGCCGCACCUUGCCUUGCUGCCCGGGCCGUGCACCCGUGGGCCCCAGGGCGACGCGGCGGGGGCGGCCCUAGCGA

>hsa-mir-1972-1 MI0009982

UAUAGGCAUGUGCCACCACACCUGGCUUAAAUGUGUCAUUUAAAAAUUCAGGCCAGGCACAGUGGCUCAUGCCUGUA

>hsa-mir-1973 MI0009983

UAUGUUCAACGGCCAUGGUAUCCUGACCGUGCAAAGGUAGCAUA

>hsa-mir-1976 MI0009986

GCAGCAAGGAAGGCAGGGGUCCUAAGGUGUGUCCUCCUGCCCUCCUUGCUGU

>hsa-mir-2052 MI0010486

CUGUUUUGAUAACAGUAAUGUCCCUUUAGUUCAAAGUUACCAGCUAUCAAAACAA

>hsa-mir-2053 MI0010487

CUUGCCAUGUAAAUACAGAUUUAAUUAACAUUUGCAACCUGUGAAGAUGCAAAACUUUAAGUGUUAAUUAAACCUCUAUUUACAUAGCAAG

>hsa-mir-2054 MI0010488

CUGUAAUAUAAAUUUAAUUUAUUCUCUAUCAUUAAAAAAUGUAUUACAG

>hsa-mir-2110 MI0010629

CAGGGGUUUGGGGAAACGGCCGCUGAGUGAGGCGUCGGCUGUGUUUCUCACCGCGGUCUUUUCCUCCCACUCUUG

>hsa-mir-2113 MI0003939

UUUUCAAAGCAAUGUGUGACAGGUACAGGGACAAAUCCCGUUAAUAAGUAAGAGGAUUUGUGCUUGGCUCUGUCACAUGCCACUUUGAAAA

>hsa-mir-2114 MI0010633

CCUCCAUGCUCCUAGUCCCUUCCUUGAAGCGGUCGGAUAAUCACAUGACGAGCCUCAAGCAAGGGACUUCAAGCUGGUGG

>hsa-mir-2115 MI0010634

ACUGUCAUCCCACUGCUUCCAGCUUCCAUGACUCCUGAUGGAGGAAUCACAUGAAUUCAUCAGAAUUCAUGGAGGCUAGAAGCAGUAUGAGGAUCAUUUA

>hsa-mir-2116 MI0010635

GACCUAGGCUAGGGGUUCUUAGCAUAGGAGGUCUUCCCAUGCUAAGAAGUCCUCCCAUGCCAAGAACUCCCAGACUAGGA

>hsa-mir-2117 MI0010636

GCUCUGAUUUACUUCUGUCCGGCAUGGUGAACAGCAGGAUUGGCUGUAGCUGUUCUCUUUGCCAAGGACAGAUCUGAUCU

>hsa-mir-2276 MI0011282

GUGUUCUUCCAGUCCGCCCUCUGUCACCUUGCAGACGGCUUUCUCUCCGAAUGUCUGCAAGUGUCAGAGGCGAGGAGUGGCAGCUGCAU

>hsa-mir-2277 MI0011284

GUGCUUCCUGCGGGCUGAGCGCGGGCUGAGCGCUGCCAGUCAGCGCUCACAUUAAGGCUGACAGCGCCCUGCCUGGCUCGGCCGGCGAAGCUC

>hsa-mir-2278 MI0011285

GUGCUGCAGGUGUUGGAGAGCAGUGUGUGUUGCCUGGGGACUGUGUGGACUGGUAUCACCCAGACAGCUUGCACUGACUCCAGACCCUGCCGUCAU

>hsa-mir-2355 MI0015873

CAGACGUGUCAUCCCCAGAUACAAUGGACAAUAUGCUAUUAUAAUCGUAUGGCAUUGUCCUUGCUGUUUGGAGAUAAUACUGCUGAC

>hsa-mir-2392 MI0016870

AUGGUCCCUCCCAAUCCAGCCAUUCCUCAGACCAGGUGGCUCCCGAGCCACCCCAGGCUGUAGGAUGGGGGUGAGAGGUGCUAG

>hsa-mir-2467 MI0017432

GGACAGGCACCUGAGGCUCUGUUAGCCUUGGCUCUGGGUCCUGCUCCUUAGAGCAGAGGCAGAGAGGCUCAGGGUCUGUCU

>hsa-mir-2681 MI0012062

GCCCCCUUUUCACGCAUUUGUGUUUUACCACCUCCAGGAGACUGCCCAAAGACUCUUCAGUAUCAUGGAGUUGGUAAAGCACAGAUGCAUGAAUAAUUCAACGUG

>hsa-mir-2682 MI0012063

ACCUUCCUGAAAGAGGUUGGGGCAGGCAGUGACUGUUCAGACGUCCAAUCUCUUUGGGACGCCUCUUCAGCGCUGUCUUCCCUGCCUCUGCCUUUAGGACGAGUCUCAAA

>hsa-mir-2861 MI0013006

GGCGCCUCUGCAGCUCCGGCUCCCCCUGGCCUCUCGGGAACUACAAGUCCCAGGGGGCCUGGCGGUGGGCGGCGGGCGGAAGAGGCGGGG

>hsa-mir-2909 MI0013083

GGUGUUAGGGCCAACAUCUCUUGGUCUUUCCCCUGUGGUCCCAAGAUGGCUGUUGCAACUUAACGCCAU

>hsa-mir-3064 MI0017375

GGUCUGGCUGUUGUGGUGUGCAAAACUCCGUACAUUGCUAUUUUGCCACACUGCAACACCUUACAG

>hsa-mir-3065 MI0014228

CUGCCCUCUUCAACAAAAUCACUGAUGCUGGAGUCGCCUGAGUCAUCACUCAGCACCAGGAUAUUGUUGGAGAGGACAG

>hsa-mir-3074 MI0014181

GCUCGACUCCUGUUCCUGCUGAACUGAGCCAGUGUGUAAAAUGAGAACUGAUAUCAGCUCAGUAGGCACCGGAGGGCGGGU

>hsa-mir-3115 MI0014127

UCUGAAUAUGGGUUUACUAGUUGGUGGUGAAUUCAUGAGUCGCCAACUAUUAGGCCUUUAUGUCCAGA

>hsa-mir-3116-1 MI0014128

CUUUAUUGAGUCCCUACUAUGUUCCAGGCACUGGGUAUCGUAGGUGCCUGGAACAUAGUAGGGACUCAAUAAAG

>hsa-mir-3117 MI0014130

CCCUAAAGGGCCAGACACUAUACGAGUCAUAUAAGGGAAGGCAUUAUAGGACUCAUAUAGUGCCAGGUGUUUUGUGGG

>hsa-mir-3118-5 MI0014243

CACACAUACAAUAAUAUUCAUAAUGCAAUCACACACAAUCACCAUGUGACUGCAUUAUGAAAAUUCUUCUAGUGUG

>hsa-mir-3119-1 MI0014134

AUUAACUCUGGCUUUUAACUUUGAUGGCAAAGGGGUAGCUAAACAAUCUAUGUCUUUGCCAUCAAAGUUAAAAGCCAUAGUUAAU

>hsa-mir-3120 MI0014136

GUCAUGUGACUGCCUGUCUGUGCCUGCUGUACAGGUGAGCGGAUGUUCUGCACAGCAAGUGUAGACAGGCAGACACAUGAC

>hsa-mir-3121 MI0014137

AAAUGGUUAUGUCCUUUGCCUAUUCUAUUUAAGACACCCUGUACCUUAAAUAGAGUAGGCAAAGGACAGAAACAUUU

>hsa-mir-3122 MI0014138

ACCAGCUCUGUUGGGACAAGAGGACGGUCUUCUUUUGGAAGGAAGACCAUCAUCUUGUCCGAAGAGAGCUGGU

>hsa-mir-3123 MI0014139

AUGGAUUUGAUUGAAUGAUUCUCCCAUUUCCACAUGGAGAGUGGAGCCCAGAGAAUUGUUUAAUCAUGUAUCCAU

>hsa-mir-3124 MI0014140

GCGGGCUUCGCGGGCGAAGGCAAAGUCGAUUUCCAAAAGUGACUUUCCUCACUCCCGUGAAGUCGGC

>hsa-mir-3125 MI0014142

GAGAAUGGGUAGAGGAAGCUGUGGAGAGAACUCACGGUGCCUGUGGUUCGAGAUCCCCGCCUUCCUCCUCCUUUCCUC

>hsa-mir-3126 MI0014143

AUGAUUAUAUGAGGGACAGAUGCCAGAAGCACUGGUUAUGAUUUGCAUCUGGCAUCCGUCACACAGAUAAUUAU

>hsa-mir-3127 MI0014144

GGCCAGGCCCAUCAGGGCUUGUGGAAUGGGAAGGAGAAGGGACGCUUCCCCUUCUGCAGGCCUGCUGGGUGUGGCU

>hsa-mir-3128 MI0014145

UUCCUCUGGCAAGUAAAAAACUCUCAUUUUCCUUAAAAAAUGAGAGUUUUUUACUUGCAAUAGGAA

>hsa-mir-3129 MI0014146

GUACUUGGGCAGUAGUGUAGAGAUUGGUUUGCCUGUUAAUGAAUUCAAACUAAUCUCUACACUGCUGCCCAAGAGC

>hsa-mir-3130-1 MI0014147

CUUGUCAUGUCUUACCCAGUCUCCGGUGCAGCCUGUUGUCAAGGCUGCACCGGAGACUGGGUAAGACAUGACAAG

>hsa-mir-3131 MI0014151

GAGUCGAGGACUGGUGGAAGGGCCUUUCCCCUCAGACCAAGGCCCUGGCCCCAGCUUCUUCUC

>hsa-mir-3132 MI0014152

GGUGGGAUGGGUAGAGAAGGAGCUCAGAGGACGGUGCGCCUUGUUUCCCUUGAGCCCUCCCUCUCUCAUCCCACC

>hsa-mir-3133 MI0014153

CAGAAAUUGUAAAGAACUCUUAAAACCCAAUAGUAAAAAGACAACCUGUUGAGUUUUAAGAGUUCUUUAUAUAUUCUG

>hsa-mir-3134 MI0014155

UGUAUCCAAUGUGUAGUCUUUUAUCCCUCACAUGGAGUAAAAUAUGAUGGAUAAAAGACUACAUAUUGGGUACA

>hsa-mir-3135a MI0014156

UCACUUUGGUGCCUAGGCUGAGACUGCAGUGGUGCAAUCUCAGUUCACUGCAGCCUUGACCUCCUGGGCUCAGGUGA

>hsa-mir-3135b MI0016809

UGCCCAGGCUGGAGCGAGUGCAGUGGUGCAGUCAGUCCUAGCUCACUGCAGCCUCGAACUCCUGGGCU

>hsa-mir-3136 MI0014158

AAUAUGAAACUGACUGAAUAGGUAGGGUCAUUUUUCUGUGACUGCACAUGGCCCAACCUAUUCAGUUAGUUCCAUAUU

>hsa-mir-3137 MI0014160

UACAGGUCUGUAGCCUGGGAGCAAUGGGGUGUAUGGUAUAGGGGUAGCCUCGUGCUCCUGGGCUACAAACCUGUA

>hsa-mir-3138 MI0014161

CCCUCCUCGGCACUUCCCCCACCUCACUGCCCGGGUGCCCACAAGACUGUGGACAGUGAGGUAGAGGGAGUGCCGAGGAGGG

>hsa-mir-3139 MI0014162

GGCUCAGAGUAGGAGCUCAACAGAUGCCUGUUGACUGAAUAAUAAACAGGUAUCGCAGGAGCUUUUGUUAUGUGCC

>hsa-mir-3140 MI0014163

CCUCUUGAGGUACCUGAAUUACCAAAAGCUUUAUGUAUUCUGAAGUUAUUGAAAAUAAGAGCUUUUGGGAAUUCAGGUAGUUCAGGAGUG

>hsa-mir-3141 MI0014165

UCACCCGGUGAGGGCGGGUGGAGGAGGAGGGUCCCCACCAUCAGCCUUCACUGGGACGGGA

>hsa-mir-3142 MI0014166

UUCAGAAAGGCCUUUCUGAACCUUCAGAAAGGCUGCUGAAUCUUCAGAAAGGCCUUUCUGAACCUUCAGAAAGGCUGCUGAA

>hsa-mir-3143 MI0014167

UAGAUAACAUUGUAAAGCGCUUCUUUCGCGGUUGGGCUGGAGCAACUCUUUACAAUGUUUCUA

>hsa-mir-3144 MI0014169

AACUACACUUUAAGGGGACCAAAGAGAUAUAUAGAUAUCAGCUACCUAUAUACCUGUUCGGUCUCUUUAAAGUGUAGUU

>hsa-mir-3145 MI0014170

UAUAUGAGUUCAACUCCAAACACUCAAAACUCAUUGUUGAAUGGAAUGAGAUAUUUUGAGUGUUUGGAAUUGAACUCGUAUA

>hsa-mir-3146 MI0014172

GCUAAGUCCCUUCUUUCUAUCCUAGUAUAACUUGAAGAAUUCAAAUAGUCAUGCUAGGAUAGAAAGAAUGGGACUUGGC

>hsa-mir-3147 MI0014173

GUCCGGGUUGGGCAGUGAGGAGGGUGUGACGCCGCGAAGUGCACCUCGCCCUUGUCCAACUCGGAC

>hsa-mir-3148 MI0014175

GAGUUAAGAUGGAAAAAACUGGUGUGUGCUUAUUGAUGUAGCCAACAAGCAUACAUCAGUUUUUUCCAACUUAACUC

>hsa-mir-3149 MI0014176

AUACAUACAUGUACACACACAUGUCAUCCACACACAUACAUAUAUAUAUGUUUGUAUGGAUAUGUGUGUGUAUGUGUGUGUAU

>hsa-mir-3150b MI0016426

GAGGGAAAGCAGGCCAACCUCGAGGAUCUCCCCAGCCUUGGCGUUCAGGUGCUGAGGAGAUCGUCGAGGUUGGCCUGCUUCCCCUC

>hsa-mir-3151 MI0014178

GGGGUGAUGGGUGGGGCAAUGGGAUCAGGUGCCUCAAAGGGCAUCCCACCUGAUCCCACAGCCCACCUGUCACCCC

>hsa-mir-3152 MI0014179

GUGCAGAGUUAUUGCCUCUGUUCUAACACAAGACUAGGCUUCCCUGUGUUAGAAUAGGGGCAAUAACUCUGCAC

>hsa-mir-3153 MI0014180

GACAAAUUUUAAAUGUCCCUGUCCCCUUCCCCCCAAUUAAAGUAGAUUGGGGGAAAGCGAGUAGGGACAUUUAAAAUUUGUU

>hsa-mir-3154 MI0014182

GGCCCCUCCUUCUCAGCCCCAGCUCCCGCUCACCCCUGCCACGUCAAAGGAGGCAGAAGGGGAGUUGGGAGCAGAGAGGGGACC

>hsa-mir-3155a MI0014183

UCCGGGCAUCACCUCCCACUGCAGAGCCUGGGGAGCCGGACAGCUCCCUUCCCAGGCUCUGCAGUGGGAACUGAUGCCUGGA

>hsa-mir-3156-2 MI0014230

UGCAGAAGAAAGAUCUGGAAGUGGGAGACACUUUCACUAUAUAUAGUGGCUCCCACUUCCAGAUCUUUCUCUCUGUA

>hsa-mir-3157 MI0014185

GGGAAGGGCUUCAGCCAGGCUAGUGCAGUCUGCUUUGUGCCAACACUGGGGUGAUGACUGCCCUAGUCUAGCUGAAGCUUUUCCC

>hsa-mir-3158-1 MI0014186

AUUCAGGCCGGUCCUGCAGAGAGGAAGCCCUUCUGCUUACAGGUAUUGGAAGGGCUUCCUCUCUGCAGGACCGGCCUGAAU

>hsa-mir-3159 MI0014188

CCAAAGUCCUAGGAUUACAAGUGUCGGCCACGGGCUGGGCACAGUGGCUCACGCCUGUAAUCCCAGCAUUUUGG

>hsa-mir-3160-1 MI0014189

GGACCUGCCCUGGGCUUUCUAGUCUCAGCUCUCCUCCAGCUCAGCUGGUCAGGAGAGCUGAGACUAGAAAGCCCAGGGCAGGUUC

>hsa-mir-3161 MI0014191

CCUCGAGAGCUGAUAAGAACAGAGGCCCAGAUUGAAGUUGAAUAGUGCUGGGCCUUUGUUUUUACCAAGUUCCCUGG

>hsa-mir-3162 MI0014192

CUGACUUUUUUAGGGAGUAGAAGGGUGGGGAGCAUGAACAAUGUUUCUCACUCCCUACCCCUCCACUCCCCAAAAAAGUCAG

>hsa-mir-3163 MI0014193

UUCCUCAUCUAUAAAAUGAGGGCAGUAAGACCUUCCUUCCUUGUCUUACUACCCCCAUUUUAUAGAUGAGGAA

>hsa-mir-3164 MI0014194

CUUGGAAACUGUGACUUUAAGGGAAAUGGCGCACAGCAGACCCUGCAAUCAUGCCGUUUUGCUUGAAGUCGCAGUUUCCCAGG

>hsa-mir-3165 MI0014195

CCGGUGGCAAGGUGGAUGCAAUGUGACCUCAACUCUUGGUCCUCUGAGGUCACAUUGUAUCCACCUUACCACUGG

>hsa-mir-3166 MI0014196

AAAUUUUUUUGAGGCCAGUAGGCAUUGUCUGCGUUAGGAUUUCUGUAUCAUCCUCCUAACGCAGACAAUGCCUACUGGCCUAAGAAAAAUUU

>hsa-mir-3167 MI0014198

GGCUGUGGAGGCACCAGUAUUUCUGAAAUUCUUUUUUCUGAAAUUCUUCAGGAAGGAUUUCAGAAAUACUGGUGUCCCGACAGCC

>hsa-mir-3168 MI0014199

AAGAUCAUGAGUUCUACAGUCAGACAGCCUGAGUUGGAGGCUCAUCUUCACUUCUUGCUGUGUGACCCUGGGCCAGUGACUU

>hsa-mir-3169 MI0014200

AUGUGAAAACAUAGGACUGUGCUUGGCACAUAGCACAAAGUCUUAUGGUACUGUGUGCCAAGCAUAGUCCUGUGUUUUUACAU

>hsa-mir-3170 MI0014201

CUGGUAACACUGGGGUUCUGAGACAGACAGUGUUAGCUCCAGAAGCAUUGCCUGUCUUAGAACCCCUAUGUUACCAG

>hsa-mir-3171 MI0014202

UAUAUAUAGAGAUGUAUGGAAUCUGUAUAUAUCUAUAUAUAUGUGUAUAUAUAGAUUCCAUAAAUCUAUAUAUG

>hsa-mir-3173 MI0014204

UCCCUGCCCUGCCUGUUUUCUCCUUUGUGAUUUUAUGAGAACAAAGGAGGAAAUAGGCAGGCCAGGGA

>hsa-mir-3174 MI0014208

GUUACCUGGUAGUGAGUUAGAGAUGCAGAGCCCUGGGCUCCUCAGCAAACCUACUGGAUCUGCAUUUUAAUUCACAUGCAUGGUAAU

>hsa-mir-3175 MI0014209

CCUGGGGGGCGGGGAGAGAACGCAGUGACGUCUGGCCGCGUGCGCAUGUCGGGCGCUUUCUCCUCCCCCUACCCAGG

>hsa-mir-3176 MI0014210

UGGCCUCUCCAGUCUGCAGCUCCCGGCAGCCUCGGGCCACACUCCCGGGAUCCCCAGGGACUGGCCUGGGACUACCGGGGGUGGCGGCCG

>hsa-mir-3177 MI0014211

CCACGUGCCAUGUGUACACACGUGCCAGGCGCUGUCUUGAGACAUUCGCGCAGUGCACGGCACUGGGGACACGUGGCACUGG

>hsa-mir-3178 MI0014212

GAGGCUGGGCGGGGCGCGGCCGGAUCGGUCGAGAGCGUCCUGGCUGAUGACGGUCUCCCGUGCCCACGCCCCAAACGCAGUCUC

>hsa-mir-3179-1 MI0014213

CAGGAUCACAGACGUUUAAAUUACACUCCUUCUGCUGUGCCUUACAGCAGUAGAAGGGGUGAAAUUUAAACGUCUGUGAUCCUG

>hsa-mir-3180-1 MI0014214

CAGUGCGACGGGCGGAGCUUCCAGACGCUCCGCCCCACGUCGCAUGCGCCCCGGGAAAGCGUGGGGCGGAGCUUCCGGAGGCCCCGCCCUGCUG

>hsa-mir-3180-4 MI0016408

GCUCCGCCCCACGUCGCAUGCGCCCCGGGAACGCGUGGGGCGGAGCUUCCGGAGGCCCCGCUCUGCUGCCGACCCUGUGGAGCGGAGGGUGAAGCCUCCGGAUGCCAGUCCCUCAUCGCUGGCCUGGUCGCGCUGUGGCGAAGGGGGCGGAGC

>hsa-mir-3181 MI0014223

CGGCGACCAUCGGGCCCUCGGCGCCGGCCCGUUAGUUGCCCGGGCCCGAGCCGGCCGGGCCCGCGGGUUGCCG

>hsa-mir-3182 MI0014224

GCUGCUUCUGUAGUGUAGUCCGUGCAUCCGCCCUUCGAUGCUUGGGUUGGAUCAUAGAGCAGU

>hsa-mir-3183 MI0014225

CUCUGCCCUGCCUCUCUCGGAGUCGCUCGGAGCAGUCACGUUGACGGAAUCCUCCGGCGCCUCCUCGAGGGAGGAGAGGCAGGG

>hsa-mir-3184 MI0014226

AAGCAAGACUGAGGGGCCUCAGACCGAGCUUUUGGAAAAUAGAAAAGUCUCGCUCUCUGCCCCUCAGCCUAACUU

>hsa-mir-3185 MI0014227

GAAUGGAAGAAGAAGGCGGUCGGUCUGCGGGAGCCAGGCCGCAGAGCCAUCCGCCUUCUGUCCAUGUC

>hsa-mir-3186 MI0014229

AGCCUGCGGUUCCAACAGGCGUCUGUCUACGUGGCUUCAACCAAGUUCAAAGUCACGCGGAGAGAUGGCUUUGGAACCAGGGGCU

>hsa-mir-3187 MI0014231

GCUGGCCCUGGGCAGCGUGUGGCUGAAGGUCACCAUGUUCUCCUUGGCCAUGGGGCUGCGCGGGGCCAGC

>hsa-mir-3188 MI0014232

GGCGCCUCCUGCUCUGCUGUGCCGCCAGGGCCUCCCCUAGCGCGCCUUCUGGAGAGGCUUUGUGCGGAUACGGGGCUGGAGGCCU

>hsa-mir-3189 MI0014233

GCCUCAGUUGCCCCAUCUGUGCCCUGGGUAGGAAUAUCCUGGAUCCCCUUGGGUCUGAUGGGGUAGCCGAUGC

>hsa-mir-3190 MI0014235

CUGGGGUCACCUGUCUGGCCAGCUACGUCCCCACGGCCCUUGUCAGUGUGGAAGGUAGACGGCCAGAGAGGUGACCCCGG

>hsa-mir-3191 MI0014236

GGGGUCACCUCUCUGGCCGUCUACCUUCCACACUGACAAGGGCCGUGGGGACGUAGCUGGCCAGACAGGUGACCCC

>hsa-mir-3192 MI0014237

GGAAGGGAUUCUGGGAGGUUGUAGCAGUGGAAAAAGUUCUUUUCUUCCUCUGAUCGCCCUCUCAGCUCUUUCCUUCU

>hsa-mir-3193 MI0014238

UCCUGCGUAGGAUCUGAGGAGUGGACGAGUCUCAUUACCCAGCUCCUGAGCAGGA

>hsa-mir-3194 MI0014239

AGGUGGCAGGGCCAGCCACCAGGAGGGCUGCGUGCCACCCGGGCAGCUCUGCUGCUCACUGGCAGUGUCACCU

>hsa-mir-3195 MI0014240

CCGCAGCCGCCGCGCCGGGCCCGGGUUGGCCGCUGACCCCCGCGGGGCCCCCGGCGGCCGGGGCGGGGGCGGGGGCUGCCCCGG

>hsa-mir-3196 MI0014241

GGGUGGGGGCGGGGCGGCAGGGGCCUCCCCCAGUGCCAGGCCCCAUUCUGCUUCUCUCCCAGCU

>hsa-mir-3197 MI0014245

GGCGAGGGGAGGCGCAGGCUCGGAAAGGCGCGCGAGGCUCCAGGCUCCUUCCCGAUCCACCGCUCUCCUCGCU

>hsa-mir-3198-1 MI0014246

GACUGUGCUCUCACUGUUCACCCAGCACUAGCAGUACCAGACGGUUCUGUGGAGUCCUGGGGAAUGGAGAGAGCACAGUC

>hsa-mir-3199-1 MI0014247

GGUGACUCCAGGGACUGCCUUAGGAGAAAGUUUCUGGAAGUUCUGACAUUCCAGAAACUUUCUCCUAAGGCAGUCCCUGGGAGUCACU

>hsa-mir-3200 MI0014249

GGUGGUCGAGGGAAUCUGAGAAGGCGCACAAGGUUUGUGUCCAAUACAGUCCACACCUUGCGCUACUCAGGUCUGCUCGUGCCCU

>hsa-mir-3201 MI0014250

GGGAUAUGAAGAAAAAUAAGAGGCUAGGAUUGCCUCUUAUUUUUACAUGCCC

>hsa-mir-3202-1 MI0014252

UAUUAAUAUGGAAGGGAGAAGAGCUUUAAUGAUUGGAGUCAUUUUCAGAGCAUUAAAGCUCUUCUCCCUUCCAUAUUAAUG

>hsa-mir-3529 MI0017351

GGCACCAUUAGGUAGACUGGGAUUUGUUGUUGAGCGCAGUAAGACAACAACAAAAUCACUAGUCUUCCAGAUGGGGCC

>hsa-mir-3591 MI0017383

CAGUAGCUAUUUAGUGUGAUAAUGGCGUUUGAUAGUUUAGACACAAACACCAUUGUCACACUCCACAGCUCUG

>hsa-mir-3605 MI0015995

ACUUUAUACGUGUAAUUGUGAUGAGGAUGGAUAGCAAGGAAGCCGCUCCCACCUGACCCUCACGGCCUCCGUGUUACCUGUCCUCUAGGUGGGACGCUCG

>hsa-mir-3606 MI0015996

UUGUUGCUAUCUAGGUUAGUGAAGGCUAUUUUAAUUUUUUUAAAAUUUCUUUCACUACUUAGG

>hsa-mir-3607 MI0015997

AAGGUUGCGGUGCAUGUGAUGAAGCAAAUCAGUAUGAAUGAAUUCAUGAUACUGUAAACGCUUUCUGAUGUACUACUCA

>hsa-mir-3609 MI0015999

GUAACAGUAACUUUUAUUCUCAUUUUCCUUUUCUCUACCUUGUAGAGAAGCAAAGUGAUGAGUAAUACUGGCUGGAGCCC

>hsa-mir-3610 MI0016000

AAGAGCCGCGGCGUAACGGCAGCCAUCUUGUUUGUUUGAGUGAAUCGGAAAGGAGGCGCCGGCUGUGGCGGCG

>hsa-mir-3611 MI0016001

AGCAGGUCUAAUAAGAAUUUCUUUUUCUUCACAAUUAUGAAAGAAAAGAAAUUGUGAAGAAAGAAAUUCUUACUAGUUUUGCU

>hsa-mir-3612 MI0016002

GGGACUGGGGAUGAGGAGGCAUCUUGAGAAAUGGAAGGAAUGGGAUCUACUUCCAGUUCACUAGAGGCGUCCUGACACCCCUAGCUC

>hsa-mir-3613 MI0016003

UGGUUGGGUUUGGAUUGUUGUACUUUUUUUUUUGUUCGUUGCAUUUUUAGGAACAAAAAAAAAAGCCCAACCCUUCACACCACUUCA

>hsa-mir-3614 MI0016004

GGUUCUGUCUUGGGCCACUUGGAUCUGAAGGCUGCCCCUUUGCUCUCUGGGGUAGCCUUCAGAUCUUGGUGUUUUGAAUUCUUACU

>hsa-mir-3615 MI0016005

GACUCUGGGACGCUCAGACGCCGCGCGGGGCGGGGAUUGGUCUGUGGUCCUCUCUCGGCUCCUCGCGGCUCGCGGCGGCCGACGGUU

>hsa-mir-3616 MI0016006

UGUCACUCCGCCAGCAUCAUGAAGUGCACUCAUGAUAUGUUUGCCCCAUCAGCGUGUCACGAGGGCAUUUCAUGAUGCAGGCGGGGUUGGCA

>hsa-mir-3617 MI0016007

AGGUCAUAGAAAGACAUAGUUGCAAGAUGGGAUUAGAAACCAUAUGUCUCAUCAGCACCCUAUGUCCUUUCUCUGCCCU

>hsa-mir-3618 MI0016008

UAAGCUGAGUGCAUUGUGAUUUCCAAUAAUUGAGGCAGUGGUUCUAAAAGCUGUCUACAUUAAUGAAAAGAGCAAUGUGGCCAGCUUG

>hsa-mir-3619 MI0016009

ACGGCAUCUUUGCACUCAGCAGGCAGGCUGGUGCAGCCCGUGGUGGGGGACCAUCCUGCCUGCUGUGGGGUAAGGACGGCUGU

>hsa-mir-3620 MI0016011

GUGAGGUGGGGGCCAGCAGGGAGUGGGCUGGGCUGGGCUGGGCCAAGGUACAAGGCCUCACCCUGCAUCCCGCACCCAG

>hsa-mir-3621 MI0016012

GUGAGCUGCUGGGGACGCGGGUCGGGGUCUGCAGGGCGGUGCGGCAGCCGCCACCUGACGCCGCGCCUUUGUCUGUGUCCCACAG

>hsa-mir-3622b MI0016014

AGUGAUAUAAUAGAGGGUGCACAGGCAUGGGAGGUCAGGUGAGCUCAGCUCCCUGCCUCACCUGAGCUCCCGUGCCUGUGCACCCUCUAUUGGCU

>hsa-mir-3646 MI0016046

UUCAGUAGGUUGGGUUCAUUUCAUUUUCAUGACAACCCUAUAUGGGAAAAUGUUGUGAAAAUGAAAUGAGCCCAGCCCAUUGAA

>hsa-mir-3648 MI0016048

CGCGACUGCGGCGGCGGUGGUGGGGGGAGCCGCGGGGAUCGCCGAGGGCCGGUCGGCCGCCCCGGGUGCCGCGCGGUGCCGCCGGCGGCGGUGAGGCCCCGCGCGUGUGUCCCGGCUGCGGUCGGCCGCGCUCGAGGGGUCCCCGUGGCGUCCCCUUCCCCGCCGGCCGCCUUUCUCGCG

>hsa-mir-3649 MI0016049

GCUUGGAACAGGCACCUGUGUGUGCCCAAGUGUUUCUAGCAAACACAGGGACCUGAGUGUCUAAGC

>hsa-mir-3650 MI0016050

UCAAGGUGUGUCUGUAGAGUCCUGACUGCGUGCCAGGGGCUCUGUCUGGCACAUUUCUGA

>hsa-mir-3651 MI0016051

GAUUCGAUGGGCCAUAGCAAUCCUGUGAUUUAUGCAUGGAGGCUGCUUCUCCUCAGCAGCUGCCAUAGCCCGGUCGCUGGUACAUGAUUC

>hsa-mir-3652 MI0016052

CGGCUGGAGGUGUGAGGAUCCGAACCCAGGGGUGGGGGGUGGAGGCGGCUCCUGCGAUCGAAGGGGACUUGAGACUCACCGGCCGCACGCCAUGAGGGCCCUGUGGGUGCUGGGCCUCUGCUGCGUCCUGC

>hsa-mir-3653 MI0016053

UCCCUGGGGACCCCUGGCAGCCCCUCCUGAUGAUUCUUCUUCCUGAGCACGCUCAUGAUGAGCAAACUGAGCCUCUAAGAAGUUGACUGAAGGGGCUGCUUCCCCAAGGA

>hsa-mir-3654 MI0016054

UUCAUGAGCUGCAAUCUCAUCACUGGAAUGUUCCAGCGACUGGACAAGCUGAGGAA

>hsa-mir-3655 MI0016055

GCUUGUCGCUGCGGUGUUGCUGUUGGAGACUCGAUUGUUGGUGACAGCGAAAGAACGAUAACAAAAUGCCGGAGCGAGAUAGU

>hsa-mir-3656 MI0016056

CUUUCGGCCAGCGGGACGGCAUCCGAGGUGGGCUAGGCUCGGGCCCGUGGCGGGUGCGGGGGUGGGAGG

>hsa-mir-3657 MI0016057

UGUGUCCCAUAAUUAAAUAAUGAAAUCUGAAAUCACCAAUAAUGGGACACUAAUGUGAUUAAUGUUGUUGUGUCCCAUUAUUGGUGAUUUCAGAUUUCAUAUAUGAUUAAGGACAUA

>hsa-mir-3658 MI0016058

UAUUUAAGAAAACACCAUGGAGAUGAAAUGCCUUUGAUUUUUUUUUUCUUUUUGUA

>hsa-mir-3659 MI0016060

UCUACAAGCAGAUACAAGGAUGCCCUUGUACACAACACACGUGCUGCUUGUAUAGACAUGAGUGUUGUCUACGAGGGCAUCCUUGUGUCUGUGUGUGUG

>hsa-mir-3660 MI0016061

GAAAGAAGAACUGGACAAAAUUAAAAUGCUCUUCUGUCAUUGUAAUAGUUCAUAUGGGCACUGACAGGAGAGCAUUUUGACUUUGUCAAGUGUGUCUGCU

>hsa-mir-3661 MI0016062

CACCUUCUCGCAGAGGCUCUUGACCUGGGACUCGGACAGCUGCUUGCACUCGUUCAGCUGCUCGAUCCACUGGUCCAGCUCCUUGGUGAACACCUU

>hsa-mir-3662 MI0016063

UGUGUUUUCCUCAACGCUCACAGUUACACUUCUUACUCUCAAUCCAUUCAUAUUGAAAAUGAUGAGUAGUGACUGAUGAAGCACAAAUCAGCCAA

>hsa-mir-3663 MI0016064

CCCGGGACCUUGGUCCAGGCGCUGGUCUGCGUGGUGCUCGGGUGGAUAAGUCUGAUCUGAGCACCACACAGGCCGGGCGCCGGGACCAAGGGGGCUC

>hsa-mir-3664 MI0016065

CUGUAAACUUGAAGGUAGGGAACUCUGUCUUCACUCAUGAGUACCUUCCAACACGAGCUCUCAGGAGUAAAGACAGAGUUCCCUACCUUCAAUGUGGAU

>hsa-mir-3665 MI0016066

GCGGGCGGCGGCGGCGGCAGCAGCAGCAGGUGCGGGGCGGCGGCCGCGCUGGCCGCUCGACUCCGCAGCUGCUCGUUCUGCUUCUCCAGCUUGCGCACCAGCUCC

>hsa-mir-3666 MI0016067

AGUAAGGUCCGUCAGUUGUAAUGAGACCCAGUGCAAGUGUAGAUGCCGACUCCGUGGCAGAGUUCAGCGUUUCACACUGCCUGGUCUCUGUCACUCUAUUGAAUUAGAUUG

>hsa-mir-3667 MI0016068

UGAGGAUGAAAGACCCAUUGAGGAGAAGGUUCUGCUGGCUGAGAACCUUCCUCUCCAUGGGUCUUUCAUCCUCA

>hsa-mir-3668 MI0016069

AUAUAUGAAAUGUAGAGAUUGAUCAAAAUAGUUUCUAUCAAAAUAGUUUUGAUCAAUCUCUGCAAUUUUAUAUAU

>hsa-mir-3670-1 MI0016071

UCUAGACUGGUAUAGCUGCUUUUGGAGCCUCACCUGCUGAGAGCUCACAGCUGUCCUUCUCUAGA

>hsa-mir-3671 MI0016072

AUGUUAUUGCUGCUGCUGUCACAUUUACAUGAAAAUAAAAUGUAAAUUAUUUUAUUUCUAUCAAAUAAGGACUAGUCUGCAGUGAUAU

>hsa-mir-3672 MI0016073

UCUUUGUGAUUACCAUGAGACUCAUGUAAAACAUCUUAGACUAUUACAAGAUGUUUUAUGAGUCUCAUGAUAAUCACAAAGA

>hsa-mir-3673 MI0016074

AUAUAUAUAUAUGGAAUGUAUAUACGGAAUAUAUAUAUAUAUGGAAUGUAUAUACGGAAUAUAUAUAUAUAUGGAAUGUAUAUACGGAAUAUAUAUAUAUAU

>hsa-mir-3674 MI0016075

ACAUCACUAUUGUAGAACCUAAGAUUGGCCGUUUGAGAUGUCCUUUCAAGUUUUUGCAUUUCUGAUGU

>hsa-mir-3675 MI0016076

GGAUGAUAAGUUAUGGGGCUUCUGUAGAGAUUUCUAUGAGAACAUCUCUAAGGAACUCCCCCAAACUGAAUUC

>hsa-mir-3677 MI0016078

GGCAGUGGCCAGAGCCCUGCAGUGCUGGGCAUGGGCUUCUCGUGGGCUCUGGCCACGGCC

>hsa-mir-3678 MI0016079

GAAUCCGGUCCGUACAAACUCUGCUGUGUUGAAUGAUUGGUGAGUUUGUUUGCUCAUUGAUUGAAUCACUGCAGAGUUUGUACGGACCGGAUUC

>hsa-mir-3679 MI0016080

CGUGGUGAGGAUAUGGCAGGGAAGGGGAGUUUCCCUCUAUUCCCUUCCCCCCAGUAAUCUUCAUCAUG

>hsa-mir-3680-1 MI0016081

AAAUUUAAGGAGGGACUCACUCACAGGAUUGUGCAAAUGCAAAGUUGGCUUUUGCAUGACCCUGGGAGUAGGUGCCUCCUUAAAUUU

>hsa-mir-3681 MI0016082

ACUUCCAGUAGUGGAUGAUGCACUCUGUGCAGGGCCAACUGUGCACACAGUGCUUCAUCCACUACUGGAAGU

>hsa-mir-3682 MI0016083

UAAGUUAUAUAUGUCUACUUCUACCUGUGUUAUCAUAAUAAAGGUGUCAUGAUGAUACAGGUGGAGGUAGAAAUAUAUAACUUA

>hsa-mir-3683 MI0016084

GGGUGUACACCCCCUGCGACAUUGGAAGUAGUAUCAUCUCUCCCUUGGAUGCUACGAACAAUAUCACAGAAGGUGUACACCC

>hsa-mir-3684 MI0016085

AAUCUAAAGGACCUGUACUAGGUUUAACAUGUUGAGCAUUACUCAUGUUAGACCUAGUACACGUCCUUUAGAUU

>hsa-mir-3685 MI0016086

GUACAUUUCCUACCCUACCUGAAGACUUGAGAUUAUAGUCUUUGGGGGGAUGGGCAAAGUAC

>hsa-mir-3686 MI0016087

CUCACCUCAUUCAUUUACCUUCUCUUACAGAUCACUUUUCUGCACUGGACAGUGAUCUGUAAGAGAAAGUAAAUGAAAGAGGUGAG

>hsa-mir-3687 MI0016088

CGCGCGUGCGCCCGAGCGCGGCCCGGUGGUCCCUCCCGGACAGGCGUUCGUGCGACGUGUG

>hsa-mir-3688-1 MI0016089

UCUUCACUUUCAAGAGUGGCAAAGUCUUUCCAUAUGUAUGUAUGUAUGUCUGUUACACAUAUGGAAAGACUUUGCCACUCUUUAAAGUGAAGA

>hsa-mir-3689b MI0016411

GAUCCUGUGCUCCCUGGGGGGUCUGAUCCUGUGCUUCCUGGGAGGUGUGAUAUCAUGGUUCCUGGGAGGUGUGAUCCCGUGCUUCCUGGGAGGUGUGAUAUUGUGGUUCCUGGGAGGUGUGAUCCCGUGCUCCCUGGGAGGUGUGAUC

>hsa-mir-3689d-2 MI0016835

ACUGGGAGGUGUGAUCUCACACUCGCUGGGAGGUGUGCUAUCGUCUUCCCUGGGAGGUGUGAUCCUGUUCUUCCUGAGCG

>hsa-mir-3689f MI0016837

AGGUGUGAUAUCGUGCUUCCUGGGACGUGUGAUGCUGUGCUUCCUGGGAGGUGUGAUCCCACACUC

>hsa-mir-3690-1 MI0016091

CCCAUCUCCACCUGGACCCAGCGUAGACAAAGAGGUGUUUCUACUCCAUAUCUACCUGGACCCAGUGUAGAUGGG

>hsa-mir-3691 MI0016092

UUGAGGCACUGGGUAGUGGAUGAUGGAGACUCGGUACCCACUGCUGAGGGUGGGGACCAAGUCUGCGUCAUCCUCUCCUCAGUGCCUCAA

>hsa-mir-3692 MI0016093

CCAUUCCUGCUGGUCAGGAGUGGAUACUGGAGCAAUAGAUACAGUUCCACACUGACACUGCAGAAGUGG

>hsa-mir-3713 MI0016134

GGUAUCCGUUUGGGGAUGGUUUCACUAUCCCCAGAUGGAUACCAA

>hsa-mir-3714 MI0016135

GAAGGCAGCAGUGCUCCCCUGUGACGUGCUCCAUCACCGGGCAGGGAAGACACCGCUGCCACCUC

>hsa-mir-3907 MI0016410

GGGUUGGAAAGCUGUAGGUGUGGAGGGGCAUGGAUACGGGGGCCAUGAGGGUGGGGUCCAGGCUGGACCAGGCCUGCCCUGAGUCCCCCAGCAGGUGCUCCAGGCUGGCUCACACCCUCUGCCUCUCUCUCUUCCUUCCUGGCCCCAACCC

>hsa-mir-3908 MI0016412

GCCUGAGCAAUGUAGGUAGACUGUUUCUAAAAAAAUAAAAAGUUAAAAAAAUUUAUGUUAACGUGUAAUGUGUUUACUAAUUUUUUUUUUUUUUUUUGGAGACAGAGUCUCCCUCUGUCGCCAGGC

>hsa-mir-3909 MI0016413

GGUAUGCUGUUGCGCUGUCCUUCCUCUGGGGAGCAGGCUCCGGGGGACAGGGAAAAGCACACAAGGAACUUGUCCUCUAGGGCCUGCAGUCUCAUGGGAGAGUGACAUGCACCAGGACC

>hsa-mir-3910-1 MI0016414

CUUUUGCUGUCAGUUUUUCUGUUGCUUGUCUUGGUUUUAUGCCUUUUAUAUCAAGGCACAUAAAAGGCAUAAAACCAAGACAAGCAACAAAAAAAGGAUUGAUCACAGAAG

>hsa-mir-3911 MI0016415

GGGUGAGGAUGUGUGUGGAUCCUGGAGGAGGCAGAGAAGACAGUGAGCUUGCCAGUUCUGGUUUCCAACACUUCCUUUCCUGCGCUUCUCGAUUCCCAGAUCUGCACCC

>hsa-mir-3912 MI0016416

AGAGAGGAAUGAACAGUUAAAUUAUAACAUGUCCAUAUUAUGGGUUAGUUGUGGACACAUACUAACGCAUAAUAUGGACAUGUUAUAAUUUAACUGUUCCUUUCU

>hsa-mir-3913-1 MI0016417

UUGUUUAUAAUAAACUGAAAUAUUUGGGACUGAUCUUGAUGUCUGCCAAAACCUUGGCAGACAUCAAGAUCAGUCCCAAAUAUUUCAGUUUAUUAUAGACAG

>hsa-mir-3914-1 MI0016419

UGGACUUCAGAUUUAACUUCUCAUUUUCUGGUUCCUUCUAAUGAGUAUGCUUAACUUGGUAGAAGGAACCAGAAAAUGAGAAGUUGAGUAGGAACUCUA

>hsa-mir-3915 MI0016420

CAAGUUGGCACUGUAGAAUAUUGAGGAAAAGAUGGUCUUAUUGCAAAGAUUUUCAAUAAGACCAUCCUUUCCUCAAUAUUCUGUGGUGUCAUCUUUG

>hsa-mir-3916 MI0016422

AUCCCAGAGAAGAAGGAAGAAGAGGAAGAAAUGGCUGGUUCUCAGGUGAAUGUGUCUGGGUUCAGGGGAUGUGUCUCCUCUUUUCUUCUGGGAU

>hsa-mir-3917 MI0016423

GGCGCUUUUGUGCGCGCCCGGGUCUGUUGGUGCUCAGAGUGUGGUCAGGCGGCUCGGACUGAGCAGGUGGGUGCGGGGCUCGGAGGAGGCGGC

>hsa-mir-3918 MI0016424

AGGCGGUUAAGCCAUGGGACAGGGCCGCAGAUGGAGACUGCUCAAGGUCAAAGGGGUCUCCAGCUGGGACCCUGCACCUGGUUCGUAGCCCCU

>hsa-mir-3919 MI0016425

CCUGAGCACCAUUUACUGAGUCCUUUGUUCUCUACUAGUUUGUAGUAGUUCGUAGCAGAGAACAAAGGACUCAGUAAAUGGUGCUCAGG

>hsa-mir-3920 MI0016427

ACUGAGUGAGGGAGUCAGAGAGUUAAGAGAAUUAGUACAGGUGAGAUUGUACUGAUUAUCUUAACUCUCUGACCCCCUCACUCAGU

>hsa-mir-3921 MI0016428

CCUAGCCCAGUACAAGGCAUAUGGUACUCAAGAGACUUAGAAAUCCCUAAGUCUCUGAGUACCAUAUGCCUUGUACUGGGCUAGG

>hsa-mir-3922 MI0016429

GGAAGAGUCAAGUCAAGGCCAGAGGUCCCACAGCAGGGCUGGAAAGCACACCUGUGGGACUUCUGGCCUUGACUUGACUCUUUC

>hsa-mir-3923 MI0016430

GGUAGAGUGAGCUCUAAUCCAAUAUUACUAGCUUCUUUAUAAGAAGAGGAAACUAGUAAUGUUGGAUUAGGGCUCACUCUACU

>hsa-mir-3924 MI0016432

UAAAUGAAAAAGUAGUAGUCAAAUAUGCAGAUCUAUGUCAUAUAUACAGAUAUGUAUAUGUGACUGCUACUUUUUUGUUUA

>hsa-mir-3925 MI0016433

GUGGGAAUAGCAAGAGAACUGAAAGUGGAGCCUGUCACAUCUCCAGACUCCAGUUUUAGUUCUCUUGCUAUUUCCAC

>hsa-mir-3926-1 MI0016434

AAAAUGGAGCUGGCCAAAAAGCAGGCAGAGACUUUAAAAGCGUCUCUGCCUGCUUUUUGGCCAGCUCCGUUUU

>hsa-mir-3927 MI0016435

UGCCAAUGCCUAUCACAUAUCUGCCUGUCCUAUGACAAACAUGGCAGGUAGAUAUUUGAUAGGCAUUGGCA

>hsa-mir-3928 MI0016438

GCUGAAGCUCUAAGGUUCCGCCUGCGGGCAGGAAGCGGAGGAACCUUGGAGCUUCGGC

>hsa-mir-3929 MI0016439

AGUGGCUCACACCAGUAAUCCCAGCACUUUGGGAGGCUGAUGUGAGUAGACCACU

>hsa-mir-3934 MI0016590

CACAGCCCUUCCUGUCCCCAGUUUUCAGGUGUGGAAACUGAGGCAGGAGGCAGUGAAGUAACUUGCUCAGGUUGCACAGCUGGGAAGUGGAGCAGGGAUUUGAAUCC

>hsa-mir-3935 MI0016591

GGAUGUGUUCCUGUCCCAGAAGGAGCUGAUGGUUGUAUCUAUGAAGGUAAGCAUUUUUGUAGAUACGAGCACCAGCCACCCUAAGCAAAGGCAGAGAAUGCUUA

>hsa-mir-3936 MI0016592

AUGAUUCAGAGCAUCUGUCCAGUGUCUGCUGUAGAUCCCUCAAAUCCGUGUUUGGACGCUUCUGGUAAGGGGUGUAUGGCAGAUGCACCCGACAGAUGCACUUGGCAGCA

>hsa-mir-3937 MI0016593

AGAAGAAUGCCCAACCAGCCCUCAGUUGCUACAGUUCCCUGUUGUUUCAGCUCGACAACAACAGGCGGCUGUAGCAAUGGGGGGCUGGAUGGGCAUCUCAAUGUGC

>hsa-mir-3938 MI0016594

AGGAAUUUUUAACCCGAUCACUAGAUUAUCUACAAGGGAAUUUUUUUUUAAUUUAAAAAAUUCCCUUGUAGAUAACCCGGUGGUCAGGUUGGAUGGCUCCAUG

>hsa-mir-3939 MI0016596

CUGGCUUCCAAAGGCCUCUGUGUGUUCCUGUAUGUGGGCGUGCACGUACCUGUCACAUGUGUACGCGCAGACCACAGGAUGUCCACACUGGCUUCCAAACACAUCU

>hsa-mir-3940 MI0016597

GCUUAUCGAGGAAAAGAUCGAGGUGGGUUGGGGCGGGCUCUGGGGAUUUGGUCUCACAGCCCGGAUCCCAGCCCACUUACCUUGGUUACUCUCCUUCCUUCU

>hsa-mir-3941 MI0016598

GAGUCAGAAUUCUCAUCAGGCUGUGAUGCUCAGUUGUGUGUAGAUUGAAAGCCCUAAUUUUACACACAACUGAGGAUCAUAGCCUGAUGGUUCCUUUUUGUUU

>hsa-mir-3942 MI0016599

UCUUCAGUAUGACACCUCAAAGAAGCAAUACUGUUACCUGAAAUAGGCUGCGAAGAUAACAGUAUUUCAGAUAACAGUAUUACAUCUUUGAAGUGUCAUAUUCACUGAC

>hsa-mir-3943 MI0016600

CACACAGACGGCAGCUGCGGCCUAGCCCCCAGGCUUCACUUGGCGUGGACAACUUGCUAAGUAAAGUGGGGGGUGGGCCACGGCUGGCUCCUACCUGGAC

>hsa-mir-3944 MI0016601

UCCACCCAGCAGGCGCAGGUCCUGUGCAGCAGGCCAACCGAGAAGCGCCUGCGUCUCCCAUUUUCGGGCUGGCCUGCUGCUCCGGACCUGUGCCUGAUCUUAAUGCUG

>hsa-mir-3945 MI0016602

GAUGUUGAUGCACGUGACGGGGAGGGCAUAGGAGAGGGUUGAUAUAAAAUGCAAUUACAGCCUCUUAUGCUUUCCAAAGUGGGGGAUGAUUCAAUGAU

>hsa-mir-3960 MI0016964

GGCGCCCCGGCUCCCCGCGCCCCCGAUCGGGGCCGCCGCUAGUAGUGGCGGCGGCGGAGGCGGGGGCAGCGGCGGCGGCGGCGGAGGCGCC

>hsa-mir-3972 MI0016990

GCCCAUUUGCCUUGGCUUGGGGUGGCAGUCCUGUGGGAAUGAGAGAUGCCAAACUGGACCUGCCAGCCCCGUUCCAGGGCACAGCAU

>hsa-mir-3973 MI0016991

GCCCAGGGUAGCUCUCUGUAUUGCUUGUUACAUUUUAGGAUUGCUUGCCCCUUGCUCCAAUGGUGCAGGCAGAAGAAAUGCAAACAAAGUACAGCAUUAGCCUUAGC

>hsa-mir-3974 MI0016992

GUUCAGGGAAAAGGUCAUUGUAAGGUUAAUGCACCCAUAUUUUAAUAUCAAACUAUGACAAAUUUGACUACAGCCUUUCCGUACCCCUGCCAAAAC

>hsa-mir-3975 MI0016993

CAUGUAGGUGAGUGAUUGCUAUUUCAAAAGACUAGAGGUAGGAAAUGAGGCUAAUGCACUACUUCACAUG

>hsa-mir-3976 MI0016994

UGAGGGAUAUAGAGAGCAGGAAGAUUAAUGUCAUAUUGGAGUUGGACUGCAGGGCUUCCUUUACACAAUAAAUAUUGUAUGAAGUGCUGAUGUAACCUUUACUGCAGCAUGACAUGGGAUUUGGCUGUUUUUAUGGCUC

>hsa-mir-3977 MI0016995

UUGUGCUUCAUCGUAAUUAACCUUAAGUGGUUCGGGUAAAUCACUUUAAUUUGUUAUGUGUUGGCAGAAU

>hsa-mir-3978 MI0016996

UCAGUGGAAAGCAUGCAUCCAGGGUGUGGAGCCAAAAUUAGAAGGGCCAAAAUUCUACCUGGCCCACUACCACAGCAACCUUGGGCAUCGUUUUCUUUUGA

>hsa-mir-4251 MI0015861

CACGUCCUCCAGCUUUUUUCCUUAGUGGCCAAUUCCUGAGAAAAGGGCCAACGUGCUUCCA

>hsa-mir-4252 MI0015864

UGGGGGGCUGGCAGCUCAUCAGUCCAGGCCAUCUGGCCACUGAGUCAGCACCAGCGCCCAAUC

>hsa-mir-4253 MI0015860

CCAGCCAUCGCCCUUGAGGGGCCCUAGGACUUACUUGUGCAGGGCAUGUCCAGGGGGUCCAGGUCUGC

>hsa-mir-4254 MI0015862

CUUGGGAGGAGGGUGGGGUGGCUCCUCUGCAGUGAGUAGGUCUGCCUGGAGCUACUCCACCAUCUCCCCCAGCCCC

>hsa-mir-4255 MI0015863

GAGCAUCCUUCAGUGUUCAGAGAUGGAGUCAGUAUUGGUCUGGCCAUUUUUAGGGCAAAGAGGCAGCAUCAU

>hsa-mir-4256 MI0015855

UGUUCCAUUUAUCUGACCUGAUGAAGGUCUCCUGGCAUUGAUUAGGUCUGAUGAUCCAUUUCUG

>hsa-mir-4257 MI0015856

GGCUUAGAAACAGUCCCUAGGUAGGAUUUGGGGAGGAGCUAAGAAGCCCCUACAGGGCCCAGAGGUGGGGACUGAGCCUUAGUUGG

>hsa-mir-4258 MI0015857

ACGCCCCCCGCCCCGCCACCGCCUUGGAGGCUGACCUCUUACUUUCGGUCGGUCUUCUUCCCUGGGCUUGGUUUGGGGGCGGGGGAGUGUC

>hsa-mir-4259 MI0015858

GAUGGGCCCCUUGUGUCCUGAAUUGGGUGGGGGCUCUGAGUGGGGAAAGUGGGGGCCUAGGGGAGGUCACAGUUGGGUCUAGGGGUCAGGAGGGCCCAGGA

>hsa-mir-4260 MI0015859

AACAAGGUGACUUGGGGCAUGGAGUCCCACUUCCUGGAGCCCACACCCCAGCUUGUCACACACCAAC

>hsa-mir-4261 MI0015868

GGUGGAAGUGGGUUCCUCCCAGUUCCUGAGACAGGAAACAGGGACCCAGGAGACCAGC

>hsa-mir-4262 MI0015872

GAAAGCUGCAGGUGCUGAUGUUGGGGGGACAUUCAGACUACCUGCAGCAGAGCC

>hsa-mir-4263 MI0015876

AUAGUGCUCUUCAGGGUUUUACUUGGGAGAUUGGAGUGGCCAGUGUUCCUAAACAAUUCUAAGUGCCUUGGCCCACAACAUAC

>hsa-mir-4264 MI0015877

AAAGCUGGAUACUCAGUCAUGGUCAUUGUAACAUGAUAGUGACAGGUACUGGGUAAGACUGCAUAG

>hsa-mir-4265 MI0015869

UGCAGUGGGUUGGAGCUUCAGCCUACACCUGUAAAGAAUUGGUCAGCCUGGGGACUGGUGAUCUCUGCAGCUGUGGGCUCAGCUCUGGGCUGGGCCUGG

>hsa-mir-4266 MI0015870

CCACUGCUGGCCGGGGCCCCUACUCAAGGCUAGGAGGCCUUGGCCAAGGACAGUC

>hsa-mir-4267 MI0015871

CUCAGCAGGCUCCAGCUCGGUGGCACUGGGGGAAGGCUCCAGACCCCAGCCUCUGUCAUCCCUGCAUGGAGCCCACAUCUCC

>hsa-mir-4268 MI0015874

AUGCACAUCAGGUUCUAGAGGUUUUGCCCUAGCGGCUCCUCCUCUCAGGAUGUGAUGUCACCUG

>hsa-mir-4269 MI0015875

ACAGCGCCCUGCAGGCACAGACAGCCCUGGCUUCUGCCUCUUUCUUUGUGGAAGCCACUCUGUCAGGCCUGGGAUGGAGGGGCA

>hsa-mir-4270 MI0015878

ACAAAUAGCUUCAGGGAGUCAGGGGAGGGCAGAAAUAGAUGGCCUUCCCCUGCUGGGAAGAAAGUGGGUC

>hsa-mir-4271 MI0015879

AAAUCUCUCUCCAUAUCUUUCCUGCAGCCCCCAGGUGGGGGGGAAGAAAAGGUGGGGAAUUAGAUUC

>hsa-mir-4272 MI0015880

UUUUCUGCACAAAUUAAUCAGUUAAUGCAUAGAAAGCAUUCAACUAGUGAUUGUGUUAUAAGAG

>hsa-mir-4273 MI0015881

UCCCCUGUGUGUGUUCUCUGAUGGACAGUAAGCCUUGACUUAUGGCUAAAUGCUUCUUCACAAUGGUCACAUGCAUAGGGCUUU

>hsa-mir-4274 MI0015884

GGGGCAUUUAGGGUAACUGAGCUGCUGCCGGGGCCUGGCGCUCCUCUACCUUGUCAGGUGACCCAGCAGUCCCUCCCCCUGCAUGGUGCCC

>hsa-mir-4275 MI0015883

ACAUUUUUGUCCAAUUACCACUUCUUUUUGCCACCUGAGCACAGUCAGCAGUCAGCAUAAAAAAGUGAUAAUGGGAAGUUAAUGUCU

>hsa-mir-4276 MI0015882

CACAGUCUGACUCAGUGACUCAUGUGCUGGCAGUGGCCACGUAAAUAGAGCUACUGUGUCUGAAAGCAAU

>hsa-mir-4277 MI0015886

CUGGGUCGAGGCAGUUCUGAGCACAGUACACUGGGCUGCCCCCACUGCCCAGUGCCCUGCUCAGCUCAAGUCCUUGUGCCCCUC

>hsa-mir-4278 MI0015888

AUCUAACACCAGGAGAAUCCCAUAGAACAUUGACAUCAACACUAGGGGGUUUGCCCUUGUGGGGAAGAA

>hsa-mir-4279 MI0015887

UGCUCUGUGGAGCUGAGGAGCAGAUUCUCUCUCUCUCCUCCCGGCUUCACCUCCUGAG

>hsa-mir-4280 MI0015889

AAUCAGGGUGGAGUGUAGUUCUGAGCAGAGCCUUAAAGGAUGAGGUAUGUUCAAGACUGAAUGACACCUUUGUGAU

>hsa-mir-4281 MI0015885

GCUGGGGGUCCCCCGACAGUGUGGAGCUGGGGCCGGGUCCCGGGGAGGGGGGUUCUGGGCAG

>hsa-mir-4282 MI0015890

GGUGAAGUUCCAGGGGAAGAUUUUAGUAUGCCACAUUUCUAAAAUUUGCAUCCAGGAACAUCAUCCU

>hsa-mir-4283-1 MI0015892

ACUCUGAUCCUGGGGCUCAGCGAGUUUGCAAGGGGUGUUUCUGUCCAUGGUCAGGCUUGCCAGCCUUGGUCCUUGGGCCC

>hsa-mir-4284 MI0015893

GUUCUGUGAGGGGCUCACAUCACCCCAUCAAAGUGGGGACUCAUGGGGAGAGGGGGUAGUUAGGAGCUUUGAUAGAGGCGG

>hsa-mir-4285 MI0015891

AUUAGCUGGGGCGGCGAGUCCGACUCAUCAAUAUUUUAAGGAAUGACCCGGCCUUGGGGUGCGGAAUUGCUGCGCGGGCGGGGGC

>hsa-mir-4286 MI0015894

UACUUAUGGCACCCCACUCCUGGUACCAUAGUCAUAAGUUAGGAGAUGUUAGAGCUGUGAGUACCAUGACUUAAGUGUGGUGGCUUAAACAUG

>hsa-mir-4287 MI0015895

UAGUUCUUUUUCUCCCUUGAGGGCACUUUUCAGUUCCUGAGAUCAAUGUGGUCCCUACUGGGGAGACCAUAGGAGCCC

>hsa-mir-4288 MI0015896

AUGGAGGUGGAGAGUCAUCAGCAGCACUGAGCAGGCAGUGUUGUCUGCUGAGUUUCCACGUCAUUUG

>hsa-mir-4289 MI0015898

CCUUGGGAGGGCAUUGUGCAGGGCUAUCAGGCAGUUUCCUGGGCCCUGUCUGCAGAGCCUAAACAGAUCA

>hsa-mir-4290 MI0015899

GCCACCAAGAAGGUGAAGGGAGGGUCAGUCCCAAUCUGAAUCCCACCAAAAUAGGUGGUAGAGGGUUGCCCUCCUUUCUUCCCUCACCUCUGACC

>hsa-mir-4291 MI0015900

CGCCGGGGGCUUCAGCAGGAACAGCUGGGUGGAGGCAGAGCUGUUCUGCUGUGGCUGCAGCCCUG

>hsa-mir-4292 MI0015897

GAGACACCAGAAGGCCACCUGCUUAGGAGGCCAGAGGUGCCCCUGGGCCGGCCUUGGUGAGGGGCCC

>hsa-mir-4293 MI0015826

AGAGACACCUGUUCCUUGGGAAGCUGGUGACAUUGCUAAUUCAUUUCACACCAGCCUGACAGGAACAGCCUGACUGAA

>hsa-mir-4294 MI0015827

CCGAUGCCUCGGGAGUCUACAGCAGGGCCAUGUCUGUGAGGGCCCAAGGGUGCAUGUGUCUCCCAGGUUUCGGUGC

>hsa-mir-4295 MI0015822

CUUUGUGGAACAGUGCAAUGUUUUCCUUGCCUGUGGCAAGACCACUUCGGUUCAAGGCUAAGAAACUAGACUGUUCCUACAGAGA

>hsa-mir-4296 MI0015823

UUGGGCUUUGAUGUGGGCUCAGGCUCAGAGGGCUGAAGUGGUUGUGGGGAGGGGCUUCUGGGGACUGUGUCCAUGUCUCUGUCGUUUU

>hsa-mir-4297 MI0015824

AGCACGCACGUGCCUUCCUGUCUGUGCCUGCCUUCGAAGUGCACGGCAGGGCCAGGACGGGUCGCUGUGGGUGGGG

>hsa-mir-4298 MI0015830

GGGGAGGUACCUGGGACAGGAGGAGGAGGCAGCCUUGCCUCAGAAACCAAACUGUCAAAAGUGUAGGUUCCAC

>hsa-mir-4299 MI0015829

GGGUUCUGACCAAUCAUGUUACAGUGUUUUCUCCUUUAGAGAGAGCUGGUGACAUGAGAGGCAGAAAAAGGA

>hsa-mir-4300 MI0015831

UGAGUUUAGAAGAGGGCCAGCUAAAUCAGCAGAGACAUGAGGUGAUCAAAAACCUUUUUUCAAAGCAGUGGGAGCUGGACUACUUCUGAACCAAUA

>hsa-mir-4301 MI0015828

ACCAGCCACCUCCCACUACUUCACUUGUGAACAUUGCAUUCGUGGAGGGUGGCAGGUGCAGCUCUG

>hsa-mir-4302 MI0015833

UCAGGAGGGACCAGUGUGGCUCAGCGAGGUGGCUGAGUUUACUUAAGGUAUUGGAAUGAG

>hsa-mir-4303 MI0015834

AGAAAAUAGCUUCUGAGCUGAGGACAGCUUGCUCUGCUUUUCUUUAGCUUAGGAGCUAACCAUGGU

>hsa-mir-4304 MI0015832

AGAGAAGUGGCCGGCAUGUCCAGGGCAUCCCCAUUGCUCUGUGACUGCUGCCAUCCUUCUCC

>hsa-mir-4305 MI0015835

CUGCCUUAGACCUAGACACCUCCAGUUCUGGGUUCUUAGAGGCCUAAUCCUCUACAAACUCAGUUUUCAGACUGUGAGGGAAAUUCUCUGUCUUAUUGCUUU

>hsa-mir-4306 MI0015836

AAGCUGCUUAGUGUCCUUAGAGUCUCCAGAGGCAUCCCUAACCCAGAAUCUUUUGACUGUCCUCUGGAGAGAAAGGCAGUAGGUCUGUACC

>hsa-mir-4307 MI0015838

UCAGAAGAAAAAACAGGAGAUAAAGUUUGUGAUAAUGUUUGUCUAUAUAGUUAUGAAUGUUUUUUCCUGUUUCCUUCAGGGCCA

>hsa-mir-4308 MI0015839

UAUGGGUUCAGAGGGAACUCCAUUGGACAGAAAUUUCCUUUUGAGGAAAUCUUUCCCUGGAGUUUCUUCUUACCUUUUUCC

>hsa-mir-4309 MI0015837

UCUGGGGGUUCUGGAGUCUAGGAUUCCAGGAUCUGGGUUUUGAGGUCUUGGGUUGUAGGGUCUGCGGUUUGAAGCCCCUCUUG

>hsa-mir-4310 MI0015840

UGGCGUCUGGGGCCUGAGGCUGCAGAACAUUGCAGCAUUCAUGUCCCACCCCCACCA

>hsa-mir-4311 MI0015841

UCAGAGAGGGGAAAGAGAGCUGAGUGUGACCUGGAGCAGCUCAGGAGGGCUUCCUGGGUGAGGUGGCAGGUUACAGGUUCGAUCUUUGGCCCUCAGAUUC

>hsa-mir-4312 MI0015842

GAAAGGUUGGGGGCACAGAGAGCAAGGAGCCUUCCCCAGAGGAGUCAGGCCUUGUUCCUGUCCCCAUUCCUCAGAG

>hsa-mir-4313 MI0015843

GAUCAGGCCCAGCCCCCUGGCCCCAAACCCUGCAGCCCCAGCUGGAGGAUGAGGAGAUGCUGGGCUUGGGUGGGGGAAUCAGGGGUGUAAAGGGGCCUGCU

>hsa-mir-4314 MI0015846

GGCCAUUCCUCUCUGGGAAAUGGGACAGGUAGUGGCCACAGUGAGAAAGCUGGCCUGUCCUUCUGCCCCAGGGCCCAGAGUCUGUGACUGGA

>hsa-mir-4315-1 MI0015844

UGGGCUUUGCCCGCUUUCUGAGCUGGACCCUCUCUCUACCUCUGGUGCAGAACUACAGCGGAAGGAAUCUCUG

>hsa-mir-4316 MI0015845

AGUGGCCCAGGGUGAGGCUAGCUGGUGUGGUCACCCACUCUCCAGCCCAGCCCCAAUCCCACCACAACCAC

>hsa-mir-4317 MI0015850

AAAAGGCGAGACAUUGCCAGGGAGUUUAUUUUGUAGCUCUCUUGAUAAAAUGUUUUAGCAAACAC

>hsa-mir-4318 MI0015847

GCUUCUUAAUUAUGUCAUAAACCCACUGUGGACAAGGGCCUUGUCUUAGACAGUCACUGUGGGUACAUGCUAGGUGCUCAA

>hsa-mir-4319 MI0015848

UUGGCUUGAGUCCCUGAGCAAAGCCACUGGGAAUGCUCCCUGAGGACGUUAUAUGAGUGCUCAGCUCAUGGGGCUAUGAUGGUCA

>hsa-mir-4320 MI0015849

GACAUGUGGGGUUUGCUGUAGACAUUUCAGAUAACUCGGGAUUCUGUAGCUUCCUGGCAACUUUG

>hsa-mir-4321 MI0015852

CUGGUCUCCGCAGAGCCUCUGCCCCUCCCGAGACACCCGCUACCUGGUGUUAGCGGUGGACCGCCCUGCGGGGGCCUGGC

>hsa-mir-4322 MI0015851

ACCGCGAGUUCCGCGCCUGGCCGUGUCGCCCCACGAGGGGGACUGUGGGCUCAGCGCGUGGGGCCCGGAGCAU

>hsa-mir-4323 MI0015853

CGGGGCCCAGGCGGGCAUGUGGGGUGUCUGGAGACGCCAGGCAGCCCCACAGCCUCAGACCUCGGGCAC

>hsa-mir-4324 MI0015854

CGGCCCCUUUGUUAAGGGUCUCAGCUCCAGGGAACUUUAAAACCCUGAGACCCUAACCUUAAAGGUGCUGCA

>hsa-mir-4325 MI0015865

GGGGAAGAUGUUGCACUUGUCUCAGUGAGAGAUGCUUCUAGAUCCAGGAGGCAGACCUCAAGGAUGGAGAGAAGGCAGAUCCUUUGAGAU

>hsa-mir-4326 MI0015866

GCUGCUCUGCUGUUCCUCUGUCUCCCAGACUCUGGGUGGAUGGAGCAGGUCGGGGGCCA

>hsa-mir-4327 MI0015867

GGCCUGGGUAGGCUUGCAUGGGGGACUGGGAAGAGACCAUGAACAGGUUAGUCCAGGGAGUUCUCAUCAAGCCUUUACUCAGUAG

>hsa-mir-4328 MI0015904

AACAGUUGAGUCCUGAGAACCAUUGAGAACCAGUUUUCCCAGGAUUAACUGUUCCG

>hsa-mir-4329 MI0015901

UAGAGAGGAAGGUGUACCAGGGUUUUGGAGUUUUUUUUUCCUCCUGAGACCCUAGUUCCACAUUCUGGAGC

>hsa-mir-4330 MI0015902

AAUUGUCAGCAGGCAAUUAUCUGAGGAUGCAGGAGAGGAAGGGGGCUUCUUUUUGACGCCUACUUCAUCAGCUGCUCCUCAGAUCAGAGCCUUGCAGGUCAGGCC

>hsa-mir-4417 MI0016753

GAAAACAACCAGGUGGGCUUCCCGGAGGGCGGAACACCCAGCCCCAGCAUCCAGGGCUCACCUACCACGUUUG

>hsa-mir-4418 MI0016754

UGGUUUUUGCUCUGAGUGACCGUGGUGGUUGUGGGAGUCACUGCAGGACUCAGCAGGAAUUC

>hsa-mir-4419a MI0016755

UGGUGGUGUGUGCCUGUAGUCUUAGCUACUCGGGAGGCUGAGGGAGGAGACUGCAGUGAGUGGAGGUCACGCCACUG

>hsa-mir-4419b MI0016861

CUCAGGCUCAGUGGUGCAUGCUUAUAGUCCCAGCCACUCUGGAGGCUGAAGGAAGAUGGCUUGAGCCU

>hsa-mir-4420 MI0016757

CUCUUGGUAUGAACAUCUGUGUGUUCAUGUCUCUCUGUGCACAGGGGACGAGAGUCACUGAUGUCUGUAGCUGAGAC

>hsa-mir-4421 MI0016758

CUGGGUCUCCUUUCUGCUGAGAGUUGAACACUUGUUGGGACAACCUGUCUGUGGAAAGGAGCUACCUAC

>hsa-mir-4422 MI0016759

AGUUCUUCUGCAGACAAAAGCAUCAGGAAGUACCCACCAUGUACCAGUGGGCCCUUCUUGAUGCUCUUGAUUGCAGAGGAGCC

>hsa-mir-4423 MI0016760

AUCAUGUACUGCAGUUGCCUUUUUGUUCCCAUGCUGUUUAAGCCUAGCAUAGGCACCAAAAAGCAACAACAGUAUGUGAA

>hsa-mir-4424 MI0016763

CUUACAUCACACACAGAGUUAACUCAAAAUGGACUAAUUUUUCCACUAGUUAGUCCAUUUCAAGUUAACUCUGUGUGUGAUGUAGU

>hsa-mir-4425 MI0016764

GUGCUUUACAUGAAUGGUCCCAUUGAAUCCCAACAGCUUUGCGAAGUGUUGUUGGGAUUCAGCAGGACCAUUCGUGUAAAGUAA

>hsa-mir-4426 MI0016765

AGUUGGAAGAUGGACGUACUUUGUCUGACUACAAUAUUCAAAAGGAGUCUACUCUUCAUCUUG

>hsa-mir-4427 MI0016766

GAAGCCUCUUGGGGCUUAUUUAGACAAUGGUUUCAUCAUUUCGUCUGAAUAGAGUCUGAAGAGUCUUU

>hsa-mir-4428 MI0016767

UUGGCAGGUGCCAUGUUGCCUGCUCCUUACUGUACACGUGGCUGGCAAGGAGACGGGAACAUGGAGCCGCCAU

>hsa-mir-4429 MI0016768

AGGGAGAAAAGCUGGGCUGAGAGGCGACUGGUGUCUAAUUUGUUUGUCUCUCCAACUCAGACUGCCUGGCCCA

>hsa-mir-4431 MI0016771

UGGUUUGCGACUCUGAAAACUAGAAGGUUUAUGACUGGGCAUUUCUCACCCAAUGCCCAAUAUUGAACUUUCUAGUUGUCAGAGUCAUUAACCC

>hsa-mir-4432 MI0016772

GCAUCUUGCAGAGCCGUUCCAAUGCGACACCUCUAGAGUGUCAUCCCCUAGAAUGUCACCUUGGAAAGACUCUGCAAGAUGCCU

>hsa-mir-4433b MI0025511

UGUGUUCCCUAUCCUCCUUAUGUCCCACCCCCACUCCUGUUUGAAUAUUUCACCAGAAACAGGAGUGGGGGGUGGGACGUAAGGAGGAUGGGGGAAAGAACA

>hsa-mir-4434 MI0016774

UCACUUUAGGAGAAGUAAAGUAGAACUUUGGUUUUCAACUUUUCCUACAGUGU

>hsa-mir-4435-1 MI0016775

AGGCAGCAAAUGGCCAGAGCUCACACAGAGGGAUGAGUGCACUUCACCUGCAGUGUGACUCAGCAGGCCAACAGAUGCUA

>hsa-mir-4436a MI0016776

GCCUCACUUUUCCACUUAUGCCUGCCCUGCCCCUCGAAUCUGCUCCACGAUUUGGGCAGGACAGGCAGAAGUGGAUAAGUGAGGA

>hsa-mir-4436b-1 MI0017425

GUGUCCUCACUUGUCCACUUCUGCCUGCCCUGCCCAAAUGGUGGAGCAGAUUCGAGGGGCAGGGCAGGAAGAAGUGGACAAGUGAGGCCAU

>hsa-mir-4437 MI0016778

ACUUUGUGCAUUGGGUCCACAAGGAGGGGAUGACCCUUGUGGGCUCAGGGUACAAAGGUU

>hsa-mir-4438 MI0016781

UAAGUGUAAACUUAAGGACUGUCUUUUCUAAGCCUGUGCCUUGCCUUUCCUUUGGCACAGGCUUAGAAAAGACAGUCUUUAAGUUUACACUUC

>hsa-mir-4439 MI0016782

CCAGUGACUGAUACCUUGGAGGCAUUUUAUCUAAGAUACACACAAAGCAAAUGCCUCUAAGGUAUCAGUUUACCAGGCCA

>hsa-mir-4440 MI0016783

CUCUCACCAAGCAAGUGCAGUGGGGCUUGCUGGCUUGCACCGUGACUCCCUCUCACCAAGCAAGUGUCGUGGGGCUUGCUGGCUUGCACUGUGAAGAU

>hsa-mir-4441 MI0016784

CAGAGUCUCCUUCGUGUACAGGGAGGAGACUGUACGUGAGAGAUAGUCAGAUCCGCAUGUUAGAGCAGAGUCUCCUUCGUGUACAGGGAGGAGAUUGUAC

>hsa-mir-4442 MI0016785

GCGCCCUCCCUCUCUCCCCGGUGUGCAAAUGUGUGUGUGCGGUGUUAUGCCGGACAAGAGGGAGGUG

>hsa-mir-4443 MI0016786

GGUGGGGGUUGGAGGCGUGGGUUUUAGAACCUAUCCCUUUCUAGCCCUGAGCA

>hsa-mir-4444-1 MI0016787

GUGACGACUGGCCCCGCCUCUUCCUCUCGGUCCCAUAUUGAACUCGAGUUGGAAGAGGCGAGUCCGGUCUCAAA

>hsa-mir-4445 MI0016788

UUCCUGCAGAUUGUUUCUUUUGCCGUGCAAGUUUAAGUUUUUGCACGGCAAAAGAAACAAUCCAGAGGGU

>hsa-mir-4446 MI0016789

CUGGUCCAUUUCCCUGCCAUUCCCUUGGCUUCAAUUUACUCCCAGGGCUGGCAGUGACAUGGGUCAA

>hsa-mir-4447 MI0016790

GUUCUAGAGCAUGGUUUCUCAUCAUUUGCACUACUGAUACUUGGGGUCAGAUAAUUGUUUGUGGUGGGGGCUGUUGUUUGCAUUGUAGGAU

>hsa-mir-4448 MI0016791

AGGAGUGACCAAAAGACAAGAGUGCGAGCCUUCUAUUAUGCCCAGACAGGGCCACCAGAGGGCUCCUUGGUCUAGGGGUAAUGCCA

>hsa-mir-4449 MI0016792

AGCAGCCCUCGGCGGCCCGGGGGGCGGGCGGCGGUGCCCGUCCCGGGGCUGCGCGAGGCACAGGCG

>hsa-mir-4450 MI0016795

UGUCUGGGGAUUUGGAGAAGUGGUGAGCGCAGGUCUUUGGCACCAUCUCCCCUGGUCCCUUGGCU

>hsa-mir-4451 MI0016797

UCUGUACCUCAGCUUUGCUCCCAACCAACCACUUCCACAUGUUUUGCUGGUAGAGCUGAGGACAGC

>hsa-mir-4452 MI0016798

UGGAUCACUUGAGGCCAAGAGUGCAAGGCUGUAGUGUGCACAGCCUUGAAUUCUUGGCCUUAAGUGAUCCC

>hsa-mir-4453 MI0016799

UGGAGAGCUUGGUCUGUAGCGGUUUCCUUCGGGGCAGGUGGGGACUGCUCCUUUGGGAGGAAGGAGGAGGCCCAGGCCGCGUCUUCAGG

>hsa-mir-4454 MI0016800

CCGGAUCCGAGUCACGGCACCAAAUUUCAUGCGUGUCCGUGUGAAGAGACCACCA

>hsa-mir-4455 MI0016801

AGAAGGGUGUGUGUGUUUUUCCUGAGAAUAAGAGAAGGAAGGACAGCCAAAUUCUUCA

>hsa-mir-4456 MI0016802

AUGAACCUGGUGGCUUCCUUUUCUGGGAGGAAGUUAGGGUUCA

>hsa-mir-4457 MI0016803

GGAGUACUCCAGUCAAUACCGUGUGAGUUAGAAAAGCUCAAUUCACAAGGUAUUGACUGGCGUAUUCA

>hsa-mir-4458 MI0016804

GAGCGCACAGAGGUAGGUGUGGAAGAAAGUGAAACACUAUUUUAGGUUUUAGUUACACUCUGCUGUGGUGUGCUG

>hsa-mir-4459 MI0016805

ACCCAGGAGGCGGAGGAGGUGGAGGUUGCAGUGAGCCAAGAUCGUGGCACUGACUCCAGCCUGGGG

>hsa-mir-4460 MI0016806

GUUUUUUGCCCAUAGUGGUUGUGAAUUUACCUUCUCCUCUUUGCAGUGAUAAAGGAGGUAAAUUCACAACCACUGUGGGCAGAAAC

>hsa-mir-4461 MI0016807

GAGUAGGCUUAGGUUAUGUACGUAGUCUAGGCCAUACGUGUUGGAGAUUGAGACUAGUAGGGCUAGGCCUACUG

>hsa-mir-4462 MI0016810

CUUCCCAGCUGCCCUAAGUCAGGAGUGGCUUUCCUGACACGGAGGGUGGCUUGGGAAA

>hsa-mir-4463 MI0016811

AAUAGAUUAUUGGUCACCACCUCCAGUUUCUGAAUUUGUGAGACUGGGGUGGGGCCUGAGAAUUUGC

>hsa-mir-4464 MI0016812

GGAACCUUAGUAAGGUUUGGAUAGAUGCAAUAAAGUAUGUCCACAGCUGAAAGGACAUACUUUAUUGCAUGUAUCCAAACCUUACUAAUUCA

>hsa-mir-4465 MI0016816

CAUGUGUCCCCUGGCACGCUAUUUGAGGUUUACUAUGGAACCUCAAGUAGUCUGACCAGGGGACACAUGA

>hsa-mir-4466 MI0016817

ACGCGGGUGCGGGCCGGCGGGGUAGAAGCCACCCGGCCCGGCCCGGCCCGGCGA

>hsa-mir-4467 MI0016818

UGGUGGCGGCGGUAGUUAUGGGCUUCUCUUUCUCACCAGCAGCCCCUGGGCCGCCGCCUCCCU

>hsa-mir-4468 MI0016819

AGUCUUCUCCUGGGGCUUUGGUGGCUAUGGUUGACUGGGCCACUCAGAGCAGAAGGAUGAGAUG

>hsa-mir-4469 MI0016820

CCGACGCGGAGAGCGGCUCUAGGUGGGUUUGGCGGCGGCGAGGACACCGCCGCUCCCUCUAGGGUCGCUCGGAGCGUGA

>hsa-mir-4470 MI0016821

CGAGCCUCUUUCGGCUUUCCAGUUUGUCUCGGUCCUUUGGAACGUGGCAAACGUGGAAGCCGAGAGGGCUCU

>hsa-mir-4471 MI0016822

CCAAAUUUAAAACUUAAACCUCUACUAAGUUUCCAUGAAAAGAACCCAUGGGAACUUAGUAGAGGUUUAAGUUUUAAAUUUGA

>hsa-mir-4472-1 MI0016823

UGGCAGACCCUUGCUCUCUCACUCUCCCUAAUGGGGCUGAAGACAGCUCAGGGGCAGGGUGGGGGGUGUUGUUUUUGUUU

>hsa-mir-4472-2 MI0016824

UGGUGGGGGUGGGGGGUGUUGUUUUUGUUUUUGAGACAGAGUCUUGCUCCGUCGCCCAGGCCGGAGU

>hsa-mir-4473 MI0016825

AAGGAACAGGGGACACUUGUAAUGGAGAACACUAAGCUAUGGACUGCUAUGGACUGCUAGUGCUCUCCGUUACAAGUAUCCCCUGUUACCU

>hsa-mir-4474 MI0016826

UUGCCUACCUUGUUAGUCUCAUGAUCAGACACAAAUAUGGCUCUUUGUGGCUGGUCAUGAGGCUAACAAGGUAGGCAC

>hsa-mir-4475 MI0016827

AUCUCAAUGAGUGUGUGGUUCUAAAUGACUCAUAGUCAAGGGACCAAGCAUUCAUUAUGAA

>hsa-mir-4476 MI0016828

AAAAGCCUGUCCCUAAGUCCCUCCCAGCCUUCCAGAGUUGGUGCCAGGAAGGAUUUAGGGACAGGCUUUG

>hsa-mir-4477a MI0016829

UCCUCCUCCCAUCAAUCACAAAUGUCCUUAAUGGCAUUUAAGGAUUGCUAUUAAGGACAUUUGUGAUUCACGGGAGGAGGU

>hsa-mir-4478 MI0016831

GGCCGAGGCUGAGCUGAGGAGCCUCCAAACCUGUAGACAGGGUCAUGCAGUACUAGGGGCGAGCCUCAUCCCCUGCAGCCCUGGCC

>hsa-mir-4479 MI0016838

GAAACCAAGUCCGAGCGUGGCUGGCGCGGGAAAGUUCGGGAACGCGCGCGGCCGUGCUCGGAGCAGCGCCA

>hsa-mir-4480 MI0016841

GCAGAGGUGAGUUGACCUCCACAGGGCCACCCAGGGAGUAAGUAGCCAAGUGGAAGUUACUUUACCUCUGU

>hsa-mir-4481 MI0016842

GGAGUGGGCUGGUGGUUUUUUAAGAGGAAGGGAGACCUAAGCUAGCACAUGAGCACGCUC

>hsa-mir-4482 MI0016843

AGUGAGCAACCCAGUGGGCUAUGGAAAUGUGUGGAAGAUGGCAUUUCUAUUUCUCAGUGGGGCUCUUACC

>hsa-mir-4483 MI0016844

AAAAAACAACAUACUUAGUGCAUACCCAUAUAAUAUUAGGGGUGGUCUGUUGUUGUUUUUCU

>hsa-mir-4484 MI0016845

GGGUUUCCUCUGCCUUUUUUUCCAAUGAAAAUAACGAAACCUGUUAUUUCCCAUUGAGGGGGAAAAAGGCGGGAGAAGCCCCA

>hsa-mir-4485 MI0016846

AGAGGCACCGCCUGCCCAGUGACAUGCGUUUAACGGCCGCGGUACCCUAACUGUGCA

>hsa-mir-4486 MI0016847

GCAUGCUGGGCGAGGCUGGCAUCUAGCACAGGCGGUAGAUGCUUGCUCUUGCCAUUGCAAUGA

>hsa-mir-4487 MI0016848

ACUGUCCUUCAGCCAGAGCUGGCUGAAGGGCAGAAGGGAACUGUCCUUCAGCCAGAGCUGGCUGAAGGGCAGA

>hsa-mir-4488 MI0016849

GGUAGGGGGCGGGCUCCGGCGCUGGGACCCCACUAGGGUGGCGCCUUGGCCCCGCCCCGCCC

>hsa-mir-4489 MI0016850

GGGGGUGGGGCUAGUGAUGCAGGACGCUGGGGACUGGAGAAGUCCUGCCUGACCCUGUCCCA

>hsa-mir-4490 MI0016852

AUAGUUUCUGCAAUGCUCAAAUCUCUGGCCAAAGACCAGAACUUAAUGGUCUCUGGUAAGAGAUUUGGGCAUAUUAGAAACUAA

>hsa-mir-4491 MI0016853

ACAUUUGGUCACACCAGUCCACAUUAACGUGGACCAGACAAUAUUAAUGUGGACUGGUGUGACCAAAA

>hsa-mir-4492 MI0016854

CUGCAGCGUGCUUCUCCAGGCCCCGCGCGCGGACAGACACACGGACAAGUCCCGCCAGGGGCUGGGCGCGCGCCAGCCGG

>hsa-mir-4493 MI0016855

CCAGAGAUGGGAAGGCCUUCCGGUGAUUAUCACAGCCAUGCCUUUACCUCCAGAAGGCCUUUCCAUCUCUGUC

>hsa-mir-4494 MI0016856

AGUUUUAGUUACCCUGGUCAUCUGCAGUCUGAAAAUACAAAAUGGAAAAUUCCAGACUGUGGCUGACCAGAGGUAACUGAAACC

>hsa-mir-4495 MI0016857

AAGAAAUGUAAACAGGCUUUUUGCUCAGUGGAGUUAUUUUGAGCAAAAAGCUUAUUUACAUUUCUG

>hsa-mir-4496 MI0016858

ACAUCAGCUCAUAUAAUCCUCGAAGCUGCCUUUAGAAAUGAGGAAACUGAAGCUGAGAGGG

>hsa-mir-4497 MI0016859

ACCUCCGGGACGGCUGGGCGCCGGCGGCCGGGAGAUCCGCGCUUCCUGAAUCCCGGCCGGCCCGCCCGGCGCCCGUCCGCCCGCGGGUC

>hsa-mir-4498 MI0016860

AGGGCUGGGCUGGCAGGGCAAGUGCUGCAGAUCUUUGUCUAAGCAGCCCCUGCCUUGGAUCUCCCA

>hsa-mir-4499 MI0016862

AAGACUGAGAGGAGGGAACUGGUGAGUUGUACAUAGAAAUGCUUUCUAACUCCUUGUCUCAGUCUGUUU

>hsa-mir-4500 MI0016863

CAGGAGAGAAAGUACUGCCCAGAAGCUAAAGUGUAGAUCAAACGCAUAAUGGCUGAGGUAGUAGUUUCUUGAACUU

>hsa-mir-4501 MI0016864

UAUGUGACCUCGGAUGAAUCACUGAAAUAUGUCUGAGCUUCUGUUUCAUCAGAUGUCACAUUUU

>hsa-mir-4502 MI0016865

AGCCUUUAGCAAGUUGUAAUCUUUUUGCUGAUGGAGGGUCUUGCCUCCAUGGGGAUGGCUGAUGAUGAUGGUGCUGAAGGC

>hsa-mir-4503 MI0016866

ACAAUGUAGAUAUUUAAGCAGGAAAUAGAAUUUACAUAUAAAUUUCUAUUUGUUUCUAUUUCCUGCUUAAAUAUCUACAUUGC

>hsa-mir-4504 MI0016867

CUAAGAUAAUGUCCUCCAGGUUCAUCUCUGUUGUCAUUUGUGGCAUGGACCAUUUGUGACAAUAGAGAUGAACAUGGAGGAUAUUAUCUUAA

>hsa-mir-4505 MI0016868

GGAGGCUGGGCUGGGACGGACACCCGGCCUCCACUUUCUGUGGCAGGUACCUCCUCCAUGUCGGCCCGCCUUG

>hsa-mir-4506 MI0016869

UGGCCUCUGCCAUCAGACCAUCUGGGUUCAAGUUUGGCUCCAUCUUUAUGAAAUGGGUGGUCUGAGGCAAGUGGUCU

>hsa-mir-4507 MI0016871

UCUGGGCUGAGCCGAGCUGGGUUAAGCCGAGCUGGGUUGGGCUGGGCUGGGU

>hsa-mir-4508 MI0016872

AGGACCCAGCGGGGCUGGGCGCGCGGAGCAGCGCUGGGUGCAGCGCCUGCGCCGGCAGCUGCAAGGGCCG

>hsa-mir-4509-1 MI0016873

CUUUAAUACUAUCUCAAACUAAAGGAUAUAGAAGGUUUUCCCUUUCUCUUGCCCUGAAACCUUCUGUAUCCUUUAUUUUGAGAUAGUAUUAGAA

>hsa-mir-4510 MI0016876

GUGUAUGUGAGGGAGUAGGAUGUAUGGUUGUUAGAUAGACAACUACAAUCUUUUCUCACAACAGACAG

>hsa-mir-4511 MI0016877

AAAAAAAAGGGAAAGAAGAACUGUUGCAUUUGCCCUGCACUCAGUUUGCACAGGGUAAAUGCAAUAGUUCUUCUUUCCCUUUUUUUA

>hsa-mir-4512 MI0016878

CUCAGCCCGGGCAAUAUAGUGAGACCUCGUCUCUACAAAAAAUUGAGACAGGGCCUCACUGUAUCGCCCAGGCUGGA

>hsa-mir-4513 MI0016879

AUUCUAGGUGGGGAGACUGACGGCUGGAGGCCCAUAAGCUGUCUAAAACUUCGGCCCCCAGAUUUCUGGUCUCCCCACUUCAGAAC

>hsa-mir-4514 MI0016880

GUUGAGACAGGCAGGAUUGGGGAAACAUCUUUUACCUCGUCUCUUGCCUGUUUUAGA

>hsa-mir-4515 MI0016881

GCGGGAGGUGUAACAGGACUGGACUCCCGGCAGCCCCAGGGCAGGGGCGUGGGGAGCUGGUCCUAGCUCAGCGCUCCCGGA

>hsa-mir-4516 MI0016882

AGGGAGAAGGGUCGGGGCAGGGAGGGCAGGGCAGGCUCUGGGGUGGGGGGUCUGUGAGUCAGCCACGGCUCUGCCCACGUCUCCCC

>hsa-mir-4517 MI0016883

AGGUAAAUAUGAUGAAACUCACAGCUGAGGAGCUUAGCAAGUAGCUAAGGCCAGAGCUUGUGUUUGGGUGGUGUGGCUG

>hsa-mir-4518 MI0016884

UGGGGGAAAAGUGCUGGGAUUGAUUAGUGAUGUCUGCUGGGGAACCGGGGCUCAGGGAUGAUAACUGUGCUGAGAAGCCCCCU

>hsa-mir-4519 MI0016885

AACCUCAGCAGUGCGCAGGGCUGCACUGUCUCCGUCUGCGGCCUGCAGUAAGCGGGUA

>hsa-mir-4520a MI0016886

GUGUGCCACCUGCGUGUUUUCUGUCCAAAUCAGAAAAGGAUUUGGACAGAAAACACGCAGGAAGAAGGAA

>hsa-mir-4521 MI0016887

UCGGCUAAGGAAGUCCUGUGCUCAGUUUUGUAGCAUCAAAACUAGGAUUUCUCUUGUUAC

>hsa-mir-4522 MI0016889

GCGGGCGUUGCCUGGGGGCCUCGCAGGGGGAGAUCCAGCCCAGGCUGGUUCCGCUGACUCUGCCUGUAGGCCGGUGGCGUCUUCUGG

>hsa-mir-4523 MI0016890

GCGGGGGACCGAGAGGGCCUCGGCUGUGUGAGGACUAGAGGCGGCCGAGGCCCGGGCCGGUUCCCCCGA

>hsa-mir-4524a MI0016891

GAACGAUAGCAGCAUGAACCUGUCUCACUGCAGAAUUAUUUUGAGACAGGCUUAUGCUGCUAUCCUUCA

>hsa-mir-4524b MI0019114

UAGCUGGGUGGAUGUGUUCUUUUGAAGGAUAGCAGCAUAAGCCUGUCUCAAAAUAAUUCUGCAGUGAGACAGGUUCAUGCUGCUAUCGUUCCAAAGAGGAAGGGUAAUCACUGUC

>hsa-mir-4525 MI0016892

GUCAGAGGGGGGAUGUGCAUGCUGGUUGGGGUGGGCUGCCUGUGGACCAAUCAGCGUGCACUUCCCCACCCUGAA

>hsa-mir-4526 MI0016893

UGCGGUGACAUCAGGGCCCAGUCCCUGCUGUCAUGCCCCAGGUGACGUGCUGGGCUGACAGCAGGGCUGGCCGCUAACGUCACUGUC

>hsa-mir-4527 MI0016894

CCAGAAGUGGUCUGCAAAGAGAUGACUGUGAAUCCAAGAUCCACAUCAGCUCUGUGCUGCCUACAUCUGA

>hsa-mir-4528 MI0016895

UAUUCUACUGAGAGUACAGAUCUUUAUAUAUAUGAUCAUUAUAUGUAUGAUGAGAUCAUUAUAUGUAUGAUCUGGACACCCAGUAGAAUC

>hsa-mir-4529 MI0016896

AUGACAGGCCAUCAGCAGUCCAAUGAAGACAUGAAGACCCAAUGUCUUCAUUGGACUGCUGAUGGCCCGUCACUGGGA

>hsa-mir-4530 MI0016897

CGACCGCACCCGCCCGAAGCUGGGUCAAGGAGCCCAGCAGGACGGGAGCGCGGCGC

>hsa-mir-4531 MI0016898

GCCUAGGAGUCCUUGGUCAGUGGGGACAUGGAGAAGGCUUCUGAGGA

>hsa-mir-4532 MI0016899

ACAGACCCCGGGGAGCCCGGCGGUGAAGCUCCUGGUAUCCUGGGUGUCUGA

>hsa-mir-4533 MI0016900

UGAGAAUGUGGAAGGAGGUUGCCGGACGCUGCUGGCUGCCUUCCAGCGUCCACUUCCCUUUCUCUCUCUCC

>hsa-mir-4534 MI0016901

UGUGAAUGACCCCCUUCCAGAGCCAAAAUCACCAGGGAUGGAGGAGGGGUCUUGGGUACU

>hsa-mir-4535 MI0016903

AACUGGGUCCCAGUCUUCACAGUUGGUUUCUGACACGUGGACCUGGCUGGGACGAUGUG

>hsa-mir-4536-1 MI0016906

AUGUGGUAGAUAUAUGCACGAUAUAUAUACUGCCCUGCUUUUAUACAUACAUACAUACAUACCUAUAUCGUGCAUAUAUCUACCACAU

>hsa-mir-4536-2 MI0019149

AUGUGGUAGAUAUAUGCACGAUAUAGGUAUGUAUGUAUGUAUGUAUAAAAGCAGGGCAGUAUAUAUAUCGUGCAUAUAUCUACCACAU

>hsa-mir-4537 MI0016908

UGAGCCGAGCUGAGCUUAGCUGGGCUGAGCUAACCAGGGCUGGGCUGAGCUGGGCUGAGCUGAGCUGAGC

>hsa-mir-4538 MI0016909

GAGCUUGGAUGAGCUGGGCUGAACUGGGCUGGGUUGAGCUGGGCUGGGCUGAGUUGAGCCAGGCUGAUCUGGGCUGAG

>hsa-mir-4540 MI0016911

AAGCUGCAUGGACCAGGACUUGGCACCUUUGGCCUUAGUCCUGCCUGUAGGUUUA

>hsa-mir-4632 MI0017259

GAGGGCAGCGUGGGUGUGGCGGAGGCAGGCGUGACCGUUUGCCGCCCUCUCGCUGCUCUAG

>hsa-mir-4633 MI0017260

UGGCAAGUCUCCGCAUAUGCCUGGCUAGCUCCUCCACAAAUGCGUGUGGAGGAGCUAGCCAGGCAUAUGCAGAGCGUCA

>hsa-mir-4634 MI0017261

GGACAAGGGCGGCGCGACCGGCCCGGGGCUCUUGGGCGGCCGCGUUUCCCCUCC

>hsa-mir-4635 MI0017262

CCGGGACUUUGUGGGUUCUGACCCCACUUGGAUCACGCCGACAACACUGGUCUUGAAGUCAGAACCCGCAAAGUCCUGG

>hsa-mir-4636 MI0017263

UAGAUUCAGAACUCGUGUUCAAAGCCUUUAGCCCAGCAAUGGGAGAGUGCUAAAGGCUUCAAGCACGAGUUCUGAAUCUA

>hsa-mir-4637 MI0017264

CCCUUACUUGGAUCUGCAAUUAGUAUUUUAAUCAUAGAUUGUAUUUAGUUAGUUUUUAAUACUAACUGCAGAUUCAAGUGAGGG

>hsa-mir-4638 MI0017265

GACUCGGCUGCGGUGGACAAGUCCGGCUCCAGAACCUGGACACCGCUCAGCCGGCCGCGGCAGGGGUC

>hsa-mir-4639 MI0017266

UUGCUAAGUAGGCUGAGAUUGAUGUCAGGUUAUCCCCAAGCAUAACCUCACUCUCACCUUGCUUUGCAG

>hsa-mir-4640 MI0017267

CUGUGGGCUGGGCCAGGGAGCAGCUGGUGGGUGGGAAGUAAGAUCUGACCUGGACUCCAUCCCACCCACCCCCUGUUUCCUGGCCCACAG

>hsa-mir-4641 MI0017268

GGGGGGCAGGGGGCAGAGGGCAUCAGAGGACAGCCGCCUGGUGCCCAUGCCAUACUUUUGCCUCAG

>hsa-mir-4642 MI0017269

CACAACUGCAUGGCAUCGUCCCCUGGUGGCUGUGGCCUAGGGCAAGCCACAAAGCCACUCAGUGAUGAUGCCAGCAGUUGUG

>hsa-mir-4643 MI0017270

GUGUGCCCUAGCAUUUAUAAUCAUGUGUUCAUUCACAUGAUCAUAAGUGGACACAUGACCAUAAAUGCUAAAGCACAC

>hsa-mir-4644 MI0017271

GCGGCGGUGCUCUGCCUCUUUCUCCAUCCACCCUGGUCCAGGUCCACAGCAGUGGAGAGAGAAAAGAGACAGAAGGAUGGCCGU

>hsa-mir-4645 MI0017272

UGAUAGGGAAACCAGGCAAGAAAUAUUGUCUCCUCAAGUUGCGACGAGACAGUAGUUCUUGCCUGGUUUCUCUAUCA

>hsa-mir-4646 MI0017273

ACUGGGAAGAGGAGCUGAGGGACAUUGCGGAGAGGGUCUCACAUUGUCCCUCUCCCUUCCCAG

>hsa-mir-4647 MI0017274

CCAGGAGGGUGAAGAUGGUGCUGUGCUGAGGAAAGGGGAUGCAGAGCCCUGCCCAGCACCACCACCUCCUAUGCUCCUGG

>hsa-mir-4648 MI0017275

UGUGGGACUGCAAAUGGGAGCUCAGCACCUGCCUGCCACCCACGCAGACCAGCCCCUGCUCUGUUCCCACAG

>hsa-mir-4649 MI0017276

UCUGGGCGAGGGGUGGGCUCUCAGAGGGGCUGGCAGUACUGCUCUGAGGCCUGCCUCUCCCCAG

>hsa-mir-4650-1 MI0017277

UUCUGUAGAGAUUAUCAGGCCUCUUUCUACCUUCCAAGGCUCAGAAGGUAGAAUGAGGCCUGACAUAUCUGCAGGA

>hsa-mir-4651 MI0017279

CGGCGACGGCGGGGUGGGUGAGGUCGGGCCCCAAGACUCGGGGUUUGCCGGGCGCCUCAGUUCACCGCGGCCG

>hsa-mir-4652 MI0017280

UAUUGGACGAGGGGACUGGUUAAUAGAACUAACUAACCAGAACUAUUUUGUUCUGUUAACCCAUCCCCUCAUCUAAUA

>hsa-mir-4653 MI0017281

UUGUCCAAUUCUCUGAGCAAGGCUUAACACCAAAGGGUUAAGGGUUUGCUCUGGAGUUAAGGGUUGCUUGGAGAAUUGGAGAA

>hsa-mir-4654 MI0017282

CUGGCUGGUUGUGGGAUCUGGAGGCAUCUGGGGUUGGAAUGUGACCCCAGUCUCCUUUUCCCUCAUCAUCUGCCAG

>hsa-mir-4655 MI0017283

CCAAGGGCACACCGGGGAUGGCAGAGGGUCGUGGGAAAGUGUUGACCCUCGUCAGGUCCCCGGGGAGCCCCUGG

>hsa-mir-4656 MI0017284

AGGCUGGCGUGGGCUGAGGGCAGGAGGCCUGUGGCCGGUCCCAGGCCUCCUGCUUCCUGGGCUCAGGCUCGGUUU

>hsa-mir-4657 MI0017285

AAUGUGGAAGUGGUCUGAGGCAUAUAGAGUAUAUGCCAAGAACACUACCAUAU

>hsa-mir-4658 MI0017286

GCUGCCCUUCACUCAGAGCAUCUACACCCACUACCGGUGAGUGUGGAUCCUGGAGGAAUCGUGGC

>hsa-mir-4659a MI0017287

GAAACUGCUGAAGCUGCCAUGUCUAAGAAGAAAACUUUGGAGAAAAAUUUUCUUCUUAGACAUGGCAACGUCAACAGUUUC

>hsa-mir-4660 MI0017288

ACUCCUUCUGCAGCUCUGGUGGAAAAUGGAGAAGACUUUUCCUUUCCUCCAUCUCCCCCAGGGCCUGGUGGAGU

>hsa-mir-4661 MI0017289

UUUACUCUGAACUAGCUCUGUGGAUCCUGACAGACAGCCUGAUAGACAGGAUCCACAGAGCUAGUCCAGAGUAAA

>hsa-mir-4662b MI0017293

CACAAUUUCUAUUUAGCCAAUUGUCUAUCUUUAGGCAUUCAGAAUAGCUAAAGAUGGACAAUUGGCUAAAUAGACACUGUG

>hsa-mir-4663 MI0017292

CUGUGGUGGAGCUGAGCUCCAUGGACGUGCAGUGGCAUCUGUCAUUGCUGCCUUCCUGGAGCUCAGGCCCUUGCAG

>hsa-mir-4664 MI0017294

GUUGGGGGCUGGGGUGCCCACUCCGCAAGUUAUCACUGAGCGACUUCCGGUCUGUGAGCCCCGUCCUCCGC

>hsa-mir-4665 MI0017295

CUCGAGGUGCUGGGGGACGCGUGAGCGCGAGCCGCUUCCUCACGGCUCGGCCGCGGCGCGUAGCCCCCGCCACAUCGGG

>hsa-mir-4666a MI0017296

AUCACUUAAAUACAUGUCAGAUUGUAUGCCUACAAAAUCCCUCCAGACUGGCAUACAAUCUGACAUGUAUUUAAGAGAU

>hsa-mir-4666b MI0019299

UGUCUAAAUUGCAUGUCAGAUUGUAAUUCCCAGGCCCUUCCUCCAAUACUGGGAAUUACAAUUUGACAUGCAAUUUAGACA

>hsa-mir-4667 MI0017297

UGACUGGGGAGCAGAAGGAGAACCCAAGAAAAGCUGACUUGGAGGUCCCUCCUUCUGUCCCCACAG

>hsa-mir-4668 MI0017298

AGGGAAAAAAAAAAGGAUUUGUCUUGUAGCCAGGAUAUUGUUUUAAAGAAAAUCCUUUUUGUUUUUCCAG

>hsa-mir-4669 MI0017300

GCCUCCCUUCACUUCCUGGCCAUCCAGGCAUCUGUGUCUGUGUCCGGGAAGUGGAGGAGGGC

>hsa-mir-4670 MI0017301

CUCUAGGAAGCGACCAUGAUGUAACUUCACAGACUCUCCAAAAGUCUGAAGUUACAUCAUGGUCGCUUCCUAGAG

>hsa-mir-4671 MI0017302

UAUUUUAAGACCGAAGACUGUGCGCUAAUCUCUUAGCACUGAAGAUUAGUGCAUAGUCUUUGGUCUCAAAAUA

>hsa-mir-4672 MI0017303

GGCUGCUUCUCGCCUCUGUCCAGCUGUGUGGCCUUGGACAAGCCUCUUGGUUACACAGCUGGACAGAGGCACGAAACAGCC

>hsa-mir-4673 MI0017304

GUCCAGGCAGGAGCCGGACUGGACCUCAGGGAAGAGGCUGACCCGGCCCCUCUUGCGGC

>hsa-mir-4674 MI0017305

CCCAGGCGCCCGCUCCCGACCCACGCCGCGCCGCCGGGUCCCUCCUCCCCGGAGAGGCUGGGCUCGGGACGCGCGGCUCAGCUCGGG

>hsa-mir-4675 MI0017306

CAUGAGAAAUCCUGCUGGUCAACCAUAGCCCUGGUCAGACUCUCCGGGGCUGUGAUUGACCAGCAGGACUUCUCAUG

>hsa-mir-4676 MI0017307

UGAAUGAAAGAGCCAGUGGUGAGACAGUGAGUUGAUUACUUCUCACUGUUUCACCACUGGCUCUUUGGUUCA

>hsa-mir-4677 MI0017308

GCAAAGCAGCAAUUGUUCUUUGGUCUUUCAGCCAUGACCUGACCUUCUGUCUGUGAGACCAAAGAACUACUUUGCUUGGC

>hsa-mir-4678 MI0017309

GGAAAAAACAAGGUAUUGUUCAGACUUAUGAUUUUUGGGGUCAAAGAUUCUGAGCAAUAACCUAUUAAAAAACC

>hsa-mir-4679-2 MI0017311

UAUCUUUUUUCUGUGAUAGAGAUUCUUUGCUUUUUGUUUCUAACAAAGCAAAGAAUCUCUAUCACAGAAAAAAGACG

>hsa-mir-4680 MI0017312

UAUAAGAACUCUUGCAGUCUUAGAUGUUAUAAAAAUAUAUAUCUGAAUUGUAAGAGUUGUUAGCAC

>hsa-mir-4681 MI0017313

GGCAACGGGAAUGCAGGCUGUAUCUGCAGGGCAUUGUGCUAACAGGUGCAGGCUGCAGACCUGUCACAGGCC

>hsa-mir-4682 MI0017314

UGCCCCUGGUCUGAGUUCCUGGAGCCUGGUCUGUCACUGGGGAAGUCCAGAGCUCCAAGGCUCAGUGCCCAGGGGACGCA

>hsa-mir-4683 MI0017315

GACACGCAAGACGAGGCGGGCCUGGAGGUGCACCAGUUCUGGCCGCUGGUGGAGAUCCAGUGCUCGCCCGAUCUCAAGUUC

>hsa-mir-4684 MI0017316

GCACCAGGGGUACCUCUCUACUGACUUGCAACAUACAUUUGUCUUGGUGUGUUGCAAGUCGGUGGAGACGUACCCUUGGUGC

>hsa-mir-4685 MI0017317

UAGCCCAGGGCUUGGAGUGGGGCAAGGUUGUUGGUGAUAUGGCUUCCUCUCCCUUCCUGCCCUGGCUAG

>hsa-mir-4686 MI0017318

GGCUUCCUGUAUCUGCUGGGCUUUCUGGUGUUGGCAGCCCAAGAUGACACCCUGGGCCCAGCAGGAGCCAGAAGCC

>hsa-mir-4687 MI0017319

ACCUGAGGAGCCAGCCCUCCUCCCGCACCCAAACUUGGAGCACUUGACCUUUGGCUGUUGGAGGGGGCAGGCUCGCGGGU

>hsa-mir-4688 MI0017321

GUCUACUCCCAGGGUGCCAAGCUGUUUCGUGUUCCCUCCCUAGGGGAUCCCAGGUAGGGGCAGCAGAGGACCUGGGCCUGGAC

>hsa-mir-4689 MI0017322

GGUUUCUCCUUGAGGAGACAUGGUGGGGGCCGGUCAGGCAGCCCAUGCCAUGUGUCCUCAUGGAGAGGCC

>hsa-mir-4690 MI0017323

GAGCAGGCGAGGCUGGGCUGAACCCGUGGGUGAGGAGUGCAGCCCAGCUGAGGCCUCUGC

>hsa-mir-4691 MI0017324

GGAGCACUCCCAGGUCCUCCAGGCCAUGAGCUGCGGCCCUGAUGUCUCUACUCCAGCCACGGACUGAGAGUGCAUAGGAGUGUCC

>hsa-mir-4692 MI0017325

GUUUUACUUGAUACCCACACUGCCUGGGUGGGACACUCAGGCAGUGUGGGUAUCAGAUAAAAC

>hsa-mir-4693 MI0017326

GUUUAAAGAAUACUGUGAAUUUCACUGUCACAAAUUCAAAUAAAGUGAGAGUGGAAUUCACAGUAUUUAAGGAAU

>hsa-mir-4694 MI0017327

CAAAUACAUAGGUGUUAUCCUAUCCAUUUGCCUCUCUCAGAAAAUAGAGUCAAAUGGACAGGAUAACACCUAUGUAUUUG

>hsa-mir-4695 MI0017328

CCUGCAGGAGGCAGUGGGCGAGCAGGCGGGGCAGCCCAAUGCCAUGGGCCUGAUCUCACCGCUGCCUCCUUCCC

>hsa-mir-4696 MI0017329

CAAAGCCACUGCAAGACGGAUACUGUCAUCUAUUCCAGAAGAUGACAAUGUCCAUUUUGCAGUGGCUUUG

>hsa-mir-4697 MI0017330

GGGCCCAGAAGGGGGCGCAGUCACUGACGUGAAGGGACCACAUCCCGCUUCAUGUCAGUGACUCCUGCCCCUUGGUCU

>hsa-mir-4698 MI0017331

UGCUUCUCCUGGGGUCUUCCUCUACAUUUCCACCUAGACGGGCCUGGGUCAAAAUGUAGAGGAAGACCCCAGAAGGAGCA

>hsa-mir-4699 MI0017332

AGCAAUUGGAGAAGAUUGCAGAGUAAGUUCCUGAUUAAGAAAUGGAAUUUACUCUGCAAUCUUCUCCAAUUGCU

>hsa-mir-4700 MI0017333

UCAGUGAGGUCUGGGGAUGAGGACAGUGUGUCCUGAAAUUCACAGGACUGACUCCUCACCCCAGUGCACGAGGA

>hsa-mir-4701 MI0017334

CCUUGGCCACCACACCUACCCCUUGUGAAUGUCGGGCAAUGGGUGAUGGGUGUGGUGUCCACA

>hsa-mir-4703 MI0017336

UUAUGCAUAUUAGCAAUACAGUACAAAUAUAGUGUGUUUGAUUUGCACUGUAGUUGUAUUGUAUUGCCACUCUGUAUAA

>hsa-mir-4704 MI0017337

CUUAUCCUAGACACUAGGCAUGUGAGUGAUUGUCUUCCUCACUCAAUCAGUCACAUAUCUAGUGUCUAGAAUGAG

>hsa-mir-4705 MI0017338

CUCACAAGAUCAAUCACUUGGUAAUUGCUGUGAUAACAACUCAGCAAUUACCAAGUGAUUGGUUUUGUGAG

>hsa-mir-4706 MI0017339

GCUACGGGGAGCGGGGAGGAAGUGGGCGCUGCUUCUGCGUUAUCUGGAAGGAGCAGCCCACUCCUGUCCUGGGCUCUGUGGU

>hsa-mir-4707 MI0017340

GGUUCCGGAGCCCCGGCGCGGGCGGGUUCUGGGGUGUAGACGCUGCUGGCCAGCCCGCCCCAGCCGAGGUUCUCGGCACC

>hsa-mir-4708 MI0017341

UUUAGGAGAGAGAUGCCGCCUUGCUCCUUGAACAGGAGGAGCAAGGCGGCAUCUCUCUGAUACUAAA

>hsa-mir-4709 MI0017342

CUGCUUCAACAACAGUGACUUGCUCUCCAAUGGUAUCCAGUGAUUCGUUGAAGAGGAGGUGCUCUGUAGCAG

>hsa-mir-4710 MI0017344

GACCGAGUGGGGUGAGGGCAGGUGGUUCUUCCCGAAGCAGCUCUCGCCUCUUCGUC

>hsa-mir-4711 MI0017345

AAAUGUGCAUCAGGCCAGAAGACAUGAGCCCUUUGGAAAGGUCUCGUGUCUUCUGGCUUGAUGCACAUUU

>hsa-mir-4712 MI0017346

GACAGGAUUCCAGUACAGGUCUCUCAUUUCCUUCAUGAUUAGGAAUACUACUUUGAAAUGAGAGACCUGUACUGUAUCUGUU

>hsa-mir-4713 MI0017347

GUCCCCAUUUUUCUCCCACUACCAGGCUCCCAUAAGGGUCGAAUGGGAUCCAGACAGUGGGAGAAAAAUGGGGAC

>hsa-mir-4714 MI0017348

AUUUUGGCCAACUCUGACCCCUUAGGUUGAUGUCAGAAUGAGGUGUACCAACCUAGGUGGUCAGAGUUGGCCAAAAU

>hsa-mir-4715 MI0017349

GGGGAAUGAAAGUUGGCUGCAGUUAAGGUGGCUAAUCAGCUGAUGGUGCCACCUUAACUGCAGCCAAUUCUAAUUCCCC

>hsa-mir-4716 MI0017350

CAUACUUUGUCUCCAUGUUUCCUUCCCCCUUCUGUAUACAUGUAUACAGGAGGAAGGGGGAAGGAAACAUGGAGACAAAGUGUG

>hsa-mir-4717 MI0017352

GGCAGUGUUUAGGCCACAGCCACCCAUGUGUAGGGGUGGCUACACAUGGGUGGCUGUGGCCUAAACACUGCC

>hsa-mir-4718 MI0017353

AGCUGUACCUGAAACCAAGCACCUGUUUGUGACUUGGCUUCAGUUACUAGC

>hsa-mir-4719 MI0017354

ACAAUGAUGACUUGUAUGUUAUAGAUUUGUGAUUACAUUAAAACUUAAAAUUUCACAAAUCUAUAAUAUGCAGGUCAUCACUGU

>hsa-mir-4720 MI0017355

AAGCCUGGCAUAUUUGGUAUAACUUAAGCACCAGGUAAAAUCUGGUGCUUAAGUUGUACCAAGUAUAGCCAAGUUU

>hsa-mir-4721 MI0017356

GGGCCUGGUCAUGGUCAAGCCAGGUUCCAUCAAGCCCCACCAGAAGGUGGAGGCCCAGGUGAGGGCUCCAGGUGACGGUGGGCAGGGUU

>hsa-mir-4722 MI0017357

GGCAGGAGGGCUGUGCCAGGUUGGCUGGGCCAGGCCUGACCUGCCAGCACCUCCCUGCAG

>hsa-mir-4723 MI0017359

AGUUGGUGGGGGAGCCAUGAGAUAAGAGCACCUCCUAGAGAAUGUUGAACUAAAGGUGCCCUCUCUGGCUCCUCCCCAAAG

>hsa-mir-4724 MI0017361

ACGCAAAAUGAACUGAACCAGGAGUGAGCUUCGUGUACAUUAUCUAUUAGAAAAUGAAGUACCUUCUGGUUCAGCUAGUCCCUGUGCGU

>hsa-mir-4725 MI0017362

GUGUCUCUCUGGAGACCCUGCAGCCUUCCCACCCACCAGGGAGCUUUCCAUGGGCUGUGGGGAAGGCGUCAGUGUCGGGUGAGGGAACAC

>hsa-mir-4726 MI0017363

AGGGCCAGAGGAGCCUGGAGUGGUCGGGUCGACUGAACCCAGGUUCCCUCUGGCCGCA

>hsa-mir-4727 MI0017364

AAUCUGCCAGCUUCCACAGUGGCAGAUUUUCCCAUAGUGGGAAGCUGGCAGAUUC

>hsa-mir-4728 MI0017365

GUGGGAGGGGAGAGGCAGCAAGCACACAGGGCCUGGGACUAGCAUGCUGACCUCCCUCCUGCCCCAG

>hsa-mir-4729 MI0017366

UCUGUUUCCUCAUUUAUCUGUUGGGAAGCUAACUGUGACCUUAGCGUCCCAGCAGAUAAAUGAGGAAACAGA

>hsa-mir-4730 MI0017367

CGCAGGCCUCUGGCGGAGCCCAUUCCAUGCCAGAUGCUGAGCGAUGGCUGGUGUGUGCUGCUCCACAGGCCUGGUG

>hsa-mir-4731 MI0017368

CCCUGCCAGUGCUGGGGGCCACAUGAGUGUGCAGUCAUCCACACACAAGUGGCCCCCAACACUGGCAGGG

>hsa-mir-4732 MI0017369

GAGGGAGCUGUAGAGCAGGGAGCAGGAAGCUGUGUGUGUCCAGCCCUGACCUGUCCUGUUCUGCCCCCAGCCCCUC

>hsa-mir-4733 MI0017370

GGUCGCUUAAAUCCCAAUGCUAGACCCGGUGGCAAUCAAGGUCUAGCCACCAGGUCUAGCAUUGGGAUUUAAGCCC

>hsa-mir-4734 MI0017371

CUCGGGCCCGACCGCGCCGGCCCGCACCUCCCGGCCCGGAGCUGCGGGCUGCGGUCAGGGCGAUCCCGGG

>hsa-mir-4735 MI0017372

UGCAGUGCCUAAUUUGAACACCUUCGGUAUUCAUCAAAAAUACCAAAGGUGCUCAAAUUAGACAUUGCA

>hsa-mir-4736 MI0017373

AGGCAGGUUAUCUGGGCUGCCAUCUCCCACUGGCUGCUUGCCUGCCU

>hsa-mir-4737 MI0017374

CUGCACAGGAUGCGAGGAUGCUGACAGUGCCUCACAGCCGCACAGGACCGAGGAUGCUGACGGUGCCUCACAGCCACACAG

>hsa-mir-4738 MI0017376

GGUCGCAUUUCUCCUUCUUACCAGCGCGUUUUCAGUUUCAUAGGGAAGCCUUUCCAUGAAACUGGAGCGCCUGGAGGAGAAGGGGCC

>hsa-mir-4739 MI0017377

GGGAGGAAGAAGGGAGGAGGAGCGGAGGGGCCCUUGUCUUCCCAGAGCCUCUCCCUUCCUCCCCUCCCCCUCCC

>hsa-mir-4740 MI0017378

GCCAAGGACUGAUCCUCUCGGGCAGGGAGUCAGAGGGGACCGCCCGAGAGGAUCCGUCCCUGC

>hsa-mir-4741 MI0017379

CGGGCGGGGCGGGUCCGGCCGCCUCCGAGCCCGGCCGGCAGCCCCCGGCCUUAAAGCGCGGGCUGUCCGGAGGGGUCGGCUUUCCCACCG

>hsa-mir-4742 MI0017380

UCAGGCAAAGGGAUAUUUACAGAUACUUUUUAAAAUUUGUUUGAGUUGAGGCAGAUUAAAUAUCUGUAUUCUCCUUUGCCUGCAG

>hsa-mir-4743 MI0017381

GCUGGCCGGAUGGGACAGGAGGCAUGAAUGAGCCAUCUUUCCAAUGCCUUUCUGUCUUUUCUGGUCCAG

>hsa-mir-4744 MI0017382

GUAAUCACAUCUAAAGACUAGACUUCGCUAUGACCAGGCCAUAGUAAACAUCAUAGUAUGUCUAGUCUUUAGGUUUGAUUAC

>hsa-mir-4745 MI0017384

GUGAGUGGGGCUCCCGGGACGGCGCCCGCCCUGGCCCUGGCCCGGCGACGUCUCACGGUCCC

>hsa-mir-4746 MI0017385

GUGUCUGUGCCGGUCCCAGGAGAACCUGCAGAGGCAUCGGGUCAGCGGUGCUCCUGCGGGCCGACACUCAC

>hsa-mir-4747 MI0017386

AGGGAAGGAGGCUUGGUCUUAGCACGGGGUCUAAGGCCCGGGCUUUCCUCCCAG

>hsa-mir-4748 MI0017387

UGGCUGGCUGAGGUUUGGGGAGGAUUUGCUGGUGCUAGAGAGGAAAGCAGACCCUACCCAACCCCACGCCCUACUACAGCCA

>hsa-mir-4749 MI0017388

CCUGCGGGGACAGGCCAGGGCAUCUAGGCUGUGCACAGUGACGCCCCUCCUGCCCCCACAG

>hsa-mir-4750 MI0017389

CGCUCGGGCGGAGGUGGUUGAGUGCCGACUGGCGCCUGACCCACCCCCUCCCGCAG

>hsa-mir-4751 MI0017390

CCCGGAGCCAGAGGACCCGUAGCUGCUAGAAGGGCAGGGGUGUGGCUUCUGGGGGCUGGUCUUCAGCUCUGGCG

>hsa-mir-4752 MI0017391

AGUGUCUCCUUGUGGAUCUCAAGGAUGUGCUUCCACAUAGCAGCAUGUUCUUCAGAUGGACAAGGAGACACU

>hsa-mir-4753 MI0017392

AUAUCUACACAAGGCCAAAGGAAGAGAACAGAUAUAUCCACAGUACACUUGGCUGUUCUCUUUCUUUAGCCUUGUGUAGAUAU

>hsa-mir-4754 MI0017394

ACGCGCCUGAUGCGGACCUGGGUUAGCGGAGUGAGGCCCAGUGGUCACCGCCGCCCUCCGCAGGUCCAGGUUGCCGUGCGCAUGUGCCU

>hsa-mir-4755 MI0017395

AGAUUCAGCUUUCCCUUCAGAGCCUGGCUUUGGCAUCUAUGAAAGCCAGGCUCUGAAGGGAAAGUUGAAUCU

>hsa-mir-4756 MI0017397

GGGAUAAAAUGCAGGGAGGCGCUCACUCUCUGCUGCCGAUUCUGCACCAGAGAUGGUUGCCUUCCUAUAUUUUGUGUC

>hsa-mir-4757 MI0017398

UUCCAGCCCGAGGCCUCUGUGACGUCACGGUGUCUGCGGGAGGAGACCAUGACGUCACAGAGGCUUCGCGCUCUGAG

>hsa-mir-4758 MI0017399

GGUGAGUGGGAGCCGGUGGGGCUGGAGUAAGGGCACGCCCGGGGCUGCCCCACCUGCUGACCACCCUCCCC

>hsa-mir-4759 MI0017400

CAUUUAGGACUAGAUGUUGGAAUUAGACAGAAAAAAGUUAGACACAAAAAAUUGUGUCUAAUUCCAACAUCUAGUCCUAAAUG

>hsa-mir-4760 MI0017401

GCCAUGGUGUUUAGAUUGAACAUGAAGUUAGAAUUCUUAAGUAUCAAAACUAAAUUCAUGUUCAAUCUAAACCCCAUGGC

>hsa-mir-4761 MI0017402

GGACAAGGUGUGCAUGCCUGACCCGUUGUCAGACCUGGAAAAAGGGCCGGCUGUGGGCAGGGAGGGCAUGCGCACUUUGUCC

>hsa-mir-4762 MI0017403

CUGAUACCCCAAAUCUUGAUCAGAAGCCUUGAUCAGAAGCUAGGAAGGCUUCUGAUCAAGAUUUGUGGUGUCAAG

>hsa-mir-4763 MI0017404

CCUGUCCCUCCUGCCCUGCGCCUGCCCAGCCCUCCUGCUCUGGUGACUGAGGACCGCCAGGCAGGGGCUGGUGCUGGGCGGGGGGCGGCGGG

>hsa-mir-4764 MI0017405

UCUUCCCCAUGGAUGUGGAAGGAGUUAUCUGUCACCAGUCAGAUAACUGUCACCAGUCAGUUAACUCCUUUCACACCCAUGGGGAAGA

>hsa-mir-4765 MI0017406

UGGUGAUUUUGAACGUAGCUAUCCACCACUCAGCCUGGAAAAAGCUGAGUGAUUGAUAGCUAUGUUCAAAAUCACCA

>hsa-mir-4766 MI0017407

CUGAAGCUCCUUCUGAAAGAGCAGUUGGUGUUUAUUUUUUACUAAAUAGCAAUUGCUCUUUUGGAAGGAACUUGAG

>hsa-mir-4767 MI0017408

ACAUGGGCCCGCGGGCGCUCCUGGCCGCCGCCCGACUUCGGGGCCAGCCGGGGGCAGAGCGCGCGGGAGCCCGAGCGU

>hsa-mir-4768 MI0017409

AAACUUUGAUUCUCUCUGGAUCCCAUGGAUAUGGGAACUGUGAUGUCCAGGAGAUCCAGAGAGAAUCAGAGUUU

>hsa-mir-4769 MI0017410

GAGGAGAGGUGGGAUGGAGAGAAGGUAUGAGCUAAAAAUCCCCAAGCUCUGCCAUCCUCCCUCCCCUACUUCUCCCC

>hsa-mir-4770 MI0017411

GAGUUAUGGGGUCAUCUAUCCUUCCCUUGGAAAAUGAUCUGAGAUGACACUGUAGCUC

>hsa-mir-4771-1 MI0017412

GCUCUAGCCUAAUUUUAGAUCUGGUCUGCUUCAGUUUCACUCCAAGCAGACUUGACCUACAAUUAGCCUAGAGC

>hsa-mir-4772 MI0017414

GUGAUUGCCUCUGAUCAGGCAAAAUUGCAGACUGUCUUCCCAAAUAGCCUGCAACUUUGCCUGAUCAGAGGCAGUCAC

>hsa-mir-4773-1 MI0017415

UGCUCCCCAGCCUUUCUAUGCUCCUGUUCUGCUUUAUUUCAUCAAAGCAGAACAGGAGCAUAGAAAGGCUGGGGAGCA

>hsa-mir-4774 MI0017417

UAUAUUGUUGUCUGGUAUGUAGUAGGUAAUAACUGACAAACAGACAAUUGCCUAACAUGUGCCAGAAAACAACAUA

>hsa-mir-4775 MI0017418

AUUAAGCUUUUAAUUUUUUGUUUCGGUCACUCUUGAUAGCAGACAUUGACUGAAACAAAAAAUUAAAAGCUUUAU

>hsa-mir-4776-1 MI0017419

CUAUAUGCAGUGGACCAGGAUGGCAAGGGCUCUCCUGAAAGGACAGUAGAGCCCUUGCCAUCCUGGUCCACUGCAUAUAG

>hsa-mir-4777 MI0017421

UAGAAUAUUUCGGCAUUCUAGAUGAGAGAUAUAUAUAUACCUCAUAUGUAUAUGGUAUACCUCAUCUAGAAUGCUGUAAUAUUCUA

>hsa-mir-4778 MI0017422

UCACAUGUCCAAUUCUGUAAAGGAAGAAGAGGUAAGAAGAAGUGAAGCCCUCUUCUUCCUUUGCAGAGUUGAAUAUGUGG

>hsa-mir-4779 MI0017423

UAAAUGUCUUACUGCUUUUACUGUUCCCUCCUAGAGUCCAUUCUUUACUCUAGGAGGGAAUAGUAAAAGCAGUAAGACAUUUA

>hsa-mir-4780 MI0017424

GGCCAGUGCCAGGGGGUCAGGCUCAAGGACCAGCCCAAAGGCCAGGCCUGACCCUUGAGCCUGAUCCCUAGCACUGAUCCC

>hsa-mir-4781 MI0017426

AGGUGCACGCUCUAGCGGGGAUUCCAAUAUUGGGCCAAUUCCCCCAAUGUUGGAAUCCUCGCUAGAGCGUGCACUU

>hsa-mir-4782 MI0017427

AUUGCCCAGUUCUGGAUAUGAAGACAAUCAAGAAAAGAUUUGGUGUUCUUGAUUGUCUUCAUAUCUAGAACUGGGCAGU

>hsa-mir-4783 MI0017428

GGGAAAGCGGAGGGCGCGCCCAGCUCCCGGGCUGAUUGCGCUAACAGUGGCCCCGGUGUUGGGGCGCGUCUGCCGCUGCCCC

>hsa-mir-4784 MI0017429

UGACUGGGCUGAGGAGAUGCUGGGACUGAGAGUGUCAUGGUGGAGCCUCCGUCCCUGCUCAUCCUCUCCGCAUGUUG

>hsa-mir-4785 MI0017430

GUAGGUGGGGACGCGGCGGCGCUGCUCCUCCGCUGCCGCCGGGAGAGUCGGCGACGCCGCCAGCUCCGCGCGC

>hsa-mir-4786 MI0017433

GGGCAUGGCCUGAGACCAGGACUGGAUGCACCACUCUCCCUGUGAUGAGGUGAAGCCAGCUCUGGUCUGGGCCAUUUCAC

>hsa-mir-4787 MI0017434

CGGUCCAGACGUGGCGGGGGUGGCGGCGGCAUCCCGGACGGCCUGUGAGGGAUGCGCCGCCCACUGCCCCGCGCCGCCUGACCG

>hsa-mir-4788 MI0017435

AAUGAAGGAUUACGGACCAGCUAAGGGAGGCAUUAGGAUCCUUAUUCUUGCCUCCCUUAGUUGGUCCCUAAUCCUUCGUU

>hsa-mir-4789 MI0017436

CAUGCUACGUAUGUAUACACCUGAUAUGUGUAUGUGUAAAUACAUAUCCACACACAUAGCAGGUGUAUAUAUAGGUAGCCUG

>hsa-mir-4790 MI0017437

CAAUGUGACAUCGCUUUACCAUUCAUGUUCACUGAAAGGUAGAUUUUAAAAACAUGAAUGGUAAAGCGAUGUCACAUUG

>hsa-mir-4791 MI0017438

UAAGAACUGGAUAUGAUGACUGAAAUAAGCUCCAUAUCAAUGAGAAUUUCAAUGGGAUUAUGUGCAGUCAAUGUCCAGUAAUUA

>hsa-mir-4792 MI0017439

GCAGCCCGGUGAGCGCUCGCUGGCCUGGCAGUGCGUCGGAAGAACAGGGCGGGUGGGGCCGCGCACAUCUCUGC

>hsa-mir-4793 MI0017440

UUUCUCCUCGCUGCCCGCACAUCCUGCUCCACAGGGCAGAGGGAGGCCAAGAAGACCUCUGCACUGUGAGUUGGCUGGCUGGAGGAA

>hsa-mir-4794 MI0017441

UUUUAACAUCUGGCUAUCUCACGAGACUGUAUGUCCUAACAGUGCUUGUAGUCUCAUGAGAUAGCCAGAUGUUAAAA

>hsa-mir-4795 MI0017442

UGAUAUGGAAGAAAUCCAGAAGUGGCUAAUAAUAUUGACACUAUAACAAUAAUGUCAAUAUUAUUAGCCACUUCUGGAUUUAUGAAUCA

>hsa-mir-4796 MI0017443

UAAAUUUGUGUCUAUACUCUGUCACUUUACUUUUGGCCUCAAGUCAUUGCAGUAAAGUGGCAGAGUAUAGACACAAAUUUA

>hsa-mir-4797 MI0017444

GACUCAGAAGACAGAGUGCCACUUACUGAAAGGUUUUUUCUCUCAGUAAGUGGCACUCUGUCUUCUGAGUU

>hsa-mir-4798 MI0017445

AAGUACAACUUCGGUAUACUUUGUGAAUUGGCUUUUACAAAAGACCAACUCACGAAGUAUACCGAAGUCAUACUU

>hsa-mir-4799 MI0017446

ACUGCUAAUAUCUAAAUGCAGCAUGCCAGUCCUGAGAUGCAGGGACUGGCAUGCUGCAUUUAUAUAUUAGCAGU

>hsa-mir-4800 MI0017448

GGAGAAAGGAGUGGACCGAGGAAGGAAGGAAGGCAAGGCUGUCUGUCCAUCCGUCCGUCUGUCCACCUACCUGUCAGUCC

>hsa-mir-4801 MI0017449

UUGAGGCUUGGUUUUCUUAUGUGUAAAAUGUAAUAACAUUUCUUAUGUUUAAAACACUUUACACAAGAAAACCAAGGCUCAA

>hsa-mir-4802 MI0017450

CUGACUGGCUUGUAUGGAGGUUCUAGACCAUGUUAGUGUUCAAGUCUACAUGGAUGGAAACCUUCAAGCAGGCCAAGCAG

>hsa-mir-4803 MI0017451

AGUGGGAUUUAACAUAAUAGUGUGGAUUGAAUCACACACACAUUUCAACCCACACUAUGAUGUUAAAUCCCAUU

>hsa-mir-4804 MI0017452

UCAGUGUAUUUGGACGGUAAGGUUAAGCAAGGUGCGUCGUAUCUUGCUUAACCUUGCCCUCGAAAUACACUGA

>hsa-mir-4999 MI0017865

AUAGAAAAUAAAACACAUACUGCUGUAUUGUCAGGUAGUGAUAGGAUUUAUCACUACCUGACAAUACAGUAUGUGUUUGUUUUAUAUAUUU

>hsa-mir-5000 MI0017866

CUGAAGAGUAGAGUGUGUGGUCCCAGUUCAGAAGUGUUCCUGAGUAACUUGUGCUUAUAACUCAGGACACUUCUGAACUUGGACCAUACAGGUCUCCCUGCUU

>hsa-mir-5001 MI0017867

AGCUCAGGGCGGCUGCGCAGAGGGCUGGACUCAGCGGCGGAGCUGGCUGCUGGCCUCAGUUCUGCCUCUGUCCAGGUCCUUGUGACCCGCCCGCUCUCCU

>hsa-mir-5002 MI0017868

UCUUCCUCUCUGUCCUCUGGAAUUUGGUUUCUGAGGCACUUAGUAGGUGAUAGCAUGACUGACUGCCUCACUGACCACUUCCAGAUGAGGGUUACUC

>hsa-mir-5003 MI0017869

AUGAGUUUGCUUUGUGUCAUCCUCACAACAACCUUGCAGGGUAGAGAUGAUUUUUCCUACUUUUCUAGGUUGUUGGGGGCUGGGGCAGGGGGAACAGAG

>hsa-mir-5004 MI0017870

GGCACUUGCUUGGGGGUUAGUGAGGACAGGGCAAAUUCACGAGAUUGGGUUGUGCAGAGGCUGACACUUGGAUUUUCCUGGGCCUCAGGACUUCCUUUCAGACAUGG

>hsa-mir-5006 MI0017873

AACCAUUAGGGGGCUGUGGUUUGCCAGGGCAGGAGGUGGAAGGGAGCCCCAUUUACAGUGGUAACUUCCUUUCCCUUUCCAUCCUGGCAGGCUUCAGAGAACUUUACCAG

>hsa-mir-5007 MI0017874

GGUAAACCUUGGUGACUAAUUAGAGUCUGGCUGAUAUGGUUUGACACAGAGCUAAAUCAUAUGAACCAAACUCUAAUUAGUCAAUAAUUUCUGUU

>hsa-mir-5008 MI0017876

GGGCUGACCCCUAGGGUCAGGUGAGGCCCUUGGGGCACAGUGGUGCCAUCUCCCCUGUGCUCCCAGGGCCUCGCCUGUCCCUUGAGGUCGGCCC

>hsa-mir-5009 MI0017877

GACCAGAAGUGUUUUGGAUUUUGGACUUUUUCAGAUUUGGGGAUAUUUGCAUUAUACUUAUCCUAAAUCUGAAAGUCCAAAACCUGAAAUGACCAAUAAG

>hsa-mir-5010 MI0017878

GAUCCAGGGAACCCUAGAGCAGGGGGAUGGCAGAGCAAAAUUCAUGGCCUACAGCUGCCUCUUGCCAAACUGCACUGGAUUUUGUGUCUCCCAUUCCCCAGAGCUGUCUGAGGUGCUUUG

>hsa-mir-5011 MI0017879

AGAUGGUAUUGAGUGGAUGCUGUUAUAUAUACAGCCAUGCACUCUGUAGUUUGGGUACACAGUGCAUGGCUGUAUAUAUAACACUAUCCAUUCAUCUUUCAGC

>hsa-mir-5047 MI0017932

GAAGCGCUUGCCUAGACGAGACACAGUGCAUAAAAACAACUUUUGGGGGACAGGUAUGUUUUCUUGCAGCUGCGGUUGUAAGGUCUUGGCAAGACAAGCA

>hsa-mir-5087 MI0017976

AGCUUUCUACGGGUUUGUAGCUUUGCUGGCAUGUUAAGUGUUGUCCUACAGUCGCAAGCAUAAGAAAGAGAAAGUA

>hsa-mir-5088 MI0017977

CCCAUCAGGGCUCAGGGAUUGGAUGGAGGUGAUGGGGGCAGGGGAUGGGUCUCACCCUCCCUUCUUCCUGGGCCCUCAG

>hsa-mir-5089 MI0017978

AAGGACUUCAGUGGGAUUUCUGAGUAGCAUCCUUGGAAUCUGCACUCAAGGGAUGCUACUCGGAAAUCCCACUGAAGUCCUUUU

>hsa-mir-5090 MI0017979

UCUGAGGUACCCGGGGCAGAUUGGUGUAGGGUGCAAAGCCUGCCCGCCCCCUAAGCCUUCUGCCCCCAACUCCAGCCUGUCAGGA

>hsa-mir-5091 MI0017980

GACUGUGGCGACGGAGACGACAAGACUGUGCUGGUCGCGGGUUGUGGGGUUUAGGUCACCGGCAGGGGUCUGGAGUCCCUGGAGGUUAGGGCU

>hsa-mir-5092 MI0017981

AUCCCAGAUCAGAUGCCAAAGCCAGUGGGGACUGGACAACAUGAUGAGCCCAAACCAAUCCACGCUGAGCUUGGCAUCUGAUUUGGGA

>hsa-mir-5093 MI0017982

CCCGCCAGGUCCACAUGCCAGAGUGUCAACGUGACCCAGCCAGCCUCCUUCCUGAGCUAGGAGGAUUAGGAAAUGAGGCUGGCUAGGAGCACAGCCAGGG

>hsa-mir-5094 MI0017983

AAAAGAAAAAAAUCAGUGAAUGCCUUGAACCUAACACACUGCCUUUUAUGUGGUAGGUACAGUGGGCUCACUGAAACAUUCAACU

>hsa-mir-5095 MI0018001

CUGGGAUUACAGGCGUGAACCACCGCGCCCGGCCUAACUUUUAAGAAACGUCGGCCCGGGAGCGGUGGCUCACGCCUGUAAUCCCAGC

>hsa-mir-5096 MI0018004

AGUAGAGGUGGGGUUUCACCAUGUUGGUCAGGCUGGUCUCAAACUCCUGACCUCAGGUGAUCCAUCCACC

>hsa-mir-5100 MI0019116

CCAUGAGGAGCUGGCAGUGGGAUGGCCUGGGGGUAGGAGCGUGGCUUCUGGAGCUAGACCACAUGGGUUCAGAUCCCAGCGGUGCCUCUAACUGGCCACAGGACCUUGGGCAGUCAGCU

>hsa-mir-5186 MI0018165

UCAGCCAGCUUAUGACUUGACCCUCUCACCUGAUUUCUACCAACCUUUCCUCAGCUGAUUUCUUUCUGGGGAGAGAUUGGUAGAAAUCAGGUGAGAGGGUCAUGCCAUAAGCUGGCUAAC

>hsa-mir-5187 MI0018166

GACUAAGGGUGGGAUGAGGGAUUGAAGUGGAGCAGGAAUGCGCUUUUCUCCACUGAAUCCUCUUUUCCUCAGGUGG

>hsa-mir-5188 MI0018167

GGGAGGCAUGGAAAUUUCUCUGGUUUCAAUGGGUACGAUUAUUGUAAGCAGGAUCCAUUCAAUAAUCGGACCCAUUUAAACCGGAGAUUUUAAAAGACAGGAAUAGAAUCCCA

>hsa-mir-5189 MI0018168

GGCCCGCCUUUUAGGGGCCUCGCUGUCUGGGCACAGGCGGAUGGACAGGCUGGCCUCUGGAUGACCUGCCAACCGUCAGAGCCCAGACCCACGUGGCCUCAGUUGGGGACCAGG

>hsa-mir-5190 MI0018169

GGUCAUACCCUGGCUCCAGCCCUGUCACAUGGUUAAUGUUCCACAGCCAGUGACUGAGCUGGAGCCAGGGCCACUGCCCC

>hsa-mir-5191 MI0018170

AGUUGGCCAGGACCCCAAGCCCCCAGCACUUCAUUCUUGCUGUCCUCUCCUGGUCUGGGAGGAUAGAAGAGAGGAUAGGAAGAAUGAAGUGCUGGGCGCUUAGGGGGAUCCUGGCCAACU

>hsa-mir-5192 MI0018171

UUAGUUCCAGCCUCCUGGCUCACCUGGAACCAUUUCUCCUGGGAAGCAUGGUAGCCAGGAGAGUGGAUUCCAGGUGGUGAGGGCUUGGUACU

>hsa-mir-5193 MI0018172

CCUAGGAAAGGCUGCUGGUAACUGGGAUGGGGGUUGGGGGGAGGUAAGAAGUCUCUGACUCCUCCUCUACCUCAUCCCAGUUCCAUCACCUGAAGUGGACCUCUUGGGA

>hsa-mir-5194 MI0018173

AUUUCUUUGGGUUAACUUAAACUCAGCCCUUCUAGGCCCAUUCUUUUCACUCAGGAAUUGGAUAAGCUUUUCUGAGGGGUUUGGAAUGGGAUGGCAGGGAGAGUCACCAGACACCAUGAA

>hsa-mir-5195 MI0018174

GAGCAAAAACCAGAGAACAACAUGGGAGCGUUCCUAACCCCUAAGGCAACUGGAUGGGAGACCUGACCCAUCCAGUUCUCUGAGGGGGCUCUUGUGUGUUCUACAAGGUUGUUCA

>hsa-mir-5196 MI0018175

UCUGAGGAGACCUGGGCUGUCAGAGGCCAGGGAAGGGGACGAGGGUUGGGGAACAGGUGGUUAGCACUUCAUCCUCGUCUCCCUCCCAGGUUAGAAGGGCCCCCCUCUCUGAAGG

>hsa-mir-5197 MI0018176

UAUGGGAUUCCACAGACAAUGAGUAUCAAUGGCACAAACUCAUUCUUGAAUUUUUGCCAGUUCAAGAAGAGACUGAGUCAUCGAAUGCUCUAAAUGUCACUUCACCUCAUGU

>hsa-mir-5571 MI0019115

AUCUGACACAAAAUGUGAACCAAGCAAUUCUCAAAGGAGCCUCCCAGGAAAUUCACUUUAGGAAGUCCUAGGAGGCUCCUCUGAGAGUUGCUAAAACAAAACAUUGAGAGUCC

>hsa-mir-5572 MI0019117

AGCCAGACAAGAGGGUCAUGGGGAGUCACUGUCAACCCAGAGCAGGCACUGCCCCUGCGACCAGCCUGGGGCAUCGGUUGGGGUGCAGGGGUCUGCUGGUGAUGCUUUCCAUCUCUUUGCUUUGUCCUGAUUGUAGC

>hsa-mir-5579 MI0019133

UAUGGUACUCCUUAAGCUAACAGGCCCCUGUCACCAUUAGCUUAAGGAGUACCAGAUC

>hsa-mir-5580 MI0019135

UGCUGGCUCAUUUCAUAUGUGUGCUGAGAAAAUUCACACAUAUGAAGUGAGCCAGCAC

>hsa-mir-5581 MI0019136

AGCCUUCCAGGAGAAAUGGAGACCCUAUACAUACCUGUUUCCAUGCCUCCUAGAAGUUCC

>hsa-mir-5582 MI0019138

UAGGCACACUUAAAGUUAUAGCUACAUCAGUUAUAACUAUAUCAGUUAAAACUUUAAGUGUGCCUAGG

>hsa-mir-5583-1 MI0019139

AAACUAAUAUACCCAUAUUCUGGCUAGGUGAUCAUCAGAAUAUGGGUAUAUUAGUUUGG

>hsa-mir-5584 MI0019141

CAGGGAAAUGGGAAGAACUAGAUUUGAAUCCAGACCUUUAGUUCUUCCCUUUGCCCAAUU

>hsa-mir-5585 MI0019142

UGAAGUACCAGCUACUCGAGAGGUCAGAGGAUUGCUCCUGAAUAGCUGGGACUACAGGU

>hsa-mir-5586 MI0019143

UAUCCAGCUUGUUACUAUAUGCUUUUUAAAUGGGGCACAGAGUGACAAGCUGGUUAAAG

>hsa-mir-5587 MI0019144

AUGGUCACCUCCGGGACUCAGCCCUGUGCUGAGCCCCGGGCAGUGUGAUCAUC

>hsa-mir-5588 MI0019147

ACUGGCAUUAGUGGGACUUUUUUUUUUUUUUUUUUUAAUGUUAAAAGUCCCACUAAUGCCAGC

>hsa-mir-5589 MI0019148

GGCUGGGUGCUCUUGUGCAGUGAGCAACCUACACAACUGCACAUGGCAACCUAGCUCCCA

>hsa-mir-5590 MI0019150

UUGCCAUACAUAGACUUUAUUGUGUUGAUCAACAAUAAAGUUCAUGUAUGGCAA

>hsa-mir-5591 MI0019151

UGGGAGCUAAGCUAUGGGUAUACUGAGCUUAUGUAUGCAUCUGCAUACCCAUAGCUUAGCUCCCA

>hsa-mir-5680 MI0019280

GCAUUGGGUUAGCAGGUUAGCCCAGCAUUUCCCUUCCUGGACACACAGGAGGAGAAAUGCUGGACUAAUCUGCUAAUCCAAUGC

>hsa-mir-5681a MI0019281

AGUUUUUGAAGAGUAUUGCCACCCUUUCUAGUCCCUAUUAGACUAGAAAGGGUGGCAAUACCUCUUCCAAAAACU

>hsa-mir-5682 MI0019282

GGCCCAUGGGUCUUAUCCUGCAAGGUGCUGCAGAGACGAGGCCUGUAGCACCUUGCAGGAUAAGGUCUACUGGGCC

>hsa-mir-5683 MI0019284

GGAGCUUGUUACAGAUGCAGAUUCUCUGACUUCUUACUGCACCAGUGAAGUCAGGAUCUGCAUUUGAAUAAGACCC

>hsa-mir-5684 MI0019285

GCUGAACUCUAGCCUGAGCAACAGAGUGAGAUGGUCUUGUUUUGUUGCCCAGGCUGGAGUCCAGU

>hsa-mir-5685 MI0019287

CUCUACAUCACAGCCCAGCAGUUAUCACGGGCCCCUCCCCUCAAUGGGCCCGUGAUAACUGCAGGGCUGUGAUGUAGAG

>hsa-mir-5687 MI0019291

CCUCACUUAUCUGACUCUGAAAUCUUCUAAAUGGUACCCACUUUAUUUAGAACGUUUUAGGGUCAAAUAAGUACAGG

>hsa-mir-5688 MI0019292

GAAACACUUUGCCUUUUUACAGGAGUUUAUUAUGUUUUGGACAUAGAAACAUAACAAACACCUGUAAAACAGCAAAGUGUUUC

>hsa-mir-5689 MI0019294

AGCGUGGUAGCAUACACCUGUAGUCCUAGAUACUCAGGAGGGUGAGUAUCUAGGACUACAGGUGUGUGCUACCACGCU

>hsa-mir-5690 MI0019295

CUUUUAAUUUCAGCUACUACCUCUAUUAGGAUUUGGGAGUUAUACUAAUAGAGGUAAUAGUUGAAAUUAAGAG

>hsa-mir-5691 MI0019296

GGACAAGCUUGCUCUGAGCUCCGAGAAAGCUGACAGACAGCUGCUUGGUGUUCAGAGCUUGUCUGUCC

>hsa-mir-5692a-2 MI0019298

UACAAAUAAUACCACAGUGGGUGUACCUCAUGUGUGUACACCCUGUGAUAUUAUUUGUA

>hsa-mir-5692b MI0019311

GAUAUUAUGAAUAAUAUCACAGUAGGUGUUCACACAUAAUGUGUACACCAUGUGUGUACACCCAUGUGAUAUUUGAAGUAGUAUGUC

>hsa-mir-5692c-1 MI0019288

UAUAACAUUGUAUAUACCCACUGUGAUAUUAAGAGUAAUAGCUCUCUAGGUUAUUAUGAAUAAUAUCACAGUAGGUGUACACAAUGUUGUA

>hsa-mir-5693 MI0019300

CUGGGAAGUUAGUUCAUUUCAGUCUGUGCUGUGAGCUAGCCAGCAGUGGCUCUGAAAUGAACUCAAACUCUAG

>hsa-mir-5694 MI0019301

GCCAACUGCAGAUCAUGGGACUGUCUCAGCCCCAUAUGUAUCUGAAGGCUGAGAAGUCCCAUGAUCCGCACUUGGC

>hsa-mir-5695 MI0019302

CAAGGCCUAUCUAUCUAGAUUCUUCUUGGCCUCUCUGAGCAUGCAUUCCUGAGACUCCAAGAAGAAUCUAGACAGAUAGGCCUUG

>hsa-mir-5696 MI0019303

GUGCUCAUUUAAGUAGUCUGAUGCCUACUACUGAUGACAUACAAUGUAAGUGCUCAUUUAGGCGUCAGACUACCUAAAUGAGCAC

>hsa-mir-5697 MI0019304

AGCAUAUUCUCAAGUAGUUUCAUGAUAAAGGGUGUAUGAGAGAUCAACCCUUUAUCAUGAAACGCUUGAGGAUACGCU

>hsa-mir-5698 MI0019305

CUGUGCACCUGGGGGAGUGCAGUGAUUGUGGAAUGCAAAGUCCCACAAUCACUGUACUCCCCAGGUGCACAG

>hsa-mir-5699 MI0019306

CUGUACCCCUGCCCCAACAAGGAAGGACAAGAGGUGUGAGCCACACACACGCCUGGCCUCCUGUCUUUCCUUGUUGGAGCAGGGAUGUAG

>hsa-mir-5700 MI0019307

UUAAUUAAUGCAUUAAAUUAUUGAAGGCCCUUGGGCACCCCAGGCCUUCAAUAAUUUAAUGCAUUUAUUGA

>hsa-mir-5701-1 MI0019308

GAUUGGACUUUAUUGUCACGUUCUGAUUGGUUAGCCUAAGACUUGUUCUGAUCCAAUCAGAACAUGAAAAUAACGUCCAAUC

>hsa-mir-5702 MI0019309

GCCUCAACUCCUGGGAUAUGUUGCUGAUCCAACCUGAAAUCCUUCUGUAGGUUGAGUCAGCAACAUAUCCCAUGACUUUUGGGU

>hsa-mir-5703 MI0019310

UUGCCGUCCCCUUCCUCGUCUUUUCCCCUCAGGAGAAGUCGGGAAGGUGGCGGCGG

>hsa-mir-5704 MI0019312

UGAUCUUGUUUAGGCCAUCAUCCCAUUAUGCUAAGUCCAUGGGCAAACAUAACAGGAUGAUGGCCUAAACAAGACCA

>hsa-mir-5705 MI0019313

UCCCCAUUUACACAGGCCAUGAGCCCCGAAACACCCAUCCCAGGAUUGCUGAUGGGUGUUUCGGGGCUCAUGGCCUGUGUAAAUGGGGA

>hsa-mir-5706 MI0019314

AGCUAGGUCUUCUGGAUAACAUGCUGAAGCUUCUACGUCAUUCAGCACUUGCUUCAGCAUGUUUUCCAGAGGAUCUAGCU

>hsa-mir-5707 MI0019315

UGUAAGAACACGUUUGAAUGCUGUACAAGGCACAUAUGUGAACAUUGUACCACAUGUACAGCUUUCAAACAUGCUCUUAUA

>hsa-mir-5708 MI0019316

AUUACAGACAUGAGCGACUGUGCCUGACCAAAAGUCAACAUUAAACAACAAAUCUUGGCCAGGCACAGUGGCUCAUGCCUGUAAU

>hsa-mir-5739 MI0019412

GGUUGGCUAUAACUAUCAUUUCCAAGGUUGUGCUUUUAGGAAAUGUUGGCUGUCCUGCGGAGAGAGAAUGGGGAGCCAGG

>hsa-mir-6068 MI0020345

CCUGCGAGUCUCCGGCGGUGGCUUGUGGCUGAGUGUCACGCUGCUGGCGCAGGCUCGGCC

>hsa-mir-6069 MI0020346

UGGUGACCCCUGGGCUAGGGCCUGCUGCCCCCUGCCCAGUGCAGGAGGGUGGAGGGUCACUCCUUAGGUGGUCCCAGUG

>hsa-mir-6070 MI0020347

GGGUCACUUCUAGCCUCUCCGGUUCCAGUCCCUGGAGCUCUGUGCCUUUGGGUCCUGGUGAGCUCUUGUUGAUUGCAGUGGGAGAUGGGGAGAAUUGCCUUCA

>hsa-mir-6071 MI0020348

GUUUCUGCUGCCGGCCAAGGCUCUGGGUCCGGCUUACUCCCUAGCUUACUGUCCUCAGAACCCCCGCCACCACAGAAG

>hsa-mir-6072 MI0020349

GGGUGGGGGCUGGUGCAGGGAUGGGCUUUGCUACUGCUCCUCAUCACACUGCACCUUAGCUGCUGCCUGGA

>hsa-mir-6073 MI0020350

UAGAUGUUGGUCCAAACUGAAAGUUGAUGAGUCACUGUGCCUCUCGGGGUAGUGAGUUAUCAGCUACAGUGAGAGAGCAGUGUUUGGCC

>hsa-mir-6074 MI0020351

UACCAACCCCAUGGAAUUUUUACUCACCUUCAGUCAACUGAUUUGCUCUUUGGUGGAGAUAUUCAGAGGCUAGGUGGAGAUAGAGGUAGCCUUGAGGGUGGGUGUGG

>hsa-mir-6075 MI0020352

GACACCACAUGCUCCUCCAGGCCUGCCUGCCCUCCAGGUCAUGUUCCAGUGUCCCACAGAUGCAGCACCACGGCCCAGGCGGCAUUGGUGUCACC

>hsa-mir-6076 MI0020353

AGCAUGACAGAGGAGAGGUGGAGGUAGGCGAGAGUAAUAUAAUUUCUCCAGGAGAACAUCUGAGAGGGGAAGUUGCUUUCCUGCCCUGGCCCUUUCACCCUCCUGAGUUUGGG

>hsa-mir-6077-1 MI0020354

CCUGCGUAGAAAGGGAAGAGCUGUACGGCCUUCGCGCUAGUUUGUUUGUGUUAAGGCUGACGCUCCCUAUUUCUCCUCAGUG

>hsa-mir-6078 MI0020355

GCAGAGGAGAAAGAGCCACCGCCUGAGCUAGCUGUGGACAGAGGCAAAACUCUAACUUCAGCUGGUUUUGAGUGAGAAGUGGGUCUCUGUUAAAAUGCUA

>hsa-mir-6079 MI0020356

UCCUAGACCUAGUAUCAGUGGCCAAAGAAACUUUUUUGGAAGCUUGGACCAACUAGCUGGCU

>hsa-mir-6080 MI0020357

GCCUGCGGCUCUGGGAAUGCCGGUCUGGGGCUGGUUGUCUAGUGCGGGCGUUCCCGUUCGCGGGGA

>hsa-mir-6081 MI0020358

CCACCACGGUGCUGGCACCAGGGCCUCUGCCCCGUAGGACACCGAGGCUUAUGAAUAGGAGCAGUGCCGGCCAAGGCGCCGGCACCAUCUUGGUGAU

>hsa-mir-6082 MI0020359

UUGAAGAAUACGUCUGGUUGAUCCAGUCCACUUAAUUCUGAGAAAGUCCACAGACUGGAUCGGGUCUUGGAUUUUAGCUGGAUUCAUUGACUGGGCUAUAUUUGUCAUA

>hsa-mir-6083 MI0020360

GUAAAAAGAGUCCAGAGUCUGGGAAGGUGGAAAGGGAGCAGGAGCAUCGUCUUUAAGAGGGUCAGGUACCUUUGCUCUUAUAUCAGAGGCUGUGGGCUUCCUACAG

>hsa-mir-6084 MI0020361

AGCGUCCAGGCUGGGCCGCAGGACCGGGCGCGGAGCCUCGCAGGGUCGGGCUCGGGCUCCCUAACCGUCUCCGCUUCUUCCGCCAGUCGGUGGCCGGGCUGGCGGCGCGG

>hsa-mir-6085 MI0020362

GUCUACCAGGUGUGGGCCCAGCUUUACAUAGUUCAUGCUGAGGCCGGGAUUUCAUGCAGAAAACUGGUUGCAAAAGGUGCUGAAGGGGCUGGGGGAGCACAAGGGAGAAG

>hsa-mir-6086 MI0020363

AGGAGGUUGGGAAGGGCAGAGAUGAGCAUAAAGUUUUUGCCUUGUUUUUCUUUUU

>hsa-mir-6087 MI0020364

GGUGAGGCGGGGGGGCGAGCCCUGAGGGGCUCUCGCUUCUGGCGCCAAG

>hsa-mir-6088 MI0020365

AGAGAUGAAGCGGGGGGGCGGGGUCUUGCUCUAUUGCCUACGCUGAUCUCA

>hsa-mir-6089-1 MI0020366

CCCCGGGCCCGGCGUUCCCUCCCCUUCCGUGCGCCAGUGGAGGCCGGGGUGGGGCGGGGCGGGG

>hsa-mir-6090 MI0020367

CGCUGGGUCCGCGCGCCCUGGGCCGGGCGAUGUCCGCUUGGGGGAGCGAGGGGCGGGGCG

>hsa-mir-6124 MI0021258

GGGGAGGUAGGGAAAAGGAAGGGGGAGGAGAAGGUGAGACCAAUGUCCUGGGUGCCACUCCUGCCCAGUGCCUCCCUUCCUCGUU

>hsa-mir-6125 MI0021259

GCUCUGGGGCGUGCCGCCGCCGUCGCUGCCACCUCCCCUACCGCUAGUGGAAGAAGAUGGCGGAAGGCGGAGCGGCGGAUCUGGACACCCAGCGGU

>hsa-mir-6126 MI0021260

AGCCUGUGGGAAAGAGAAGAGCAGGGCAGGGUGAAGGCCCGGCGGAGACACUCUGCCCACCCCACACCCUGCCUAUGGGCCACACAGCU

>hsa-mir-6127 MI0021271

ACUUUCUCCAUCUACCAAGAUGAGGGAGUGGGUGGGAGGUGGGAAGGCUGCCCCAAAUGGCCUCUAACAUCCCUUCCAGUCUCCUCCUCCUCCUCCUCCUUCUUCUUCU

>hsa-mir-6128 MI0021272

AAGAAGCUUGUAGAUUUUUCUCCCUUACUAUCUAGAAUUAUAGGACUUCAGUCCAUGAUUUGGAAAAAUUACUGGAAUUGGAGUCAAAAAUAAUUUGAAAAUUAGGAAU

>hsa-mir-6129 MI0021274

GUUCCUUCUAAGGAUUCCAGUGAGGGAGUUGGGUGUAUAGGAAGGAAAUCUUAUUGUCCAAGUUUCCCUUUGAAACCUCUUAGAAUGGAAUAAAACUGCCCAAAUCAUA

>hsa-mir-6130 MI0021275

CAAUGAAUGUGGAAAUGCAGGCAUCCCUUCUAUAUACUGAUGUAAUUUUCCUUGGAUUUAUAUGCAGUACUGAGGGAGUGGAUUGUAUGGUAGUUCUAUUUUUACAUUU

>hsa-mir-6131 MI0021276

UCCCGCAUUCCCUCUGCUUUGGUCAGGUGGUGCCCUCCUUCCAUGGGUAGAGCCAGAGAUGGUGGGUUCUGGCUGGUCAGAUGGGAGUGGACAGAGACCCGGGGUCCUC

>hsa-mir-6132 MI0021277

UGCUAUUGUCUUACUGCUACAGCAGGGCUGGGGAUUGCAGUAUCCGCUGUUGCUGCUGCUCCCAGUCCUGCCCCUGCUGCUACCUAGUCCAGCCUCACCGCAUCCCAGA

>hsa-mir-6133 MI0021278

GGAAUGUCACCUGUGUGUUUUCUCUGCAUGCCCUCUUCAUUGUUCUGCUGAAGACUGGUCUCUUCAUGUGUGAGGGAGGAGGUUGGGUAUUGAGGGAAAACAGGGGGC

>hsa-mir-6134 MI0021279

UGGGCCUUGAUGUCAGUAGGGCUCAGUAGGGAACUGACCCUCUAUUUCCACCUGCAUAAAUGAAGCUGAAUGAGGUGGUAGGAUGUAGAGCUGGUUGACACUCUGCUUU

>hsa-mir-6165 MI0021472

CAGCAGGUCAGCAGGAGGUGAGGGGAGAGGAUCCACCUGUCCUGUCCUGUCCUCUCCUGCCCUGUCCUGGCUCCAGCCCCUCCC

>hsa-mir-6499 MI0022209

UCAGUCGGGCGCAAGAGCACUGCAGUUCUGUUGGGUGACAGCAGUGUUUGUUUUGCCCACAG

>hsa-mir-6500 MI0022211

CCUGCCUGCAGAAAGGAGCUAUCCACUCCAGGUGUCCUUUCUUCUGAGAGCUGGACACUUGUUGGGAUGACCUGCCUGCAGGUAGG

>hsa-mir-6501 MI0022213

GGAGUUGCCAGGGCUGCCUUUGGUGACAGCAGCAGUAGAGUUGCCAGAGCAGCCUGCGGUAACAGUA

>hsa-mir-6502 MI0022214

CAGAGUGGGAGCUCUAGAAAGAUUGUUGACCAAUCAUCUUAUUGACUAGACCAUCUUUCUAGAGUAUAACUAUUUU

>hsa-mir-6503 MI0022215

AAUGGUCCCCCCAGGGAGGUCUGCAUUCAAAUCCCCAGAAGCUGAGGAUUAGGGGACUAGGAUGCAGACCUCCCUGGGGGACCAUU

>hsa-mir-6504 MI0022216

GCAGUCUGGCUGUGCUGUAAUGCAGUCUGCACCCUGCUGCAUUACAGCACAGCCAUUCUCU

>hsa-mir-6505 MI0022217

GCAUUGGAAUAGGGGAUAUCUCAGCAUGUUGAGCCCUGUCUCUGGGGAGCUGACUUCUACCUCUUCCAAAG

>hsa-mir-6506 MI0022218

GACUGGGAUGUCACUGAAUAUGGUGUUUGUGAGUUGAUUGACAUCGUAUCAGAGAUUCCAGACACA

>hsa-mir-6507 MI0022219

GGAGGGAAGAAUAGGAGGGACUUUGUAUUGUGGUUCAGUACCAUGCAAAGUCCUUCCUAUUUUUCCCUCC

>hsa-mir-6508 MI0022220

UUCCUCUAGAAAUGCAUGACCCACCCUGAGUUUUGGUGGGCCAUGCAUUUCUAGAACUCC

>hsa-mir-6509 MI0022221

UUUUUGUGUGUGAAAUUAGGUAGUGGCAGUGGAACACUAUAUUAAUCAGGUUUCCACUGCCACUACCUAAUUUCUCAGAUGGAAA

>hsa-mir-6510 MI0022222

AGCAGCAGGGGAGAGAGAGGAGUCCUCUAGACACCGACUCUGUCUCCUGCAGAU

>hsa-mir-6511b-1 MI0022552

GGGACGGGGCCUGCAGGCAGAAGUGGGGCUGACAGGGCAGAGGGUUGCGCCCCCUCACCACCCCUUCUGCCUGCAGCGGUGGGCU

>hsa-mir-6512 MI0022224

UAUGCUCUUACCAUUAGAAGAGCUGGAAGAAGGCUGAGGGAGAUGCCUUCUUCCAGCCCUUCUAAUGGUAGGAGCAU

>hsa-mir-6513 MI0022225

GCUUUGGGAUUGACGCCACAUGUCUCAGGUCCCCAGCUGAGUCAAGUGUCAUCUGUCCCUAGGC

>hsa-mir-6514 MI0022226

UAUGGAGUGGACUUUCAGCUGGCAUUUACGAGUCAGAGUUCUUACAGAGCUGCCUGUUCUUCCACUCCAG

>hsa-mir-6515 MI0022227

CAUUGGAGGGUGUGGAAGACAUCUGGGCCAACUCUGAUCUCUUCAUCUACCCCCCAG

>hsa-mir-6516 MI0025513

UGGGUUUUGAAUUUGCAGUAACAGGUGUGAGCAUUCUAGCAGCAGUUUGAUGAUCAUGUAUGAUACUGCAAACAGGACCUA

>hsa-mir-6715a MI0022548

UGGGCUGCGUCCACAGGCACAGCCGGUUUGAGCAUUUUUACUGAAUUGCCAAACCAGUCGUGCCUGUGGGCACAACCUU

>hsa-mir-6715b MI0022549

GGUUGUGCCCACAGGCACGACUGGUUUGGCAAUUCAGUAAAAAUGCUCAAACCGGCUGUGCCUGUGGACGCAGCCCA

>hsa-mir-6716 MI0022550

GAGAGGCCAAGACCUUGGGAAUGGGGGUAAGGGCCUUCUGAGCCCAGGUCCGAACUCUCCAUUCCUCUGCAGAGCGCUCU

>hsa-mir-6717 MI0022551

CUGGUGUUUGAGGCGAUGUGGGGAUGUAGAGACAACUUCCCAGUCUCAUUUCCUCAUCCUGCCAGGCCACCAU

>hsa-mir-6718 MI0022553

AGUGAAUCCCUAGUGGUCAGAGGGCUUAUGAUAUAUUGUGAGAGCCAUGUCAUAAGCCUUUUGGCCACUAGGGAUUCAAU

>hsa-mir-6719 MI0022554

GCCCUACUCUCAGGAGGAGGCUGAUGUCUUCAGAGCAGGGUUUGGUCUGUGCUUCUCUGACAUCAGUGAUUCUCCUGCUGCUUUUAU

>hsa-mir-6720 MI0022555

UUGAGCGAGAGAUUGUGGCGCACCGAGUUCUUCCAGCCCUGGUAGGCGCCGCGGAAGAAGGGGAAGCGCGCCUGCAGGAACUGGUAGAUCUCGCUGAG

>hsa-mir-6721 MI0022556

CCCUCAUCUCUGGGCAGGGGCUUAUUGUAGGAGUCUCUGAAGAGAGCUGUGGACUGACCUGCUUUAACCCUUCCCCAGGUUCCCAUU

>hsa-mir-6722 MI0022557

GGCCUCAGGCAGGCGCACCCGACCACAUGCAUGGCUGGUGGCGGCGUGCAGGGGUCGGGUGGGCCAGGCUGUGGGGCG

>hsa-mir-6723 MI0022558

AUGCAUCGGGAUAGUCCGAGUAACGUCGGGGCAUUCCGGAUAGGCCGAGAAAGUGUUGUGGGAAGAAAGUUAGAUUUACGCCGAUGAAU

>hsa-mir-6724 MI0022559

CGCUGCGCUUCUGGGCCCGCGGCGGGCGUGGGGCUGCCCGGGCCGGUCGACCAGCGCGCCGUAGCUCCCGAGGCCCGAGCCGCGACCCGCGG

>hsa-mir-6726 MI0022571

GGGGGCGGGAGCUGGGGUCUGCAGGUUCGCACUGAUGCCUGCUCGCCCUGUCUCCCGCUAG

>hsa-mir-6727 MI0022572

GGGUGCUCGGGGCAGGCGGCUGGGAGCGGCCCUCACAUUGAUGGCUCCUGCCACCUCCUCCGCAG

>hsa-mir-6728 MI0022573

CUAGAUUGGGAUGGUAGGACCAGAGGGGCUUACUGCCCUGUGGGGCUCUCUGGACCCAGUGCCAUGCUUCUCUGCUCUGCUCUCCCCAG

>hsa-mir-6729 MI0022574

GAGGGUGGGCGAGGGCGGCUGAGCGGCUCCAUCCCCCGGCCUGCUCAUCCCCCUCGCCCUCUCAG

>hsa-mir-6730 MI0022575

CCGAAAGAAAGGUGGAGGGGUUGUCAGAGCUGCGGCUGCUCCACCCUGACACCCCAUCUGCCCUCAG

>hsa-mir-6731 MI0022576

ACAGGUGGGAGAGCAGGGUAUUGUGGAAGCUCCAGGUGCCAACCACCUGCCUCUAUUCCCCACUCUCCCCAG

>hsa-mir-6732 MI0022577

AGGCCUAGGGGGUGGCAGGCUGGCCAUCAGUGUGGGCUAACCCUGUCCUCUCCCUCCCAG

>hsa-mir-6733 MI0022578

GUGCUUGGGAAAGACAAACUCAGAGUUCCCUUCUUGUGAGCUCAGUGUCUGGAUUUCCUAG

>hsa-mir-6734 MI0022579

AGAACUUGAGGGGAGAAUGAGGUGGAGAAGCCCAGGUUCUGAAUCCCCUUCCCUCACUCUUCUCUCAG

>hsa-mir-6735 MI0022580

GCAGCCAGGGCAGAGGGCACAGGAAUCUGAGGUGACUGGCACAGAAGACUCAGGCCUGUGGCUCCUCCCUCAG

>hsa-mir-6736 MI0022581

CUGAGCUGGGUGAGGGCAUCUGUGGUUUGCUGGCUGCCUCAGCUCCUCUCUACCCACAG

>hsa-mir-6737 MI0022582

UUGGGUUGGGGUGGUCGGCCCUGGAGGGGGUUUGUUUGCUUAUUCCCCUCUGUGCUUCACCCCUACCCAG

>hsa-mir-6738 MI0022583

GAAGGCGAGGGGUAGAAGAGCACAGGGGUUCUGAUAAACCCUUCUGCCUGCAUUCUACUCCCAG

>hsa-mir-6739 MI0022584

GAAUGUGGGAAAGAGAAAGAACAAGUAAAAGGAAUUUUCAUUUUCCAGCCCCUAAUUGUUCUGUCUUUCUCCCAG

>hsa-mir-6740 MI0022585

GAAAGAGUUUGGGAUGGAGAGAGGAGAAACUUGAGGUCUCUGGGAGUUGCUUAAACCAGUUGACCGUAACCUGGCCAGAGAAUUCUGAUAGUGUCUUCUCUCCUCCCAAACAG

>hsa-mir-6741 MI0022586

AAUGGGUGGGUGCUGGUGGGAGCCGUGCCCUGGCCACUCAUUCGGCUCUCUCCCUCACCCUAG

>hsa-mir-6742 MI0022587

GAGGGAGUGGGGUGGGACCCAGCUGUUGGCCAUGGCGACAACACCUGGGUUGUCCCCUCUAG

>hsa-mir-6743 MI0022588

GGGUAAAGGGGCAGGGACGGGUGGCCCCAGGAAGAAGGGCCUGGUGGAGCCGCUCUUCUCCCUGCCCACAG

>hsa-mir-6744 MI0022589

UCACGUGGAUGACAGUGGAGGCCUCCUGGAUCUCUAGGUCUCAGGGCCUCUCUUGUCAUCCUGCAG

>hsa-mir-6745 MI0022590

GGUCCUUGAGGGCAGGAGCUGGGUCUUGGGACUGAUUGCCCCUAGUGGCUAGCCCUGGGUCUAGCAGCCUAUGGCAGUGUCUGGUAAACACUGGUGGAGUCCUGGGUGGAAGAAGGUCUGGUUCUCA

>hsa-mir-6746 MI0022591

CUUGCCCGGGAGAAGGAGGUGGCCUGGAGAGCUGCUGUCUCCAGCCGCCGCCUGUCUCCACAG

>hsa-mir-6747 MI0022592

UUUGGAGGGGUGUGGAAAGAGGCAGAACAUUCGUUCACUUUCCUGCCUUCCUCUGCACCAG

>hsa-mir-6748 MI0022593

UGGUGUGUGGGUGGGAAGGACUGGAUUUGAAAUGGUCCCACUCCUGACGAUCCUGUCCCUGUCUCCUACAG

>hsa-mir-6749 MI0022594

GGCCCUCGGGCCUGGGGUUGGGGGAGCUCUGUCCUGUCUCACUCAUUGCUCCUCCCCUGCCUGGCCCAG

>hsa-mir-6750 MI0022595

GCUGUCAGGGAACAGCUGGGUGAGCUGCUGCCCCAGAGGCCCAGCAGGUGUCCAGAACUCACCCUCUGCUCCCAG

>hsa-mir-6751 MI0022596

UCUUCUUGGGGGUGAGGUUGGUGUCUGGCCCCAGCAGCCCAGACUGAGCCUCUCUCUCUCCAG

>hsa-mir-6752 MI0022597

AUGGAGGGGGGUGUGGAGCCAGGGGGCCCAGGUCUACAGCUUCUCCCCGCUCCCUGCCCCCAUACUCCCAG

>hsa-mir-6753 MI0022598

CACCAGGGCAGAGCAGGGCUGAUCAUCUCACGUCAGAGAGAGGGGAAGGGGCUGCCCAGUGAGCCCCCACAGGGCUCUACAUCUCCAGCUGGGCCUGGCUGGAGAUCCCAGGGUCCCUGAAGGCCCCCGCCACCGUUCUGGUCUGUCUCUGCCCUGGCACCCAG

>hsa-mir-6754 MI0022599

GGCUGCCAGGGAGGCUGGUUUGGAGGAGUCUGGUGGCCUGUUCUCUUCACCUGCCUCUGCCUGCAG

>hsa-mir-6755 MI0022600

UGUUUUAGGGUAGACACUGACAACGUUAUGUGUGGUCUUUAACCUGUUGUCAUGUUUUUUCCCUAG

>hsa-mir-6756 MI0022601

ACCCUAGGGUGGGGCUGGAGGUGGGGCUGAGGCUGAGUCUUCCUCCCCUUCCUCCCUGCCCAG

>hsa-mir-6757 MI0022602

GGGCUUAGGGAUGGGAGGCCAGGAUGAAGAUUAAUCCCUAAUCCCCAACACUGGCCUUGCUAUCCCCAG

>hsa-mir-6758 MI0022603

UGGGCUAGAGAGGGGAAGGAUGUGAUGUGAGCAGAUGGUUCUCACUCAUUCUCCUCUGUCCAG

>hsa-mir-6759 MI0022604

UAUUGUUGUGGGUGGGCAGAAGUCUGUUUUCUUCAUGGUUUUCUGACCUUUGCCUCUCCCCUCAG

>hsa-mir-6760 MI0022605

CAGUGCAGGGAGAAGGUGGAAGUGCAGAGUGGGCUCACCUCUCGCCCACACUGUCCCCUUCUCCCCAG

>hsa-mir-6761 MI0022606

UCUGCUCUGAGAGAGCUCGAUGGCAGGUGCCUCCGUGUUGCCGAACCCUCCUACGCUGCUCUCUCACUCCAG

>hsa-mir-6762 MI0022607

AGAGCCGGGGCCAUGGAGCAGCCUGUGUAGACGGGGACCUGCCCUGCAUGGGCACCCCCUCACUGGCUGCUUCCCUUGGUCUCCAG

>hsa-mir-6763 MI0022608

UUCUCCUGGGGAGUGGCUGGGGAGCAGACAGACCCAACCUCAUGCUCCCCGGCCUCUGCCCCCAG

>hsa-mir-6764 MI0022609

CUGACUCCCAGGGUCUGGUCAGAGUUGCUGAGUGGGUUGAUCUCUGGUCUUUCCUUGACAG

>hsa-mir-6765 MI0022610

GUGAGGCGGGGCCAGGAGGGUGUGUGGCGUGGGUGCUGCGGGGCCGUCAGGGUGCCUGCGGGACGCUCACCUGGCUGGCCCGCCCAG

>hsa-mir-6766 MI0022611

AUGAGCGGGUGGGAGCAGAUCUUAUUGAGAGUUCCUUCUCCUGCUCCUGAUUGUCUUCCCCCACCCUCACAG

>hsa-mir-6767 MI0022612

UGAAAUCGCAGACAGGGACACAUGGAGAACGCCCCCACCAGUUCCCACGUGCUUCUCUUUCCGCAG

>hsa-mir-6768 MI0022613

CCAGGCACACAGGAAAAGCGGGGCCCUGGGUUCGGCUGCUACCCCAAAGGCCACAUUCUCCUGUGCACACAG

>hsa-mir-6769a MI0022614

AGGCCAGGUGGGUAUGGAGGAGCCCUCAUAUGGCAGUUGGCGAGGGCCCAGUGAGCCCCUCUCUGCUCUCCAG

>hsa-mir-6769b MI0022706

CUUCCUGGUGGGUGGGGAGGAGAAGUGCCGUCCUCAUGAGCCCCUCUCUGUCCCACCCAUAG

>hsa-mir-6770-1 MI0022615

UAUCCUGAGAAGGCACAGCUUGCACGUGACCUCCUGGGCCUGGCGGCUGUGUCUUCACAG

>hsa-mir-6771 MI0022616

GGUGCCUCGGGAGGGCAUGGGCCAGGCCACAUAAUGAGCCAAACCCCUGUCUACCCGCAG

>hsa-mir-6772 MI0022617

AGGCCUGGGUGUAGGCUGGAGCUGAGGACUGAGGCUCACCUUGCUCCUGACUCUGUGCCCACAG

>hsa-mir-6773 MI0022618

GGGAGUUGGGCCCAGGAGUAAACAGGAUUAGUGCUUAUGCUAAGAUGUCUUCACUGUCACUUCUCUGCCCAUAG

>hsa-mir-6774 MI0022619

UGUGCACUUGGGCAGGAGGGACCCUGUAUGUCUCCCCGCAGCACCGUCAUCGUGUCCCUCUUGUCCACAG

>hsa-mir-6775 MI0022620

GAACCUCGGGGCAUGGGGGAGGGAGGCUGGACAGGAGAGGGCUCACCCAGGCCCUGUCCUCUGCCCCAG

>hsa-mir-6776 MI0022621

CGGGCUCUGGGUGCAGUGGGGGUUCCCACGCCGCGGCAACCACCACUGUCUCUCCCCAG

>hsa-mir-6777 MI0022622

UCAAGACGGGGAGUCAGGCAGUGGUGGAGAUGGAGAGCCCUGAGCCUCCACUCUCCUGGCCCCCAG

>hsa-mir-6778 MI0022623

GUUCAAGUGGGAGGACAGGAGGCAGGUGUGGUUGGAGGAAGCAGCCUGAACCUGCCUCCCUGACAUUCCACAG

>hsa-mir-6779 MI0022624

GAGCUCUGGGAGGGGCUGGGUUUGGCAGGACAGUUUCCAAGCCCUGUCUCCUCCCAUCUUCCAG

>hsa-mir-6780a MI0022625

GACACUUGGGAGGGAAGACAGCUGGAGAGUAUGGUCACAGCAGCAUCCUCCUCUGUUUUCUUUCCUAG

>hsa-mir-6780b MI0022681

CAGCCUGGGGAAGGCUUGGCAGGGAAGACACAUGAGCAGUGCCUCCACUUCACGCCUCUCCCUUGUCUCCUUUCCCUAG

>hsa-mir-6781 MI0022626

AACCCCGGGCCGGAGGUCAAGGGCGUCGCUUCUCCCUAAUGUUGCCUCUUUUCCACGGCCUCAG

>hsa-mir-6782 MI0022627

UGGGGUAGGGGUGGGGGAAUUCAGGGGUGUCGAACUCAUGGCUGCCACCUUUGUGUCCCCAUCCUGCAG

>hsa-mir-6783 MI0022628

CCUGUUAGGGGAAAAGUCCUGAUCCGGGAACCCACAGCCCCGUUCCUGGGCUUCUCCUCUGUAG

>hsa-mir-6784 MI0022629

UACAGGCCGGGGCUUUGGGUGAGGGACCCCCGGAGUCUGUCACGGUCUCACCCCAACUCUGCCCCAG

>hsa-mir-6785 MI0022630

CUCCCUGGGAGGGCGUGGAUGAUGGUGGGAGAGGAGCCCCACUGUGGAAGUCUGACCCCCACAUCGCCCCACCUUCCCCAG

>hsa-mir-6786 MI0022631

GCCGGGUGGGGCGGGGCGGCCUCAGGAGGGGCCCAGCUCCCCUGGAUGUGCUGCGGUGGGGCCGGAGGGGCGUCACGUGCACCCAAGUGACGCCCCUUCUGAUUCUGCCUCAG

>hsa-mir-6787 MI0022632

UCGGCUGGCGGGGGUAGAGCUGGCUGCAGGCCCGGCCCCUCUCAGCUGCUGCCCUCUCCAG

>hsa-mir-6788 MI0022633

GACGGCUGGGAGAAGAGUGGUGAAGAAGAGUAUUGAUUGUGCUGUUCGCCACUUCCCUCCCUGCAG

>hsa-mir-6789 MI0022634

CGAGGUAGGGGCGUCCCGGGCGCGCGGGCGGGUCCCAGGCUGGGCCCCUCGGAGGCCGGGUGCUCACUGCCCCGUCCCGGCGCCCGUGUCUCCUCCAG

>hsa-mir-6790 MI0022635

GUGAGUGUGGAUUUGGCGGGGUUCGGGGGUUCCGACGGCGACCUCGGCGACCCCUCACUCACC

>hsa-mir-6791 MI0022636

CCAGACCCCUGGGGCUGGGCAGGCGGAAAGAGGUCUGAACUGCCUCUGCCUCCUUGGUCUCCGGCAG

>hsa-mir-6792 MI0022637

GUAAGCAGGGGCUCUGGGUGAUGUGAGGAGCAACAGGCACCCUCCUCCACAGCCCCUGCUCAUUCCU

>hsa-mir-6793 MI0022638

GUCACUGUGGGUUCUGGGUUGGGGUGAUACACAAGCCUGACCCUCCCCAACCCCUGCCCGCAG

>hsa-mir-6794 MI0022639

GGGCGCAGGGGGACUGGGGGUGAGCAGGCCCAGAACCCAGCUCGUGCUCACUCUCAGUCCCUCCCUAG

>hsa-mir-6795 MI0022640

AGGGUUGGGGGGACAGGAUGAGAGGCUGUCUUCAUUCCCUCUUGACCACCCCUCGUUUCUUCCCCCAG

>hsa-mir-6796 MI0022641

UUACCUUGUGGGGUUGGAGAGCUGGCUGGUCCAGCCCCUCAGAAGCUCUCCCCUCCCCGCAG

>hsa-mir-6797 MI0022642

CAGCCAGGAGGGAAGGGGCUGAGAACAGGACCUGUGCUCACUGGGGCCUGCAUGACCCUUCCCUCCCCACAG

>hsa-mir-6798 MI0022643

GGCAGCCAGGGGGAUGGGCGAGCUUGGGCCCAUUCCUUUCCUUACCCUACCCCCCAUCCCCCUGUAG

>hsa-mir-6799 MI0022644

GAGGAGGGGAGGUGUGCAGGGCUGGGGUCACUGACUCUGCUUCCCCUGCCCUGCAUGGUGUCCCCACAG

>hsa-mir-6800 MI0022645

ACCUGUAGGUGACAGUCAGGGGCGGGGUGUGGUGGGGCUGGGGCUGGCCCCCUCCUCACACCUCUCCUGGCAUCGCCCCCAG

>hsa-mir-6801 MI0022646

UGGCCUGGUCAGAGGCAGCAGGAAAUGAGAGUUAGCCAGGAGCUUUGCAUACUCACCCCUGCCACUCACUGGCCCCCAG

>hsa-mir-6802 MI0022647

GAGGGCUAGGUGGGGGGCUUGAAGCCCCGAGAUGCCUCACGUCUUCACCCCUCUCACCUAAGCAG

>hsa-mir-6803 MI0022648

CUCCUCUGGGGGUGGGGGGCUGGGCGUGGUGGACAGCGAUGCAUCCCUCGCCUUCUCACCCUCAG

>hsa-mir-6804 MI0022649

GGAUGUGAGGGUGUCAGCAGGUGACGGUGGGGGCCACGCUGACAGCCGCACCUGCCUCUCACCCACAG

>hsa-mir-6805 MI0022650

UGGCCUAGGGGGCGGCUUGUGGAGUGUAUGGGCUGAGCCUUGCUCUGCUCCCCCGCCCCCAG

>hsa-mir-6806 MI0022651

UGCUCUGUAGGCAUGAGGCAGGGCCCAGGUUCCAUGUGAUGCUGAAGCUCUGACAUUCCUGCAG

>hsa-mir-6807 MI0022652

GUGAGCCAGUGGAAUGGAGAGGCUGUGGGCAGGGGGAGAUGUGAAGGAAAGAACUAGGACCCAUUCAUCCACUGCAUUCCUGCUUGGCCCAG

>hsa-mir-6808 MI0022653

GGGGCCAGGCAGGGAGGUGGGACCAUGGGGGCCUUGCUGUGUGACCACCGUUCCUGCAG

>hsa-mir-6809 MI0022654

AAUGUUGGCAAGGAAAGAAGAGGAUCAUGUUUGCCCCCGUGGACAGCUCUCUGGUGUGCUCCUCCUGCCAUCCUGCCCACCCCUGCAUAAUGCUGCUUCUCUUCUCUCCUUCCCAG

>hsa-mir-6810 MI0022655

CUGGGAUGGGGACAGGGAUCAGCAUGGCACAGAUCCAAUACCUUCUGUCCCCUGCUCCCUUGUUCCCCAG

>hsa-mir-6811 MI0022656

UAUGCAGGCCUGUGUACAGCACUCAGGCAGUGCCAUGAGCCUGUGCUUGUCCCUGCAG

>hsa-mir-6812 MI0022657

UGAGGAUGGGGUGAGAUGGGGAGGAGCAGCCAGUCCUGUCUCACCGCUCUUCCCCUGACCCCAG

>hsa-mir-6813 MI0022658

GUAGGCAGGGGCUGGGGUUUCAGGUUCUCAGUCAGAACCUUGGCCCCUCUCCCCAG

>hsa-mir-6814 MI0022659

UUUCCUCCCAAGGGUGAGAUGCUGCCACCCAGCCCUGCAGAGCCCCUGACUCGCAUCCUUCCCUUGGCAG

>hsa-mir-6815 MI0022660

CACUGUAGGUGGCGCCGGAGGAGUCAUUUCCCAUCACUAAUGGCUUCUCUUGCACACCCAG

>hsa-mir-6816 MI0022661

CCGAGUGGGGCGGGGCAGGUCCCUGCAGGGACUGUGACACUGAAGGACCUGCACCUUCGCCCACAG

>hsa-mir-6817 MI0022662

AGGAUUCUGCCAUAGGAAGCUUGGAGUGGAACUGACCUGCCCCCUUUCUCUCUGACUCCAUGGCAG

>hsa-mir-6818 MI0022663

CUAUUUUGUGUGAGUACAGAGAGCAUCUGAAUGGGUACAGUUGUUGUCUCUUGUUCCUCACACAG

>hsa-mir-6819 MI0022664

GAGGGUUGGGGUGGAGGGCCAAGGAGCUGGGUGGGGUGCCAAGCCUCUGUCCCCACCCCAG

>hsa-mir-6820 MI0022665

CCUUCUGCGGCAGAGCUGGGGUCACCAGCCCUCAUGUACUUGUGACUUCUCCCCUGCCACAG

>hsa-mir-6821 MI0022666

GUGCGUGGUGGCUCGAGGCGGGGGUGGGGGCCUCGCCCUGCUUGGGCCCUCCCUGACCUCUCCGCUCCGCACAG

>hsa-mir-6822 MI0022667

UGGCCCAGGGAACCAGUUGGGGCUUCCGCUCUGCAGAGGCUCUAACUGGCUUUCCCUGCAG

>hsa-mir-6823 MI0022668

CAAGGUCAGGGUUGGUAGGGGUUGCUGUUGCUGUGAAAGCUGAGCCUCUCCUUCCCUCCAG

>hsa-mir-6824 MI0022669

GAGGUGUAGGGGAGGUUGGGCCAGGGAUGCCUUCACUGUGUCUCUCUGGUCUUGCCACCCCAG

>hsa-mir-6825 MI0022670

GGGCAUGGGGAGGUGUGGAGUCAGCAUGGGGCUAGGAGGCCCCGCGCUGACCCGCCUUCUCCGCAG

>hsa-mir-6826 MI0022671

CUUGGUCAAUAGGAAAGAGGUGGGACCUCCUGGCUUUUCCUCUGCAGCAUGGCUCGGACCUAGUGCAAUGUUUAAGCUCCCCUCUCUUUCCUGUUCAG

>hsa-mir-6827 MI0022672

UCUGGUGGGAGCCAUGAGGGUCUGUGCUGUCUCUGAGCACCGUCUCUUCUGUUCCCCAG

>hsa-mir-6828 MI0022673

GGCUCAGGAAGCAAGAGAACCCUGUGGUCUAACCUUUCCCAUCUGCUCUCUUGUUCCCAG

>hsa-mir-6829 MI0022674

CAGCGUGGGCUGCUGAGAAGGGGCAGGGUCCUCCAGCUCAUUCCUCCUGCCUCCUCCGUGGCCUCAG

>hsa-mir-6830 MI0022675

GUGCCCCAAGGAAGGAGGCUGGACAUCCCUCAUCUGUUUCUCACUGGUGUCUUUCUUCUCUCCCUUGCAG

>hsa-mir-6831 MI0022676

GUAGGUAGAGUGUGAGGAGGAGGUCUGAGCCCAUGUGUGGACCUAGGUCUGCUGUUAAACUGACUAACUCCCACUCUACAG

>hsa-mir-6832 MI0022677

GGUGGAGUAGAGAGGAAAAGUUAGGGUCAGUGGCAGAGCCAGGCAGAUGCUGACCCUUUUUCUCUUUCCCAG

>hsa-mir-6833 MI0022678

AAACGGUGUGGAAGAUGGGAGGAGAAAAAUCCCUGUUAACUUUCUCUCUCCACUUCCUCAG

>hsa-mir-6834 MI0022679

GUGAGGGACUGGGAUUUGUGGGGCGAGGAGGGACCUGUACUAGCCAUGGUUCUGAUCACAUAUGUCCCAUCCCUCCAUCAG

>hsa-mir-6835 MI0022680

UGAUGAGGGGGUAGAAAGUGGCUGAAGCGAGAUGUUUGUCUAAAAGCACUUUUCUGUCUCCCAG

>hsa-mir-6836 MI0022682

GGCUCCGCAGGGCCCUGGCGCAGGCAUCCAGACAGCGGGCGAAUGCCUCCCCCGGCCCCGCAG

>hsa-mir-6837 MI0022683

GUGGGACCAGGGCCAGCAGGGAAUGUCAGGGCCACCCCUGACCUUCACUGUGACUCUGCUGCAG

>hsa-mir-6838 MI0022684

CAGGGAAGCAGCAGUGGCAAGACUCCUAGGUCACGGAAGUCCUGCUUCUGUUGCAG

>hsa-mir-6839 MI0022685

UGUAGUCUGGAUUGAAGAGACGACCCAAGCAGGCUUUGUGUGAGCAGUGAGGCUAUUUAUUCACUUGGGUGCGAGCUCACACGAAGCCUGCUUGGGUUUUCUCUUCAAUCCAG

>hsa-mir-6840 MI0022686

UGACCACCCCCGGGCAAAGACCUGCAGAUCCCCUGUUAGAGACGGGCCCAGGACUUUGUGCGGGGUGCCCA

>hsa-mir-6841 MI0022687

GUGUUUAGGGUACUCAGAGCAAGUUGUGAAACACAGGUGUUUUUUAACCUCACCUUGCAUCUGCAUCCCCAG

>hsa-mir-6842 MI0022688

AGCCCUGGGGGUGGUCUCUAGCCAAGGCUCUGGGGUCUCACCCUUGGCUGGUCUCUGCUCCGCAG

>hsa-mir-6843 MI0022689

CCCCCAUUUCCUCAGGAAUAAAAGUGCAGCAGUGCCUGCUGUGGGGACAGCUGAGGGCAGUGAGGCCCUGGGGAGCUGCUGCAGGCAGCAGGUGGGCGGGACGCCAGCAGGCUGUCUAGCUGUUCCCAUGAUGGUCUCCUGUUCUCUGCAG

>hsa-mir-6844 MI0022690

GAACUUAAGAAUUUUGUAGAAAUCAAGCUAUUUGCUAAAAGUUCUUUGUUUUUAAUUCACAG

>hsa-mir-6845 MI0022691

AACUGCGGGGCCAGAGCAGAGAGCCCUUGCACACCACCAGCCUCUCCUCCCUGUGCCCCAG

>hsa-mir-6846 MI0022692

CAGGCUGGGGGCUGGAUGGGGUAGAGUAGGAGAGCCCACUGACCCCUUCUGUCUCCCUAG

>hsa-mir-6847 MI0022693

GACCCACAGAGGACAGUGGAGUGUGAGCUGGAAGGAGUGGGCCUGGCUCAUGUGUCUGUCCUCUUCCAG

>hsa-mir-6848 MI0022694

GUCCCUGGGGGCUGGGAUGGGCCAUGGUGUGCUCUGAUCCCCCUGUGGUCUCUUGGCCCCCAGGAACUCC

>hsa-mir-6849 MI0022695

CCUGGGAGUGGAUAGGGGAGUGUGUGGAGAGAGCACUGAGCCUGCCCACCAGCCUGUGUCCACCUCCAG

>hsa-mir-6850 MI0022696

GUGCGGAACGCUGGCCGGGGCGGGAGGGGAAGGGACGCCCGGCCGGAACGCCGCACUCACG

>hsa-mir-6851 MI0022697

CAGGGAGGAGGUGGUACUAGGGGCCAGCAACCUGAUUACCCCUCUUUGGCCCUUUGUACCCCUCCAG

>hsa-mir-6852 MI0022698

UGCUGCCCUGGGGUUCUGAGGACAUGCUCUGACUCCCCUGAUGUCCUCUGUUCCUCAGGUGCUGGG

>hsa-mir-6853 MI0022699

GGGAAAGCGUGGGAUGUCCAUGAAGUCAGGUGAUGGUGAUAAGGUCAAGGCCUGUUCAUUGGAACCCUGCGCAG

>hsa-mir-6854 MI0022700

AAAGCAAGCUCAGGUUUGAGAACUGCUGAUGUCAUCAGUCAUAACUUCUGCGUUUCUCCUCUUGAGCAG

>hsa-mir-6855 MI0022701

GCUGCUUGGGGUUUGGGGUGCAGACAUUGCCAGAGGAUGGGCAGCAGACUGACCUUCAACCCCACAG

>hsa-mir-6856 MI0022702

UGGAAAAGAGAGGAGCAGUGGUGCUGUGGCAGUGGCAGAGGUCGCUACAGCCCUGUGAUCUUUCCAG

>hsa-mir-6857 MI0022703

GCUUGUUGGGGAUUGGGUCAGGCCAGUGUUCAAGGGCCCCUCCUCUAGUACUCCCUGUUUGUGUUCUGCCACUGACUGAGCUUCUCCCCACAG

>hsa-mir-6858 MI0022704

GUGAGGAGGGGCUGGCAGGGACCCCUCCAAGUUGGGGACGGCAGCCAGCCCCUGCUCACCCCUCGCC

>hsa-mir-6859-1 MI0022705

UGUGGGAGAGGAACAUGGGCUCAGGACAGCGGGUGUCAGCUUGCCUGACCCCCAUGUCGCCUCUGUAG

>hsa-mir-6860 MI0022707

GUUAAGCAUUGGGGAGUUUGGAGUCGGUGGGUGGAGCCAAACUGGGCAGGGCUGUGGUGAGUGAGU

>hsa-mir-6861 MI0022708

GAGGCACUGGGUAGGUGGGGCUCCAGGGCUCCUGACACCUGGACCUCUCCUCCCCAGGCCCACA

>hsa-mir-6862-1 MI0022709

CGAAGCGGGCAUGCUGGGAGAGACUUUGUGAUUUGUCUCCAAAGCCUCACCCAGCUCUCUGGCCCUCUAG

>hsa-mir-6863 MI0022710

AUUUAUGAGGCAGCAGAGUCUACAAGUAAAUCAUGAAUCCAGUUGAAAAUGUUAAUGAGGCCAUAGACGUGGUGAAGGAUUGAGUGACCU

>hsa-mir-6864 MI0022711

GAAAGUUGAAGGGACAAGUCAGAUAUGCCAUUAUGUGGGUCAUGAGGUGGUGAGACUUCUCUCCCUUCAG

>hsa-mir-6865 MI0022712

AUCCAUAGGUGGCAGAGGAGGGACUUCAGAUGCUUCAUGACACCCUCUUUCCCUACCGCCUACAG

>hsa-mir-6866 MI0022713

CCAUUUUAGAGGCUGGAAUAGAGAUUCUUGAGGCUUGGAAGAGUAAGGAUCCCUUUAUCUGUCCUCUAG

>hsa-mir-6867 MI0022714

CCCGGUGUGUGUGUAGAGGAAGAAGGGAAGCUGGGAACCUGACUGCCUCUCCCUCUUUACCCACUAG

>hsa-mir-6868 MI0022715

CCAGGACUGGCAGAACACUGAAGCAGCAGGCACCUGCUUCCUUCUGUUGUCUGUGCAG

>hsa-mir-6869 MI0022716

GUGAGUAGUGGCGCGCGGCGGCUCGGAGUACCUCUGCCGCCGCGCGCAUCGGCUCAGCAUGC

>hsa-mir-6870 MI0022717

CAAGGUGGGGGAGAUGGGGGUUGAACUUCAUUUCUCAUGCUCAUCCCCAUCUCCUUUCAG

>hsa-mir-6871 MI0022718

CUCCUCAUGGGAGUUCGGGGUGGUUGCUGGAGGUCAGCACCCUGUGGCUCCCACAG

>hsa-mir-6872 MI0022719

GUGGGUCUCGCAUCAGGAGGCAAGGCCAGGACCCGCUGACCCAUGCCUCCUGCCGCGGUCAG

>hsa-mir-6873 MI0022720

CCCAGCAGAGGGAAUACAGAGGGCAAUCAGGACUGGGUCAUUCUCUCUGUCUUUCUCUCUCAG

>hsa-mir-6874 MI0022721

GCCACAUGGAGCUGGAACCAGAUCAGGCUUUAAUGUUUGAAGUAAUGUCAGUUCUGCUGUUCUGACUCUAG

>hsa-mir-6875 MI0022722

GAGUCUGAGGGACCCAGGACAGGAGAAGGCCUAUGGUGAUUUGCAUUCUUCCUGCCCUGGCUCCAUCCUCAG

>hsa-mir-6876 MI0022723

AGUUGCAGGAAGGAGACAGGCAGUUCAGGAGGUGGCACUGCUGUGUGUGAGCUGUCUGUGUUUUCCUUCUCAG

>hsa-mir-6877 MI0022724

AGUUCAGGGCCGAAGGGUGGAAGCUGCUGGUGCUCAUCUCAGCCUCUGCCCUUGGCCUCCCCAG

>hsa-mir-6878 MI0022725

AUGAGAGGGAGAAAGCUAGAAGCUGAAGAUUCUGAAAAUCACUAACUGGCCUCUUCUUUCUCCUAG

>hsa-mir-6879 MI0022726

CAGAGCAGGGCAGGGAAGGUGGGAGAGGGGCCCAGCUGACCCUCCUGUCACCCGCUCCUUGCCCAG

>hsa-mir-6880 MI0022727

GAGGGUGGUGGAGGAAGAGGGCAGCUCCCAUGACUGCCUGACCGCCUUCUCUCCUCCCCCAG

>hsa-mir-6881 MI0022728

UGCACUGGGGUAAGGAUAGGAGGGUCAGGUCUGCAGCCUUGUAUCUGCUGAUCCUCUUUCGUCCUUCCCACUCCAG

>hsa-mir-6882 MI0022729

GGCUUUACAAGUCAGGAGCUGAAGCAGCUGGAAUUCAAGCCCUGCUGCCUCUCCUCUUGCCUGCAG

>hsa-mir-6883 MI0022730

CAGACAGGGAGGGUGUGGUAUGGAUGUGUUGACCCCUGAAGUGGUUCUGAUGACCUUUCCCUAUCUCACUCUCCUCAG

>hsa-mir-6884 MI0022731

CCCGCAGAGGCUGAGAAGGUGAUGUUGGCUCAAGAAAGGGAGAUAGAUGGUAGCCCAUCACCUUUCCGUCUCCCCUAG

>hsa-mir-6885 MI0022732

CCUGGAGGGGGGCACUGCGCAAGCAAAGCCAGGGACCCUGAGAGGCUUUGCUUCCUGCUCCCCUAG

>hsa-mir-6886 MI0022733

CUUGGCCCGCAGGUGAGAUGAGGGCUCCUGGCGCUGAUGCCCUUCUCUCCUCCUGCCUCAG

>hsa-mir-6887 MI0022734

GAGAAUGGGGGGACAGAUGGAGAGGACACAGGCUGGCACUGAGGUCCCCUCCACUUUCCUCCUAG

>hsa-mir-6888 MI0022735

GUGGGAAGGAGAUGCUCAGGCAGAUCUGUCUCUGAUUGUUUCCAAGAUCUGUCUCGAUUGUUUCCAG

>hsa-mir-6889 MI0022736

CUGUGUCGGGGAGUCUGGGGUCCGGAAUUCUCCAGAGCCUCUGUGCCCCUACUUCCCAG

>hsa-mir-6890 MI0022737

UGGGCCAUGGGGUAGGGCAGAGUAGGGCUGGAUGGUAGGGCCCACUGCCUAUGCCCCACAG

>hsa-mir-6891 MI0022738

GUAAGGAGGGGGAUGAGGGGUCAUAUCUCUUCUCAGGGAAAGCAGGAGCCCUUCAGCAGGGUCAGGGCCCCUCAUCUUCCCCUCCUUUCCCAG

>hsa-mir-6892 MI0022739

GUAAGGGACCGGAGAGUAGGAAAAGCAGGGCUCAGGGCCAGAGAGACUGGGCAUAGAACUAAGGAGGAUGGUGUCCUCCUGACUGCAUCUCUCUUCCCUCUCCCACCCCUUGCAG

>hsa-mir-6893 MI0022740

CCGGGCAGGCAGGUGUAGGGUGGAGCCCACUGUGGCUCCUGACUCAGCCCUGCUGCCUUCACCUGCCAG

>hsa-mir-6894 MI0022741

CAAGAAGGAGGAUGGAGAGCUGGGCCAGACAUGCUCUUGCCUGCCCUCUUCCUCCAG

>hsa-mir-6895 MI0022742

CAGCUCAGGGCCAGGCACAGAGUAAGCAUCAAUAGCAUUGGCAAGUUGAACUGAGCUGUCUCUCGCCCUUGGCCUUAG

>hsa-mir-7106 MI0022957

GCUUCUGGGAGGAGGGGAUCUUGGGAGUGAUCCCAACAGCUGAGCUCCCUGAAUCCCUGUCCCAG

>hsa-mir-7107 MI0022958

UGCCGUCGGCCUGGGGAGGAGGAAGGGCAAGUCCAAAGGUAUACAGUUGGUCUGUUCAUUCUCUCUUUUUGGCCUACAAG

>hsa-mir-7108 MI0022959

GUGUGGCCGGCAGGCGGGUGGGCGGGGGCGGCCGGUGGGAACCCCGCCCCGCCCCGCGCCCGCACUCACCCGCCCGUCUCCCCACAG

>hsa-mir-7109 MI0022960

GUCUCCUGGGGGGAGGAGACCCUGCUCUCCCUGGCAGCAAGCCUCUCCUGCCCUUCCAGAUUAGC

>hsa-mir-7110 MI0022961

GGGGCUGGGGGUGUGGGGAGAGAGAGUGCACAGCCAGCUCAGGGAUUAAAGCUCUUUCUCUCUCUCUCUCUCCCACUUCCCUGCAG

>hsa-mir-7111 MI0022962

CUGGGGGAGGAAGGACAGGCCAUCUGCUAUUCGUCCACCAACCUGACUUGAUCCUCUCUUCCCUCCUCCCAG

>hsa-mir-7112-1 MI0022963

ACGGGCAGGGCAGUGCACCCUGCAGGUGAGAGCGGGAACACCUGCAUCACAGCCUUUGGCCCUAG

>hsa-mir-7113 MI0022964

CUCCAGGGAGACAGUGUGUGAGGCCUCUUGCCAUGGCCUCCCUGCCCGCCUCUCUGCAG

>hsa-mir-7114 MI0022965

UCCGCUCUGUGGAGUGGGGUGCCUGUCCCCUGCCACUGGGUGACCCACCCCUCUCCACCAG

>hsa-mir-7150 MI0023610

CACGGUGUCCCCUGGUGGAACCUGGCAGGGGGAGAGGUAAGGUCUUUCAGCCUCUCCAAAGCCCAUGGUCAGGUACUCAGGUGGGGGAGCCCUG

>hsa-mir-7151 MI0023611

GAUCCAUCUCUGCCUGUAUUGGCUUGGAUUCUGCAAAGCCUACAGGCUGGAAUGGGCUCA

>hsa-mir-7152 MI0023612

UUUCCUGUCCUCCAACCAGACCAUGCCACAUCCGUCUGGUCCUGGACAGGAGGC

>hsa-mir-7153 MI0023613

UGAGAACUGACAAAUGUGGUAGGUGGUCGAAGUCAUCCACACCAUGGACGGUUUACC

>hsa-mir-7154 MI0023614

UUCAUGAACUGGGUCUAGCUUGGAGCCUUGGUAGGGAAGCAAGCUGAGGAGAUAGGAGGACAAGUUGUGGGAU

>hsa-mir-7155 MI0023615

UCUGGGGUCUUGGGCCAUCUGGUUGUGACAUCACUGAUGGCCCAAGACCUCAGACC

>hsa-mir-7156 MI0023616

UUGUUCUCAAACUGGCUGUCAGAGUGUGCAUGGCAGGCUGCAGCCACUUGGGGAACUGGU

>hsa-mir-7157 MI0023617

UCAGCAUUCAUUGGCACCAGAGAUGAAAUUGGGACUCCUCUGUGCUACUGGAUGAAGAGU

>hsa-mir-7158 MI0023618

GGCUCAAUCUCUGGUCCUGCAGCCUUCUGCCUUUGGCUUUCUGAAGCGAGCUGAACUAGAGAUUGGGCCCA

>hsa-mir-7159 MI0023620

UUCAACAAGGGUGUAGGAUGGCCAGGGAAGUAUGCCGGGCCUUGGCAUUUCUAUGUUAGUUGGAAG

>hsa-mir-7160 MI0023621

UGCUGAGGUCCGGGCUGUGCCCCGUACCGGACAGGGCCCUGGCUUUAGCAGA

>hsa-mir-7161 MI0023619

UAAAGACUGUAGAGGCAACUGGUGUUCUCACGCAAAGUGGCCAGGGUGUGGGAGACUAGAUCUUUGACUCUGGCAGUCUCCAGG

>hsa-mir-7162 MI0023623

UGCUUCCUUUCUCAGCUGAACCCCUGCCCCUGGAGCCCCCAGCAGGGCCCUCUGAGGUGGAACAGCAGC

>hsa-mir-7515 MI0024354

CUUUUAUCAUUUCAUUUAUGCCUGUUAGCAUGAAAAAAGAGGCUGAGAAGGGAAGAUGGUGACAAGG

>hsa-mir-7641-1 MI0024975

UCUCGUUUGAUCUCGGAAGCUAAGCAGGGUUGGGCCUGGUUAGUACUUGGAUGGGAAACUU

>hsa-mir-7702 MI0025238

CUUAGACUGCCAGACUCCCUGAGGACAUGCAGUUUCAGGGAGUCUGGUAGUCUAAGUGU

>hsa-mir-7703 MI0025239

UUAGGGGAGGUGGAUGAGUGGGGGCAGGCAGGGGAUUGGCUAAGAACCUGACUCUUGCACUCUGGCCUUCUCCCAGG

>hsa-mir-7704 MI0025240

CGGGGUCGGCGGCGACGUGCUCAGCUUGGCACCCAAGUUCUGCCGCUCCGACGCCCGGC

>hsa-mir-7705 MI0025241

AAUAGCUCAGAAUGUCAGUUCUGUUUUAAGUAACAGAAUUGAUAACUGAGCAAGGAA

>hsa-mir-7706 MI0025242

UGGAGCUGUGUGCAGGGCCAGCGCGGAGCCCGAGCAGCCGCGGUGAAGCGCCUGUGCUCUGCCGAGA

>hsa-mir-7843 MI0025510

GUAGACAGGAUGAGGGCAGAGCCAGCUUCCUGAUCUGAUGGAAGUCAUGAAGCCUUCUCUGCCUUACGCUUGGCUCUGC

>hsa-mir-7844 MI0025514

UGAUUGUGCGGACUCGUGUGUAAGAAAAACUAGGACUGUGUGGUGUAAAUAACUACAAUUCUCUUAACUCCGUAGCAGUUGCCAACUCAGUCCUUGUACUUCGUUAACACGAAUCUGUUUCA

>hsa-mir-7845 MI0025515

GCAAGGGACAGGGAGGGUCGUGGCGACACUCGCGCCAGCUCCCGGGACGGCUGGGCUCGGGCUGGUCGCCGACCUCCGACCCUCCACUAGAUGCCUGGC

>hsa-mir-7846 MI0025516

GCCCCGCCGCCUGGCCUCUGGCCCGCUGGGGCGCGGGCUUUCGCUUUCAGUCGAGGGCUAGCGAGCGCAGCGGAGCCUGGAGAGAAGGCGCUGGGC

>hsa-mir-7847 MI0025517

GUGUCGGCUGUGGCGUGACUGUCCCUCUGUGUCCCCCACUAGGCCCACUGCUCAGUGGAGCGUGGAGGACGAGGAGGAGGCCGUCCACGAGCAAUGCCAGCAU

>hsa-mir-7848 MI0025518

GCUGGGGCUGGGUGGGUGUGGCAGGCCCACCUUGGGUAUGCAAAGCUCUGACAGUGUUUCACUUGCUACCCUCGGUCUGCUUACCACACUCCCAGUUCUGC

>hsa-mir-7849 MI0025519

GACUUUAUAUUGUUGAACAUCAGACUCAAGGCCAACAACUGACUACUUCGAAAAUGGGAAGUAGACAAUUGUUGAUCUUGGGCCUGAUGUUCAACAAUAUAAAGUU

>hsa-mir-7850 MI0025520

GGUCAGAAGUUUGGACAUAGUGUGGCUGGGUCCUCUGUGCAGGGUCUUACCCAGCCACACUACAUCCAAACUUCUGACC

>hsa-mir-7851 MI0025521

UGGCUCACUGCAGCCUCCCCGCCCCCUCAGGUGAUCCUCCCACCUCAUCCUCCCAAGUAGCUGGGAAUACAGGUGUGUGCCACCAUGCCUCUACAAGCUACCUGGGAGACUGAGGUUGGAAGAUUGCUUGAGCCUAGGAGGUCGAGGCGACAGUGAGCCA

>hsa-mir-7852 MI0025522

UAAAUGCCUUUGACUACUACAUACAUAUGCCUACUACUACUUUCAUAUGUAGGCAUAUGUAUGUAGUAGUCAAAGGCAUUUA

>hsa-mir-7853 MI0025523

UCUCACACUUCAAUAGGCAUUGUCUGGGGUCUUAUUCAAAUGCAGAUCCUGACUUCACUGGUGCAGUAAGAAGUCAGAGAAUCUGCAUCUGUAACAAGCUCCCAGGUGAUGAUGGUGCUGCUGGUGUGCAGA

>hsa-mir-7854 MI0025524

UUCCUUCCAUCUCCAUCACCUUGAGCAUCUUCUGGGCAGCUGAGGUGACCGCAGAUGGGAAGGAA

>hsa-mir-7855 MI0025525

GCUUGGUGAGGACCCCAAGCUCGGCACUAUUGUGACUGUGCUUUUGGCUUCCUUUCCAGGC

>hsa-mir-7856 MI0025526

UGUUUUAAGGACACUGAGGGAUCCUUUGUUCUGGAUCCCUCAGUGUCCUUAAAACA

>hsa-mir-7973-1 MI0025748

UGUGACCCUAGAAUAAUUACCCAACGUCUCUGAGUCUCGGUUUUGAGAGGGUGAGUAAUUAUUCUAGGGUCACACA

>hsa-mir-7973-2 MI0025749

UGUGACCCUAGAAUAAUUACUCACCCUCUCAAAACCGAGACUCAGAGACGUUGGGUAAUUAUUCUAGGGUCACACA

>hsa-mir-7974 MI0025750

GCUCGGCCCCCACAGCGAAACGGCCGCCUAAACCACCCAGGCCUUAUGGCUUCAUAGGCUGUGAUGCUCUCCUGAGCCC

>hsa-mir-7975 MI0025751

GUGCAAAGAGCAGGAGGACAGGGGAUUUAUCUCCCAAGGGAGGUCCCCUGAUCCUAGUCACGGCACCA

>hsa-mir-7976 MI0025752

UGCCCUGAGACUUUUGCUCUAAUAAUUUAUUCUAAUAAUAAUUUAGAUCAAAAGCCUCAGGGCAGA

>hsa-mir-7977 MI0025753

UUCCCAGCCAACGCACCAAAAAUGAUAUGGGUCUGUUGUCUGGAGAAAC

>hsa-mir-7978 MI0025754

UCUGGUGUAUAGCGUUGCUCAAGUCCUCUAUUUCUUUGAACAACAGUAUACACCAAAUA

>hsa-mir-8052 MI0025888

UGGAGGGCUGCGGGACUGUAGAGGGCAUGAGCUCAGGAGCUCAGGCCAGCUCAUGGUGCAAGGCCUCUG

>hsa-mir-8053 MI0025889

GCUUUUCCACUGGCGAUUUUGGAACUCAAUGGCAGAAAUGUCAAGAAGAGUUUUAUCCUUUGCCGAAAGAGAAAA

>hsa-mir-8054 MI0025890

CAGCAAUGCAGAAAGUACAGAUCGGAUGGGUUCAAUAGAUUGCUUUAGCUGAAGCAAGCCUUCCCAUUGUACAUAUCUCAUUGUCU

>hsa-mir-8055 MI0025891

UUACGGUCACACUGUGCUCACUUUGUCAAAUAAAAGAUGGAGUCGUUUGACGUCUUUUAACACCCUUUGAGCACAUGAGCAGACGGAGUGAUCAUGA

>hsa-mir-8056 MI0025892

CUUCUCUGAGCAUCCAGCCUCUAGGAGCUGUGCCUCCUGGCCUAUUCCUGUUCGUGGAUUGUCUGGAUGCAUCCUUGAGACU

>hsa-mir-8057 MI0025893

AAUUUGGGAUGUGGCUCUGUAGUAAGAUGGACUUGCGGUUCAAUCCCAGCUCAGGGCCUUUUUAAAUUA

>hsa-mir-8058 MI0025894

UGGUCACUGCCAGUGCUGAGACAUUAAAGAUCCAGGCCCCACGGAGAACCAGGGCACCUGGACUUUGAUCUUGCCAUAAUGGUAUGCCU

>hsa-mir-8059 MI0025895

UACAGGUGCAGGGGAACUGUAGAUGAAAAGGCUUGGCACUUGAGGGAAAGCCUCAGUUCAUUCUCAUUUUGCUCACCUGUU

>hsa-mir-8060 MI0025896

UUUAAAUCCUGUUUUUCCCACUUACUAUUCUGGUCAGAUAUCCCAUGAAGCAGUGGGUAGGAGGACAGGAAAAAGC

>hsa-mir-8061 MI0025897

GAGAGGAUGCCUUAGAUUAGAGGAUAUUGUUCAGUGGGAUUUGGAGAGGACGCCUUAGAUUAGAGGUAUUGUUCA

>hsa-mir-8062 MI0025898

CUUAUGCAUGCAGUGAUUUGAGGAUUAUUGCUCACGGUAAGAAAAACAUGAGUAAUAUAUUGUCUUCAAAGAACCUAUGCUCUAA

>hsa-mir-8063 MI0025899

UAGAGGCAGUUUCAACAGAUGUGUAGACUUUUGAUAUGAGAAAUUGGUUUCAAAAUCAGGAGUCGGGGCUUUACUGCUUUU

>hsa-mir-8064 MI0025900

AGAUGGAGUGAGCACACUGAGCGAGCGGACAUCCUUCCAUAGUAAAUAAGCGGUGCCGCGCGCUCCGCUGGCUCCCAAACACUCCAGUCC

>hsa-mir-8065 MI0025901

CAGUACAUGAGAGCCAUCAUUUUUGUUGUUGUUGUAUUUUUCAGCAAGGCAAGGGCUUUGAUGUCUUGUAGGAACAGUUGAAUUUUGGCUUCAUGUAAUU

>hsa-mir-8066 MI0025902

CAUCUUGCUUACAUUCCUGUGGAACAUAUUGUUGCCUCUGCAUGUUACAAUGUGAUCUUUUGGAUGUAGGGCAAAUCU

>hsa-mir-8067 MI0025903

UUCUGUGUUUCCUAGAAACUGUAAACUUAGUCAUAAAGAUGAUCCUGAGUGCACAGUCUUUCUUGAGACUUUCAGCC

>hsa-mir-8068 MI0025904

GGCUAACUCAUGUUUGUUGUAAGGAUCGUUGUGGCAAUACAGUUAUCCUUACAACAAAUACUUUGCCA

>hsa-mir-8069 MI0025905

CGCCUGAGCGUGCAGCAGGACAUCUUCCUGACCUGGUAAUAAUUAGGUGAGAAGGAUGGUUGGGGGCGGUCGGCGUAACUCAGGGA

>hsa-mir-8070 MI0025906

ACUUGAUCCCAUGUGAUUGACGGCUGACUCCAUGUGAGUGAAGCCUGAUCAUCAUGUGAGUGAAGCCUGAUCAUCAUGUGAGUGAAGC

>hsa-mir-8071-1 MI0025907

CGGCCACAUGGCCCAGGCUCUUCUCCGAGUGAUCUCGGUGGACUGGAGUGGGUGGUAGGUGGCAG

>hsa-mir-8072 MI0025908

GCGUCAAGAUGGCGGCGGGGAGGUAGGCAGAGCAGGACGCCGCUGCUGCCGCCGCCACCGCCGCCUCCGCUCCAGUCGCC

>hsa-mir-8073 MI0025909

GAUUUCAGUGACCUGGCAGCAGGGAGCGUCGUCAGUGUUUGACUGUUUAUGGUAUGUCAGGGAGCUGGUUCC

>hsa-mir-8074 MI0025910

CCAGUUCCUGAGUUUAUGCAAGAUGCCCAUGGGAGCCCAGAGACGUCCUAUGGCGAGACUGGCAUGUACUCACACAACUGA

>hsa-mir-8075 MI0025911

CCUUGCUGAUGGCAGAUGUCGGAUCUGCCUCGCUUAUACGUGCCCUUGCUGAUGGCAGAUGUCGGGUCUGCCUCGCUUAU

>hsa-mir-8076 MI0025912

CCUAUCCACACGUCUGGUCUAGAGUUCUCCUGUGAGAGCUACUAAGUCUUAUAUGGACUUUUCUGAUACAAUGUAGUGGAAAG

>hsa-mir-8077 MI0025913

GUGGGGCAGGGGGUGGGAUGGGCACUGGGAGUCCAAAGGAAAGGGCUGAGUGGGGUUCUGACUCCUCUGCCCAAC

>hsa-mir-8078 MI0025914

UCCGCCCCGCCCCCUCCACCGGGCUGACCGGCCUGGGAUUCCUGCCUUCUAGGUCUAGGCCCGGUGAGAGACUCCACACAGCGG

>hsa-mir-8079 MI0025915

UGCCUGGGUUCAGUGAUCGUCUCUGCUGGCUGUAGGGCAGCAGUCAGAGGGGACAUCACAGGCCUCCAGAGG

>hsa-mir-8080 MI0025916

AUGGCUCUAAGAAGGACACUGGUGUCAACGGCUGAGCCCUUUGAUAAGGGGGAGGUUUGGAAGACACUUAUGUCCUGGAUUGGGAAAUG

>hsa-mir-8081 MI0025917

UUAUUGUCUUUAUUUUCUGAGUCUUGCCUCUAUGGCCCCCCUCCUCUCUGAAAGCAAGGCCACUUGAGUCGUGCCUUUCUGAAUGAAGUCAAUGU

>hsa-mir-8082 MI0025918

GCCUGUGUGAUGAUGGAGCUGGGAAUACUCUGGGGAGAGAGUCCUCUUUUCAGCUGUAUUUUGCUUCCUUCCCACACAGAC

>hsa-mir-8083 MI0025919

CGAGGGGGUGCAGGACUUGACGGCUGCAACUGUGCGGCCGGCCACACCGUCACACCGGGUGCAGCGUCUGUCAUUCUGUACCCUCAUCU

>hsa-mir-8084 MI0025920

CAGAUAAAUAGAAUACUAAGUAAAAAAUCAGUAUGGAUUAAAUUUAUAUUUUCCUCGAUACUGAGUAUUUUUUCUUAGGUUUAGUUUGG

>hsa-mir-8085 MI0025921

CUAGGAGGGAUGGGAGAGAGGACUGUGAGGCAUGGGUGGCUCUAUGGUCACGCCCAUCUUCCUAC

>hsa-mir-8086 MI0025922

UCCACUAGUCUGCUAGUCUGGACUGAUAUGGUUAGCUUUUUUUUUUUUUUGAGAUGGAGUCUGGCUCUGUUGCCCAGGCUGGAGUACAGUGGC

>hsa-mir-8087 MI0025923

UCUAAGAAGUGAAGACUUCUUGGAUUACAGGGGCCCUACUUUAAGGGCCCUUUCAGUUGGAAGUUUUCCUUUCUGCCU

>hsa-mir-8088 MI0025924

GGGAGGAGGGCCUCGGUACUGGAAAGGGGUAUGGAUUCUGUUGUUUUGGCAACAUUCUUCUUAUCGGCACUGGGUCACUCAGCUCA

>hsa-mir-8089 MI0025925

AAGGAGCACUCACUCCAAUUUCCCUGGACUGGGGGCAGGCUGCCACCUCCUGGGGACAGGGGAUUGGGGCAGGAUGUUCCAG

(2) 1612 false human pre-miRNAs

>random_seq_from_cds__NO_6

GAGGGCAGGGGGCACAGUCCAACUCCAGGCUUGUAGCUGUCCAGGGGCUGGGUGCCCGCCCGGCAGCGGCAGACUGUGUCCUGUGUGGCCGUGCACA

>random_seq_from_cds__NO_16

AAGCACCAGAGACGAACAGUCUGGUGUCAGGAGCAAGAGAAAGGCUCAUGACAUCUCCAGUGUGUCCGGUAAACGUGGUCG

>random_seq_from_cds__NO_21

AUGGCGUAGAUCUUGGCCAGGUGCCCCCGCAGUGUCCUCCUCGUGCGCAUUUGGAUUCUUCCCACUGGGUCGAUGUUGUUUGUGAUCCUGAGAGAGAGUUGCAUCUGCAC

>random_seq_from_cds__NO_41

CCACCAGAUCCGCGCCACGCUCCCGCACCAGUGCGGCCAGCCCCUUAUCCAGGCGGCCUGAGGUCGAGGCACGGGGAGCUGGAGCCCGUCUGCACC

>random_seq_from_cds__NO_44

CAAAGGGGAAGACUCCCAAAAUGAUGUCUAUUCCACUGUUAUCUGCGAAAAUUAAGGCACAUUUAUGAGGGGGCCCCCUUUAAUCU

>random_seq_from_cds__NO_49

GGGUCCCAUAGGCGCAAGGGCUGCUGCCUCAGGGUCUGAAGCUUGUUCCAGUACUUCUGCCGGAACUUCUCCGCCCUCUCGGCUGCAUCCACAGAGUCUGGCUGGCUCGC

>random_seq_from_cds__NO_65

UUGGCGACUUUGUGCUGUACCGUUGAAUAGUAGGCCAGCUUGGUUAACGACCCGCCCUAUGUCGAUGGCGAAGCG

>random_seq_from_cds__NO_71

CACCUUGGUCUGGUGCUGACAUUCCUCCAGGGUCCCAUUGGGAGAGAAGGUCCCCGGGGGGCAGUUCUGACACAGGGUGUCCUGACUCU

>random_seq_from_cds__NO_76

AGGACGGCAGAGCUGGGGCGUAGCAGGGGGCUCCCAGGAAGGUGAGAUACAGCACCCAGCCUCAAGACGUCGGUUUUG

>random_seq_from_cds__NO_80

GCAUAGUUGAUGAAGAAGAGCUGCUCAUGGGUGAGAUCCAGGCCGGGCAGCUGCUGGUCCUUGCCACCCU

>random_seq_from_cds__NO_86

GCGCCCACCUUGAGGUUCUGCAGACUGUUCUCAAAGUACAGGUCCUCUGAGAAGUUCAGCAUUGGAGUACUCCUCGUCCAGGCG

>random_seq_from_cds__NO_93

AGAGUCCAGUUAAAUCCCCUUCAGGCCAAACUGGCUUUGCAGCUCCUCCAGUCCCAUCCGGUGGUACAAGGCGAUGA

>random_seq_from_cds__NO_112

UUCAGCAAGGUCAAGGUCCCUCCCACUGAUCUCUGCCUCCCGGUGGCUGCUGAAGCGCGGAGAGCUGUUCUUAGUGGAAGAAACCGGACUUUUUCGAAAAAUUGCCAGCC

>random_seq_from_cds__NO_117

GCAGGCGGACGUGGUCCUGGUUCUGCAGCAGGAGGAUGGGGUGGCUCUAUGGCGAGAGGCUCCGGGACGGAGAGACGGGAUGGUUCCC

>random_seq_from_cds__NO_129

AUGCUCACGUAAUCUUUGCAGUCGCGCCGUUCUGCCAGCGCCAUGUAGCGGCCGUCCCUGGUGAAGGUGAUUCCCCUGCAGACAAGCUUUCGGG

>random_seq_from_cds__NO_130

UAUUUGAUGUAAGACACGGAUUUUGUGCACAAGGACCAGACGGUUAUCCGCAGCAUGGAAUUCCGUGGUGUUGAGAAUGUGGCGC

>random_seq_from_cds__NO_132

CCUGCACCAGCCCUCGCUUGUACAUGGCGCACAGGAUGAAGAGCGAGUCUGCCGACCACUCGAUGUGCUGGAUCUGGUCUAGGCACGUGUACAGCUGAAGGAUCUGAAGG

>random_seq_from_cds__NO_135

UUCGGCGUGGCUGGCCGGAGCUGCCAGGAGGUGCUGCGCAAGGGCUGUCUCCGCUUCCAGGCUCCCUGAGCG

>random_seq_from_cds__NO_140

UGGAGUCCCGAUUUCAGAGCAAGUCUGGCUAUCUGAGAUACAGCUGUGAGAGCCGGAUCCGGAGUUACCUGAGGGAGGGUGAGCUCCUA

>random_seq_from_cds__NO_149

CGGAGAGUGUCCCUGGCUCUCUCGCAGGGGCAGGCGGGGUCACAGAGGAUGCUCCCGGAGAGAGGUCGGCU

>random_seq_from_cds__NO_153

UGCAUAUGUCACAGGUCCUUUGUCCACCUGCGCUGGAGAAACUAUUUGGAGGACAGGGACUGCAAAUCUG

>random_seq_from_cds__NO_159

UCACGGCCUUGGCGAUCCUCUUUGGGUUGGUCACUGUGAGAUCAUCCCCCACUACCUGGAUUCCUGCACUGGCUGUGAACUUCUGCCAAG

>random_seq_from_cds__NO_187

ACGGCCAUGACACUGUCCUUGUGCUCUCGCAGCACCUCCAUCACUGUGUGGCAUGUGAUUCUGUAGUUGCCAUCCAG

>random_seq_from_cds__NO_196

UGCAAAGACGGUGCUAUGGACUGAAUGCGAAUGAUUGGCUGGUUGGGGUCAUAUGUUCCUGGCACAGCCAAUUCAAGGUCCCGGCACAUCAGAAGUU

>random_seq_from_cds__NO_197

UUGGGGAAACAUAUUGCAGCUCUAAGGAUGUGAGCCUGAGGCAGCUGCUUUGAGAUUCGUCGGAACACAU

>random_seq_from_cds__NO_198

GAUAAUAGAGGUCCCAGGCUUGGGUGAGGUCCUUGACAUUCCCUGAUUUCAUGUACUUCCUGCACCACUCUUGGGCCUCCAUUA

>random_seq_from_cds__NO_204

UAGCCAGGUAUCAAUCUGGAUGGCUUUCACCCCCUCCACUAAGGCCUCAUUGACAUCUGGCCAGUGAC

>random_seq_from_cds__NO_207

CCCUCGGUGCUGGCAGUGGUGGUGGCAGUGGCGGCCGUGGUGGCGGCAGUGGUGGCGUUGGUGAUGUUGGCCCCGCUGGC

>random_seq_from_cds__NO_219

UUGGCUUGGGUCUCAUCAUUAACCAGGGUCCACUUUUCACAGCACUGCUGGUGGAGUUGACCCCCAUUCCCCCAAGGCCUCG

>random_seq_from_cds__NO_222

CCGGCCGCUGCCUCCGGCUGCUGUAGCUUAUUAUUAAUGCCUGAUGAGAGAUUCUAGAAUGGCAGGGGUGGGGCCUUU

>random_seq_from_cds__NO_239

GGUAGAACUCAUCCAGAGGCAAGUUUCCCAUGUUGACCAGCAUUUCACUAGUGCUAUAGUCAGCAGGAAUCCUGACUUGACUUGGAUUCU

>random_seq_from_cds__NO_242

AAGUAGGGUACUUCCUGUAGGGCUCUACUACAUAGCCAGUGCUGGCCACCAACUGUCCCAGGGUCCACAGAGCCACCCUGCCUUUUG

>random_seq_from_cds__NO_256

UUGCUCGAGCCAGCAUGCUGAUGCAAGUGAAGACUGUGGCAUCCACCUGCAUUGCCUUCUGCCUCCUUAUGGGCGAAGUCCU

>random_seq_from_cds__NO_261

CCGGCUCUCCACCAGGGUGGACUUAGCUGGACUGGGGGAGGUCCCAAAUCCCAUGAGGCCUUGGUGAGAGCUGUACCCCAGCAG

>random_seq_from_cds__NO_275

GCUCGAAGCGGACGAAGCGGACGCCCGGCCCGUAGUCGGUGAAGGUGUGGGAGAUCCUCCAUCCAGCCCCCGCCGUCACUGUCUUGGGGCACUGCCACCUGCCCGCUGCU

>random_seq_from_cds__NO_280

GCCUUCCAAGUCCUCCUUCCCCACACGGGUUACGCAGAAGGUUGCGGCGCCGCUUGCUCAGGAAGUAGAACUG

>random_seq_from_cds__NO_289

UUUUGUCACAUCCUACGAGAAUGUGCCUCAAGUCCCAGCUGGUGGACCUUGUAGCCGAGGGCUACUGGGAGGAGCUACUAGACACAUUCCGGCCGGA

>random_seq_from_cds__NO_292

CCUCUUCCAGCAUGGGGGCAGGGACACCCAGUACUGGGCAGGCUGGUAUGGGCCCCGAGUCACCAACAGCAGCAUUGU

>random_seq_from_cds__NO_310

UCCCAAUGGGCCCCAGCCCCCCAGCUGAAGGGAGCACUGGCGACUUCGCUCUUCCAGUUGGGACUGAUUGUGGGUGUGACAGCCUUGGG

>random_seq_from_cds__NO_318

UGGCCGCGUGGAAAGUGUCAAAAUUCUUCCCAAGAGGGGAUCUGAAGGAGGAGUGGCUGCCUUUGUGGAUUUUGUGG

>random_seq_from_cds__NO_336

UUUGACCGCUUAAAAGGCAUGGCCCUGGUUCUCUACAAUGAAAUUGAAUAUGCACAAGCAGCUGUAAAAGAGACCAAAGGGAGGAAAAUCGGUGGGA

>random_seq_from_cds__NO_349

CUGCCCUGGACCAGAAACUUCAGGUCUCUCAGACGGAGCCUGCAAAAUCUGACUUGUCUAAACUGGAAUCA

>random_seq_from_cds__NO_351

AGGAAGCACCUCAAGCCUGAGCAGCCUGCAGAUGGGGUAAGUGCUGUGGAUCUGGAGAAGCUGGAAGCCAGGAAAAGGCGCUUUGCAGAUUCCAAUUUA

>random_seq_from_cds__NO_398

CCAUUCCACUCCUCCUCAGUCAUGUACUUCUGACCUAAGCAAGAUUCCCUCCACAGAGAAUUCGUCCCAAGAAAUCAGUGUUGAGGAAAGGACUCCAACCAAAGCAUCUG

>random_seq_from_cds__NO_410

CGGGUGCGGUUACUGCUGCAUCUGGUGGUGUAACGGCCACAACAGGCACGGUGACAAUGGCAGGGGCAGUGAUUGCGCCGUC

>random_seq_from_cds__NO_424

UCGCUCGAGCCACAGCCCCUGUGCAGUCAGAGGUACUAGUCAUGCAGUCUGAGUACCGACUGCACCCCUAUACUGUGCCA

>random_seq_from_cds__NO_440

CAGAUCUUCCCGCCCUGUGAGUUCUCUGAGAGUCACCUGUCCCGCCUGGCCCCUGACCUCCUUGCCAGCAUCUCCAACAUCUCUCCCCACCUCAUGAUUGUCAUUGCCUC

>random_seq_from_cds__NO_441

CGUGUGAGAUGGAGGAGUUUGUGGGGCUGCGUGAAGGCUCCUCAGGGAACCCUGUGACUCUGCAGGAGCUGUGGGGCCCCUGUCCCC

>random_seq_from_cds__NO_450

AGACCAGUUUCCGGGUGGACGUUCCCUUCGACCUGCCUGAGAUCUUCUUUUUUGUGGCGCUGGGGGGGUCUCUGUGGCAUCCUGGGCAGCGCUUACCUCUUCUGUCAGCGAAUCUUCUU

>random_seq_from_cds__NO_468

AGUUUCUCACGCUCUUCUAUGGACUGCAGAUACUGCUGCUUGCCUUCCUGAGAUUCAUCCUUCUUCUUCAAAUAAGGCUCAAUGGAUUUGUACUGUGCAUAGAAGUUGCU

>random_seq_from_cds__NO_481

GGUCCCAAGUGGCCCUCUCACUACCUGAUGGUCCCCGGUGGAAAGCACAACAUGGACUUCUACGUGGAGGCCCUC

>random_seq_from_cds__NO_487

UGGGAACCUGGAAGUGAGCCCCCCAGUCACAGUCAGGGGCAAGGAAUACCCGCUGGGCAGGAUUCUCUUCGGGGACAGCUGUUAUC

>random_seq_from_cds__NO_489

CCCUGUGAAGCUCUAUUCUGACUGGCUGUCCGUGGGCCACGUGGACGAGUUCCUGAGCUUUGUGCCAGCACCCGACA

>random_seq_from_cds__NO_501

UCAGGCAGCUGGGCCUGCCCACCGCCCUCAAGAGAGGGUGUGGUGACUCUGCUGUCUGACUACGAGGUGUGCAAGGAGGGC

>random_seq_from_cds__NO_502

GAUGUGCUGACCCCAGAGCAGGCUCGCGUCCUGGAAGCUUUUUGGGUAUGAGAUGGCUGAAUUCAAGGUGACCAUCAAAUACAUGUG

>random_seq_from_cds__NO_509

UCCAGCAAAGCCAUGAUCCAGAUCCCAGAGGUAGACAGAUGAGACGCCACCUUCAAAAUACCAGGUCUCGAUACUGGUCAAAGGCAUUG

>random_seq_from_cds__NO_512

CUGGUCAACAGAAGACAGGAGAUCCUCACAUAGACUGGGGACCCAGGUCGAUCAGGUCGCUGAGGUUUUUCUCGAUUUGCUGGG

>random_seq_from_cds__NO_516

CCUGGGGAGGAGACCAGGCUGGAGAUGGACAAGUUCCCCUUCGUGGCCCUCUCCAAGGACGUACAACACCAAUGC

>random_seq_from_cds__NO_521

AAGAAUAAAACUGAUGUGGAGUAUGAGAGUGACGAGAAAGCCAGGGGCACGAGGCUGGACGGCCUGGACCUCGUUGACACCUGGAAGAGCUUCAAACCGAGAUACAAGGC

>random_seq_from_cds__NO_522

ACUCCCACUUCAUCUGGAACCGCACGGAACUCCUGACCCUUGACCCCCACAAUGUGGACUACCUAUUGGGGUCUCUUCGAGCCAGGGGACAUGCAGUACGAGCUGAACAG

>random_seq_from_cds__NO_529

GGCCCCAUGGCGCACCUGCUGCACGGCGUCCACGAGCAGAACUACGUCCCCCACGUGAUGGCGUAUGCAGCCUGCAUCGGGGCC

>random_seq_from_cds__NO_551

UGGAGGUGGUGCUCCCCUCCCCCUUGGUGGUGGUGGAGCACCUCGCCCUCCCCUUCCUCCUCCUCUUCCUCUUACUGCAUAGCCAU

>random_seq_from_cds__NO_553

UAUCCACCUCUCCCCCCACCACGACCCCGACCUCUAAUUGGAGGUGGCAUGCGAGGAGGAGGGUGGUAGUAAUAAUCUUCAUACCG

>random_seq_from_cds__NO_567

GUCUGAAAUAUUUCAUCAAGUCUUUCUGCCACCUUCUGUGGGAGGCCUGCCUCUAUCAGUGUCUUGUAGUGUUCUGUGUGAGUU

>random_seq_from_cds__NO_578

CUGGGGUUGGGAAAACAGUAAAUAGCUUGCGAAAACACGAGCAUGUUGGAAGCUUUGCCAGGGACCUAGUG

>random_seq_from_cds__NO_595

CCCUAAAAUGAUGACCUUGCACCAGCAAUGCAUCCGAGUACUUAAAAACAACAUCGAUUGCAAUCUUUGAAGUGGGAGGAGUCCCAUACU

>random_seq_from_cds__NO_605

UCUGUCCCCAUGCGGCCUAGGAUCCCUGUGACCCUCAACAUGAAGAUGGUGAUGCCCUCCUGGGUUUGACCUGAUGGGGCUGAGUCCA

>random_seq_from_cds__NO_611

GCUCCUGUCCUCAGGGAGAUGGCAGCUGUGAAGGAAUUUCUUGAGAAGCUGCUGCCUCCUGUCUAACUCAGGC

>random_seq_from_cds__NO_620

ACACCAGGUUCUUCACCCCGUGGGCCUUCAUGAUCCUCCAGAAGCUGGAUGGUCCCGGUCAGGUUAACUCUGUAAUAA

>random_seq_from_cds__NO_626

AGGCUCUUCAUGAUUCUUUGGCUCAAAGGAGUUGUAUUUAGUGUGACGACUGUUGACCUGAAAAGGGAAGCCAGCAGACC

>random_seq_from_cds__NO_639

AGCGGCACAGAGCAGGAUGACCUCUUCAGCUUCUGACCUACAGGCUGGACAGAUGGUGGGGGGCCCCUGGG

>random_seq_from_cds__NO_659

UCCUGAGGAUCGUGCAGCCACGUGGCUGGGAUCUGCCACAACAUCCUGGUCUGCUGCCCCAAGGAGCUGCUGGAA

>random_seq_from_cds__NO_661

ACCCAGGGUUCCCACUGACUCCCGACUGCAGAUCCAGCCUGGAGACGAGGUUGUCCAGAUCAACGAGCAGGUGGUGGGUGGGAUGGC

>random_seq_from_cds__NO_669

CCAUGAUGUGUACAAACCCUUCAUCUUCGCUGCUGAUACCCUGACAGAUCUGAGCAUGGUGGGUGCGUCA

>random_seq_from_cds__NO_673

CAGUGAAGAGGCACUGGAAGGAAUGGUACGGGGGCUGAGGCAGGGUGGCGUGUCCCUCCUAGGCCAGCCACAGCCCCUGACCCAGGAACAGUGGCGGAGCUCUUUCAUGC

>random_seq_from_cds__NO_675

CUGCAGGUCCUAGAAGAAGUGCUGGGUGACCCUGAGCUGACAGGAGAGAAGUUCCGCCAGUGGAAGGAGCAGAACCGGGAGCUGUACUCAGAGGGCCUGGGGGCCUGGGG

>random_seq_from_cds__NO_682

CAUGGAGCCUUGUGGAGGGGUGCUGGCUGGUUCCGGUUUUAUGGAUGAGAAAUUGGAGAGGUUGAUUAGGGCUGAGGGGCCAAA

>random_seq_from_cds__NO_698

CCAGGUCUGUGGGCCGCGUCCGGCAGAGAUGGCCCUCUGGACUGGGGGGCGAGGCCAGGGCUGCACAGGGCC

>random_seq_from_cds__NO_710

GACCGCUGGUAUGCCAUCUGCCACCCACUAUUGUUCAAGAGCACAGCCCGGCGGGCCCGUGGCUCCAUCCUGG

>random_seq_from_cds__NO_713

ACUGGGCCUCAUGGCCAUGGCCUAUUUCCAGAUAUUCCGCAAGCUCUGGGGCCGCCAGGAUCCCCGGCACCACCUCAGCA

>random_seq_from_cds__NO_714

CUGGUGCGGAACUGGAAGCGCCCCUCAGACCAGCUGGGGGACCUGGAGCAGGGCCUGAGUGGAGAGCCCCAGCCCCGGGCCCGC

>random_seq_from_cds__NO_716

UUCGCCCUCUGCUACCUGCCCAUCAGCGUCCUCAAUGUCCUUAAGAGGGGUGUUCGGGAUGUUCCGCCAAGCCAGUGACCGCGAA

>random_seq_from_cds__NO_717

GCUGUCUACGCCUGCUUCACCUUCUCCCACUGGCUGGUGUACGCCAACAGCGCUGCCAACCCCAUCAUCUACAACUUCCUCAGUGGGCAAAUUCCGGGAGCAGUUUAAGG

>random_seq_from_cds__NO_720

AUGGGUGGGUACUGCUGCUCCAUCGGCAUGGCCCCAAAGCAGUCAGAGGGAUUACAGUGGCCAG

>random_seq_from_cds__NO_723

AAGCCCUGCCGUCCUUCUCUGCCAAUCUGACCAGGGAGCCCAGGUGACCCUGGAGCACCUGGCCUGCCCGGAGCACCAUCCUUCCCUGGAGGCCCUGGUCGUCCCGGAGC

>random_seq_from_cds__NO_725

GAUUUCAUCAUAAUUCACCAUGUCUCCCCAUGGAGCCAGGCACACCAGGCAAGCCAGGGCUCCCCGAAGGCCCCGGAGCUCCAGGGUGGC

>random_seq_from_cds__NO_728

CCCCUCUUUGCCAGCUGCACCAUCCGUACCAGGUUCGCCCUGGGGGGCCAGGGUGUCCAGGGGGGCCGGGCUGGCCUGGGAGGC

>random_seq_from_cds__NO_739

CCCGGAGGACCAGGUAGGCCUGGGCUCCCAACACAGCCAGGAUCUCCCUCACUGUUGUCACCCCCGGGCCUCCUCGGC

>random_seq_from_cds__NO_763

UGGACCAGGCAGGCCCCGCUCACCUUUCCCUCCCCUUGGGGCCAGAGGGAGCAAGUGUGACCAUUGCU

>random_seq_from_cds__NO_766

UAGACCUUGCCUUCAGACUGUGGAUUGAUCUCAAUGAGCUCAUUGCUCUGGGUGUCCCGGCGGGCCUUGGAGG

>random_seq_from_cds__NO_790

GGACGGCAGACAUCCAGGUGAGCGCCAGCAGGGGCAGCACCACGCAGGAGCUCCAGAGUGAGGCCCAUGGCGUUCCUGGCCGAGGCUGAGCUGAGCAGG

>random_seq_from_cds__NO_792

CGGCCCUCUGCUUCUUGGAUUUGUCGGAGAUGCCAUCACGUGCCAUGAGCUUGUUGAAGACGAUGAUUCCGAUGAGCAUG

>random_seq_from_cds__NO_821

CAGCCGCCGGGUCGCCCUGUCUGCGCCAUGUAUAGCCCAGGCUCAUCUGCAGACCUCGGCCACUGGGUUU

>random_seq_from_cds__NO_830

CGAGAAGGCCCCGUAGAGCACACCCGAGGCCAGGGCAGAGCAGGCACUGGGGGCGGGGUCGAAGGCGGUGGCCAGGCGCAGGGAC

>random_seq_from_cds__NO_834

UCUUAUGUGAGCUCUACCACUGGGGGAGAGUGUGAUCACAAGAGAGAUGGUGGAGAUGCUCUUUUCUGAUGAUUCUGACCUGCAGUUAGCAACCACACAGAA

>random_seq_from_cds__NO_848

CGAGAACCAGGAGAUCUACCAGAAGGCCUUCGACCUCAUUGAGCACUACUUUGGUGUAGAAGACGAUGAUAGCAGCCUGGCUCCCCAAGUCGAUGAAACG

>random_seq_from_cds__NO_854

GUGAAACUUAAUAAGCAGCAGACGGACAUCGCUGUGAAUUGGGCUGGGGGCCUGCACCAUGCAAAGAAGUCCGAGGCAUCUGG

>random_seq_from_cds__NO_855

CUUCUGUUACGUCAAUGAUAUCGUCUUGGCCAUCCUGGAACUGCUAAAGGUAUCACCAGAGGGUGCUGUACAUUGACAUUGAUAUUCA

>random_seq_from_cds__NO_870

CCAAGGCCCUCUUCCGUCAGAUGGUUGAGGCCAUCCGCUACUGCCAUGGCUGUGGUGUGGCCCACCGGGACCUCAAAUGUGAGAACGCC

>random_seq_from_cds__NO_872

GUACAGCCUAUGCUGCCCCCGAGGUGCUGCAGGGCAUUCCCCACGAUAGCAAAAAAGGUGAUGUCUGGAGCAUGGGUGUGGUCCUGUA

>random_seq_from_cds__NO_881

ACAUCCUUCUAAACCAGGAUCCUUCUGGAGAGUGCAACCCAGACUUGCGUCUCCGUGGACAUCAGAAGGAAGGCUAUGGGC

>random_seq_from_cds__NO_884

CUGGCAUCUACUCCAUGAGUCUCUGUUUGGGUCAGUUGCUGAUGAUCAGAAACUUAUGAUGUUGGGAUA

>random_seq_from_cds__NO_885

CUCGUUCAAACAAUACUUCCAAACCAAGCCACUCAGUUGAUGCUCACACUGCUGAAGUGAACUGCCUUUCUUUCAAUCCUUAUAGUGAGUUCAUUCUUGCCACAGGAUCAGCUGACAAG

>random_seq_from_cds__NO_894

UUGACACCAACAGCGAUGGCACCAUAGACUUUCGGGAGUUCAUCAUUGCGCUGAGCGUGACCUCGCGCGGCCGCCUGGAGCAGAAGCUCAUGUGGGCCUUCAGCAUGUA

>random_seq_from_cds__NO_905

UAUCAUCUGUUUUGAGGUGCUUAGGUGAAGGAGAAGUCAUGAAAGAUGGACCCCUUUUCUUUACCCUGAGACUUAGACGAUU

>random_seq_from_cds__NO_906

CCUUUGCCGCCUCAGCCUUGACAGGAGAAAGUGUAUGAAAGACAAAGUUUCUUGAAUUUCGAGGAGCACUGG

>random_seq_from_cds__NO_907

GGUUAUGGUCAGAGAGAGCAGCCAGUUUCUGAAGCACAGCUUUGGGCUGGUUUAGCAGUGAGCCUGUCUUCACCCUGUU

>random_seq_from_cds__NO_939

UUGUCUGCUCAGAACCUUUACUAUGGUGAUCACUGUUCAUUUCAGUAGUAUUUGCAGUGUCUAUGAUUUCUUUGUGAUGGCUUGACUGAUAUUUAGAUGACCUUCAAUAG

>random_seq_from_cds__NO_946

CUUUAUCAUGUAUGUGCUUUUUGGAACUCUUUCUGUCAGUGGUAAAGUCUGUAGAGUUUCCAGACUGAAGACUCAGCUCUAAGCA

>random_seq_from_cds__NO_953

CUGGCCAAUUACUUUGAGGUGGACAUCCCUAAGAUCGACGUGUACCACUACGAGGUGGACAUCAAGCCGGAUAAGUGUCCC

>random_seq_from_cds__NO_954

CGUAGAGUCAACCGGGGAAGUGGUGGAAUACAUGGUCCAGCAUUUCAAGCCUCAGAUCUUUGGUGAU

>random_seq_from_cds__NO_980

CACACGCUCUGUCUCUAUCCCAGCACCUGCCUACUAUGCCCGCCUGGUGGCUUUCCGGGCACGAUACCACCUGGUGGACAAGGAGCAUGACAGGUGGAGAGGGGAGCCAC

>random_seq_from_cds__NO_1004

AUGGUGGUGGACUCCAUGAGCAGCGCGUAGUCGGCCGUCAGGGCCCUCUGGAUGCCCUCCUCGUUGUUCUUCACCAGCGC

>random_seq_from_cds__NO_1020

GUUGUCCACAUUGAGAAUCCGGAAUCCUGUCAGGUUCACGCCUGAGUAGCGGUAGGGCUCCAGGUCUAAAGCGUAGAGAUC

>random_seq_from_cds__NO_1023

GGAGCUGACGGAUCUUCAGGCGGAUGUUGUAUCUUGAUGGGGCCAUGAUGAGCUCCUGCAGUCGGAUGAGC

>random_seq_from_cds__NO_1025

UAGAGGUUCACGUAGAAGGUGUCCUUGUUGUCCAGCGGGUGGUGCUUCCAACGCAGCUGGAUGUGGGGCACCUCCAGGGCAUUGCAGAUG

>random_seq_from_cds__NO_1031

AAGCUAAAUAUGCAGCAAUUGCUAUCCAAAACCUUGGAUCUUUCAGCAGUGCUUUAUCAAAGCAGCUAAACA

>random_seq_from_cds__NO_1057

CGAGACUUGAAUCUGCCCACAAAUCACUUUGCGGUCAUUGAGGAGCAUGCCAUUCAUCUUCUCGAUGGCCUUGUCGGCAGCCUCUUGGGUCUCGAA

>random_seq_from_cds__NO_1062

CAGGUCGCCCACGUACAGGGAGGCCAUGGGGUAGCUGCUGGCCGCAGCGUUCAUGAUGGCCACCACCAAGCGCGUCUUGUACGUGG

>random_seq_from_cds__NO_1064

CAUCACAGAUAUUCAGAUUCCUCUGGAUUAUGAAACAGGAAAAGCACCGAGGAUUUGCUUUUGUUGAAUUUGAGUUGGCAGAGGGAUGCUGC

>random_seq_from_cds__NO_1106

GCUUGGUGGAACCGUGGGGGACAUAGAAAGCAUGCCCUUUAUUGAGGCCUUCCGUCAGUUCCAAUUCAAGGUCAAAAGAGAGAACUUU

>random_seq_from_cds__NO_1112

UGUCAUUAAGGCUCUGGAGCAUUCUGCACUGGCCAUCAACCACAAAUUGGAAAUCAAGGUACAUAGAUUCUGCGGACUUGGAGCCCAUC

>random_seq_from_cds__NO_1114

UCCAAGCAAUUGCCUGGGCUCGGAAUCAGAAAAAGCCUUUUUUGGGGCGUGUGCUUAGGGAUGCAGUUGGCAGUGGUU

>random_seq_from_cds__NO_1134

ACAGGAGGGGGCACCAGUGAUCGACACAUUCGCAUCUGGAAUGUGUGCUCUGGGGCCUGUCUGAGUGCCGUGGAUGC

>random_seq_from_cds__NO_1143

GUCUGCGUGGGCAUCGUGGGCAGUGGGGCUGCCCUGGCCGAUGCUCAGAACCCCAGCCUCUUUGUAAAGAUUCUCAUCGUGGAGA

>random_seq_from_cds__NO_1172

GGAUCAUCGAUGUUGUCUACAAUGCAUCUAAUAACGAGCUGGUUCGUACCAAGACCCUGGUGAAGAAUUGCAUCGUGCUCAUCG

>random_seq_from_cds__NO_1181

CGGGGGCAAAUGUGUGCUCAAGGGCAUGGGCGGCCCGCAGGUUCCGGCUGCCCUGGGUGGUCAGGAAGCCCCAGAGCCACGUGGACUGUGAA

>random_seq_from_cds__NO_1196

UCAGAGCCCAGUGGGUCAGAAGGUGGGGGCACCAGGUGGGCUUGGGGAGGCAGGAUGGUGACCACAUGCUGGCUGCUGGCUUCACAGUGGGUAA

>random_seq_from_cds__NO_1208

GGAGCCCUGCCUGCUGUGAUGGUUGGGGGCACUAGGUGGGCAGUGGAGGUCAUCAGGGUGCAGACAGGGCCGCCCCA

>random_seq_from_cds__NO_1212

UCCCAUAGAGUGAUACUUGGACUUUACUGGCAGUGAGGGCUGCCUGGAGGUGGAGGCCAAGUGCUUCGGGUGUGAGGAUGU

>random_seq_from_cds__NO_1217

GCGGCGAUCGAGUCAUCAUGGAAGCGAAUGGGUUGGGGACCUCAGGGUUUUCCGGAGCUGAAGAAUGACACAUUCCUGCGAGCAG

>random_seq_from_cds__NO_1223

GUUGAGGGUGGUGGCUCAAGCACCAUGGCUCAGGCCAAGCGCUGGCUCUAUCAGAGACCUCAGGCUAGUCACCAGCUGCUUCGC

>random_seq_from_cds__NO_1243

CCGAUGAAAAAUGCCUUUGAAGUGAAGGUAGCUGAGGCCCACUGCUAUGUCAUAGGCCAGUUUUACC

>random_seq_from_cds__NO_1248

UGGAGGAGGAGCUUGGCUCCCAGCCAGCUGGCCAAGAGAAAACCUGAAGGCAGGUCCUGUGAUGAUGAAGACUGGCAACCUGGCCUAGUGGACUCCUAGGAA

>random_seq_from_cds__NO_1251

AGGGUCCUCUGGGCUCUCGAGCAUUGGGCCUGAAAAGGGCUGGGGUCCGCCGGGCCCUCCAUGACCCCCUGGAAAAAGAUGCCUU

>random_seq_from_cds__NO_1257

GUCUUCUCCCAUCCUCAUCAUUUCCUAUGAGACCUUCCGCCUUCAUGUUGGAGUCCUCCAGAAAGGAAGUGUUGGUCUGGU

>random_seq_from_cds__NO_1265

UCUAAGGCCCUGGAGCCCCAGCUGUCAGGGUAAGAUGCUGGUCCUGGAUUAUAUUCUGGCGGUGACCCGAAGCCGUAGCAGUGA

>random_seq_from_cds__NO_1272

CCGUCAGAUCCGGCCACCCCCUGAUGGUUCUGACUGCACUUCAGACCUGGCAGGGUGGAACCACUGCACUGA

>random_seq_from_cds__NO_1273

UAAGUGGGGGCUCCGGGAUGAGGUACUCCAGGCUGCCUGGGAUGCUGCCUCCACUGCCAUCACCUUCGUCUUCCACC

>random_seq_from_cds__NO_1275

CCUGGCCUGCUGCUUCGUGGCGGCGGCCGUGGCCCUGCGCUGGUCCGGGCGCCGGACGGCGCGGGGCGCGGUGGUCCGGGCGCGACAGAGGCAGCGAG

>random_seq_from_cds__NO_1276

CGGGCCUGGAGAACAUGGACAGGGCGGCGCAGCGCUUCCGGCUCCAGGAACCCAGACCUGGACUCAGAGGCGCUGCUAGCCCUGCC

>random_seq_from_cds__NO_1280

UGAAGGGGUGCCGGCGGAGUGCGACAGCGUAGUGGUGCAUGUGCUGAAGCUGCAGGGUGCCGUGCCCUUCGUGCACACCAAU

>random_seq_from_cds__NO_1284

AGAGCCUGGCACUGUGCCUGCGAGCCCUGCUGUGUGAGGACAUGUUCCGCUUGGACCCCACUGUGCCUCCC

>random_seq_from_cds__NO_1286

CUAUACCAUGCCCUCCCCGGCCAUGAGGCGGGCCGUGCUGGAGACCAAACAGAGCCUUGAGGCUGCGGGGCACACGGCUGGUUCCCU

>random_seq_from_cds__NO_1306

CAGUGGUAUCUCCUGGUUUCUCUACUGCAUGGCCCUGUACCCUGAGCACCAGCAUCGUUGUAGAGAGGAGGUCCGCGAGAUCCUAG

>random_seq_from_cds__NO_1310

ACUGAGAAUGCAUCCAAACGCCAUCCCUUUGCCUUUAUGCCCUUCUCUGCUGGGCCCAGGGAACUGCAUUGGGCAGCAGUUUG

>random_seq_from_cds__NO_1319

CACCUCAGCACGGAGGUCCUUGUCUGACAAGAUGCUCCCAUUCUCCAUCUUUGGCCAAGAGGAGGAUAUCCAGAAAAUCCAA

>random_seq_from_cds__NO_1322

GGUCACUAAUGGCCUGUAUGUAGGACUGAGAAUUCCCUGUCCACCUGGAUGCUGCCCUGAUGGCUGAA

>random_seq_from_cds__NO_1324

AGGAGCUCUUCCCAUUUGUCCCAGCAUCACUCGUACAGAGUCUGCCAUGAGCCCCACAUAGGGCUUCAGGAUGUCAUAGUGGAAGGCUGGGGUCAGCAUCCGUCGA

>random_seq_from_cds__NO_1335

AUUCCAUUUUAACAUCCUGAAAGCAUACAUUGAGGUGAUGGCUCAUUCUGUGAAAAUGAUGCUGGGAUAAGUGGGAGAAGAUUUGCAGCACUCAGG

>random_seq_from_cds__NO_1336

ACACAAGCGUGGAGGUCUAUGAGCACAUCAACUCGAUGUCUCUGGAUAUAAUCAUGAAAUGCGCUUU

>random_seq_from_cds__NO_1345

UUGAGGUUCUCUCAGGAGAAUUCUGAUCAGAGACACCCCUAUGCCUACUUACCAUUCUCAGCUGGAUCAAGGGAACUGCAUUG

>random_seq_from_cds__NO_1346

GGCAGGAGUUUGCCAUGAUUGAGUUAAAGGUAACCAUUGCCUUGAUUCUGCUCCACUUCAGAGUGACUCCAGACCCCACCAG

>random_seq_from_cds__NO_1349

CCUACCAGAUGUACCUUGGAAGCAGGAGCCAUGACUUUUCAGCUACAUUAUUGAGCAGGGGGUGUGUGAUUUGGUUUUAUGUGAAGCUGCCUUCCCUAAGACGUUGGCUU

>random_seq_from_cds__NO_1352

AGAGGAUCAUGGUGGCCAAUAUCGAAGAAGUGUUACAACGAGGAGAAGCACUCUCAGGCAUUGGAUUCAAAGGCUAACAAUU

>random_seq_from_cds__NO_1354

UGUUAAUAGUGUAUGUCCGAUUCUGGUGGCUGUGAGAUGGUGCCCCGCCUGCUGCUGCGCGCCUGGCCCCGGGGCCCCGCGGUUGGUC

>random_seq_from_cds__NO_1372

GCCCUCCAGGCACAGUGCUGGUGAGCUUCAGCAGAUGAUGGUUGAGUGCUCCAAGUACCAUGGCCAGC

>random_seq_from_cds__NO_1373

UGACCAAAGAAGCAGCAAUGGGGCCAGGGCUUUGACCGACACUUGUUUGCUCUGCGGCAUCUGGCAGCAGCCAAAGGGAUCAUCUUGCCUGAGCUCUAC

>random_seq_from_cds__NO_1378

CUGGACAAGAUAAUAAAAUACUCGUAAGCCUUCUGGAUCCCUUGGAUUGAUUGACAUCAAUAAGGGAACCAAUUUUUGAUGUUGUAAAAGAAAUGUGUUCAUCUCCAAUG

>random_seq_from_cds__NO_1406

CAAGUUCAUCAAUGACUAUGGCACCCAUUACAUCACAUCUGGAUCCAUGGGUGGCAUUUAUGAAUAUAUCCUGGU

>random_seq_from_cds__NO_1417

CAAUGGGGGUAUCUCUUCCGAUAGGAGACCUCACAGGCUAGGCCUUGGGAUCCAACAGGACAGAUGCAGUCACAG

>random_seq_from_cds__NO_1420

GAUGAUGGCUGGGUUGUACUGCACAGCGUCUCCCCACUCCUGCAUCAGGUCCGCCGUCGGCAGCUCCUGGUAUGCCAGGGUGGUGAUGUGCUCACUUGCCCC

>random_seq_from_cds__NO_1423

CAUGGCCUCUUUGUUCAUAACGAGGGUGUAUUCAUAAAUGCCCCCAAGCACAGCCUCUGUGAUGUAGUGGGUCCCAAAAUC

>random_seq_from_cds__NO_1470

GAGAUGUCAAUGCUGAAUGCAUAUAAACCCAAGGAGCAAGGCUAAAGAGCUACCUCUUUCUGCUGUACGCUUUAUGGAAGAAUUGGGUGAGUGUGCCUUUGGAAAAAUCU

>random_seq_from_cds__NO_1473

GUGCAUGCUUUUUGAGUAUAUUAAUCAGGGGGAUCUCCAUGAGUUCCUCAUCAUGAGAUCCCCACACUCUGAUGUUGGCUGCAGC

>random_seq_from_cds__NO_1495

ACUCUCAGGCUUCAGAGAUUUAACAGCCACCUGCUCCCCUGUAUUGUCCCCUUCGGGGUCAUACCUGCAGAGCUCAACCUUCCCAAAGUGGCCCCUCUCCCAAGUCACGG

>random_seq_from_cds__NO_1507

GGUUGGAGAUUUCCUCGGGGCUUGGGCUGGCAGCAGCGUUUUAGCAUGAAGCUGAUGUUAUCCGUGCGCAGGAUCUGCUUCUUGAG

>random_seq_from_cds__NO_1522

AUUUUUCUGCUUCUUUGGAGAAUGACGCCACACUGACUGCUCAUUGUCGUUGGUUCCAUGCCAAUUGGUGAAAUAGAACCCUCAUC

>random_seq_from_cds__NO_1530

UUCUGGAACCACAUGCUUCUAUGUACUGCACUGCUGAAUGUCCCAAACAUUUUCAAGAGACACUGAUAUGUGGAAAAGACAUUUCUUCUG

>random_seq_from_cds__NO_1558

GGCCAAAGGAAGACAUGAUUUGCUGGAUCUGAAACCAUUUACAGAAUAUGAAUUUCAGAUUUCCUCUAAGCUACAUCUUUAUAAGGGAA

>random_seq_from_cds__NO_1563

CGUAUUAACAUAAUGAACCUGUGUGAGGCAGGGGUUGCUGGCUCCUCGCCAGGUCUCUGCAAACUCAGAGGGCA

>random_seq_from_cds__NO_1571

CAUGGUGGGCAUUUUCUCAACGCAUUACUUCCAGCAAAAGGGUGUUUGUUCUCCUAGCAGCCCUCAGACCUCAGUGGUGUAGCAGAGAAAUUCCAGAUCC

>random_seq_from_cds__NO_1579

GAUAAGGUGGGGGAUGCCCUGGAGGAAGUGCUCAGCAAAGCCCUGAGUCAGCGCACGAUCACUGUCGGGGUGUACGAAGCGG

>random_seq_from_cds__NO_1582

UGCGCGUCAGCAACCCGGGCCGGCUGGCGGAGCUCCUGCUCUUGGAGACCGACGCUGGCCCCGCGGCGAGC

>random_seq_from_cds__NO_1585

CCUGAACGGUGACAUGAGCAACACUACCGUCGUCCCCAGCACUGCAGGUCCGGGCCCCAGCGGCGGGCCCGGUGGCGGAGGUGGUGGUGGCGGCGGAGGCGGCGGCACCG

>random_seq_from_cds__NO_1587

UUCGCCUUUGCCAGUCUCAUCUCGUGUCUGCUUUGUUAAGUUCCAUGAUCCAGACUCAGCAGUUGUGGCACAG

>random_seq_from_cds__NO_1633

UUUUCUACUAUAAGUACCAGAGCGAAUGUAUGUCUCCACGGGGUUGACACCACAUGCAUGGACCUUGAUUAGAACCCUGAU

>random_seq_from_cds__NO_1636

UGCCCAUAAUCCCAGAAUAAAGCCCCCAGGAGGAUGCCAGUCUUUCAGCUUCGGUUUCCUGGUGUAUUAUCUGAGCUACAGGAAGUU

>random_seq_from_cds__NO_1646

AACACUGUAGCUGGUUUGGGAUUUCUAGGAUUACAGGCCAUAUCAUCUGCAAGCAUUAGGACAAUGUGACCUGUCAGGAAUACCUAGCCUCUUGACACUUCUAUAAACAG

>random_seq_from_cds__NO_1655

GAGGCCCAGGAGGUCCAUGGGGGCCUGGGACACCAUGGGGCCCAUGGGGUACAGGUGGCCCUAAAGGAUUUACUGGGCCCACCAAUC

>random_seq_from_cds__NO_1675

CACCAGGCCCGGGGUCCAGCAUACCACAAACGCCCCCUAAGACAGUCAUCACCGUCUUCAUUAGCUUCAUGGGUGUCCUCCGGCGGCUG

>random_seq_from_cds__NO_1680

UUUUUGAAACUGGGCCUGUGUUAAACAUCAGGAAUACAUAGGCAAUUCCAGCGAAGAAAUCGGCAGCA

>random_seq_from_cds__NO_1683

CAGUGUUGCUCCUAUUAUAAAAAAAGUCCAUGUGCUUGUCAUAGUGACACUCAUUCAUGUCAUUGUCGUGAAACAGUACGUGAUCUUAAGGGAAGAAACAUCUCACUAG

>random_seq_from_cds__NO_1709

CAUCAAUUGAAAAACACAGUGACUGUGGAUAAUACUGUGGGCAACGACACUAUGUUUCUAGUUACGUGGC

>random_seq_from_cds__NO_1727

GAAGACAAAGGCUGACUAUGUGAGACCAAAACUUGAGACCUACAAAAAUGGCUGAUGUUCUGGUUGCUGAGUCUACUCCUCC

>random_seq_from_cds__NO_1738

CGGAUGGGGAAGACAACACUAUAAGUGGGUGCUUUAACGAGGUCAAACAAAGUGGUGCCAUCAUCCACACAGUCGCUUUGGG

>random_seq_from_cds__NO_1774

GGAAAAUUUCACAGUGGAUGCAACUUCCAAAAUGGCCUAUCUCAGUAUUCCAGGAACUGCAAAGGGUGGGCACUUGGGCAUACAAUCUUCAAGC

>random_seq_from_cds__NO_1787

AUUGAAAAGCUCACUGGUAACUUUGAAUAACAAUGGAUAUGAUGGCAUUGUCAUUGCAAUUAAUCCCAGUGUACCAGAAGAUGAAAA

>random_seq_from_cds__NO_1833

UCCUGGAGGGUUUCUGUGGGCAGCUGCACCUUCUGGCCCAUCUGCUGUUCAUAGUGGGCAAUAGCCUUUUG

>random_seq_from_cds__NO_1844

UCUGGAUUCGCCAUCAGUCGCCCAUUAGUGUUCUCAAUGAGGCACAUUGGGCCUGUCAUGUGGAUCUCUGAUGCCAUGA

>random_seq_from_cds__NO_1846

UGAUACAAGAUAAAAAUAAAUACAACACACCCAAAUACAGGAUGAUAGUUCGUGUGACAAACAGAGAUAUCAUUUGUCAGGAUUGCUUAUGCCCGUAUAGAG

>random_seq_from_cds__NO_1849

CAAGUGGAGGUGACUGGUGAUGAAUACAAUGUGGAAAGCAUUGAUGGUCAGCCAGGUGCCUU

>random_seq_from_cds__NO_1855

GAAGAAGGAUCGGGUAGCUCAAAAGAAGGCAAGCUUCCUCAGAGCUCAGGAGCGGGCUGCUGAGAGCUAAAUCAAAGCUCUGCCUGCUUUACAGGAGG

>random_seq_from_cds__NO_1864

CCACUGCAAUGCCGAUGGCCAUUGUGUUGUGUCCUUUCAAUUAUGUCCAGGCGCAGCUGGUGUCCGUG

>random_seq_from_cds__NO_1869

GUAGUUGACGCCAGAGUUCCGGGGACCUGGCGCCUCAUUCUGCUUGGCUGUGAGCAGUUCUGUGUGGCUUCCCUGGGCACA

>random_seq_from_cds__NO_1877

GGUUGCCCACGUAGCAGCGCACCAGGCCGGGGAUGGAGUCGAAGCUCUCCAUCUCGAACUGGUACUGCACGCGGCUGUAGGCCUCGCUGAGU

>random_seq_from_cds__NO_1887

AGUCAAAUCUGGUGGCAUCACACAGCAGGAGGCAAGAUUAACUUGGUUACUAUUUGGUUUUACCCUGUGCCCACUGAUACAGCUGUUCCAACUGUGUUUUGUCUAGAUCA

>random_seq_from_cds__NO_1897

ACAGGGUUCAGGUCAGGAAGCAGGUCGUCCUUCGGGGAUUUGAUCUUCAUUGUGACGAUAUAGCCAUCUCCAAAUCUUGGACUUGAGAU

>random_seq_from_cds__NO_1911

AUCCCAUACAGCAGGAGCAGUGCCACAAGGGCAGGAAGGUUUUCUGGAGAAGUGUAGGCUUUCUUCUGAAAC

>random_seq_from_cds__NO_1914

GAAGGACAUGGAGAAAAUCACGCAGAUGGCAACCACAGCAUCCACUGAAGUGGUCAGCCACUGUAAUCUCUGAGAGCUGC

>random_seq_from_cds__NO_1930

CUUAUUUGGAAGAAGGAAGAUAAGUUCUUGACCAAUGCACUCCACCAGCUUUGCCUCUGGAACAUGGUGGAGAACUACAUCCAUCAGCUCAUUUACAUCCCCCAUCCAGG

>random_seq_from_cds__NO_1940

AGUCCGUGUAUUCCUUCUGGGUGCUCUGGAUCCUCCGUUUCCUCUGUUAGGGGCUCGGUCUUUUCCAGGGCUCUUUCUUCUCUGGUUGAACACCCCUU

>random_seq_from_cds__NO_1946

ACAGGAAGAGGAUGAAUGGGUCGCUGUAAUGUAGGAUUCUUCCAUGCCAUGAUGAAUAUCGUCAGGAGGAAGAUGCUCAUCGACAUGAUGGA

>random_seq_from_cds__NO_1953

CAUGUCAGGGAAUACCACUCCGGCCCAGAACAUGUUUUCCUCCAGUAGAGAGAGGGCACGUUGGGUGAGCUGAGUUUCAUCAUU

>random_seq_from_cds__NO_1959

CUGGAUCAAUGCAUUACAAAAGGAUGCUUGUUCUUCUGUCAUAAGAAUAGAUAGGAUCCUUCCUUGUGGAGUCAAUCCCCA

>random_seq_from_cds__NO_1970

UGUUGGGGAAAUGGCCAUUCAUGAUGGCUGUAGAGUGGGUUGGCAUUCCUUAACCAGAUCAAGACCAGAAAU

>random_seq_from_cds__NO_1998

UCUUCCGGUUAACUGUUCGGGAGGGAAUCACUGCUUGAACACUGAAACAGUAGUUUUCUCCUUUAUCCAC

>random_seq_from_cds__NO_2005

ACCUGGGCGAAGACCCAGCCGAGCAGGAGCGUCCGAGCGACGGCGGUCUCGGGGCGCGGGACC

>random_seq_from_cds__NO_2007

CUGACGAACUACUCUCAGGCAGUGUUCUCAGUAGUCCGAACUCUAAUAUGAGCAGCAUGGUAGUUACAGGCCAAUGGUAAUGAU

>random_seq_from_cds__NO_2020

AGACAGCGCUCUAAUACAGAUGGCUGAUGGAAACCAAUCACAACUUGGCCAUGAAUCAUCUUAAUGGACAGAAAAUGUAUGGAAAAAUUA

>random_seq_from_cds__NO_2031

UCCAUGAGUUCAGCUAUACGUGGAACUGGUUUCCCUUUCUGGUGACUCACAGUAGCUGGACUCUGUCCAUCCCAG

>random_seq_from_cds__NO_2038

UUGUCCAGGGCCAUACAUGGGGCCAGAGGUGGUUCCCCGGAUGAUUCUGGGGGAAACAUUUGUCACAAUGUCCCUUAUCAA

>random_seq_from_cds__NO_2053

UGGGGGAGGCAGCGAAGGAUUUCUGAUCUGUGGGAUACUCAUUGCUUUGAAUACCCUCAGUAGCAAAUUGCUGCAAUCCACCAAUAUU

>random_seq_from_cds__NO_2061

AUGAGUCUGUCCCAGAGAAUGUGCUGAGUUUCGAUGACCUUACUGCAGACGCUUUAGCUGAACCUGAAAGUCUCACAAAUCAAAA

>random_seq_from_cds__NO_2065

UGGUGUUCCUGGAACAGGCUCUCAUCCAGUAUGCCCUUCGCACCUUGGGAAGUCGGGGCUACAUUCCCAUUUAUACCCCC

>random_seq_from_cds__NO_2071

AAUAUUGUCUCAGGGUUCUUUGAAUCAUGCUGCCAGUAAGAAGCUUGACCUGGAGGCCUGGUUUCCGGGCUCA

>random_seq_from_cds__NO_2072

GGAGCCUUCCGUGAGUUGGUCUCCUGUUCUAAUUGCACGGAUUACCAGGCUCGCCGGCUUCGA

>random_seq_from_cds__NO_2082

CCUGCAAGCUGGCACAGGCCCCCGGGCUCAGGGCAGGGGAAAGGUCACCAGAAGAGUCCCUGGGUGGGCGUCGGAAAAGGAAUGU

>random_seq_from_cds__NO_2096

CAGGCUGGAUCUCUGUGGCUGCUGAACUGGACCGGGAGGAAGUUGAUUUCUACAGCUUUGGGGUAGAAGCUCGAGACCAUGGCACUCCAGCACU

>random_seq_from_cds__NO_2098

GCGGCUCAAUGAGGAUGCAGCUGUGGGCACCAGCGUGGUGACGGUGUCAGCUGUGGACCGUGAUGCUCAUAGUGUCAUCACCUACCAG

>random_seq_from_cds__NO_2103

AUCGAUGCAGACACGGGGGCUGUCACCACCCAGGCUGAGCUGGACUAUGAAGACCAAGUGUCUUACACCCUGGCCAUUACUGCUCGGGACAAUGGCAUUCCCCAGAAGUC

>random_seq_from_cds__NO_2110

AACAUCCCUGAGGUCUUUCAGCUGGACAUCUUCUCCGGGGAGCUGACAGCCCUGGUAGACUUAG

>random_seq_from_cds__NO_2119

CUGCCUGCGGGAGCCCUGCGAGAACUACAUGCGCUGCGUGUCGGUGCUGCGCUUCGACUCCUCCGCGCCCUUCAUCGCCUCCUCCU

>random_seq_from_cds__NO_2120

CCGUGCUCUUCCGGCCCAUCCACCCCGUCGGAGGGCUGCGCUGCCGCUGCCCGCCCGGCUUCACGGGUGACUACUGCGAGACCGAGG

>random_seq_from_cds__NO_2127

ACUGUUGGGUCAGACAGGGCUCCCACAGGGCCCAUCAGAGCAGAAGGUGGCUGUGGUGACCGUGGAUGGCUGUGACACAGGAGUG

>random_seq_from_cds__NO_2128

GCCUUGCGCUUCGGAUCUGUCCUGGGCAACUACUCCUGUGCUGCCCAGGGCACCCAGGGUGGCAGCAAGAA

>random_seq_from_cds__NO_2160

UGCCCUGGGCCUGGCUCAGCUGGUCUUCCUCCUGGGAAUCAACCAGGCUGACCUCCCUGUUUGCCUGCACAGUCAUUGCCAUCCUGCUGC

>random_seq_from_cds__NO_2166

UCCUAUGUGGUGCUUAGCAAGGAGGUCCGGAAAGCACUCAAGCUUGCCUGCAGCCGCAAGCCCAGCCCUGACC

>random_seq_from_cds__NO_2173

CCCCUGAGGAGCGGCUGCGGGAGAAUGGAGAUGCCCUGUCUCGAGAGGGGUCCCUAGGCCCCCUUCCAGGCUC

>random_seq_from_cds__NO_2186

GCUAGGCCAGUGAAAUAGAUGGGGUCCCUGGUGAACGUGUAGGUUUGCCAGCAUUGACCUUCGUCUGU

>random_seq_from_cds__NO_2198

GAGGGCUAUACAUCAUCUGAGUGAGAGGAUGAAAAGGGAGAUCUGUUUGCACAAAAUUCUUCGCAAAAUCUGAUGAU

>random_seq_from_cds__NO_2200

GCCAAAUUCAGUCCGAAUAAAGGUGUUAUUGAUGAGAUCUGUAAUAUCCUUAAAGUUCUUCCCAUAAUCCUCACCUUCGAUAUAGCUUGGACUGUCCAAAAG

>random_seq_from_cds__NO_2211

AAUUAGCCCACUCAUGGCACAACCCUAUGUGAGCAUCAAUCUCUACAAUUUUCUCAAUGCUGCUGGGCUCCAUCAGUGG

>random_seq_from_cds__NO_2218

GACUCAUGCAUACGAUUCCACUUCGUCAUCUUCCACCAGCACCAUAUCAUAGGCACUGAGGGCUGCACAGAAAAUGAUGCAGGUGACUCCCUCGAAG

>random_seq_from_cds__NO_2221

UAAUUCGUUCUAAUUGGUUCAGGUAGCUAAGAUGCGGAGUCAUUAAGCUGGUAUUCUGCAGCUCUCUCGAAGCAGGCUUGCACCCCACCAUCCUUCCACAAC

>random_seq_from_cds__NO_2234

UCACAAAGAUGGCUGUCUGGGGCAACAAGUAGCAUGCCCAUGACUCUGGGGUACUGGGACAUCCGUGGGGCUGGCCCACGCCAUC

>random_seq_from_cds__NO_2239

UGGGGAAGCGGCCAUGGUUUGCAGGAGACAAGGAUCACCUUUGUGGAUUUCCUUGCCUAUGAUGUCCU

>random_seq_from_cds__NO_2247

UCAUUUGCUGCAGUGUUGUGGUAUAAAUGGCACGAGUGAUUGGACCAGUGGCCCACCAGCAUCUUGCCCCUCAGAUCGAAAAGUGGAG

>random_seq_from_cds__NO_2263

UGAAGAACGGCCAAGGUUUUGCACUAGUAUAUUCUAUUACAGCUCAGUCCACGUUUAACGACUUACAGGACCUGAGGGAACAGAUUUUACGGGUUAAGGACACGGAAGAU

>random_seq_from_cds__NO_2276

UGGAACGGAACACAUCACUAGAUCCUGGGAACCUGGAUCAUCCAUGUCAUGUUGGGGCAGUGGAUGCGAUUGCAGACGAACCAGGAGGAGAGUUUGCUCCACUCAUCAGG

>random_seq_from_cds__NO_2277

ACUGCGGCCAUAGAUGGACAGGCGGGGCUCAGCAUGCUGGUACUUGGCCUCCACCAGGUCCGCACCUACCUCCCUUGAUGAUAGUGGCAA

>random_seq_from_cds__NO_2285

AGGAAGGUUGUCUGUUCGGAAGGGGUCCUCUCCCUUCUUCACAGGAGGAGUAAAGACCUGGAUAGAAGCUCUCAUUU

>random_seq_from_cds__NO_2288

UGGAGAUGAGGAAAUGUAUUCAUCAAUGUGGGACAGUUUGGUGGAAGAUGUUUCACUUAGUGGAAUGGACAAAUUAACAGUCUUC

>random_seq_from_cds__NO_2294

UAACUCUUGGCCAGUUCGUGGGCUUGUUUUGUAUCAACUGUCCUUGUUGGCAAAUCACACUUGUUUCCCACUAGCACCAUAGGUACAUCAUCCGA

>random_seq_from_cds__NO_2334

UUGCACAUGAUGACAGGUGUGAUGUACCUACAGUUUGUACCCAAGUCUCACCUCUUCUUCACUGCCGGAAAAGAU

>random_seq_from_cds__NO_2339

AAAAUGAAGGAACACAAAGCCAUUUGUAAAGCUGCAGGGAAAGAGGGUUCCACUUCCCAGCAACCCCAUCCUAAUGGCUUAUGGC

>random_seq_from_cds__NO_2360

ACUUGCCCACAGAGGCUGGGAAAGGAUGAUAGGCUGGGAGAAUGGUCUGAGCUACCUGCCCGUCCUGCUGGGGCAUCAUGGCAGUGG

>random_seq_from_cds__NO_2366

ACAUAGGGUUGGGUGAGGCCUGUAAGAUCCCAGGGGAUGUAAUCAUUGGAGAGGAUGUGGUGUCGGAAACAUACGUGUGAGGAGAUU

>random_seq_from_cds__NO_2385

UGUUCUUGUUCACCAGGAAGGGAUCUGCGUGUCAUCCUCUGUUUCUUCAUAGCAGCUGACUUCUCACCAUAAUAGGGGUACACCAUGAGUUCCCCCUGGGAGUCCCGCUU

>random_seq_from_cds__NO_2392

CUGGCAGGGGCUACUGGCACAGCCUGACUCGCAGUCCCGGGGACUGGGGCAGAAGCAGCGGGGUCCAGAGGCGGUGUGCAC

>random_seq_from_cds__NO_2396

AGGGGUUGGAGAGGCACUCGUUGAUGUCUCCCUCACAACGCUCCCCAGCAAAGCCAGGCAAGCAGCGACAACUGUAGCCUCCAAUCCUAUCCAUGCAC

>random_seq_from_cds__NO_2403

UUCACCAGGGUCCUGACAGUUUUUCCCAGUGUAGCCCAGGGGGCAGCUGCAGCGGUAGGUACCCAGGCCAUCAACACACGUUCC

>random_seq_from_cds__NO_2422

AUGCACACCUUUGAAACCCUGGCAUGCACAGACAUGUGAAGCCUCCAAUCUUAUCCAGACAGGUAGCAUCAUUCUGGCAGGGGUCUGAAUGGCA

>random_seq_from_cds__NO_2423

CUCAUUGAUGUCCAUCUCACAACGAGGUCCUGCAUAACCCUUCAGACACUCACAGUGGAAGGCGCCAUCCGUGUUCACACA

>random_seq_from_cds__NO_2424

UUUUCCUGCAUGCUCACAAGGAUUGCUAUUGGCCCAUGGCACAUUCAUCCACAUCUUCUGUGCAGUCAGCCCCUUUGUAGCCUUGUGGGCAGGUGCA

>random_seq_from_cds__NO_2443

AGGCUGCUAUGGAGGGACUCAAUGGCCAGGAUUUGAUGGGACAGCCCAUCAGCGUUGACUGGUGUUUUGUUCGGGGUCCACCAAAAGGCAAGAGGAGGAGG

>random_seq_from_cds__NO_2453

CCUUCACAUUUCCUUUCACUUUCACCAAGCCAGAAAUGAUACGAUUUGGCAGGUCAUCAUUUAAGGUUGGAUGCUGACUCAGAGCUC

>random_seq_from_cds__NO_2465

CCAGCCAAUCCCAGCCAUCCCCCUGUGACUCCAAGAGUGGGGGCCAUACCCCUAAAGCACUCCCUGGCCCAGGUGGGAGCAUGGGGCUGAAGAAUG

>random_seq_from_cds__NO_2489

UACGCCCAGGUGGCUCAGACAUGCUGCCUGCUCAGCAGAAGAUGGUGCCACUGCCAUUUGGUGAGCACCCCCAGCAGGAGUAUGGCAUGGGCCCCAG

>random_seq_from_cds__NO_2493

AACCUCAAGUCCCCCCAGACUCCAUCGCAGCUGGCAGGCAUGCUGGCGGGCCCAGCUGCUGCUGCUUCCAUUAAGUCCCCCCCUGU

>random_seq_from_cds__NO_2518

GGAGGUGGCACUGGGGGUGGGAAGGAGCCAGGAGGAGGCAUGCCCUGGUGGAGGAAGCCCAGACCCCAAUGAUGAUACCACAGG

>random_seq_from_cds__NO_2547

GGUCAACACUGGACGUAUGAGGGGCCCACAUGGUCAGGACCAUUGGCCAGCCUCUUACCCUGAGUGUGGA

>random_seq_from_cds__NO_2554

AGUGUGCUCUGGACAGUUUUUUAUAGAAGGUCCCAGAUUUCAAUGGAACAGGCUGGAAAAGCUUCAGGGGACAUUGUUCUCCACA

>random_seq_from_cds__NO_2556

GGCAGGAUCCUCGUAUACCACAGGGUGAAAUGCUGAGUCUAGGUGUAGGAAUCUUGGUUGGCUGUCU

>random_seq_from_cds__NO_2562

UCCCAUGGCUUUGUAGGGAUAGGAAGCGUCUGAGUCGAUGCCCUUGUUAUCAAUGAUGUACUGGAAAGCCG

>random_seq_from_cds__NO_2592

GUGUUGCUUUUGGGUCUUUAUGCUCCUGGGAUAAGGAACUUUGAAGCACAUGCUCCUUUAAGUCUUUAUUACCCUGUGGUGCAGUGUCUACCUCUCCC

>random_seq_from_cds__NO_2625

GGAUGUCCUUGGGCUCAAUGACCCUUGUGCAGCUUUGGGGCCCGGGGCUCAGAGCCCACGUCCACCACAUAGAUGCGAGAGGAGAUGAGACUGGGCAGCACCAGCU

>random_seq_from_cds__NO_2629

AAGCGUUUUUGGGGUCGCGGUCCGGACUUUGGGCGGGGGGUCCGGCCCCAGGACAGUUUUACCGCAUUCCGUCCACUC

>random_seq_from_cds__NO_2630

CCGAUUCCUUCAUGGAUCCGGCGUCUGCACUUUACAGAGGUCCAAUCACGCGGACCCAGGAACCCCAUGGUGACCGGG

>random_seq_from_cds__NO_2633

UUCUCGGCCAGAUGGUGGAUUGAUGAGGAGCUUCUGGGAGAUGGACACAGCUAUAGUCCUAGAGCUAUUCAUUCAUGGCUGACCAGGG

>random_seq_from_cds__NO_2636

CAGUGCUAAGCCAGACCGAGGCCCGCGACUUAGUAGAACGCUGCAUGCGAGUGCUGUACUACCGAGAUGCCCGUUCUUACAACCGGGUUUCAAAUCG

>random_seq_from_cds__NO_2655

UGAGGACACUCGGUCUCUAGCAAUUUCUUCAGGUCAUCCCUGUAGACGGCAUGGAAAUUCCCCUUUAUCAGGGAGUACUUGUGGUAGACGUCGAUGAUAG

>random_seq_from_cds__NO_2674

AGUUUGAGUAUCGGACAUGUCAUGCUGCCCAAGGACAUAGCCAAGCUGGUCCCUAAAACCCAUCUGAUGUCUGAAUCUGAAUGG

>random_seq_from_cds__NO_2675

AGGAAUCUUGGCGUUCAGCAGAGUCAGGGAUGGGUCCAUUAUAUGAUCCAUGAACCAGGAACCUCACAUCUUGCUGUUCCGGCGCCCACUACCCA

>random_seq_from_cds__NO_2676

AGAAACCAAAGAAAUGACUUAGCUCUCGGAGGCUACAGCCUCCCCGUCGCGGCUCUCCGUCUCCUCCGGGAUGUCCUGCAUUCUG

>random_seq_from_cds__NO_2686

CCUCCCUCUUGCGGUGAUCUCAGCUGAUUUCCAUUUCGAUCCCCUCUGGCUAGAGUCUGAGUCAGCUCUGCAGAGUUCAAUGGAGC

>random_seq_from_cds__NO_2689

UCCUGCAGCAGCCGCUCGCCACGAGGGGACUCAAGGGACUCAGAGCGGAAAAGGCCCCUGGGCAGGGU

>random_seq_from_cds__NO_2698

ACCAGCAGGAUGCACUCCUGUACCCCGUGCCGCUUGAGCACGGCGGGGCGGGUCACUUUCCCGGAUGAAUUGCUGGAAGCGU

>random_seq_from_cds__NO_2703

AUCUUCAGUGUCCUCACAUGGUGCAGCUCUGUCUGGAUUAGCUCCAUAGAUGACAUCUUGCUGCUUCAUCACCUCCUUUUUAUGCUG

>random_seq_from_cds__NO_2706

GCGCAGCCCCAGGGGAGACUCAUCAUUGAAAUGUCCCAGCAAUGUUGGUGGUAGAAACACUCUUGGCUAA

>random_seq_from_cds__NO_2721

UGAUUUUCCAGUUGCCAGAGAAGUUGGGCAUAAUGGUGAAUGAAUACAAGAAAAUUCUUUUGCUGAAAGGAUUUGAGCUCAUGGAUGAUUAUCAUUUUACAUCAAUUAAG

>random_seq_from_cds__NO_2726

CAAAAGGAAUAAGGUGUCCCAAGAGCAGAGUAAGCCCCCAGGUCCCUCAGGAGCCAGCACAUCUGCAGCUGUGGAUCAUCCCCCACUAC

>random_seq_from_cds__NO_2742

UUCUUGGGUCUCAAACGUGAAGGGCUUCUUUGCUUUCAGUACCAUAACUGGCAAACAGCGCUUCUGAAACCC

>random_seq_from_cds__NO_2743

UUCUCUGAUAGAUUCCUGCUGGGCCACCAUCUGUUUCUGUUCAGGCCUUAACAUGAGGAGAGACUUUUGGUGCAGCACGUUGCUUUGCG

>random_seq_from_cds__NO_2756

ACCCUUGGUGUGGGUCUGAAUCAGAGGAUCUGGCGUGGCAUCCCGUAGCCAGUCAUGCCUGCCUGAGACGCCCCGCGGUUGGUGCCCAUC

>random_seq_from_cds__NO_2757

UGUAACCCGAUCACGUUCUUGCCCUCUUGCAGCUGGUUAUCCGAGAAGUUCCGAGGAUUCUCCUUGGAUUUCCUUAGGGAACCAGUUGGGAUCCCCAGAGAAGAGCCCAU

>random_seq_from_cds__NO_2766

AGCUGAUCCUGGAGGUUAGCAGCCCAAGUCCUAGAAGACAAGGGUGUUGGCUUCGGGCUGGUAGACUCUGAGAAG

>random_seq_from_cds__NO_2795

CUUCUUCCUCCUCUUCCUCUUCCUCUGAGCGGUCAUGGACUCGAUUUUCAUCAUUAAUGGAGUAAUGACCAGUGUCCUCCAUGCUGUCAGAGUCCUUAUCUUCACCUGA

>random_seq_from_cds__NO_2796

GCUCUCUGUGUCUGUGCCUCGACUUUCUGUGCUGGUGCGGUUGGGGCCACCAUCAUCCCCAGUCAAGCUCAAACUCAGGUCUGAGG

>random_seq_from_cds__NO_2801

GAUUUUUCGGGUCCUGUUGGGCCACCUCAGGCUUGGGCCCGAGUUCUAGUAGGCGCCGAGCAAAGGUGGCAGCUGUCUUGAAGUUCU

>random_seq_from_cds__NO_2802

UGAGCUUGAAGAACAGAUUGAGGGCUGUACGCAGCACCAGGAUCAUGUGCACAGGCUGCAGGUUUGAGUGGG

>random_seq_from_cds__NO_2809

CAGAUCCUGAGUUGGACUUGUUCCCUUGGUUGGGGGCACAAAGAAACCAUCUUCAGCCCCACCAGCUGCCCCAGGGGAUAUAUCCCA

>random_seq_from_cds__NO_2813

AGCAGCUUGGCAUUAGGGUCAAUGUCUGGGAUCUGUCUCCUUCUCUGGGUCAAAUGUCUCCUUUAGGCUCUCAGCUU

>random_seq_from_cds__NO_2814

CUUCAUCUAAGCCAUGGGUAGCAGCUGUGAGAUAGGCCAGGGACUCUCUGUCCACAGUUCUUCAGGAUCCGCACACGCUCUGACACAUC

>random_seq_from_cds__NO_2822

GACAGCAUAUUUGAUGUGGUUGCUUGUGGUAUAGAUAAAUACCCCACUCUCAUCCCAGGCCCCACUCUUGACACGAAUGUU

>random_seq_from_cds__NO_2823

CUCAUGAAUGUUACAUAAAGCAUCCAGUUUGCGGUUACAGAUCACAAUGGCCGUGUUUGGCUAGUAGUGCUACAUGUGACAUGUCUG

>random_seq_from_cds__NO_2830

UAGCAUAUUGCCAUGAACAGCAUAGGCUGGCCGUUCCCGUUCCAGCUUAAACACAAUCAUACCACCAUCAUGGCCCUGCUGCAAAG

>random_seq_from_cds__NO_2834

AGGGGCAUAGUGGGGUGGAAGGCAGCCCAGUUUACUCCACGAUCGUGACCCCUCUAGUACAUGCUUCACC

>random_seq_from_cds__NO_2842

CUUCUGAGUUUUCCUUGGCUGCUGGCUUCUCAUCUUCUCCCAUGAGACGAAUGUUGUGCUUUUCCCGGAAUUCAUUUAGU

>random_seq_from_cds__NO_2845

UCCUUCAGUGUAUCGAUCACUAGGCCUGUGCUCAUUCAACUCCAUCUCCAACUCAGCUGCUUUGGAUGCCAGGCCUCGCUGUUCCUGCCGAAGGCGG

>random_seq_from_cds__NO_2854

CAUGGCGGGGUCCCCGGGGGUCACACACAUACAGAUUGUGGUCAUUUUCUGGCAAGAGGUCCACAUCGUGCAAGAACAGGC

>random_seq_from_cds__NO_2856

UGUUCCAUUUCCAGCCCUGGUGGAUGACAUAGAUGCCAUAAGCAAGCUGCUGGCGCUGCAAGAAGGGGUGCAGGUGGUAGAGCAGCAGGCGCAGGUGGUGCUCCCGGGCA

>random_seq_from_cds__NO_2862

UUGGUUGAACAAGCAGGCGGCCCUGGGAGAGCCUCAGCUCUGCUAUAUCCUGGAUGCCAUCCUGUUUCUGUAUGGAAUUGUC

>random_seq_from_cds__NO_2864

GACUUACGAGACUCUGAAGCAUGAGAAACCACCACAGUAGUUCACUGGGUGGCAGGCUGUGUUCCAAGUUCCACGAAAUAG

>random_seq_from_cds__NO_2873

GUGUUGUCCUCUAUGGGGCUGGCAGCCAUGUGACAUGUUGGAGGCUCCGGGCCCGAGUGAUGGCUGCG

>random_seq_from_cds__NO_2887

CAGCUGCCAAAUAUGGAAGGGCUGGACUCAGAAGAAGGGGAUGGAGCCUGGUGCCCUGAGAUUCCAGUGGAACCUGAUGACCUG

>random_seq_from_cds__NO_2902

GCUGACAUAGUGAACCUCCAAGGAGUGACAGGAGGCAACACAUACUCAGUGCCUGCCGUCACCAUGGACCUGCUCUCAGG

>random_seq_from_cds__NO_2903

AAAAGAUGUGGCUGUGGAGGAGUUCCCCAGGAAACUCCUAACUUUCAAAGAGAAGCUGGGAGAAGGACAGUUUGGGGAGGGU

>random_seq_from_cds__NO_2914

AUAUGGUUUUGUGCUUCUAACUGGUGCUGCCAGCUUUAUAAUGGUGGCCCACCUAGCCAUCAAUGUUUCCAAGGCCCGCAAGAAGUACAAAGUGGAGG

>random_seq_from_cds__NO_2921

CCCGUUGGAUGUCCCCUGGUAAAGACGCCAGCUGCUAGUCCAAAAGUGGUAUCAUUGGCUCUUUCUAGAACCUCAGCUUCAGUGUCAAA

>random_seq_from_cds__NO_2938

UACCGUGUCCUUACCUCGGAGCAGAAGGCCAAAGCCCUGAAGGGCCAGUUCAACUUUGACCACCCGGGAUGCCUUUGACAAU

>random_seq_from_cds__NO_2974

ACUGUGAAGUGAUGAUAGGGAAUGAAGUCCCUGCCUCGGCAGUGGUCACAUCUGUGGGCAUCUUCUUCUAAAAAGCGGGCACA

>random_seq_from_cds__NO_2975

UUUCAAGUGCAUGGCUUUUCUCUGGUCCUUGAGCCACAGCUCGUAGGCUGUUUUCUGCAUCAUAGGGUUACAGAAUCGAAU

>random_seq_from_cds__NO_2980

UUUCCUUUUAAUGACGUUGGAGGUGACAGGUUUUUCAGUCUGACACCACUUGUGAGGUGACAGACUUCUUCACUUUCCUUAUCACU

>random_seq_from_cds__NO_2981

AUGGAGAGUAACCAUGUUUAACUUCUCUGUUAGCUUAAUGGAAUACUCUGAACAGGUUAUUCCAGGUCCUAUUUGUCUUUUCCU

>random_seq_from_cds__NO_2997

GACAGAAGUGAUGUGCAUAUAGGCAUCCUGGAUGGCUGGGCCUAUCUCUUCUGCUUUGUCUUGGUCUUCAAA

>random_seq_from_cds__NO_3009

AAGGCGACCCUACGGAGAAGCAACUUCAGAUCAUCCUGGAGGAUGCACCUCUCUGGCAGAGAUUCA

>random_seq_from_cds__NO_3011

CCCCAAUGCCAUGUACUCCCUCCUGCUGGACUUUGUCCCUACGGACAGUCACCGCUGGAAGUACGUCAACG

>random_seq_from_cds__NO_3017

UGCCGCUCCUCUGCCUCUGCCUGCUCCCCACACCCACCAUGGCUGUGAGCACUAUUCGGGUCUCCGAGGACA

>random_seq_from_cds__NO_3023

CACCUGGACAGCAGUGGCCUCGCAUCCCUUCGCGGGCUGGGGUGGCCCAGGAGCGGGUGGGCACCAUUCUCCUUCCUCACU

>random_seq_from_cds__NO_3031

AUUCCUUGUGCUGUUCCCUUUGAAGAUCUGCCAGUUGAUCUGGUUGGAACUGUAAGCUACAUAGAACUCU

>random_seq_from_cds__NO_3040

CAUAGGCAUGUUGCUGUCCUUAUGUAGUAUUCCUUUUUGGCAGAUUAGGAGGGGACCUAUCAAGCCUGAGUGA

>random_seq_from_cds__NO_3048

UAACAUCAGUUUUGUAGGGGUCAUCAUAGGGCACAUAAUCAAUUUCAGCAUAGUCAUCUUCACUGCUCUGGACCUCUUCCUUUGGAAUGAUCUCAAUGUAAU

>random_seq_from_cds__NO_3058

GUCUGGCUGAGGUCUAAAGAAAGGGUUGUAUGGCUGAGGUCUGGAGAAAGGGUUGUAUGGCUGAGGUCUGGAGAAAUGGGC

>random_seq_from_cds__NO_3059

AUCUGACCGAGGGCUGGGGAAAGGUUUCUCUGAAUGAGUUCUGGAGAGAGAGUCGUGUGGCUGAGGUCUGGAGAGAGGUUUGUCUG

>random_seq_from_cds__NO_3063

UCUGAUAAAGACCUGGAGGACAGCUUGCCUGACCAGUGUCAUUUGAGGAAUUCUGAUUAUGGUCAGGAAGUGAGGCUAUCCAG

>random_seq_from_cds__NO_3077

CAUAGUCAGCAUCACUCUCUUCAUCUUCAGGUUCUAAACGAUCAUGCAUUUUCCGUGUAGCCAUGACUGUAGAUUCUGGAGGUU

>random_seq_from_cds__NO_3105

CCCCAGAGGUUGGAGCAGUUCAUCGCUAUUGGCUUAUUAACAUGUAGUUCUGAGCAUUUCACAGCCUCUGCAUGCUGGAGUUACUGC

>random_seq_from_cds__NO_3107

GCCUACAGUACAUGGUUCCCUGCCCAGGAGUGGUGAGGGCUGGACAUUGCACCCCUGGAGUAGGAAGUGAUGCUAUGCCCUUUGCAG

>random_seq_from_cds__NO_3117

CAGGUUGGCAAGGGUGCAGACCAGUGUCCAGAGUCAAUGCAGCGGAGCAUGUCCAAGCCCCUCACUCUGUAGCCGGGCUGGCACUCAAAUUUACA

>random_seq_from_cds__NO_3122

CAGAAGCCAAGCAUUCCAGCUUGCUGGGCCCAUUUACUUGGUACCCGUCAGUGCAGUGGAAGCUGC

>random_seq_from_cds__NO_3127

UUGUUGUUAGGUUCAUUAUCAGCCCAGUUCUCAGCCUCGUUGGUGAGAGCCUUUUUGGUUCCCACCCAUGUCCAUGUCUUAUUGUUCUUUCGGAUCC

>random_seq_from_cds__NO_3128

CAAUCCAGUAGUAGGAGCUGUAGUAGGGUAGGACCUUAUUGAGGUAAUCAAUUUCAUUUUUAUUCUGGAUGGCCACUAAGU

>random_seq_from_cds__NO_3129

CUGUGUAGCGAUUCUGGCAGUAUUUACGGGAAAUAUUCCAUGAGUAUGCUUUUGUGCUGUAAUGAUAAGUCCAUGCU

>random_seq_from_cds__NO_3145

UUAAAGGAUGUAAGAAGGCUUUUGGUAGCUUCCAUCUGAUUCAAGGCUUUGGCAGCUGCCUGGCAGGAACAAAUUUCUUUG

>random_seq_from_cds__NO_3147

CAAGGGAAUGUUGGACUCAGUGGGAGCCUUCACAGGUAGGUAGCAGGCCAGACCAGUGUCCUGUGGCUCCACAUGUCCG

>random_seq_from_cds__NO_3153

GGAUUUGCUGUGUCCACUGCCCUUGAGUGGUGCAUUCAACCUGGGCUGGUCCCUGCAACAUGAAGCCUUC

>random_seq_from_cds__NO_3164

CAGGUGUGUGUACCUUUGCUGACAAUAAGCACUGGCCUCAUCAUAAGUCAUAGCUUCCGUGGAGGUGUUGUAAGACCAGGCUCCACUCUCUUUAAUG

>random_seq_from_cds__NO_3166

UGAUUGGAGCUGGGGUCAGUGGCCUAAUUUCUCUGAAGUGCUGUGUGGAUGAGGGACUUGAGCCCACUUG

>random_seq_from_cds__NO_3175

GAACAACAGAUGAAUCGGUGGUUCAACCAUGAAAAUUAUGGCCUUGAGCCUCAAAACAAGAUACAUUAUGAAGGAACCUGUACUAAAUGA

>random_seq_from_cds__NO_3186

AAAAUGCUCAGAUUCUGCUGUCUCUGGCCAAUGGGAGGUGGUCACUAUGCAUGAAGAGAAGCAAGAGUCAGCCA

>random_seq_from_cds__NO_3233

GUUUCCAGCUGGUCCCGCUCCCGCCUCAGGGUGCCCAGCUCCCUCUGCAGCCCCUCCUGGGUCUCCUGGGGCCUGGCAGCCUGGUCCAAGGUCAAUU

>random_seq_from_cds__NO_3236

GCCUUCCUGAGCUGAGCUGUCCUGGCCCCCACAUCCCACACCAGGCAGGCCAGAAGCAGCAGCUGGACAGCUGGCAUCUC

>random_seq_from_cds__NO_3249

UCUACAUAGUGACUUCGAACUUGGCCUGAAGCUGAUGAAGGAACCAUCCUGCUUGAACGUCCGCAGCAGCGAGCCCAGCAUGAAGG

>random_seq_from_cds__NO_3254

AGAGACUUCAACAUCCCUGGCUUCCCGACUGUGAGGGUUCUUCAAGGCCUUUACCAAGAACGGCUCGGGAGCAGUAUUUCCAGGUGG

>random_seq_from_cds__NO_3258

CCUGCUGUUCCGGAAUGGCUCUGUCUCCCGAGUCCCCGUGGCUCAUGGAAUCCAGGUCCUUCUAUACCGCUUACCUGCAGA

>random_seq_from_cds__NO_3274

GGCUCUAUUCCCUGUCCUUCAUGGGCCUGCUGGCCAUGUACACCUACUUCCAGGCCAAGAUAAGGGCCCUGAAGGGCCAU

>random_seq_from_cds__NO_3278

GGUCACCAAGUCCUGUCACCUGUGCGACGCCGGGCAGCCCCACCUGCAGCACGGGGCAGCCUUCCUGACCGACUACAACAACCAGGCCGA

>random_seq_from_cds__NO_3302

ACUAUGGAGAUUCAACUGCAGGCACCUCCUCCGAUUGCCAACCCUGUCCGUGUCCUGGAGGUUCAAGUUGUGCUGUUGUUCCCAAGACAAAG

>random_seq_from_cds__NO_3308

UCUGCAGAGUGGGCAAGGCUGUGAGAGGGUGUGACUGCCAUGCCUUGGGCUCCACCAAUGGGCAGUGUGA

>random_seq_from_cds__NO_3312

CAGGAAUUAGAGAGUCUCAUAGCAAACCUUGGAACUGGGGAUGAGAUGGUGACAGAUCAAGCCUUCGAGGAUAGACUAAAGGAAGCAG

>random_seq_from_cds__NO_3328

CAAGAAGCCGAGAUCAAUGCCAGAAAAGCCAAAAACUCUGUUACUAGCCUCCUCAGCAUUAUUAAUGACCUCUUGGAGCAGCUGGGGGCAGCUGGAUACAGUGGACCUGA

>random_seq_from_cds__NO_3331

CCAUCUGGCUGCUUCAACACCCCGUCCAUUGAAAAGCCCUAGCCUAGACUUCUCUCCGAGUGCUUUCCAAAUCUGUAGUUGCGCAGUCU

>random_seq_from_cds__NO_3334

UGCCUCCACUUGGCUGCCUUUCUUAAGCUGAGGUUCUGUUGUCUGGUUAUUAGCAUCAGCUUUUUCACUUUCCUUGGGUUCAUCUGGA

>random_seq_from_cds__NO_3360

UACUUCUAUGCCUUUGUUGCAUCGGGCUGCAGCUCCUUGCCACUGCAGCUACAACAAUUCUGGCUGCUAGUUCCUUGUAAUAU

>random_seq_from_cds__NO_3377

AGGAUUUAUCAAGCUGUGCAGGGAGAUGUGGGGAAGGGUAUUCUAGAGAUGCCACCUGCAACUGUGAUUAUAACUGUC

>random_seq_from_cds__NO_3417

GCAUUCCGAGGGUCAUUAUUUCUGGAUGCUAAGUCCAUUCAGUCCACCAUCUCCAGCUCGCAGAAUUACUGAAGUUUGGG

>random_seq_from_cds__NO_3419

CUGGCGUUUUACCAAUGAUAUAAAAGAUGCAGGGUACCCCAAACCAAUUUUCAAAGGAUUUGGAGGACUAACUGGACAAAUAGUGGCAGCGCUUU

>random_seq_from_cds__NO_3436

CCGUAGAUGCUCAGGGACUUGAGGAGGGUAGAUCAUCUCUGCCUGAGUAUCUUUGACUGUGGGAGGAU

>random_seq_from_cds__NO_3441

UUUGUAGCCAUAGUCAGCAUUGUAAGUUGGUGGACUGUCAAUCAAAUGUGAUCUGGCAUGUCAACACAUAACUCAUAAUUGC

>random_seq_from_cds__NO_3445

AGCAGCAGGGCGCGGGCGAGCAUUCUAUGUUCUUAAGGGUUCUUGAUAAGACAGAGUGCCUUUGUCUUGGAAUACAUCUUGGAU

>random_seq_from_cds__NO_3452

CCCUUCAUAUUUCCACUUCAUUUCUAUGUUAUUUCUGUUCAUGUAAUCCACAUUAACAGUACAUGGUUCCCAAGCAAACAGGUGGGGAUG

>random_seq_from_cds__NO_3461

GCACUUUAAUUCUGGUACAUUUUGGUGUGAGAGACCAUCCGUAUGUGAGACAUUCUACCUCCUCUGUCUUCUUUCCUCCAGCUGUGUAGUAGCCAGU

>random_seq_from_cds__NO_3466

UUUUGUCUAUGCUCAUUGGAAAGUAAAAGCUUUUAAAAGUAUAGUAAUAUUGGGCAAUUCUUCCAUUUUCCACAUGAGGAAAACC

>random_seq_from_cds__NO_3468

GCCUUGGAGGAAUAAGGGUCUCCUGGGAGCCCUGGUGUUGGUCGAGGCUGCCCAGGGAGGACCCGAGGUUCAGGCAU

>random_seq_from_cds__NO_3489

UCCCAAACAUCCUGCAUGCCGAUGACAGCGUAGAUGAAGAAGAGCAUGACGAUGAGCAGAGCCACGUAGGGUAGGGCCCUG

>random_seq_from_cds__NO_3498

CAUUGGAGUCUAUGGCCUUGUACAGCAGCCUGAGGCCAUCCCUCGAAGGUGGAGACCGUGAAGAGGGACAUCAUGGCUGAGAGCACAUUGUCGAA

>random_seq_from_cds__NO_3511

GGCAGAAGUCAGGCUCUCCGCCUCGGCCAGGUUGUCCACGGCAAUGGCCAGGAAGACAUUGAGCAGGAUGUCA

>random_seq_from_cds__NO_3514

UGAGGGCUUGGGGAAAGUUGUCAAAGUUGCUGCGCCGUACUUCUGUGUCUUCAAAGUCAUACCUCCCCCCAAAGAGCUGCAUGC

>random_seq_from_cds__NO_3534

UCAGGCAGAACAAGGCCCGGGGUGGCCUUGGCAGAAUCUCAGGAACUGGCUUCUUGGGCUGUUUCUUCCUCAGGCCUUCAUCCUGGGG

>random_seq_from_cds__NO_3540

GCCAUAGCCUUUGGGCCCAUACUUCUUGCCGUAGCAGGACUUGCAGUAAAUCUCCUCACCAUGCACGGCCACAGUGGUACUGUCCAGAUU

>random_seq_from_cds__NO_3544

CCAAGGCGCCCGGCGGCGGCGGCGGCAUGGCCAAGGCCAGCGCGGCUGAGCUGAAGGUCUUCAAGUCCG

>random_seq_from_cds__NO_3546

AAAAAGCUGCAGCUUUAUGAGCCCGAAUGGAGCGACGAUAUGGCCAAGGCGCCCAAAGGCUUAGGCAAGGUGGGGUCCAAGGGCCGUGAAGCUCCGCUGAUGUCCAAGAC

>random_seq_from_cds__NO_3549

CUCUCCAGCAAGGCCAAGGCGCAAAAGAGCUCUGGGCCUGUCCCCUCUGCCAAGGGCCAGGAGGAGCGCGCCUUCCUCAAGGUG

>random_seq_from_cds__NO_3551

AAAGAGGACAGUGCAGAAUGUCCUGGAUCUCCGGCAGAACCUGGAAGAGACCAUGUCCAGCCUGCGAGGGUCCCAGGU

>random_seq_from_cds__NO_3552

GACUCACAGGCUCCCUGGAGAUGACCUGCUACGACAGCGAUGAUGCCAACCCACGCAGCGUGUCCAGCCUCUCCAACCGCUCGUCCCCUCUG

>random_seq_from_cds__NO_3571

CAUCCAGUGAUACCACCCAUGCUUCAAAGGUCCCAGAUCUGCAUGCUACAAGCUCAGCAUCUGGGGGCCCUCUCCCUUCCUGCUUCACCCCCA

>random_seq_from_cds__NO_3572

GUCCGGCACCCAUCCUCAAUAUUAACUCAGCCAGCUUCUCCCAGGGCCUGGAGCUAAUGAGUGGUUUCAGUGUGCCAAAAGAGACCCGC

>random_seq_from_cds__NO_3574

AGCAGUACUCCCGUCCCCACCCCACCUGCUCCCCCUGCUGCUCCCACAGAAGAAGAGACGGAAGAGCUGACUUGGAGUGGAAGCCC

>random_seq_from_cds__NO_3575

CAGAGCUGGGCAACUGGACAGGUAAUCAGCGGGAUCGGAACACUCUUCCCAAGAAAGGGCUCAGGGUACCAGCUUCAGUCCC

>random_seq_from_cds__NO_3584

AUCUCAAGCCUCAACAGCAUCACUAGCCAUUCCAGCAUCGGCAGCAGCAAGGAUGCUGAUGCGAAAAAGAAGAAAAAA

>random_seq_from_cds__NO_3594

UUCCAAGUGUUCAAGGGACUAUAUUUCUAAAAUGGACCCAGCCUCUACCCUGGGACUAAGCACUGAGUCCAUCCAUGGCUACA

>random_seq_from_cds__NO_3614

CAGCAGCUUGGCGAUCUCCAUCUCCGUCUUGCCUCCACAGAUGUGUCGCUGGUUGUGGAAGCGGAGCUCGGUCAGCGUGUUG

>random_seq_from_cds__NO_3617

AACUCCAGAGCCUCAGUAAACCGGACCAAGAUCUCAUUUGUGAUGCAGUCUGAGUUGUUGACGUUCACCUCAGUCAUCUCGGGGUCAUUGUUCUUCACUCUCUCCAGAGG

>random_seq_from_cds__NO_3618

CUCAUCAAAUAUGCUGGGAGCUGCCUCCUCCUCCACCUUGGCCGGUCCUUCAGAGGGCUUGGUGGGGCCACUGGGCGUCUGUUUCUC

>random_seq_from_cds__NO_3627

CACAGAAGUUGAGCAUGGCCUCCCGGUUGUACACACCCGUGGACUGUUUCUCCGUCUGGUUUCUCUGCCGCAGCC

>random_seq_from_cds__NO_3638

CGGGAUGCCACCUUCUGCUUCAUCGUGUCGCUGGCGGUGGCUGAUGUGGCCGUGGGUGCCCUGGUCAU

>random_seq_from_cds__NO_3641

GUGACCCCCCGGAGGGCGGCGGUGGCCAUAGCCGGCUGCUGGAUCCUCUCCUUCGUGGUGGGACUGACCCCUAUGUU

>random_seq_from_cds__NO_3649

GUCGGGCAGCUUUGCUGGAACAGCCGCCCCGCUGCACAGCUGUAGAAGCUGGACCGUUCCCGAGGAUU

>random_seq_from_cds__NO_3652

UGGAUGAGGGGGUAUCGGCCCUGGUUGCAGGAGAAGCCGGCAAAGUCAUCUAAGUCCAGUGCCCAGACCAUGGCCCCGCCCAGUCCCUUCUGCUUCA

>random_seq_from_cds__NO_3654

UGAUCCUGGAUUCUCUGUUUGGUGGCCCCCUUCCAGGAGCAGACCUUCAUAGUAGGCCAGCAUCCCUCCUUC

>random_seq_from_cds__NO_3658

AAUCCAGGUUCCUGGGCGAUUUUGUCCACCUCGUAUCCAGCAUCCACAUAGGUCUGCCCAGCUGGAACCGC

>random_seq_from_cds__NO_3661

GGCCGAGUUGACAAAGGUCUGACGGUUGUUGGCCGUGGCUACCAUAUCUGUGAACCUUCUGAGUGCCGAAAUUCCAGCCUCC

>random_seq_from_cds__NO_3670

UCAGGUCCAGCAAGAUCAGUGACCGGAGGCCCCUCAUGGAACUGCCCACUUCCUGGAUCUCAUUGUGU

>random_seq_from_cds__NO_3687

UGUCCUGGCUGAGAAAGCCACUGACUGUCCCUGUUGAAUAGCGGAGGGUGAGUUCUGUUCCAUUGUGCUUGUAGCUGGAGGAAUC

>random_seq_from_cds__NO_3699

CCAACCAUCUCAUUGUAGGUAGGAUUGCAGGUUUUCCGGGCCACUUUGGUUUUCCUCUUAGUGGUUUUCUGAGGGUCAGGAAGGAGGUAAAUUUUCACAUAGGGGUCAGG

>random_seq_from_cds__NO_3704

UGGAACUCCUCAAAGGUCCGCUGGAUGUAGGUGGCCUCGUGAGUGUUCUCUCGCAUCACCUUUACCACAUAUAUCAUAGCCUUUGUUGG

>random_seq_from_cds__NO_3708

GGCAUCGUACACAUACUUGAGGUCCUCCAGGUCUGAGAGUUCAGGGAUCCCACAGGACAACAUCCAGGCCCAGAAGGUUGAGGAAGAGGUGGGUGUGCUU

>random_seq_from_cds__NO_3720

GCCACAGCAGCACAGUAAGGCUGCCAGCAGAUACUGGUAGCGGAUGCUGAACUGAGAGUCCUUGAGGCCGU

>random_seq_from_cds__NO_3726

UGUCUUUAAGCUUGCGCUGGUCUUCUUCCCGGAGGCUGCCAAACUCAUAGCGGGGGCUGAACUUGUCUCCAGGGGGGCUGGUGAACUUGA

>random_seq_from_cds__NO_3736

CACGGCGGCCAGGGCGUUGCAGAUGGCCUUGACGGACUGGACCACCCUGUCAGCCUUCAGUGGGAAGUC

>random_seq_from_cds__NO_3739

GUAGCCGAAUGUCAAUGUCAAACUUGCGGCAGUAUUGGAUGUACUCAUGACUGCCCAAGGCAUGCUUGCUUCUGCAGGAAC

>random_seq_from_cds__NO_3745

CUCCUGGCCACGGGUUUUCCAGAGGUGUCUUUGCUGAAGUCCAGCGGCCCUCGGCCCUCCUUCCAGCCCC

>random_seq_from_cds__NO_3763

UUUGAAUGAGUCAGUAUCAGAGCAGUUAGGUGUUGGAAUAAAAGUUGAAGCUGCUGAUACUGAACAAACAAGUGAAGA

>random_seq_from_cds__NO_3771

GCCCUUGUCUCCUCUUCAGGCUUGGAGUUCAGCCCUGGGAUCCCAAACCACCUUCGGGCCUGUCUUUGAAGACCAGCCCC

>random_seq_from_cds__NO_3774

UGGGGGGCAACCUGGUCAUCAUGAACCCCACCAAGGCACAGGAUGCCGGGGUCUACCAGUGCCUGGCCUCCAACCCAGUGG

>random_seq_from_cds__NO_3791

CAGGUCCCCCAGGAGGUGUGGUGGUGAGGGACAUUGGCGACACCACCAUCCAGCUCAGCUGGAGCCGUGGCUUCGACAACCACAGCCCCAUCGCUAAGUACACCCUGCAA

>random_seq_from_cds__NO_3800

GUGACCGUGAGGGCCUACAACCGGGCUGGCACUGGGCCUGCCAGCCCUUCUGCCAACGCCACGACCAUGAAGCCCCGCUCCGCG

>random_seq_from_cds__NO_3804

AUCGUGAGGAAUGGAGGGCACAAGCAUGAUGGUGGAGAACAUGGCAGUCCGCCCAGCACCACACCCUGGCAC

>random_seq_from_cds__NO_3810

UCAUCCAUCAAGGAGGUUUGGACUGCAUCCGGUGGGGGGCCGUAAGGAUUUUCUUCUGGGUCUUCUACCUCAGGGGCAAUGG

>random_seq_from_cds__NO_3821

AUCUCGUGGAUGAUAUUGGACUUUUAAGAUGGGUGAAGGGAAUCGAAACCUCUGGUCACAGUCGCCUGAAAGAACAUCCCACUG

>random_seq_from_cds__NO_3831

CUAUAUUCAUGUACCUCGAGACCAUCUAGCUUAUCGAUAUGAGGUGCUGAAAAUUAUUGGCAAGGGGAGUUUUGGGCAGGUGGCCAGGGUCUAUGAUCAC

>random_seq_from_cds__NO_3837

CCAGUCUCGGUUCUACAGAGCUCCAGAAAUCAUCUUAGGAAGCCGCUACAGCACACCAAUUGACAUAUGGAGUUUUGGCUGCAU

>random_seq_from_cds__NO_3865

GGGGUGCAAGCCAUCUGCCUGGAGUUGCCACAGAGCCUUGUUGAAGGUGGGUGUCAGGUCAGGCAACGAAGACAUCUCCAG

>random_seq_from_cds__NO_3870

CCCUGGAGAGUUCGCCCUGAGGGAGAGGAUGGCACUGGCCACCUGAGCCACCUCCUGCUCUGUGACUGCGGGGCUGCUGAGAACUG

>random_seq_from_cds__NO_3880

CAGAUGCAGGUCUCCUGAAUGGAAGCUCCCGGGCGCCGGUUCCGGAAAUAGUGCAGCUGACACCGCUC

>random_seq_from_cds__NO_3888

ACUGGCACCGAACAUCCUGCCAGCUCUGAGGCCGACCCUGGCGGACCCGAGGGAAGGUGGAGGUGCAGUC

>random_seq_from_cds__NO_3890

UGGAAUCUCCUGUCCAGGUCCAGCUGCAGAGAGACAGGGUUCACAUCCAUUCUGUGACUGCCACCAGCGCAUGGGGCCGGAGGAU

>random_seq_from_cds__NO_3903

ACUUCCAUCUCCAGGGGUUCAGUUUUGGGCCACACUGCCUCCGGGCUGCAGUUGCCCACACUGCAAUUGCCCACACUGGCUGGCGCCAUGGGAGAACCCAUUGAUGUUCA

>random_seq_from_cds__NO_3904

GGAAGGGGAAGGUGUCCUGGAUGGGAACAUGGUGCUGCGACUGAUCCAGCUCAUCUUCCUCAUCUUCUUCAUCCACAUCAUUAU

>random_seq_from_cds__NO_3908

GUUGAGGGCUAUGCCGGGUGGCAUGUUUCCAGGGAAUCUGGAAGCGUUUAGAGUCCCUGUGUAGCCAGAUGA

>random_seq_from_cds__NO_3918

GACUCCAAGUCGAUCAACUUUGCAAAUAGGGAAAAGAGAUGCUAAUAGCAGUUUCUUUGACAAUUCUAGCAGUCCUCAUCUUUUGGAUCAAUUAAAAGC

>random_seq_from_cds__NO_3933

ACAGCAGAAAGCUGAGUUCUCAGAUCAGAAACAUCAGAAGGAAAUAGAAAAUAUGUGUUUGAAGACUUCUCAGCUUACUGGG

>random_seq_from_cds__NO_3939

AAGUCAGCGCAUUAGUAAGUUACAGGAAGACACUUCUGCUCACCAGAAUGUUGUUGCUGAAACCUUAAGUGCCCUUGAGAA

>random_seq_from_cds__NO_3956

GAAGACAUACCAGGAGGUGAAUUUGGUGAACAACCAAAUGAACAGCACCCUGUGUCUUUGGC

>random_seq_from_cds__NO_3959

CUGCAGACCUAUGUUGACUCAUUAAAGGCCGAAAAUUUGGUCUUGUCAACGAAUCUGAGAAACUUUC

>random_seq_from_cds__NO_3978

GGAGAAAACACAAGAGCUUGAGUCUCAUCAAAGUGAGUGUCUCCAUUGCAUUCAGGUGGCAGAGGCAGAGGUGAAGGAAAAGACG

>random_seq_from_cds__NO_3979

GAACUCCUUCAGACUUUGUCCUCUGAUGUGAGUGAGCUGUUAAAAGACAAAACUCAUCUCCAGGAAAAGCUGCAGAGUUUGGAAAAGGACUCAC

>random_seq_from_cds__NO_3996

UAUUGUUUUGCAAUCUUCAGUGAAUGGCCUCAUUCAAGAAGUAGAAGAUGGCAAGCAGAAACUGGAGAAGAAGGAUGA

>random_seq_from_cds__NO_4018

CCCACAGUGCUUGCUUCCCGGGUGGAGAGUGACACGACCAUUAAUGUUAUGAAAUGGAAGACGGUCUCCACGAUAUUCCUGGUG

>random_seq_from_cds__NO_4032

AAUAAUCAAUGCCUUGGUCGCUAUAUUGGUAAUCUCUGGGGUAGUCAUCCUGGUACUCGCCAUGAUACUCAUCAGGGUAUUCU

>random_seq_from_cds__NO_4035

AAAUUGUCGAGUUGUCCACCGGCUGUAAUGUUAGCUUCUGAUCAUAGAUGUCUCUUCUGGUACCUGGUGCUAACAUCCCUGCCCUGGCUGGCUCCUUUAUUAGUGCCCAU

>random_seq_from_cds__NO_4058

AUAUGCAGGAUACAUUACCUGUUUCACUAGUAGGACGAAUGGCAAUUGGUUCUGCCAGCUCGGUUUUGCCAGAUCUUGUAAC

>random_seq_from_cds__NO_4059

CCAAGCAACCCUCUGGGGCAAAGUCAGCAACAUGAGUCUUCUCUUUCUCUAAUGCACUUUGAGACACAAACAUGGGGAAGUA

>random_seq_from_cds__NO_4071

AGCCACUCUAUUGUAAAGGACCAAGGAAUCCUCAGAUGUAGUACAAUUAUUAUUCAGAGAAGGUGUUGGUCUUUCCUUAAAAGGAGCAGA

>random_seq_from_cds__NO_4084

AAACUGCUCAUCUCUGUCAUGGUAUUCUGUUGUUCUCAGGGCAUGUGUAACACCUUCGAUGCUGUCAACUAUGGGGCAGGCAAA

>random_seq_from_cds__NO_4085

AUCAUAUGUUGGAUAAACACUUGUAUUUAUUUCCAGUUCUUGGAUGUGGUUGAAUUUUGCAGCGAUAAAGGGUUGGAUCUCUCAUGCAUCCAUUG

>random_seq_from_cds__NO_4102

CUGAUAGCCGACUGGCCGGUGGUGGUCUUGGGCAUGUGCACCAUGUUCAUCGUAGUCUGUGCCUUGGUUGGAGUAUUAGUGCCAGAGCU

>random_seq_from_cds__NO_4111

CACGCCAGCUUUAAAAUACAGCAUGCUCUUCUCUCCCACAGAGAAAGGGGAGAGCAUGAUGAACAUUUACUUGGACAACUU

>random_seq_from_cds__NO_4140

CUUGAACUAUGGCCCACACUCUUGCCAGCAGAUGGGGGACUGCUUGUGCCACCAGUGCUCUCCUACCACUAGCAGCUUUGUCCAGAUCCAAAAC

>random_seq_from_cds__NO_4142

UGCAGGGCAGAGUAAAGCCAGCCGGAAUGCAGAAUUCUCUGCCUAGGAAUUUUUUCCUCCACCCAGUGCAGC

>random_seq_from_cds__NO_4155

UAUGAAGCGCUAUCAGGGGGUGCCACCACUGAGGGCUUCGAAGACUUCACCGGAGGCAUUGCUGAGUGGUAUGAGUU

>random_seq_from_cds__NO_4167

AGAUCUCUGCCUUUGAGCUGCAGACCAUCCUGAGAAGGGUUCUAGCAAAGCGGCCAAGAUAUCAAGU

>random_seq_from_cds__NO_4168

CAGAUGGCUUCAGCAUCGAGACAUGCAAAAUUAUGGUUGACAUGCUAGAUGUCGGACGGGAGUGGCAAGCUGGGGCUGAAGGAGUU

>random_seq_from_cds__NO_4172

AUCCUCCUCACUUCAGUGCUGGGCUUUGCCAUCUACUGGUUCAUCUCCCGGGACAAAGAGGAAACUUUGCCACUUGAAGAUGGGUGGUGGGGGCCAGGCACGAGGUCCGC

>random_seq_from_cds__NO_4187

UGUCGGUGCUGGAGCGGCAAUGACCUAUGGCUGGAGCCUCCUUGGCACGAGCGCACCAUCCGAGGCACAGCUGCACUUCUGCAC

>random_seq_from_cds__NO_4191

GCAGCGGGUGCCCUCGGUCAUUGGUGCUUCAGGGUCACAGUCGCCCUGAGCUCCCAUAGUCCCCAAGGUCCAGGGUGUGC

>random_seq_from_cds__NO_4207

GGGAGGUGCGGUUGGAGCCGUCGUCGCGGACGCGCAGCCACUCGACGGUCACCCGGGCCUGGGCGCUGCGCGGGGACAGCCGCCCGUGCCUGUG

>random_seq_from_cds__NO_4209

CAUGCCGAACACCAGCAGGUGUGUGCUGGCCUCCGACGCCAGGAACCUGCCGGCCACCUCCUCGGAAGCUCUGGCUGAACCUCU

>random_seq_from_cds__NO_4216

UUUGCAGGGAGGCAAGUGAGGCCUAUCUGGUUGGCCUUUUUGAAGACACCAACCUGUGUGCUAUCCAUGCCAAACGUGUAACAAUU

>random_seq_from_cds__NO_4233

CUCCGCCGCGCCCCCAGCCGCCGCCUCCCCGGGCUCGGGCUGCUCCCCUGAGGCGCCCGGGCCGCGACCGGAUCCAGGUGGCGAGGGCG

>random_seq_from_cds__NO_4243

CUCAUAGUUGACAUCGAUGGGAUCCUUGCUGCUAUCAUCAGACCCUCCCCUGAGCAGACUGUAGGCCACCUCGAUGUCCAGC

>random_seq_from_cds__NO_4251

UUGUAGUAGGAGUUGGUUCCUUUAACGAUGUCCACCAGGCCAAGGGUGGCACUGAAGACCUUCCCACCUUUCUCCAGGACAUGCGCAGAGUGUUCCAGUCCCAGAAUCAG

>random_seq_from_cds__NO_4267

GCCUUGCUACCAAUUCCAUCCUGGCCUUUGCCCUGUCACUCCUCCAGCUUCCGCUGUCUUCUUGACUUUCUGCUGGUCAUCCCACCGAAGCUCAGAGAACCCAUCCACCU

>random_seq_from_cds__NO_4274

CUUCAUUGGUGGUGCUGCCCACCUUGAGUGCCGUCUCAUACUUGUGCUUCAGAUGCUUGCCCACCUCAUGGAAAGGCGCCAA

>random_seq_from_cds__NO_4275

CUGCCGCCAGCAGGUCCGCACCGUGCAUGAGCCUGACACGCCGUGGCACUUGCAGGUGGUCUCCACCCCAGCC

>random_seq_from_cds__NO_4284

ACCCGAUCUGGUGGGUCGCUGGCUGUUGGGCCACAGUAUUCCUCCCUGGGCUCGCAGCCCAUCCUGUGUGCCAGCAUCCCGGGCCUGG

>random_seq_from_cds__NO_4287

UUGCCUCAGCCGGUGUGGCCUUUGCAGUGACACGCUCAUGUGCAGAAGGCACGGCCGCCAUCUGUGGCUGCAGCAGCCGCCACCA

>random_seq_from_cds__NO_4297

GCCAGGACAAGAUCCGGCCCCUGUGGCGCCACUACUUCCAGAACACACAAGGGCCUGAUCUUCGUGGUGGACAGC

>random_seq_from_cds__NO_4304

CGGGGCACUUGGGGACCCUGUCUUUCCGGCUCACGUUGUCCCUGCACACACCCAGGGCCCACAACGCGUCCC

>random_seq_from_cds__NO_4306

AUCGGUCCUUGCUGCAGAACCCACUGCCCUCCGGCGAAGAGCCGAGGUAGCGCCUCUGGCGGCUCUCAUACAGGAGGAGGUAGGGGUACGCGGAGGU

>random_seq_from_cds__NO_4309

UCCGCUCCUCCAGCUGCAGCAGCAGCAGCUCCAGAGAGUGACCCUGCCGGUCCAGGCAGGCCACGCUCUCCCGGAGCCUGCUGGCAGUCUCCUCUUCUUCCGUCUCCAGA

>random_seq_from_cds__NO_4313

AAAAGCUUGAGGGGCUCGUGGUGCUCCUGGCACAGGUCUUGCUUCUGCAGACCAGGAUGCUGCUGCGCCAUCUCGGCCACCUUGGUCAGCAGCCG

>random_seq_from_cds__NO_4316

GCUUCCUCCUGCAGUUUUCUGGCGAGUUCCACAGCCUCCAUUCUAGAAGCAUUUGCGGUGGACGAUGGAAG

>random_seq_from_cds__NO_4322

CGCCAUCUCGUUCUCGAAGUCCAGGGCCACGUAGCACAGCUUCUCCUUGAUGUCGCGCACGAUCUCGCGCUCAGCCU

>random_seq_from_cds__NO_4333

AUCAUCUUUGCCAUCAGAACUGGACCAGCUUAUCUCUCUGAAGAGCUUUGCAAAGGAUUUCCAGUUUUAGAUCAUUUA

>random_seq_from_cds__NO_4337

UCAAGCCAAGAAGGGUUUUCUUCUUUGCAAAGUAACGAGUUUCCAUAUUUGCCAAACCCAGAAGUGUUGCAUGAGCCCUUUUCUAAUUCUUGGCU

>random_seq_from_cds__NO_4348

UUGUUCAAAGCAAUCAAUAUGGCUGUGUUGACAAGGUCAGAAAGCCGGGAGUGGUGGUUCUUGAGAACAAUGG

>random_seq_from_cds__NO_4359

GAUGAGACAUGGAUUGUCUGCUGAGUGCCAUGCUGCUGCCAAAAUCACCAGCCCAUCACCAGUUUUGCUUCAAGUCCAAAUAUCGAA

>random_seq_from_cds__NO_4367

CCAAAUAAUGAGCUUCUCUUUGCACACCAGACAAGCCCAUCCACCUUCAUCUAUGUUAAUGGUCAGCUGGUCAUCGACCUUCAGCCAAUGUUAGGGC

>random_seq_from_cds__NO_4391

CAGCCCCAGGAGCUUCCGGGCCUCCGGGAGUCCGGCCGCUGCGGGGCGCAGCCGCCCCUUGUCCCCGGGAGGCGCCGCCGGCCCGCGCCG

>random_seq_from_cds__NO_4400

CCGUUGGGAGAAAAGGCGAGAGAAAGCACGGGGCCACGGUGGCCUGUGAAAAGCCUCACCGAGUUCCCCUGCUGAGCGCUCCACAGCCGG

>random_seq_from_cds__NO_4402

UAUAUCCUCAGCGGGUACGUCCGAUCAAAUGACCACAGCCUGGCGGUGCGGUCGUGGGACCCGCUGGCGAAGUAC

>random_seq_from_cds__NO_4410

CACAGGGCAGUAUUGUUGUCACUUUGGAGGUAGCGGAUAAGGUAGUUGUAGCUGUCUUCUUGGAGACGGACCACGUACUUGUUAUC

>random_seq_from_cds__NO_4411

UAGGAAUGCUCGAAGCUUGAAGUUAGAUAGGAUGUCCUGGAUGGUUUGAGUGGUCUGUAGCUGCUCAAU

>random_seq_from_cds__NO_4412

GACAUCCUUCUGGCUAGCAUUCUGCAGAAACAUUCCAUGGAAGCGGCUGUAAAAACUUUCCACUGUGCUCUUCGGACUGUUUUGGACCAGGUUGAGAUGGAGGUAGACAA

>random_seq_from_cds__NO_4414

CAGUCGUCCAAACUGUACUUCAUAUUGCUGGGGUUCUGCCUGGCAAGGGGCUGCAGACACUAUGUUGGCACAACCAGAUUC

>random_seq_from_cds__NO_4416

CCAUCUGAGUCCACGUACUGCCGGCGUUUGAGGUAGCAGGACACUGCCAUCUGAAUCUGCUCGGUACGCACUCGUUUCAUAAUGCGG

>random_seq_from_cds__NO_4423

UGAUCGCUGAGGACGGGGCUCUCUUGGGGAAAAUUGAGAAAGUGCGAGUUCUUAGAAAUGAUCGACGAGAAGGGCCUCAUGCGCUCACGGGUUC

>random_seq_from_cds__NO_4432

UUGCUGAUGGUGUGGUUGGAGUUUAUGAAUGUCACAAUGCUGGGGGAAACCAGGGAAUGGGCCUUGACGAAGGAGAAGUCGGUGAAGCACAUGGA

>random_seq_from_cds__NO_4434

AAAUGGGAACAGAUCGAGGGCAACUCCAAGCUGAGGCACGUGGGCAGCAACCUGUGCCUGGACAGUCGCACGGCCAAGAGCGGGGGCCU

>random_seq_from_cds__NO_4457

GCUGAUUGUGCUCAGGUAUGCGGAUGAGGAGCUCCAGCUGGACUUCGAUGACUUCCUCAACUGCCUGGUCCGGCUGGAGAAUGCGAGCCGGGGUGUU

>random_seq_from_cds__NO_4462

AGGUGCUCCUGAUAUGGAAGAUAUAUUGACUGAAUCAGAAAUUAAAUUGGAUGGUGUCAGACAAAAGAUAUUCCAGGUAGCCCAAGA

>random_seq_from_cds__NO_4470

GCUUCCUGGUGCUGGGCUACUUGCUCUACCUGGUCUUCGGCGCAGUGGUCUUCUCCUCGGUGGAGCUGCCCUAUGAGGACCUGC

>random_seq_from_cds__NO_4472

GGCGUGUCGGUGCUCAGCAACGCCUCGGGCAACUGGAACUGGGACUUCACCUCCGCGCUCUUCUUCGCCAGC

>random_seq_from_cds__NO_4474

UCACCCUCCUGUUCCUGACGGCUGUGGUCCAGCGCAUCACCGUGCACGUCACCCGCAGGCCGGUCCUCUACUUCCACAUCC

>random_seq_from_cds__NO_4503

UGAUCAGAAGAAAGAACUGUGAGGGCUUCUAGUGCAGACUGCAAAGUCCUUAUUGGCAUCUGAACGGCAGGUAGAAACGGU

>random_seq_from_cds__NO_4504

UCCAGAAUUUCACUCCCUGUGGUUGGUAUAAGGGUGUGAUAUUUUUUCAACAGGAAAGAGAGGCACACCCAUUGAUCAUGAAUAUAUU

>random_seq_from_cds__NO_4527

CCGCCGACACCUCCACCGCCCUCUGCAGCAGGUAGCGCGCUCGCUUGCGCGUCAGGGCGUCCGCCUGGCCC

>random_seq_from_cds__NO_4532

GCCCGUCCUCGCGGGGCCGCAAACAUGGCCCGACGGCUGCUAGCACUUCCACGGCAGCCUCGGCGCCAGGCGCGCGCCACCCGGCGAGC

>random_seq_from_cds__NO_4537

ACCCACUCCAUAAUGAGUGACACUUUGACAGCGGAUGUCAUUGGUCGAAGAGUUGAAGUUAAUGGAGAACAUGCAACAGUA

>random_seq_from_cds__NO_4553

UGCCGGGGCUCCAUGACAAUUCAAAAGGUGAAGGGAUUGCUGUCACGUCUUCUCAAAGUUCCUGUGUCAGACCUUCUGUUGUCCUAUGAAAGUCCCAAAGAAGCCGGGCA

>random_seq_from_cds__NO_4557

CUCCCACCUAAGGAAAGCCGGCACCCAGAUUGAGAACAUCGAGGAAGACUUCAGGAAUGGCCUUAAGCUCAUGCUGCUUUUGGAAG

>random_seq_from_cds__NO_4563

UCGUGAACACCCCUAAACCCGAUGAAAGAGCCAUCAUGACGUACGUCUCUUGCUUCUACCACGCUUUUGCGGGCGCGG

>random_seq_from_cds__NO_4567

CUGCGGAUCAGCAACCGUCCUGCCUUCAUGCCCUCCGAGGGCAAGAUGGUGUCGGGAUAUUGCUGGUGCCUG

>random_seq_from_cds__NO_4581

GAUGAAUGAGUUCAGAGCCUCCUUCAACCACUUUGACAGGGAGGAAGAAUGGCCUGAUGGAUCAUGAGGAUUUCA

>random_seq_from_cds__NO_4582

GAGCCUGCCUGAUUUCCAUGGGUUAUGACCUGGGGUGAAGCCGAAUUUGCCCGCAUUAUGACCCUGGUAGAUCCCAACGGGCAAGGC

>random_seq_from_cds__NO_4588

CUGAAAGGCAACAAUGACAUUUUAAGUAUAACUCAGCCUGAUGUCAUUUACCAAAUCCAUAAGGGAAUACUUGCUGGCUGGGGCAGAUAUCAUUGAAA

>random_seq_from_cds__NO_4593

UGCCAAUGCCAAGGGCAGCCUUGUUUGCACUCCAAAAUCUUUUUGAGGAGAAAUAUGCUCCCCGGCCUAUCUU

>random_seq_from_cds__NO_4594

UGAUUUCAGGGACGAUCGUUGAUAAAAGUGGGCGGACUCUUUCCGGACAGACAGGAGAGGGAUUUGUCAUCAGCGUGUCUCAUG

>random_seq_from_cds__NO_4597

UGAUGGCCAAGCACCUAAAGGGAUUUUGCUAUGGAUGGCUUGGUCAAUAUAGUUGGAGGAUGCUGUGGGUCAACACCA

>random_seq_from_cds__NO_4602

CCAAUUUUGCUGUGAUUGAAGCUGGGUUAAAGUGCUGCCAAGGGAAGUGCAUUGUCAAUAGCAUUAGUCUGAAGGAAGGAGAGGAC

>random_seq_from_cds__NO_4607

AGAAGCAAUGCAUGGGGUUUUCCUUUACCAUGCAAUCAAGGUCUGGCAUGGACAUGGGGAUAGUGAAUGCUGGAAACCUCCCUGUGUAU

>random_seq_from_cds__NO_4612

GCUGUUGGCCACCUUAUCCCUUUCAUGGAAAAAGAAAGAGAAGAAACCAGAGUGCUUAACGGCACAGUAG

>random_seq_from_cds__NO_4633

CUGGCAGACUGACACGCUGCAGUGCAGAUGCACCCGGUCCCCUGAGGGCCUGUUUCUCCACUGUAGGGUUCACAAAGCUGAAGGUGA

>random_seq_from_cds__NO_4634

AGAUGCUGAAGCGCUGGUGGUGAGAGGGAAAUGGAAGAUCCAAGGCUUUCUGGACAGGGAUCAGCUGGG

>random_seq_from_cds__NO_4637

GGUAGUCACCAACACCGUAGUAAGAGCCAUAGUUUUUAUCCUUUGGCAAUCUGAAGUUCCAGAGUGAGGGGUCCAGGCUGGGUCUCAGGAAAGGGUGGUGGGAGAGUGA

>random_seq_from_cds__NO_4664

AGCUUUAAUAACUGGGGUCUGGCAUGCGUUCUGUCACACCUCCAAUCUUAAAGUUCAUCGUAGAUCUC

>random_seq_from_cds__NO_4672

AGCCCCGGGGCAAAGAGCCUGAUGAGACUGUGCACAGUUUUGCCCAGCGCCGCCUUGGACCUGAGGGUGGCGUCUCUAGCCAUGGACAGUCUCUGCCGUGGAGUGUUUGC

>random_seq_from_cds__NO_4678

CCAUCUGCCUGUCCAGGGGAUUUGGACAUUUGGUGCCAUCUUCAGAAGAUCCAGGAGUCCUGGGAAUCGUGUAUGACU

>random_seq_from_cds__NO_4694

CAAGCCAGACAGGAGCUUGGAUCUAGGAGGCAGUAUGGUAAUAAAAUUUGUGGAUGCGCUUCAGGAGCGUGGUUU

>random_seq_from_cds__NO_4699

UGGGUGGCGUUGGUAGGAUGGAUCAGAAUAUUGCCAAGUACAAGGUGAAGAUCCGAGGCAUGAAGUGGUACUCAAGCUUUAUUG

>random_seq_from_cds__NO_4707

CUUUCUCCUGCGUGUCCCUGCUUCUCCUCGCCGGUCUCCUCCGCAGGGUUCUCUUCUUCUUGUUUCUGGGACAUGUUAGCCGUC

>random_seq_from_cds__NO_4708

UUCUUUUGGGGGUGUGUACCAGCCCGUUUUUUUCGUGGAUCUGCACCAAGGACUUGUAGGACUGCUGUGCUCUUGUCAGACUGUAUUGAGACUUUGUUGGCU

>random_seq_from_cds__NO_4717

GGGUCAUCAGACUCCACCGACCAGAUGGGGGGCACAGCAGGGUAUGACUCCCGUGAUGUUGCAGUGGAUGCGGACAGGAUCCCC

>random_seq_from_cds__NO_4719

GAACUCGCAGCUCAGCUCGUCCAGGCAGGCGCUGGCAAUGCGGAAGCGCUCGUGGCCGCGGUGGAAGAUGGACUCGAGCAG

>random_seq_from_cds__NO_4728

CAAGCCAGGGUCACUGUUCAUGGUCUGAACGAUUUCCAUGCCCCUGGUUGAGCACAACCCAGUUUGGGUCAAUCUGAGCAUCACCCUCA

>random_seq_from_cds__NO_4757

GAUGAAAAUUUUAAACUUAGACACUCAGCUCCAGGCCUGCUUUCCAUGGGCGAACAGUGGUCCAAGUACAAAUGGCUGUCAGUUCU

>random_seq_from_cds__NO_4763

GUGCUGGUGCCUGCUGCUAAAGUUUGUCCUGCUCCCGAAGCACUGGUCACACAACCAACUGAAUUGCAGAGAGUAAUAGUAAACUCA

>random_seq_from_cds__NO_4768

AGUGAUGGUUGUCAUUGUUUGAGGAGGUUUUAAUGCAUUUUCAUUGGCCGAUUCUACAAAUGAAUGAGGACUGAGCCA

>random_seq_from_cds__NO_4770

UUCAUAUCUUAUAAUUAUUCCCAUUUAGUUCCGCUGGUGGAGACCAUUCUACAUGAAGUUCUGUAGAACUGAUUUUCUGCAUCUUAGGU

>random_seq_from_cds__NO_4772

CAGAAAAAUCGUACUUGGCAAAUGGAACCAGAUUCCAGAUGGUAGCUGAGGUUUCAUGACCUUCGUAGGAAACAC

>random_seq_from_cds__NO_4779

UUACAAGGCUGACAUCCUUCCAGUCUGAGGAUCAAAUCCAAAGUAAUGGUCUUUGCAUUGGUCACAACGUUGCCCAGCAAUGGAAGCAUCUUGG

>random_seq_from_cds__NO_4780

CAAACACACUGACCAGUCAGGCUAUUACAGAUGUGAUUAACUGCACCAGUUGUAUGGCAUGAGCAUGGCAGGCAGCCA

>random_seq_from_cds__NO_4787

AAGUCCUUUGGCUUCUUUUUUGCACUCACACUGCCCAGAGUGAGGAUUGCAGAAUUUGUUCACUGAGCCAUGGAGGUUACACUGGCAG

>random_seq_from_cds__NO_4794

UGGAAUGGCUGUUGCAUUGACAAGGUUUACAAUUGAAAGCGUAAACUUGAUCACCUUGGCGGAAAGGCUUGUCAUUAUAAA

>random_seq_from_cds__NO_4803

UUCAACCGUGACACUCUAUUAUCAGCUGUGUCUCCUGCAUCAUUAGGAAUGCAGUACCGCUGUGCCAAAGGGUGGACCCGCGGGUGGCUG

>random_seq_from_cds__NO_4838

ACUAAAGGCCUGUGAGGCUGGAAGAGUGACACGCUAUGGGAUUGCAGGUCCUUGGUCACUACCUCCGGCGGCAGCGAGAUCCCA

>random_seq_from_cds__NO_4841

GAUGGGAUUCUUUUUGGGAUGGUGGGGGCCUAUGACUGGGGAGGCUCUGUGCUAUGGCUUGAAGGAGGCCACCGCCUUUUCCCCCCACGAAUGGCAC

>random_seq_from_cds__NO_4849

UCCAUCUGACCCCAUCACUGGAGGUGACCCCACAGGCCAUCAGUGUGGUUCAGAGGGACUGUAGGCGGCGAG

>random_seq_from_cds__NO_4852

CUACACUUCCAUGUGCUGGGAUACAUCAGAUUACCUCCGGCCAGUGGCCUUGACUGUGACCUUUGCCUUGGACAAUACUACAAAGC

>random_seq_from_cds__NO_4853

CAGGGCCUGUGCUGAAUGAGGGCUCACCCACCUCUAUACAAAAGCUGGGUCCCCUUCUCAAAGGAUUGUGGCCCUGACAAUGAAUGUG

>random_seq_from_cds__NO_4855

CGGAAAGUGCUGGUAUCUACAACUCUGGAGAACAGAAAGGAAAAUGCUUACAAUACGAGCCUGAGUCUCAUCUUCUCUAGAAACCUCCACCUGGCCAGUCUCACUCCUCA

>random_seq_from_cds__NO_4864

CCGAAGAGGGCAGUGUCCUACAGCUGACUGAAGCCUCCCGUUGGAGUGAGGAGCCUCUUGGAGGUGGUUCAGACCCGGCCUAUCCUCAUCUCCCUGUG

>random_seq_from_cds__NO_4865

GAUCCUCAUAGGCAGUGUCCUGGGAGGGUUGCUCCUGCUUGCUCUCCUUGUCUUCUGCCUGUGGAAGG

>random_seq_from_cds__NO_4881

UCAGCACCCUGGCUCUGGCCAUGGCGGGAGGAAGGCUGGCUUGGUCCUGCCUCAGAGCCCCAUUGCUCACACUCUUCCUUGGACC

>random_seq_from_cds__NO_4885

GGAGCGUGUUGCGGCGAGCCAUGGGGAGGUGGAGCGACUUGAGCGUCAUGGCCUCCAUGCCCCGCACCAU

>random_seq_from_cds__NO_4886

GCUGUUCAUCACCUCCAGGCUGUGGCGCUUGUUCACAGUGCCGUGGUGCUUGGUGGCCGUGAGGG

>random_seq_from_cds__NO_4888

GGGUCACCAGGUCCGGGCUGCUGCCGCUGACGUACUUGUGGCGGUGGCUGGCCAGGUCUGGGGUGCUGGUGGCAGGUCGUG

>random_seq_from_cds__NO_4891

AUUAUUCUUUGGGUUCCUCUGGUCAGAUGGACUGACAAGUUUGUUGCUGUAGACCCCCUGUGGCCCAUAAGGGACAGUGUAGG

>random_seq_from_cds__NO_4892

GGUGCCUCUCCCGCAUCUCCGGUUGGCUGUACACCAGAUCCUCUGGCUGGUUGUAGGCAUGGGUGUUGAUAAUGUUGAGGU

>random_seq_from_cds__NO_4906

GGCAUUCUUGCCCUGUGCUUUCCACCGACAGCGUGCACUCGAUAACAUUGCUGUCCAGCAGGCGAAUCCG

>random_seq_from_cds__NO_4911

CAUCAUCAUCAUCGGUGUGGGUUGUCUCUUCAUGAGUUAGUUGCUGUCAUUCUUGAUGACGACCUCUAAUCCGUGGUGCC

>random_seq_from_cds__NO_4928

AACCCAUCGGACUCCCCAUCUCGAUCCCAGGCUGCAGGAUCCAGUAGCAGCUGGGGCACCUGGCCCAGAGGUUCUGCCAC

>random_seq_from_cds__NO_4931

CAGCAGGAUCCCAGCCCUCCUUUCCAGUCUCCCCUUGAGGGGGUGGCCUCUGCUCUCCAGUGGGGUCCCACCUGGGGGAGGUGGGGG

>random_seq_from_cds__NO_4932

ACCUGAGGUAGAAGCCCAGGGGAGUGGAGUCUGGAAGGGUCUCCCCGAGCUCAUCCUCCUCGCUCUCUUCUCUGCUCUCCU

>random_seq_from_cds__NO_4939

GGCCUGGCUCAGCUCCCGCAGCAGACUCACCCAUGGCAGGCUCUGACCCCAACAUGACCUCUGGGGAGGCUUGGUCACCCCCUGGGGCCACAUCA

>random_seq_from_cds__NO_4950

CCCUAGAGACCUCCGUCGCUGUUGAGUCUCUUUUUCAAGAGUUCUCAAUGUCUCUUGGUCCUGUUCUUCUA

>random_seq_from_cds__NO_4957

UCUCCCUUUCUAGUGUCUCAUGGCUCUGGUUUUCCAGAGUCUUCAGUGACUCUUGAAUCUCCUCUCCCAGAGACUUCAGGGU

>random_seq_from_cds__NO_4961

GGGACUGGCCUGUACUGGCCUCUUGCCGCUGGCCCCCAGGCUCCUCUGGUCCAGGCAGGACGCUGGCAGGAAUGGCCACCCUG

>random_seq_from_cds__NO_4963

CUGAGGUGUGGGGGGGAUGGGGGUGCUGGCCAAGGUAGGGGUACGGGCCUGGAGGAAUUCUUGGUUCUUAA

>random_seq_from_cds__NO_4970

CUGGCCCAGCGACGUCUCCAUGUGUGCCACGCGCUCCUGGUAGCCGCGCACUGCCCCGCGCCACGCCUCGCCCAGUCGCCUUGCCAGCU

>random_seq_from_cds__NO_4974

CCAGGUUGUCGCGCGCCACCUCGGCCGCGUGCUUCUCCCGCCAGCGUUGGUCAACGAGGGCCCGCAGGGCCGCCA

>random_seq_from_cds__NO_4997

UGUGUUUCCCAAGGCCUCCCAUCUGAAUGAGACAUUGUCAUCUGCAUCCUCCACAGAGCAAGUCAGAUGGAGCUCACAGGU

>random_seq_from_cds__NO_4999

GUGUAACUGGACAGCUUUGCAGAGGUCUUUGUGGAUAUCUGGGCUCUGUAAGAGCCUGUGUCUUCCAUCUUCAGGUUGCUGA

>random_seq_from_cds__NO_5002

AACUCCAGGGGAAGAGUUACUGACUCCCCCAGAAUCCCGUUCACCAUCAAUGGGGUUAAGCUGCUUUGUGAAACUACAUUCCCCUG

>random_seq_from_cds__NO_5019

UCAUUGGGAGAAGCUGUCUUUGCUCGGUGCUUAUCAUCUCUGAAGGAUGAGAGAAUUCAAGCUAGCAAAAAGCUGAAGGGUCCCCAGAA

>random_seq_from_cds__NO_5031

CCCUUCAUCUCAGGGUCCAGAGGUGACUGGUGUGGAUCCUGCUCGCUGUGCCAAGAUGGCCCUCCAGAUGCGGCAUACCAUCCCUCCCCCUGAUGCGGGUGUAGAUGAGG

>random_seq_from_cds__NO_5034

GCACCAGGGCCUGGUACUCCCGUUCCUUCCCCGCCAGGAUUUCGCGCAGCCCGAUCAGUCUCUGCCCUC

>random_seq_from_cds__NO_5039

CUGGGCGAGCUGCCGGCGGUUGGGAGUGUGGAUGUGUGCCCCGAGGCAGCGCAGCAGCUCUUCCACAUG

>random_seq_from_cds__NO_5046

CCAUCUCAAUGACAGUGCAGCCCAGUGACCAGAUGUCAGCUGCUUUCCCAUACCCGCGUGGGCCCUGGUCAAUGAUUUCUGGGGCCAUAUACUGCAGAGUUCCCUGUGAA

>random_seq_from_cds__NO_5078

ACUUCUCCGGCGGUGGUGUCUGUGGAUGGGCGGCCCGUGAGACUCCAACUCUGUGACACUGCCGGACAGGGA

>random_seq_from_cds__NO_5101

CCCUUCCUCGUCGUGGUACCCAACCUGUGGGUUCUCUAGAACUAAAGUCUGCCUGCCAGGUGACUGCUGGA

>random_seq_from_cds__NO_5103

CUUGAGUCAUGUCCUGGUGAGACGGUUGUGGCAGAAGAUCCCGCCGGGCUGAAGGGUCCCUUUGCUACUACACCAGAAGCAGGCAUUGGCUU

>random_seq_from_cds__NO_5111

AGUCAGGUUGACAAUGGCUCAAAGAAAGGAGGAGAACGGUUAAGUAUUUUAACCAAGAGCCUUUUGCUGAGGAGAACAAAAGACCAGCUGGACUC

>random_seq_from_cds__NO_5117

UUUCCCUUAACGGCACCUUCUUCAAGAUGGAGCUUUUUGAAGGCAUGCGAGAGAGCACCAAGGAUUUCAUCUCUGUUGGCAGAAUUG

>random_seq_from_cds__NO_5128

GAUGAAAAUGAGGCACAAGCUGGCUGGAUUGAGGGGGCAGCCAUCCUUUUCUCAGUGAUCAUCGUGGUGUUAGUG

>random_seq_from_cds__NO_5129

ACUGCCUUUAAUGAUUGGAGCAAAGAGAAGCAAUUCCGGGGGCUGCAGUGCCGCAUUGAACAGGAGC

>random_seq_from_cds__NO_5137

GUACUCCCAUCUACAUCCAGUACUUUGUCAAGUUCUUCAUCAUCGGCAUCACUGUACUGGUGGUGGCUGUGCCAG

>random_seq_from_cds__NO_5149

AACGAGAAAGGCGAGGGUAGAGCAAGAAAAGCUGGACAAGAUCUGGCCUAAGCUUCGGGUCCUGGCGCGAUCUUCUCCCACUGACAAGCACACCCUGGUGAAAGGGCAUA

>random_seq_from_cds__NO_5151

GGAUGUUGGUUUUGCCAUGGGGCAUCGCAGGCACAGAUGUAGCAAAGGAGGCUUCAGACAUCAUCCUAACAGAUGACAACUU

>random_seq_from_cds__NO_5152

CACCAGCAUUGUGAAGGCAGUGAUGUGGGGACGAAAUGUCUAUGACAGCAUCUCCAAGUUCCUGCAGUUCCAG

>random_seq_from_cds__NO_5156

CAUUCUAUCAGCUCAUUGUCAUCUUUAUCCUUGUCUUUGCGGGGUGAGAAAUUCUUUGAUAUUGAUAGUGGGAGGAAGGCAC

>random_seq_from_cds__NO_5164

UAAGUUUGGGACUAGGGUGCUCCUGUUGGAUGGUGAGGUCACUCCAUAUGCCAAUACAAACAACAAUGCGGUGGAUUGCAACCAAGUGC

>random_seq_from_cds__NO_5182

GGGACGGAGCGGGAGGCCGAGGCUGGGGGCAGGUCCCGGCGCACGCCCAUAAGCAGAAUCCCCAGGGCCAGACUGACUCCCA

>random_seq_from_cds__NO_5209

AACAUCUGAUGAUUCUGGGCUAGAGCUUGAUAAUAACAAUGCAGCAAUGGCAAUUGAUCCAGUAAUGGAUGGUGCUAGUGAAAUUGAAUUAGUAUUCAGGCCUCAUCCCA

>random_seq_from_cds__NO_5210

CACUUAUGGAAAAAGAUGACAGUGCACAGACGAGGAUACAUAAAGACUUCUGGUAACGCCACUGUUGAUCACUUAUCCAAGUAUCUGGC

>random_seq_from_cds__NO_5225

AAAGAGUCUUGACAUUGGAAUGGCUGAGGAUGUAGGAACAAAAUUCUGCAACUUGUUCUAUGAAAUCAGGGUUCAGCU

>random_seq_from_cds__NO_5232

GUGUUCUGGCUUCUUGGGAUGAGGCACACACCACAUCCAGAUUCCCUUGGUGUGAGACUUCACUGUGGAGC

>random_seq_from_cds__NO_5244

UCUGAAUCUCGUUACCUCCCAGCAGACCAGGAGUAUACUGUUGGCUGUUAGGAAUAUGUUGUGGC

>random_seq_from_cds__NO_5252

AACAACCUACAGAAUAUCAUCUAUAACCCGGGUAAUCCCGUUUGUUGGCACCAUUCCUGAUCAGCUGGAUCCUGGAACUUUGAUUGUGA

>random_seq_from_cds__NO_5270

CCGCAUGCCCUAUAUCUCAGACAAGCACCCUCGACAAACCUUGGAAGUGAUUAACCUUCUGAGAAAGCACCGGGAGCUAUGUGAUGUGGUGCUAGUUGU

>random_seq_from_cds__NO_5276

GUGUUCAAUGCAGUGAUGGCCUGGGUCAAAUACAGUAUUCAGGAAAGACGUCCUCAAUUACCCCAGGGUGCUGCAGCAUG

>random_seq_from_cds__NO_5280

CAGACCAAUGAAUGGAGAAUGGUGGCUUCAAUGAGCAAAAGGAGAUGCGGAGUUGGGGUCAGUGUUCUUGAUGAUCUGUUAU

>random_seq_from_cds__NO_5281

AUGCAGUAGGAGGCCAUGAUGGAUCCUCUUAUCUCAAUAGUGUUGAAAGGGUAUGACCCCAAAACAAACCAGUGGAGCAGUGAUGUGGCCCCUACAAGCACCUGCAGGAC

>random_seq_from_cds__NO_5294

GAUGGCCAUGGCAGUCAGUGGCAGCAGAAAGAGGCAGCAGAAGGUGAAGAGGUUAUAGGUGGUCUCUUGCCAUUGAGCCU

>random_seq_from_cds__NO_5296

UGCCCCCAGAAGUUUCCUUACACCUGAACGGGAUCCAAGCGGGUUGAGUACUGCUGCCUGGCGGUCCAAUCC

>random_seq_from_cds__NO_5301

UGCAGCUGUUCUACUUUCCUUUUCUCUUGGAGCCCUGGCCGAUCGGGCAUUGAUGAGAACAAGGGCUGUGUAGAGGGCAAUCUC

>random_seq_from_cds__NO_5307

GGUGUGCUGCGGAAACUGGGGCUGCCGUAGCUGUCUGGGCCCUGUCCCAGUUCCCCAAGCCCAG

>random_seq_from_cds__NO_5309

CUCUCUCUGCCCUCAGCCUUGCCCCGCUCAGGGCUGUAUUCAAGGUGGCAUGAGGCCCCAUUGAGCCCUGCCUUGGCCAAGUUGU

>random_seq_from_cds__NO_5311

GGCAGCUGCCCGUCUGGGAGCCCCAAGGUGUAGGUGAGGGUAUCUGCUCCUUGGGCCCCUGCUGGAGGGGUCUUGACCACUG

>random_seq_from_cds__NO_5316

UGCAGGUGUGGGUCUUCUUUGCAGCCAGCAGCUCCCCGUGAGGCUCGGUGCUGUCUCUGUGGGGCCCUGUCCAUUCUAUUUCCAGCGCCC

>random_seq_from_cds__NO_5322

CUCUGCCCGACGUCUCUCCGAUCCUGUCUUUGAGAGAAACGAGCUCCUCCUCCUCUUUCUUCCUGUUCUCAAAGUGAGCCUCGAUCAGCGCCUG

>random_seq_from_cds__NO_5332

ACCUCGAUCAUCUUCUGGUACAUGUCCUUCCGCAGGCUGGGCUUCUUCCGGGCCUUCGUGAGCUCCUCCUCCAGGUAGGU

>random_seq_from_cds__NO_5334

UCUGGUUGUAGCGCUCCCCGUCCUUCACCACAUCCCCAUGGUAGGCAGGUACGAAGGGCCUCAGCACAUCC

>random_seq_from_cds__NO_5335

ACCAUCAGCCGGUCCAGGCAGCGCUGCUCUGACUCACAGUGCUUCUUCAGGAUCCUGCCAUUGG

>random_seq_from_cds__NO_5339

CCCGUGGAGGAGGCCGAGGAAGAGGACAGUUUCCUCAGGGGCAGGUUGCCCCGAGGGCUUCCCUGCGUCUCCUCCAAGGCC

>random_seq_from_cds__NO_5346

CACAGUCGUCUCCUCUGGCCUUUUGCCCACUUCAGGCUCCCCAGAGCCCGGCAUGCCACAGGGCAGAUAUC

>random_seq_from_cds__NO_5348

CUGCCUCUCCACCAGGGCCUCUGGGGGCUGCAGGUCCUCAAGCUCACGGGCUCUCCCAGACGGCUCAGUGAGGGCAAGAUCCUGUG

>random_seq_from_cds__NO_5360

GCAGUACACAGCCAUCAUGCAGCGCGCUGGCUCCAGCGGUGGCCGCGGGGAAUGUGACAUCAGCGGCGCCGGGCGCUUGGGG

>random_seq_from_cds__NO_5361

CUGGAGGAGGCAGCUCGCCUCAGCUGCGCUGUGCACACCUCGCCCGGGGGAGGACGCAGACCCGGGCAGGCGGCAGGGAUGUCGGCGAAGGAGAGGCCAAAGGGCAAAGU

>random_seq_from_cds__NO_5381

CUCCUGCACUGGCUCCAUCCGCUAUAAAACCUUGACAGACCAUGAGCCCAGUGGGAUAGUGAGGGUUGAGGCUCACCCAGAGAA

>random_seq_from_cds__NO_5390

CUGUGUCCACAGCGUUCAAGGCAAACAGUGAGUUUUUCCAGAUUCUCAGUCAGAAUGGAGCACCUCAGGCAGAUGUGAGCAUGUACAGUCUU

>random_seq_from_cds__NO_5402

UGGGGAGCAGCGGGUCGCAGGAGCAGCUUGUCAGCAUCGCCUCCUCCAGUGAGGCCAGUGGGCACCGUGUGGAGGAGACGAAGGCGGAGCAGGAUGACCUUGCAGCAGGU

>random_seq_from_cds__NO_5409

CCAUGUCCCACCCCCAGAGACAGGCCAGGGAUGCUACCCUCUUCUGUGAGCCCUGGACCCUGAACAUGCAGCC

>random_seq_from_cds__NO_5415

CAGCCCCCGGAACUGCACCGGAAGGCCUGCAUGGGCUGCCCUUGUCCGAGGGCUUGCAGCCUUACCCAGCUUUCCCUUUUC

>random_seq_from_cds__NO_5430

CACCCAGUGAGCCUCCUGCUGCAGGUGCCCAAUGUGGGAGGCGGCUUGGAGGCCUGGCACAUCCGGGGUUG

>random_seq_from_cds__NO_5437

GCCCAGAACUUUGGUCUCCAGCAUCUCUCCAGCGGCCACUUCUUGCGGGAGAACAUCAAGGCCAGCACCGGAAGUUGGU

>random_seq_from_cds__NO_5467

CCGUCGCUAUAUCCGCAGACUCCGGGCUAUCCGGGAGCUCAAUAGGAACUGUAGAAGAUUUGAAAAAUAAUGAAAGCCAAUG

>random_seq_from_cds__NO_5469

CUGUGCUGAUGCUGGCCUACUUGACGAGAGCUUCCUGAGAAGAUGUCUGAAUUUUUAUGGCCUUCUCAUUCAGCUGCUGCUCCGCAUCCUGGACCCCGCAUA

>random_seq_from_cds__NO_5479

AGCAACUUUGUGGCCCCAAGUGCCGUGACCUGAAAGUUGAAAACCCUGAGAAAUACGGCUUUGAACCAAAGAAGCUGUUGGACCAACUGA

>random_seq_from_cds__NO_5480

CGGAUAUUUACUUACAGCUGGACUGUGCUCGGUUCGCGAAAGCCAUUGCUGACGACCAGGAGAUCCUACAGUAAGGAAUUGUU

>random_seq_from_cds__NO_5488

AGGCCUGGGGAUGGCCGAGGGAAUGGGCAGUUGACUGGAAGAUUUAGCAGCUGCUCCCUGCGGCCGGGGCAG

>random_seq_from_cds__NO_5491

GAGGGCCUCUUUCCAGAUGUAGCCCGGAGAGCCCUGACUGACCCCUUCCUAUCAUUACUCCGGAGUCGAGGCGUCAGGCUGCUUGGGGAGGGGGUGCUCCGCGUCAAAGA

>random_seq_from_cds__NO_5498

GUGCAUUUGCCCAGUAUAACAUGGAUCAGUUCACGCCUGUGAAGAUAGAAGGAUAUGAAGAUCAGGGUCUUAAUUACAGAGCAC

>random_seq_from_cds__NO_5518

GCUGAGCAUUUCCCUGGGACCAGGAGACAGAGAUGUGGCCUAUUCCUGCAUUGUCUCCAACCCUGUCAG

>random_seq_from_cds__NO_5519

CUGGGACUUGGCCACAGUCACGCCCUGGGAUAGCUGUCAUCAUGAGGCAGGCACCAGGGAAGGCCUCCUACAAAGAUGUGCUG

>random_seq_from_cds__NO_5530

UAUAUUCUUUAAGAACCAAUUCCCAGUCUAAGUCUGGAAUGGUCAAGUUUGCAAGAGUUCCCAAACAUUCAAUCACAAACUCCUCUUCUUCAUCAUUAGAGAUCUGGGCU

>random_seq_from_cds__NO_5535

GAAGCAGUCCAACUUGUACCAUCUUAUUCCUCAGUCCUGUGUCAAAGGAUAGGUUUAGUAAAAGUCGGAGGGUGAUAUUCAGCAG

>random_seq_from_cds__NO_5549

UUGCAUCUCAUGCUGUGCUCAGCGGGUUGGCCACGCGGCCCAGGAAGUGCAGGGCAGUGGCGCUUUGAUCAUACACAGCAAACAGGA

>random_seq_from_cds__NO_5553

AGGUCAGAGGCAUAGUGAGGCUGGAUCAGCAGCAGGCAGGCGCUCUCAGUGAAGGGCACUUGAGUCACCGAGAAGUUGUCCUGGAUGUCACUCCAGUGCUGGAAGGUGC

>random_seq_from_cds__NO_5556

CCAGUCUUCCAUCCUGUCACAGCCUGCAUGAACCUGUCAAUCUUCUCAGCAGCAACAUCCAGUUCUGUGAAGUCCAGAGAGCGUGGGAGGACC

>random_seq_from_cds__NO_5557

ACAGGGGUAUAGAGAGCCAGGCCCUGCACAAACGGCUGCUUCAGGUGCAGGCCUGGGGCUGUGAACACGCC

>random_seq_from_cds__NO_5560

GCCUGUCAGCUGUGUGGUCCAAGGCUCCCAGAUAGAGAGAGGCCAGGGUGCCAAAGACAGCCGUUGGGGAGAGGACGG

>random_seq_from_cds__NO_5578

GCUCCAGCUUCUGGUCAUGAUUCACCAGCUGUCCACCCUGCGGGACCAGCUCCUGACAGCCCACUCGGAGCAGAAGAACAUGGCUGCCA

>random_seq_from_cds__NO_5588

UUCCCCGAGUCCCGAAACAGCAGCCACAUCAAGAGGCCCAUGAACGCCUUCAUGGUGUGGGCCAAGGAUGAGCGGAG

>random_seq_from_cds__NO_5603

CACUGGCCACCUUCAGAAAUAUGUCCCGUGGUUCCCCGGGGCCCCACAUUCUGGGUCUCACAGAUGAC

>random_seq_from_cds__NO_5607

CACUGCGCUGGACUUCCAAGGUGACACGGCGUCCAUGCCCUUCUCGUUGCUCAACCCAGGAGAGGCCCUGCACCAUGGCCAGCUGCC

>random_seq_from_cds__NO_5612

CCUUCGUGCGUUCCCGGUAGGCGGCGAUGACCUGUAGGUCCCGCAAGUGCUGUGUGCGGACGCUCUGCUGCGCCCAACACGCUCGUCCCGCGCCGGCCC

>random_seq_from_cds__NO_5624

CGUCACAGGUUCUGUAGCGCUGACAGCCUCGGCUGGCUGUUUAUUGGCAUCCAGAAUCUCGGGAAAGC

>random_seq_from_cds__NO_5625

CGGAGGACUGCAGGAGCACCAUGGAGGCUCUCGAGGGCUGCCCUGCAGAGCCUCCUGGUCUGGGUUCCCACAACAAUAGGGGCAUGC

>random_seq_from_cds__NO_5627

CUCCUGCUGCCUGGGCCGGGGAGGCCUGAGAGCCAUGGGGUAGCUGUGAAGUAGGCCUGGCCAAGCAGUGCUGAGUCGGGGA

>random_seq_from_cds__NO_5629

GCGCUGGCGGGCCUCGAAGGGGACGUGGGUGGUUUCCUGGAAGGCCGCCGCUCCACUUUGGGGCCACUGACAGGCUCCGU

>random_seq_from_cds__NO_5634

CGGAGCCUGGUCUUGGAAGAGGAGACCACUUUCCUGCUGAAGCUAGCUGAGCGCGUCAAGGCCACAUCCAUCUCAGGCACCAGUACAACG

>random_seq_from_cds__NO_5641

CCUGGGAAGCAGAGAUAGGAGAGUCCGGCUGGUUGCUGAAGAUCCGAAGAAUUCCAAACCCACAGGACAAUGUCUGGAGGCUCCC

>random_seq_from_cds__NO_5648

UCCACGGGGACUGGGGCUGGCUCACGUAUCCGGCUCAUGGGGUGGGACUCCAUCAACGAGGUGG

>random_seq_from_cds__NO_5652

CACCAUUGCGGCCGACGAGAGCUUCACAGGUGCCGACCUUGGUGUGCGGCGUCUCAAGCUCAACACGGAGGUGCGCAGUGUGGG

>random_seq_from_cds__NO_5655

GGAGGUGAGGGGCCAGUGCGUGCGGCACUCAGAGGAGCGGGACACACCCAAGAUGUACUGCAGCGCGGAGGGCGAGUGGCUCGUGCC

>random_seq_from_cds__NO_5681

CCCGCCGGCACCUCUGAUCUACUUCUGCAUGUCACACUGCAGCAGCAACACAAUGGAUGGGUCACUCUUGGCUGCCUCCUUGAACUCCUCCAAUGUA

>random_seq_from_cds__NO_5688

ACCGUGGGUAUGCAGACAGUCCUUCAAAAGGCAGGAGCAGCUCCAUAUGUGCAGGCAUUUGACUCGCUGCUUGCUGGUCCUGUGGCAGAGUACU

>random_seq_from_cds__NO_5694

CCUGGAGCAAAACGGGGGCCUGUGGCAAAAGAACUGAGCGGACUGCCAUCUGGACCCUCUGCCGGAUCAUGUCCUCCUCCCCCUCCACCAUGCCCCCCUCCUCCCCCAGU

>random_seq_from_cds__NO_5700

UUGGCCUGGUAUUCGAUGACGUGGUGGGCAUUGUGGAGAUAAUCAACAGUAAGGAUGUCAAAGUUCAGGGUAAUG

>random_seq_from_cds__NO_5727

UCUGCCCCUGCCAAAACAGACUGUGUGCAUCUCCUUUCAGAAGUUCAACGGUAUCCCCGGCCUGGAGCUGUAAAGGGGGUCCUUCAUGCAGAGCUGG

>random_seq_from_cds__NO_5744

UUCCACAAGGGUUUCACUCUAUUAAAUCAGGAAGGCCUUUGUAGAUGUCUUCAUCAUUAAUGCUUUCUUCUGUUGGGAAGGGCCCUGAUUCCUGUGGCCA

>random_seq_from_cds__NO_5746

AGUUCACUUUUCCUCAUUCCAAACGUCUCACAACAGGCCGUGAGAAAUGUCCUUAUGUUCUUCAAACAGAGAAACCUGGGACAUCUGCGGCCUCAGG

>random_seq_from_cds__NO_5778

UGGUCGGGGGUUCCAGAUAGAUUCGACCCCGCAUGGCCAGCUCUAUCAGGAUGCCCCCUCGCAGGCCUGAU

>random_seq_from_cds__NO_5780

CCAUAAGAGUAAGGCGGAUAUCCUUAGAGUCUCCAGAAUCUUCAUUGUCUGGACUUUUCUCCCAAUUACUGUCUUCCUCACUUU

>random_seq_from_cds__NO_5793

AAUUGGGAGCCAGGUCCUGAUUCCUCUUCAGCAAGGCCUCACUGAAGGAAGUUUCAUCAGGUGCUGGCUUGACCCGGGG

>random_seq_from_cds__NO_5795

UCUGUCACCCCCUCAUAAUGAGCCUCUUUGAUCUCUUCCGGGGCUUUUUCGGCUUUCCUGGACCUCGGAGGCCACAGAGAUCCCUUUUUUGGAGGGAUGACUCGAGAUG

>random_seq_from_cds__NO_5817

AUUAUGAAACUGUCCUGUCCCUGGGAAUUCCCAUUCCCUAAGCCAGACAUGAUCACCCGUUUGGAAGGGGAGGAGGAGUCUCAGAAUUCUGACGA

>random_seq_from_cds__NO_5824

AAGGGUUCAAGGCAAGCUUGGGUUGGUGUCGAAGAAUGAUGAGAAGGUAUGACCUGUCUCUGAGGCAUAAAGUGCCCGUGC

>random_seq_from_cds__NO_5826

CGCAUGACUAUGAGGUAGCUCAGAUGGGGAAUGCAGAUGAGACGCCCAUUUGUUUAGAGGUGC

>random_seq_from_cds__NO_5830

GACUGAAGACUUGAUGCAGGACUGGUUGGAAGUGGUGUGGAGACGGAGGACAGGAGCAGUGCCCAAGCAGCGAGGGA

>random_seq_from_cds__NO_5847

CCGGAGGUCUGCUUCUCGGGGACGUGGCUCCCAACUUUGAGGCCAAUACCACCGUCGGCCGCAUCCGUUUC

>random_seq_from_cds__NO_5848

CACGACUUUCUGGGAGACUCGAUGGGGCAUUCUCUUCUCCCACCCUCGGGACUUUACCCCAGUGUGC

>random_seq_from_cds__NO_5851

AGCAGAGAAGGAUGAAAAGGGCAUGCCUGUGACAGCUCGUGUGGGUGUUUGUUUUUGGUCCUGAUAAGAAGC

>random_seq_from_cds__NO_5865

GACAGUUAAUGAAUGGGAGCUUCUCCAUGGACGGGACAGGACAAUCCUAUCAGGACUUGAGGGAUGGGAGCCCCUAUGGAAUCCCCCA

>random_seq_from_cds__NO_5866

GUCUCCAUCCUCCAUAUCGUCCCUGCCAUCCCACGCUCCUUUGCUCAAUGGGCUGGAUUACACGGUGGACAGUAAUUUGGGCAUCAU

>random_seq_from_cds__NO_5871

UAUAUCAUUAGCUUUAAAAGAGAUGAGCACCUUCAAGACAAUGCUGCCUGCCCGGUCCCUUCACUGCCUGACUCUUGGUGUUGAUAG

>random_seq_from_cds__NO_5873

AUCUUCUUCGUCCACGAACUUGUUCUCGUCAUAUUCAUCCACGUCCACCUUCCGGAAGCGGGCCGACGACACUGUGUUCUUCGACAUA

>random_seq_from_cds__NO_5874

UCAGUGGGUCGGAGAGGCAUCCUCCAAGCCCAGCAGCUCUCUGACGAGUCUCUCUCCAAACUGCGCCCGGCUGUA

>random_seq_from_cds__NO_5879

CAUGGACAGUGGGAGGCCGGUGCAGUCACUCUUCCGCGCGCGGAGCAAGCCGCAGCAGUAAAUCCCCUUGCUUCUCAAACU

>random_seq_from_cds__NO_5889

AUGGAUUAUAGGGCGGCCGAGGCGUCGACUCCAUCCACAGCUGUCGCUGCCUCUGGCCCAAGGAGCUC

>random_seq_from_cds__NO_5926

AGAUAUCACAGAAACCACUGGUAGAGAGAACACUAAUGAAGCCUCCUCUGAAGGGAACUCUUCUGAUGAUUCUGAAGAUGAG

>random_seq_from_cds__NO_5940

CUGUGAUGCAUCCAUCUGGUGAUAGUGACACAACGAUGUUAGAAUCUGAAUGUCAAGCUCCUGUACAGAAGGGAUAU

>random_seq_from_cds__NO_5948

GCCGCUGCCGCCGCCGUCCGGGUCUGCGCCCGCCACGCCGAGGCCCAGGCGGCCGCGCGGCAGGGAA

>random_seq_from_cds__NO_5949

CUGGCGCUGAAGACCCUGGGGACAGAUGGCCUUUUUCUCUUUUCCUCCUUGGACACUGACGGGGAUAUGUACAUCAGCCCUGAGGAGUUCAAACCCAUUG

>random_seq_from_cds__NO_5950

CUGAGAAGCUAACAGGGGUCUUGUUCUGUCACCCAGACUGGAGUGCAGUGGUGCAGUCACAGCUCACUGCAGCCUCAACUUCCC

>random_seq_from_cds__NO_5978

CACUCUUUGUAACUUGGAAACUCAGGAGCCUUUGGAAAGUACUGCUGCAGUUUGCUCCAAGCCUCUUUAGAAACAAGCCUUCUU

>random_seq_from_cds__NO_5992

AGCUGCAAGUUCAGAGUGCAAGGAAGGCUAAGAAGUCGAAUCUUUCUUGUUGCAUUCUGUUUGCUUUGACAGU

>random_seq_from_cds__NO_6016

AUUUUGUAUUUUUCAUCAAUAAAGAACUUGAGUUUGCCAAUCAUAGGGCGGUAUAUUCUGUGGAGAUCCUUCAGGGCUGCCCCCCAGUGUGAGCUCUGAGGCUUCC

>random_seq_from_cds__NO_6028

UCCACUGCAGCCCUUCUACCGCUGGAGGACGUGGGUCCCUCCUGGGGGUUGUUAUGAUCCCUGCUCUCCAUGAUGUCGUCG

>random_seq_from_cds__NO_6031

CUUCAAAUGAACAACAAGAGCUUUUCUGUCAGAAGUUGCAGCAGUGUUGUAUACUGUUUGAUUUCAUGGACUCUGUUUCAGACUUGAAGAGCA

>random_seq_from_cds__NO_6042

UUGUACUUCUGGAAUAACGAAUAUAUUCUUAGUUUGAUUGAGGAGAACAUUGAUAAAAUUCUGCCAAUUAUGUUUGCCAGUUUGUACAAAAUUUCC

>random_seq_from_cds__NO_6050

UGCCCAUGGGGGCCCUGCUUGCCUGGGUACCCAGGGGGCCCAGGAGGUCCUGGAGGACCCAUCAUGCCCACCGCACCCAGGGCUUCCCGCUUGGCACUCACGGCGACCUC

>random_seq_from_cds__NO_6059

UCCCAUCCUUGCCGUUGAUGCCUGGGGGGCCCCGUUGCUCCUUUCGGGCCUGUGAUCCCCUGGGGUCCACGAAUACCUG

>random_seq_from_cds__NO_6062

CCCCGGUGGUCCAGGGAUGCCUUGCUCUCCAGAGGCACCCACAUCUCCCUUGGGACCCCGGCUUCCCCUG

>random_seq_from_cds__NO_6065

UGGGGGCCCCGAUGGUCCAUCUGGUCCAGGGUCCCCCCUUGGGGCCUCGGAUUCCAAUCUCACCAGGGAGGCCAACAG

>random_seq_from_cds__NO_6070

UCCCGGUGGACCUCUCAAUCUGCGCCAGAGCGAGCACUACCACCUGGAGGAGAACAAGGAGGCUGCGGGGGGAGGCCGUAGC

>random_seq_from_cds__NO_6076

AGAUUGUGAAACCUGGCUUCAACAUCAGCAUUCUGAAAAUAUUCAUCACCAUGAUGUCUGAGAGUGUUCGGAUGAUGCUGGAAC

>random_seq_from_cds__NO_6078

AAGUGUGCCUUCAGCCACCAGGGCAGCAUCCAGUUGGACAGGUACCCUGGACUCAUACCUGAAAGCAGUGUUCAACCUUAGCAAAAUC

>random_seq_from_cds__NO_6099

GCCAGAGGCUGGAAUGUGGACAGUGAAGGACCUCAAGCAGUGGAAGGCACUCUGUUCGCAUUACUGGCCUCAGUA

>random_seq_from_cds__NO_6108

AAUGUUACAGUCACUCCUGGAGAGAGAGCAGUUUUAACAUGUCUCAUCAUCAGUGCGGUGGAUUACAAUC

>random_seq_from_cds__NO_6149

GAGAAAAGGAAAUCAAAUAUGAAGUUGAUGUCUUGGGUGCCACCAGCUAUAGAAGGAGGAGAUGAAACAUCUUACUUCAUUGUG

>random_seq_from_cds__NO_6155

AUGGAACCACCUAGUCUGGAAGAUGCUGGAAAAAUGCUGAAUGAGACUGUGUUGGUGAGCAACCCUGUACAGCUGGAGUGUAAGGC

>random_seq_from_cds__NO_6170

UCAUGUAUCUGACACAGGCCGUUAUGUGUGUGUUGCUGUGAAUGUAGCAGGAAUGACUGACAAAAAAUAUGACUUAAGUGUCCAUGG

>random_seq_from_cds__NO_6173

CUACGGCUGAUGCAGACCACAAUGGAAGAUGCUGGCCAAUAUACUUGCGUUGUAAGGAAUGCAGCUGGUGAAGAAAGAAAAAUC

>random_seq_from_cds__NO_6177

UGGCUUCCAGUCCAGCUGGCCACAAGAGCAGGAGCUUCAGUCUUAAUGUAUUUGGUAUCUCCUACAAUUGCUGGUGUAGGUAGUGAUGGCAACCCU

>random_seq_from_cds__NO_6192

UGGUGAACAAUUUCAUCUCUUUGACCUGUGAGGUCUCUGGUUUUCCACCUCCUGACCUCAGCUGGCUCAAGAAUGAACAGCCCAUCAAACUGAACACAAAUACUCUCAUU

>random_seq_from_cds__NO_6209

UACUGAUGGAACCCCAGCUCCCAGUAUGGCCUGGCUUAGAGAUGGCCAGCCUCUGGGGCUUGAUGCCCAUCUGACAGUCA

>random_seq_from_cds__NO_6215

AACUGAUGAGCCCCGGGAUAUCACUGUGUUACGGAACAGACAAGUGACAUUGGAAUGCAAGUCAGA

>random_seq_from_cds__NO_6218

CUACAAGAGAAUUUAUUCUCACUGUAAAUGGUUCCUCCAAACAUAAAGGGGGGCCCCCAGAGCCUUGUAAUUCUUUUAAAUA

>random_seq_from_cds__NO_6230

CAUGCAGUUCUUCCUAGUGGCGGCUUACAGAUCUCCAGAGCUGUCCGAGAGGAUGCUGGCACUUACA

>random_seq_from_cds__NO_6231

UGUGUGUGGCCCAGAACCCGGCUGGUACAGCCUUGGGCAAAAUCAAGUUAAAUGUCCAAGGUUCCUCCAGUCAUUAGCCCUCAUCUAAAG

>random_seq_from_cds__NO_6232

GAAUAUGUUAUUGCUGUGGACAAGCCCAUCACGUUAUCCUGUGAAGCAGAUGGCCUCCCUCCGCCUGACAUUACAUGGCAUAAA

>random_seq_from_cds__NO_6237

AUGUUGUGGCUGGAGGAUUCUGGCUUCUAUACCUGUGUUGCUAACAAUGCUGCAGGUGAAGAUACACACACUGUCAGCCUGACUGUG

>random_seq_from_cds__NO_6249

AGCCUUGUCCAGGUGGAUGGUAGCUGGUCGGAAUGGAGUCUUUGGGAAGAAUGCACAAGGAGCUGUGGACGCGGCAACCAAACCA

>random_seq_from_cds__NO_6262

GGUUCAGCAAUGAGAAAGAUAGUUUCUAUUCUAAAUCCCAUUUAUUGGACAACAGCAAAGGAAAUAGGAGAAGCAGUCAAUGGCUUUACC

>random_seq_from_cds__NO_6277

CUUCAAGUGUAUCUGUCCACCAGGACAACAUUUAUUAGGGGACGGGAAAUCUUGCGCUGGAUUGGAGAGGCUGCCAAAUUAUGG

>random_seq_from_cds__NO_6283

GGGGUUCUGCCUCAAGAACUGUCCACCCAAUGAUUUGGAAUGUGCCUUGAGCCCAUAUGCCUUGGAA

>random_seq_from_cds__NO_6284

UACAAACUCGUCUCCCUCCCAUUUGGAAUAGCCACCAAUCAAGAUUUAAUCCGGCUGGUUGCAUACACACAGGAUGGAGUGAUG

>random_seq_from_cds__NO_6285

CAUCCCAGGACAACUUUCCUCAUGGUAGAUGAGGAACAGACUGUUCCUUUUGCCUUGAGGGAUGAAAACCUGAAAGGAGUGGUGUAUA

>random_seq_from_cds__NO_6295

CUGUGGGCUGCUCCAGGUCUGAGGUCUCAGGGGCUGGGCUCUUGGCUUCAUCCUUGGGACACACAAGGGACUCCAGCAG

>random_seq_from_cds__NO_6301

CCCCAUAGGAGGGAGGCCAGCUGUCCGGAGUGGCUUGAGGGGCAUAGGAGGAAGGCUGGACCUUAGAGAUGGCC

>random_seq_from_cds__NO_6311

GGGUAGCUGUGCUGCAGAGAGCUGAACCUGUCAGUCAUCUUGGUGGCUGACCGGCCUCCCGCACUGACAGCGGUGACCCUGGCAUAG

>random_seq_from_cds__NO_6315

CCGUCAGCUCCAGGCGGUCCUGGUGGCCGCUGCCACUGCUGCUGCUGCUGCUGCUGCUCCUGGGUCC

>random_seq_from_cds__NO_6320

CUGUCUUUGCCCAGAGCAUCCCGUGGAACCUGGAGCGGAUUACCCCUCCACGGUACCGGGCGGAUGAAUACCAGCCCCCCGGACGG

>random_seq_from_cds__NO_6322

CAUGGUCACCGACUUCGAGAAUGUGCCCGAGGAGGACGGGACCCGCUUCCACAGACAGGGCCAGCAAGUGUGACAGUCAUGGC

>random_seq_from_cds__NO_6356

GCCCAUGCUGACAGCCUCACCCAGGAUGCUGCUCCCUUCUUCUUCCUCGAAGCCUCCAGGCCUGGGCACAGAGACACCGCUGUC

>random_seq_from_cds__NO_6372

AAGCCCUAGGAGGGGAAGGCUGGGAGAAGCUAUCAUUGGUGCCCUCCUCUGCCCGCCUGCAUUAUGGCAC

>random_seq_from_cds__NO_6394

UUCCCGGUUGAUCGUGGAUUAUUUGAGGGAUAAAGUAGCCAAUAUUUGGUGCAGCUUCAAUGUCUUUCUGAAGAUUAAGGAUAUUU

>random_seq_from_cds__NO_6405

GUGGGAAGGAGCACUCCCCCUCACAGUAAUAGGCCGAGUAGCCUUGGGGAGCGAUGACCCAGUCCCAGCCAGC

>random_seq_from_cds__NO_6410

GUGACAUCCAGCACCAGCCAGCCCUCGUCUCCAGCUCGGAGCGUCUGAAGAUCCAAAAAGAACAAGUCAGACUCCCCUGUUGGACUGCUCCUGGACCACCUGGAACAUGC

>random_seq_from_cds__NO_6415

CGAGCACCGCCAGGAUCUCGCGCUGCACGUCCCGGCGCUCGCGCGCGCCCAGACGUCGCUGGGGACAGCCGGGCGGGGGUCGCA

>random_seq_from_cds__NO_6437

AUGUUGACUAUAUAUCCUGUUGAAACGUGGCUUCCUCCAGUCCUGAUCGACGGAACUCUGCGAGGAUGGCUCUCAGGAAGCUCUGUUCCAGAACAGA

>random_seq_from_cds__NO_6444

UUUGGCCUGUUAGCUUCCUGCAAGAUUUGCACAUAGACUUGGUGGGGCUCCGUCAGCUUCAUGCCAUUGACCUCAAU

>random_seq_from_cds__NO_6451

CUUUUACUAUUACCUAGAAACCCGAAGCUGCUGCCUAAUCCGAUUCAUAGUCAAGACAGAACUCUUUCUGCGGAUACGAUGGGGAGUAGAGGUCGCUUCAUUCUGGGCUU

>random_seq_from_cds__NO_6454

UGAAGCCUUCUUGUCAUCCUCAGUAUAAGAGAGUCCAGUCUCUCUGGUUUUCUCUGGGGCUUUCAGAGCUGG

>random_seq_from_cds__NO_6457

GGAGGCGGAGUGCCUUGAUUCAAUCCUUUUGGCCACAUGUUCUGCUGGGGUCCAAGAAGGGCUCUCUGCACUCUUACUCUU

>random_seq_from_cds__NO_6458

GGCUCUCACGGGUUUCUGGCACUUGGCUGCACUCUCUUGUGGUUUAUUCAACUCCGCAAAUAGUUCUGAGGAAAGUGGCCUGAAUUU

>random_seq_from_cds__NO_6468

GGCCCGUUCCGAGGAAUUGUAAUAGCUCCCCAGGAAGACCAGGAUCCAGAAGCCGUUGCCCGCGGACAGCUCGCCCUUCUCCUUC

>random_seq_from_cds__NO_6469

CUGUUCACGUUCUCCCUGCACACCCCCAGGGCCCAGCUGGUGCGGUCCCCAACCUCCACCUCCCAGUAGUGGCGGCCUGAGGUGA

>random_seq_from_cds__NO_6477

GCUCCCCAGAGCGCUCGCAGGCCGCACACAGGAGGCGCAGCUCGUCGCCACAGAAGGCGGCCAGUGGCUCGCGGUGC

>random_seq_from_cds__NO_6495

CUGAGGAGCUCAUCUACCCAGACCGCAGGCUGGAGACCAAGUAUCCGGUGGUGUCCGAUGGCAAGAGGUUCCUGGGCUCUGGA

>random_seq_from_cds__NO_6500

GGCAUUGGGGGAUGAAGCUCUGCCCACGGUCCUGGUCGGCGUGUUCAUCGAACAGCCCACGCCGUUUGUGUCCCUGUUCUUCCAGC

>random_seq_from_cds__NO_6507

CCCUGCGGGGUGAGCUGCAGUCCUCAGAUCUCUUCCACCACAGCAAGCUGGACCCCGACAUGGCCUUCUGUG

>random_seq_from_cds__NO_6518

AGUGACCUGGAUAUUCUGUUGUUAGCCCAGCAGGAAUAUGGGCACCUCUUGCUGUAGCGACAACAGUAAAACUGCCACUCCCG

>random_seq_from_cds__NO_6519

AUCCAGCACUGACUCGAAGUAGCGGCUCUGGAAUCCUGCCACCAGCCCAUUGUUGGAGCACGUCUGGUACCCAUUCCAUGCCAGCCCUGUUGAUCUCCUCCCACCAGCAC

>random_seq_from_cds__NO_6520

UCCGUGGGUUCCCCGAGGCUCUGUGGCGUGGGCAUGCAGGCGUAGUUCCAUUGUCUGUCAGAACCUUCCUUCUUGCUGAAG

>random_seq_from_cds__NO_6531

UUUUAAUUGGUGACGGAGCUGGUUUCAAAGUUGCAAUGGGAGCUUUUGAUAAAACCAGACCUGUAGGU

>random_seq_from_cds__NO_6540

CUGCUGCACACGGAUGGUCCCUUGGCCAGCUCCUGGCGCCACUACAUUGCCAUCAUGGGCUGCCGCCCGCCAUCAGUGUUC

>random_seq_from_cds__NO_6541

UUACCUGGUAGGCUCCCACAUGGCCGAGUUUCUGCAGACUGGUGGUGACCCUGAGUGGCUGCUGGGCCUCCACCGGGCCCCCGAGAA

>random_seq_from_cds__NO_6543

CAGGGCCUUGCUGAAGACCGGCGAGCACACUUGGUCCCUGGCCGAGCUCAUUCAGGCUCUGGUCCUGCUCACCCA

>random_seq_from_cds__NO_6548

UCGGAUAUGAGGACUUCACUCGGAGAGGGGCUCAGGCACCCCCUACCUUCCGGGCCCAGGGAUUAUA

>random_seq_from_cds__NO_6549

CCUGGGAAGACCAUGGCUACUCGCUGAUCCAGCGGCUUUACCCUGAGGGUGGGCAGCUGCUGGAUGAGAAGUUCCAGGCAGCCUAUAGC

>random_seq_from_cds__NO_6557

CUUGACAGAUUCUGAUCUCCCGUAUGGCCGUGUGGUAUCUCUCCAGCUUCUCUCCCAACUUCACUGUGUUGUCUGGGAGCUUCAUGC

>random_seq_from_cds__NO_6558

UGGCACCUGCGAGUUAGGGUUGUUUCCGAGUUUUCUUUCUUGGGUUUCAUCCCGAAAGGAGAUGGUCCUGGGGCUGGAGCGUUUGAGGGAGCUGCUGUAGGUUCGCUGUG

>random_seq_from_cds__NO_6559

UGGGUGAGGACAUAUCUGCCUCUUCCUCCUUGCUGGGGAUUUUCACCCUGGAGUGUGACGGGUGGGAGGCGGGAGGCCACAGUCGGAGGGUCUGAAGCCCCCUGCUUUG

>random_seq_from_cds__NO_6568

CUGCCUCCGCUCCUGCCGUGUUCUGAGGAUGCUCUGGAUGUCCUCGUCCUCAUCUUUGGAGGCUGGGGGCAGUGGCUUGGGCACCUCUGC

>random_seq_from_cds__NO_6569

UUCUUCCACGCUCGGUAGUCCUCUCAGAAGCAGAGGCCUGCCGGUCUCCAUUCUGGCUGAGCCUGGGAGCCUCAUCGUCCGUGGUGGAGC

>random_seq_from_cds__NO_6571

CCUGCUGACAGCCAUCAUGGCGGCGGCAUUUGAAGCCUCGGGAGCCUUAGCAGCAGUGGCGACUGCUAUGCCGGCUGAGCAUGUGGCC

>random_seq_from_cds__NO_6576

GAUCUUGGCUCCUACAAGAGAAAUUGCUGUACAGAUACAUUCUGUUAUUACAGCCAUUGGAAUAAAAAUGGAAGGCUUAGAGUGUC

>random_seq_from_cds__NO_6595

UGGAUGAUCGUAUUUCUUUGGAACAACCACCAAAUGGAAGUGACACCCCCAAUCCAGAGAAAUAUCAAGAAUCACCUGGAA

>random_seq_from_cds__NO_6614

GCCAGAGCUGCGCGGUGAGGAAGACCAUCUCAGAUGGGGAUCCCCACAGUGUUCACCUGGUCCUUGA

>random_seq_from_cds__NO_6615

GUCCCAGCAGGCUGUAGGCGAUGCGCUUCUGGUGGCCGGGCAGCCGCACCCCAAUCCUCUUGAUGUCGCUCGUUGGUCAUCUGCACCACC

>random_seq_from_cds__NO_6619

GGCGGGCACGCUCCUGCUGCCAGCACUGCAUCAUGAGCUGGUAGAUGGCGGAGGGGCAGUCCAUGGGUGUGG

>random_seq_from_cds__NO_6629

AAUCACCGCCAAGUUGCCAGAUCCCUCCGGGGCACAGCGUCUGGAAUUCGUGCACCUUGCUGCCGG

>random_seq_from_cds__NO_6632

GAGACGCUAAGCGAGGUGGUGCUGCGGCCCUCCAGCCUCACCUUGGGGGGCUCCUGUCUGGUUGAUG

>random_seq_from_cds__NO_6642

CCUGGAAGGCCAGGUAGAAGCCUUUGCGGGUGAGCGGCCCCACGGAGCGCUCCUCCACGUUCAGCUUCACGUGGCGUGCCUCGA

>random_seq_from_cds__NO_6657

AACUCCACGUUUUCUGCUACCUGGAACCGAGGAGUCUCUUCUCCUUCAAGUCCGUGGUUCUGGUGGAGCCCGACU

>random_seq_from_cds__NO_6663

UGUCAAGAAUUUGGCCUGGGUGGACUCAGACCAGCUCCUCUAUCAGCAUUUUUGGUGUCUCCCAGUGAUCAAAAAGAGAGUGGUUG

>random_seq_from_cds__NO_6666

UGCAGCGUUCAGCCAGCUCCACCUGCGCCGUGUGGAAGCCGCGGCCACAGCACAGCAGCUCACAGCCGUCGA

>random_seq_from_cds__NO_6667

UGGCCUUGGACGUCUUGUUGCAUGUGCGGCCCCUCGUGCCCAGCACGCCGCUGCGCAUGUCCUGCUCACAGAAGUCGGGGCU

>random_seq_from_cds__NO_6683

CUUUACCUGGUGACUGUUCUCCCUUGGUCACUCGACGGGAAUCCUCCAGCUGAGUGAUGAUGGUCUGCACACGGUCAUUCCCCGCC

>random_seq_from_cds__NO_6687

AUGAUCACCUCGUGGCGGCAGGUGGGGCAGCGGAAACGGCCUCCAGACAUGGACACUGAGCUGCCCCGGCUGGUCCAGUAGGGAUU

>random_seq_from_cds__NO_6721

AGCACACGAUGUAUUUUGCCUGAUUUAGAUCUGGGGGCUGGUGGGGGCCAUAGCCUUGGCAGCUUGCUGGGCUAACCAGUACUUAUAUCUUUUGGUCUUGGGCUUCUCAA

>random_seq_from_cds__NO_6731

CCUCUCCAGCCUCCUGAGGACCCGGGGCUGCGUGAGCGAGCAGAGGCUUAAGGUCUUCAGCGGGGCGCUCCA

>random_seq_from_cds__NO_6734

UCCUACCGCAUUGGGGAGGACGGCUCCAUCUGCGUCUUGUACGAGGAGGCCCCACUGGCCGCCUCCUGUGGGCUCCUC

>random_seq_from_cds__NO_6751

AAGAAGUUCCAGCACAAGACUUGAGAAAACUCUGUGAGAGACUCAGGGGUAUGGAUUCUAGCACUCCC

>random_seq_from_cds__NO_6754

UGAUGAAGCUUAUGACCUGCUUGAUAAACUUCUAGAUCUAAAUCCAGCUUCAAGAAUAACAGCAGAAGAAGCUUUGUUGCAUC

>random_seq_from_cds__NO_6758

AUUUGGAUGGAGAGCUCUGUCUAGGGGCUGGGGUUGGAGCGUGAGUUGGUCGAUGGUGUGGUAGAAGUCACAGGGGUCAAAUUCAAC

>random_seq_from_cds__NO_6763

UGCUGAGUGGCAGGAAAGAGACCCGGCUGGCCCCAUGCGAUGGGCUGAGCCCCCGGCAUCACCUGAGCGACU

>random_seq_from_cds__NO_6768

UCACGGAUUGGCUCGUGUCCAGCCUCAAACACAAUGUACCUGGUACACAGGAUCUUCAACAUCCUCUUCCAAUAUUGUCCUGGUACACAGCUUGUUCACACUGCUUAUCC

>random_seq_from_cds__NO_6770

CAGCCCGCCUGGGCUGUUUUUAUGGCCACAAAUCUGUGAUUCCCUUCCUUCCCACAAACAUAUCCAAAGGCCCGGUGAUCUGUAAUG

>random_seq_from_cds__NO_6774

AAAGUGGCUUCACUGCGAUCCUGACCCUUUCUUUCUGGAGUCUUUCUUGGCGCUGGUUUUCACAGCUACUUG

>random_seq_from_cds__NO_6789

UACCGGGUGGACCAUUACUUAGGCAAGCAGGGCUGUGGCGCAGAUCCUGCCUUUCCGAGACCAGAACCGCAA

>random_seq_from_cds__NO_6790

GGCUUUGGACGGCCUCUGGAACCGGCACCAUGUGGAGCGGGUGGAGAUCAUCAUGAAAGAGACCGUGG

>random_seq_from_cds__NO_6793

AGUCUUACAGUGAGCAGGUGCGCAGAGAGCUGCAGAAGCCAGACAGCUUCCACAGCCUGACGCCGACCUUCGCAGGUGGUCCUAG

>random_seq_from_cds__NO_6806

CCCACAGUCACCCACUGGCCUGGAUGGCGAGCAGCUGGUCGUGCUGACCACGAGCCCCUCCCAGCCACACCGCCGCAUGAGCCUUAG

>random_seq_from_cds__NO_6819

ACGGGGAAUGACAUGCCAAGCUCGGAGCUCCUACCUGGUAGACGAGGUGCUGUGGGGCCACC

>random_seq_from_cds__NO_6822

GGUGGAGGAGGAGGGGGCGGGGGAGGGGGCGGGUGGGGAAGCUGGGGCUGACAAGGAGCAGAAUGGCUGCCUGCCACCCCCAGAGAGUGAGU

>random_seq_from_cds__NO_6825

CCACCACUGCCUCCACUACCGCUGCCACCCCUGAAGAAGAGAGGGAACCACAGCACAGGCCUGUGUCUCCUUGUGAUGUUUUUCAUGG

>random_seq_from_cds__NO_6830

AACAACCUGCCCCUGAGCCACAAGGUCUACAUGAGGAACUCUAAGUAUCCCCAGGAUCUGGUGAUGAUGGAGGGGAAGAUG

>random_seq_from_cds__NO_6831

AUGAGCUACUGCACUACUGGGCAGAUGUGGGCCCGCAGCAGCUACCUGGGGGCAGUGUUCAAUCU

>random_seq_from_cds__NO_6832

UACCAGUGCUGAUCAUUUAUAUGUCAACGUAUCUGAGCUCUCUCUGGUCAAUUUUGAGGAAUCUCAGACGUUUUUCGGCUUAUAUAAGCUCUAACUCA

>random_seq_from_cds__NO_6835

AAGAGGGGCUCCUCAUCCUUCUGGUAAUGAAGGCUAAUGUUGACUUCCUGGGAGAAGUAGCCCUUCAGGGAGAUGAGAU

>random_seq_from_cds__NO_6861

UAGAUGAGGAGGAAGGAUGGGUGGGGAUUUGAAUCUUUCCUCAGAUCGAAACGCAUACAUUUCUUGACCAUAAGGCCCU

>random_seq_from_cds__NO_6874

CAGGCUGCAGCUGGACAGAGUGGCAGAGGCGGCUGAGGCUUGGGCAGCCAGGUCCCGGUAAGCAGGCGACUUCAGGAAGCGU

>random_seq_from_cds__NO_6880

AUUUGUGAAGAAAGAUCCCCAGACGUGUCUUGAACUCUUUGGCUCCUCUCCAGGCAGGUGGUGGGGAAGGCGGCCAGGGUGCGG

>random_seq_from_cds__NO_6884

AGGAUGCUGUGGGCUCCAUGAUGCUAGGGUCGGUGCUGCUGCUGGGCUGGGCAGAGUCAGGGCUGGGGCAGAGGGUGGAGGUAG

>random_seq_from_cds__NO_6886

GUUGUCCCUCAGUCCUGUCGGGCACUUCCAGUCUCGUCUGCGGAGGGGCCUUGACGGCAAUC

>random_seq_from_cds__NO_6887

ACUGUCUGCUCCUUAAAGUUGCCAACAGCACGGAUAUCCUGGUAAGUCACAUAGGCCAGCUCUCUUGUUGGCCUUGUCCUCAGUCAGGUGCUUGAAGCUCAGAGAGCAGC

>random_seq_from_cds__NO_6893

AUCCAGCUUCCUUUUGGCCCGGCAGCCGGCCUGCCGGCAGGCAUCGCACAACUUGGCCCUCGGGUCCGUGGGGAGUGGCGU

>random_seq_from_cds__NO_6894

CGAGGCAGGUGCCUGGCGCCGCUGCGGGAGGCGCCGUCUGCGGGUACAGCGGUGUGUAGUAGGUAGCAGUAGCUGGGCAGA

>random_seq_from_cds__NO_6898

CCUUGCAUUUGAUAUGUCUGUGUUCCAUCCUGGGAGAGUCUUAUAUUGAACUUCAACUUUAUUUAAGACUUCUUGGUUUGCCUGGGAUAUGAGGUAUGAUUUCACCAUCU

>random_seq_from_cds__NO_6919

GCAUGACCAUCGCUGCCUACCAGACCCUGUACGAGAGCAGCGUGGCGUUUGGCAUGAGGAAGG

>random_seq_from_cds__NO_6922

GAAGCACAAUGAGGAGAUUCAGUUCCUGAAGCGAACAAAUCAGCAGCUGAAGGGCCCAACUGGAAGGCAUUAU

>random_seq_from_cds__NO_6923

UGCACCAAAGAAGUGAAAUGUCCCUGCUCUUCUCUCGAUGCAACUCUAUCGUCACAGUCAAGAAAAAUAAGAGACACAUGGCUGAGGUGAAUGCAUCCC

>random_seq_from_cds__NO_6932

UGCAUCUGCCUCAGAGCCCGAGUACAUGGAGGAGGGUGCGGCGGCAGCACAUGGAGCGUUGUACCAGCUUCCUGGUGGAUGAGCUGGGCGUGGUGGA

>random_seq_from_cds__NO_6937

CUGGAGCUCUUGGCUCAAGACUAUAAGCUGCGAAUUAAGCAGAUUACGGAGGAAGUGGAGAGGCAGGGUGUC

>random_seq_from_cds__NO_6940

UCCACGGCCAUCACCAACUCCCUGCAGACCAUGCAGCAGGACAUGAUAGGAUGGCUUGAAACCCCUCC

>random_seq_from_cds__NO_6955

GUGAAAAUGAUGUUAAGUGGUGUGUGCUUGUUUGCUGUUUUCAUAGCUUUGCUGCCCAUCCUUGGACAUCGAGACUAU

>random_seq_from_cds__NO_6958

AAGAGUUAAAUUUAAAAGUCAGCAGCACAGACAAGGCAGAUCUCAUCAUUUGGAAAUGGUAAUCCAGCUCCUGGCGAUAAUGUGUGUCUCCUGUAUUUGUUGGAGCCCAU

>random_seq_from_cds__NO_6969

CCCGCACGUGUACCGUUUCUCCUCAUAAGAGGUAUCCGUGAACUCCAGGAGCAGGCGGAUGGCGUGCGCCAGCCCCAC

>random_seq_from_cds__NO_6979

AAAUUAAUGACAUAUUUCAAAAACUCAACAUAUUUGAUCAGUCUUUUUAUGAUCUAUCGCUGCAAACCAGUGAAAUCAAAGAAGAAGAAAAGGAACUGAGAAGAACUACA

>random_seq_from_cds__NO_6985

GGCAUUCCUGCUGAAUGUACCACCAUUUAUAACAGAGGUGAACAUACAAGUGGCAUGUAUGCCAUCAGACCCAGCAACUCUCAAGUUUU

>random_seq_from_cds__NO_6989

UACUGGCAAUGUCCCCAAUGCAAUCCCGGAAAACAAAGAUUUGGUGUUUUCUACUUGGGAUCACAAAGCAAAAGGACACUUCAACU

>random_seq_from_cds__NO_7016

CAGCAGAGUUAGGGGUGCCUCCCUCCAGGGGCUGGAGGUGGAGUUCAGCCCCCCGAUAUUGU

>random_seq_from_cds__NO_7025

CACCACCCACUACUACCUGGUCAUGCAGCUGUGUUUCUGGUGGGGAGCUCUUUGACCGGAUCCUGGAGCGGGG

>random_seq_from_cds__NO_7026

UGUCUACACAGAGAAGGAUGCCAGUCUGGUGAUCCAGCAGGUCUUGUCGGCAGUGAAAUACCUACAUGAGAAUGGCAUCGUCCACAGAGACUUA

>random_seq_from_cds__NO_7035

CUGACCCAAUUACCCUGCCAGCAUGGCCGCCGGCCCACUGCCCCUGGUGGCAGGUCCCUCAACUGCCUGGUCAAUGGCUCCCUCCACAUCAGCAGCAGCCUGGUGCCCAU

>random_seq_from_cds__NO_7039

GAUCCUGUCUGGCUCGCUUAGGAUUCUGCAGGUCACCAGGUGGUGAGGCCUUGCUUGCUGAGAUGCUCCGGAGAGAAUG

>random_seq_from_cds__NO_7049

GUCAGGGACUUAUCAAUGGCCAGAAUGUCACAGGCCCUUGCUCCUCAGGUCUUCGAGGCAGGAGGCGGCCACGCCGUGCUGUUCGCGGCUGUAGUUGCGGCG

>random_seq_from_cds__NO_7051

GCUGUGGCUGAAGAGGCUCUGGGUGCUCUUGGAGGUGGCUGUGCAUGUGGUCGUGGGUAAAGUGCUUCUGAUAUUGUUUCCAGACAGAGUCAAGCG

>random_seq_from_cds__NO_7053

CCCAGUAUUUCUGGUUCGUCUUGAAGGUCCGUUGGCAGCGACUAGAGGACACGACUGAGCUAGGGGGUCUGGCCCCAAA

>random_seq_from_cds__NO_7066

UCCCGCUGCUCCUCAACCCUGCGCAGCUGAAAACCCUUGACGGAUGGCUGAAAGCAGGUCGCUACGGGCAUC

>random_seq_from_cds__NO_7072

AUCUUUUGGGUCCAGCCAAACCAGAUCCUCUUUGGUUGUCCACUGGGUAACCUGAAUUCUGCUGGUGGAGGAG

>random_seq_from_cds__NO_7076

CUAUGCUUUCUCUUCUCUUUCAUGAUAUCCUUGGUGUCCUGCAGCAUCUUCUCCUUCCAAAGAUCAAAGAAGUAUGAAGGGUCUGUGUAGAAUUUGAGUGCCUCUUUUCC

>random_seq_from_cds__NO_7079

CCUUCUUCUUCCUUGGGAUCCAGCUGAGUGACUUUAACCUGUAGUCGGUCGACCCUCUCAGCAAGGGAGCUUACCCGAGAGGCAAAG

>random_seq_from_cds__NO_7092

GUGGAAGGUAGUGACCAGCACAGCCAGCGCCUGCUCCAGAGAACUGCACAUCAUGCGGCUGGCGCUGCUCUGGGCCCUGGGGCUCCUGGG

>random_seq_from_cds__NO_7093

CGCGGGCAGCCCUCUGCCUUCCUGGCCGCUCCCAAAUAUAGGGUGGCACUGAGGAGCAGCAGGCAGAGUCAGA

>random_seq_from_cds__NO_7098

GGUCCUCCCAUUAUUUCGCGAAUCCAAGAUCUCCACCUGCCAGGCCACACCUGUGCCCUGAGCUGGCGGGAAUCUGUACACACUCAGAAGCCACCAGAGC

>random_seq_from_cds__NO_7101

AAUGUACGAGUGGCACUAGUGGGCCUGGAGGCCUGGACCCAGCGUGACCUGGUGGAGAUCAGCCCAAACCCAGCUGUCACCCUCGAAAACUUCC

>random_seq_from_cds__NO_7114

AUAGACCAUCGAUGCCAGCGUGUGGAUCUCCUGGGGGCACAGGAAUGUCGAAGCAAAUGCCAUGGACAUGGGGGUCUGUGACAG

>random_seq_from_cds__NO_7120

AGUGUCCUCGCUCUACCUCUGAGAUGGCGGACGGGGAGCUGAACGUGGACAGCCUCAUCACCCGGCUGCUGGAGGGUACGAGGAUGUCGUCCAGGAAA

>random_seq_from_cds__NO_7150

GCAGUCUCUUGGUACUUUUGAUGCACAGCGUUUAUGGCAGUUGAAUUUGCAAUCCUUUACACUGCAUUCCUUGGCGAAAGAGGCCUUUC

>random_seq_from_cds__NO_7153

GUCUUGGAACUGAGAGGCCGGGUCCUGGUAAAGAUACAUUUGACAGACGUCUCUUUCUUACUCCACUACAGUUAUUUGG

>random_seq_from_cds__NO_7179

GUGGAGAAGUGGGAGCACGCAGUCAUGGUAAACCAGGAGAAACGUGGACUUCAGGAACUCUUUGUCUC

>random_seq_from_cds__NO_7203

AGCAUGUUAGCCUAGGUUUGUUUGGGGCAAGCUGUCGACUUGGCCGUCUAAUAGACUGGUUUUGCUGUAUCUU

>random_seq_from_cds__NO_7254

GAACCGCCGUUCUUCUGACACUGGCUCCAAGGCACCCCCCACCUUCAAGGGUCUCACUUAUGGACCAGUCAGUAAGAGAAGG

>random_seq_from_cds__NO_7256

CCAGCGGCGCUUUGCGGAGGAGGCUGAGGGUGGGCUGUGCCGGCUGCGGAUCCUGGCUGCAGAGCGUGGCG

>random_seq_from_cds__NO_7257

AUGCUGGUUUCUACACUUGCAAAGCGGUCAAUGAGUAUGGUGCUCGGCAGUGCGAGGCCCGCUU

>random_seq_from_cds__NO_7270

CAGCUCAUCUGCAGUCACAUUUAGUGAAGAAACUAUAUGGUCUAUACUGCUGGCUCAUCAGGUGACUGG

>random_seq_from_cds__NO_7280

UCGUUCUUGGUUACCAUGUAUAUUUUUUGCUGGAGGCAUAACAAUGGGAAACAUUGGUCGACAACUGGCAAUGGUACGAAUGUAAAGUU

>random_seq_from_cds__NO_7292

CAUGGGCAGCUAUCGAUGUACCUGCAAAAUAGGAUUUGGGCCGGAUCCUACCUUUUCAAGUUGUGUUCGCUGAUCCCCCUGUGAUCUCG

>random_seq_from_cds__NO_7293

GAAGAGAAAGGGCCCUGUUACCGACUUGUCAGUUCUGGAAGACAGUGUAUGCACCCUCUGUCUGUUCACCUCACCAAG

>random_seq_from_cds__NO_7295

CGGUUUCUGGCGUUCAUAGACGCAGGCCAAUCCAUCACCAUGUAGGUAAAGGACCUGUAUUUGUCAAGCCAAAGAACAC

>random_seq_from_cds__NO_7301

GUUCAGUGAACAACAGAGGAAAUGUGUGGGAUAUUGAUGAGUGUACUCAGGUCCAACACCUCUGCUCCCAGGGCCGCUGUGA

>random_seq_from_cds__NO_7309

AUCUCUGUGCUCAUGGGCAGUGCAGGAACACUGAGGGCUCUUUUCAAUGUGUGUGUGACCAGGGUUACAG

>random_seq_from_cds__NO_7314

GCUGGCUCCUUCCGCUGCCUCUGUUAUCAGGGCUUUCAAGCCCCACAGGAUGGGCAAGGGUGUGUGGGAUGUGAAUGAAUGUGAACUG

>random_seq_from_cds__NO_7321

GAUGAAUGUCAAGACCCCAGUAGUUGUAUUGAUGGCCAGUGUGUUAAUACAGAGGGCUCUUACAACUGCUUCUGUACUCACCCCAUG

>random_seq_from_cds__NO_7326

GGUUUUCUAAAUAGCUUUGAGGAGUUACAGGCUGAGGAAUGCGGCAUCCUCAAUGGAUGUGAAAAUGGUCGCUG

>random_seq_from_cds__NO_7336

UUGGUAACCACUUCCUCUGCCAUCAAAGCUAGCUACUAUAAUGUUUUCUGUGCUUGCAAGGUAAGUGGCCCAG

>random_seq_from_cds__NO_7354

UUAUUUUCUUGUUUGUAGAGAUAUUCAUGAUCCUGAAAUCCAUCUUAAGGAGUAUAACUUCAGUCUAUAAGUAUUUUUUAAGUAAUCAGUUAGAGUGUAAGUUUUGCGA

>random_seq_from_cds__NO_7364

GGACCUGUAGUAGCGAUGCAGCACCAGGAUGAAGUCAGUGAUGGUCAGCAUCCCCCACAAAGCUCUGCUUCUUGCUGUCCCAUAG

>random_seq_from_cds__NO_7382

UGAUCCAAAAGAAGAUCACAAUUACAGUGCAAGUAGCAUGGCAGCACAGCGUUGUGCAUCCAGGUCUAGCGUGUCUUCCCUGUCUUCUGU

>random_seq_from_cds__NO_7407

GGCAUCUGUUUCUGCAGUUUGAGGUGUUAAGGCACGUGCCCUGGAACUCCAGCCCGUAGAUCACUGGUGCAUCGUCCUCCAUGAU

>random_seq_from_cds__NO_7412

GACAGCACUUUUCAAAAACCUGGAUUCAGAAUAUGAACAACAUAAUCUCCUUCCCUUUGGACAGUUUGCUGA

>random_seq_from_cds__NO_7439

GAAGCUCUGGGUCCUCUGUCCAAUGCUAUGGUCCUGCAGCCCCCUGCACCCAUGCCUAGGAAGUCGCAGGCA

>random_seq_from_cds__NO_7442

GCUGACUGAGAUGGACCUCGAGGAGGCGGGAAGAGUUUAAAGAACGCUGUACUCAGUGUGCUGCUGUCUCAUGGGGUCUUACUGAUGAAGGCAAAU

>random_seq_from_cds__NO_7453

UAGCUUAACUUGCCACGUGGUAAAAAUGACUGGAAUGGGAGAAGUGGAUUUUCUGACAUUUGAUCCUAUAGCUAAAAUGGCA

>random_seq_from_cds__NO_7461

UCUCUUCUCCUUCCUGCUCAGAAUAAAGACUUCCCUUCUCCAUGAAGAAGUGAGCUUAGUUGAGAAGAAAC

>random_seq_from_cds__NO_7465

GAGAGAAAACCCCCGCCGCUUUGUCAUCUUCCCCAUCGAGUACCAUGAUAUCUGGCAGAUGUAUAAGAAGGCAGAGGCUUCCUUUUG

>random_seq_from_cds__NO_7476

AGAGGAUGGGAGUGAUGUCAAGUCCAACAGAGAAUUCUUUUACCUUGGAUGCUGACUUCUAAUCUACACAUU

>random_seq_from_cds__NO_7490

AAUCCUGUCCCGGUAGCAGCAAGGGUCUUCACGAUGGCUUUGCUAUCAUUACAUUUGACUGCAUAAAAGGGGGUGACAC

>random_seq_from_cds__NO_7503

AGGCUGCGGGCACGCAGGGGCUGGCACACGGCUAGGCAGCGCUCGGCGCUCAGGCCUGCCACGCUCAGCACCGUGGCGUAGGCGC

>random_seq_from_cds__NO_7511

UGCCAGCUGAAAAGGGGCUCUGUGGACAGGAUGAGAGGCCUGGACCCCAGCACGCCAGCCCAAGUGAUCGCUCCCAG

>random_seq_from_cds__NO_7520

CAGCAAACAAGACGGAUUCUCCUUCCAGGAAAAGAGGAUAAACGAAGCCGACAUCUUGGUGCUGACGGCGUCU

>random_seq_from_cds__NO_7534

UUGAAAAGAAGCCAGAAAAGUUUAAAGUCCAGUGUUUGACAGACAUCAAAAACCUGUUUUUCCCCAACACAGAACCCUUUUAUGCUGCUUUUGGAAACC

>random_seq_from_cds__NO_7547

GGCCAUCAUCUUGAGGGACGCCAUGUCUGCCCGGUCCACGCCCACAGCAUAGAGCUCAAUACCAGAUGCUUGGGCCCGAGC

>random_seq_from_cds__NO_7548

CGCCACCUCAUUCACCUGGUCCUGGGGCCUCCCAUCUGUAACAAUGAUGGCCACCUUAGGGAUGUUAGAAGAGGGCUCUCG

>random_seq_from_cds__NO_7549

AGCCCCUGCCUCCACUGUGAAGGCUUCGUCCAUUGCUGUCUGGAUGGCUAGGCCUGACAUGGUGCCUGUUGACAAGGGUGUG

>random_seq_from_cds__NO_7553

GGCGGCGUCCAGGGCUGCCCCCGGGACCUCGGGUCUCCAGCCUCCGGAAGCCCGGGCGGGCCACGGGGUCGGGGGCGGCGGAGGGCAGCAGCAGCAGCG

>random_seq_from_cds__NO_7554

GCCAGAGCAGCAGGAGGAGUCCCGGGAGGCGGCGCGCGGGGGCCGGGCGCGGCAUAUUAGAGGAUGAUAGUAAGUUCUCCUGGAGCAAG

>random_seq_from_cds__NO_7560

GGCACUGACAAUAUAUUCAGAAUGGAAGUCCUUAAGAGCAACUAACAGGUUCUUGAUCAGACUGACUAUCUUUUCUUCAAGUUCAUAAUAUUUCACUGUCCAGCCAACUA

>random_seq_from_cds__NO_7568

AAUUCUUGAGUAACUCGUACCAAGCCAUCAAACACGUUAUCCUUGAGUCCCUGGAAACUGGCUUGGCCUUCCUGAGUCAACAGUUCCUG

>random_seq_from_cds__NO_7570

AAACCCACUCAGCAUUGUUCUGCAGAUUUCUUCUCAGCUUUGAAGACACUUCUCUCAGGGUGAGCCCUGUGUGUUCCC

>random_seq_from_cds__NO_7571

AGUGGUACUUGUUGACAUAAUCAUAAAGGACCCCUGUGGCCUUGGGCACGUUGUCUUUCAGAGAGGUUAGCAAGCCAGA

>random_seq_from_cds__NO_7574

CGUCAUCUUCAUCCAUAUCCAUGCCCACGGUGCCUACGGCUGGGGAGGCUGCUGAGGUGGAGAUGCCUUUCUUGUCUUUC

>random_seq_from_cds__NO_7576

UUGCCAUCUUCUUCAUAUUCUGCACUGAAGUCACGGUGUGCAAAUGUUCCUUUAGUCUUAGAGGCUAACGUACCAUCUUCGAUUUUGUGUGU

>random_seq_from_cds__NO_7586

AAGCUCUUGUCAUAGACUGGUAGGAUGAUAUUUUUGAGGAACCUUAGGUGUCCUUCUAAGGAUCCUGCAAUGUCAAGGUGUGCCUUUUCUUGGUCAUUGGAA

>random_seq_from_cds__NO_7592

CUGAAUAUUCCCGAGAAAGAACCGAACCCUUGACAUCUCCUUUGGUAGAUGACUCAAUGGAAAAGUAAGAGGUGAGGCUUUCCAAGCUAAGCUU

>random_seq_from_cds__NO_7595

UUUUUCGUGGUUAAGCUCACAGUACUGUUAUGACUACCCUCCACAAAUUUGUUGCUCAGAGACAGAGCUGUGGCUAACUUCAAUCCCCUUUUUCUUGUCAAUCUUGUGGU

>random_seq_from_cds__NO_7612

CUGAGAAUCUGGGGCAGGCCCAUUUCCAUGACCCUUUUCCAGAAGAAGUCCAUGCUAUGUGGCCAGCUUUCAACAGUGUCUUGAUCU

>random_seq_from_cds__NO_7613

CGUUGCGCAGGUCAGCCUGACUAGAGAAGUCCAGUUUGGGGAUGUUCAAUUUGUGGAAGUAUUUAGUGUUGCUAUCCAGGGUAA

>random_seq_from_cds__NO_7627

GCCAAAGAUGCUGAACUUAAAGCCUCCUGUAACCAAUUGAUGAUUAAGGUUAUUUUGGUGUCCUGUAGGCUUUCCAGAUACACU

>random_seq_from_cds__NO_7638

CAUUAAAGUUGAUUUUGGCAUCAUCUAAUGCAAUUUGUAUAUCAUUUUCUGUAAUUCUAUACUUUUUUGUGAGAGCAGUCAGUUUCUCCUUG

>random_seq_from_cds__NO_7641

UUGCAAGGUCUCAAAAAAUGGGAGGUUAAUGGAGUGAACAUCUUGGUUUUUAUCAUACUUUACAAAAGCAACAAUUGUAAAUUCUUGGGGCUUCUCAACG

>random_seq_from_cds__NO_7644

GUUGUUAAAUUGGGUCUUGAGUUUCCAGGUGCCUGUCUGCUCAGCUGGAGUAAGCAGGGCACUGACUUUGUGUUC

>random_seq_from_cds__NO_7673

UCCAUUUGGAGAAGCAGUUUGGCAGGCGACCAGUGGGCGAGGAUCUCACUUCUGGCUUCUGCUUGCAAACGGGGUAUGGAAA

>random_seq_from_cds__NO_7677

UGCUUCGCACCCUUCUGCUUGAGUUACAAACUUCAGGGUAUCCACCAAGGCUCUGUCCUCUCUCUGGAGCUCAUAGGUUGCGCUGACAGA

>random_seq_from_cds__NO_7683

CUUGGCUCCGGGAGCAAUGACUCCAGAUGAAGAUAUUUGCAACUGUAAUCCAGCUCCAGUGGGGAGUUCAAAGGC

>random_seq_from_cds__NO_7690

UAUUUGGAUCAAAUAUAAGAUUCCCUUCUAUUUUGGCUGAGGCUGGGUCAAGUGAUGGAAGAGAAACAGAUUUGUAGAGUUGA

>random_seq_from_cds__NO_7694

UCUGAAGAAGAACCUCCUGGUCCCUUGUCUUUAGGCUCCAUUUUCCGCAGAGCCUGGAUGGCAGCUUUCUGGAUCAUCAGU

>random_seq_from_cds__NO_7695

GAUGGCUUUGUACUUUGGACACAUUUCAGGAUUGAAGACUUGAGUUCUGGAGUUAACUGCUCCAUGGUUUGGCCCAUAUUUCCAA

>random_seq_from_cds__NO_7709

CUGGCUGGUCUUCAGGAUGAAGCUGCAGAGCUGGGGAACCUCCAGCUCAACCCUUGCAGUUGAUCCUGGU

>random_seq_from_cds__NO_7711

GUGCUUGAAUCGGGUCGCAUCUUCUUGGACAGACCAGGCUGACAUUUUCCAGCAUUUCCUCUUCCGGCCCUGGCGCCCGC

>random_seq_from_cds__NO_7713

UGACAGAGGGGCCAUUGGGGAAGGUGGCUAGCUUAUCCCGCCCCUUCAAGAAGAAGGUCAGCAGCUC

>random_seq_from_cds__NO_7719

GAUUUCAUUGAGGAAACGCAGACACUCAAUACCACCAUUGUUGAUGCUCUCCUCUGUGUAGAAGUCA

>random_seq_from_cds__NO_7730

CCAGGGCCGUGCAGAGCAGGACGACGCAGGAGCAGCUGAAGGCAGCCCCACUCUGCUUCUCCUUCUCCAC

>random_seq_from_cds__NO_7733

GCGCAGCCUCCGGCGUGGGUUGGGGAAUGAGGGGUUGUCGGCCCUGGGCAUCCUGCUCCUCGGGCUUCUCCGACGUGGACCC

>random_seq_from_cds__NO_7734

ACUGCUGUGGGCACUCCCGUUGGGCUCCUUGGUCUCAAUGAGGGCAGGGGAGCUGGACUUUGAGGAAGCUG

>random_seq_from_cds__NO_7742

GAACUGCUGCUGGUCCUUCUGGCUCUCGUCUUUCUUCAUGUCUUUCAGCAUCUCGUCAGCCACGUGCUUGGGCAGGAU

>random_seq_from_cds__NO_7744

GUAGUAGGACAUGAUGCCCACAGCGAUGGCGCACAGGUAGAGGAAGACGUUGGCCAGGAUCCUCCCGCAGCAGCUGCAUCCCCUUGAGC

>random_seq_from_cds__NO_7750

CAGCACCAGCAGGGUCUCGUGGCGCUGCCUUUUGAAGUAGGUCUGGUAGAGGUUCUCCAAGGACUCCGGCAC

>random_seq_from_cds__NO_7764

UGCACUUCCAGUCCAUGUGCUCGUGCCUGUGGUACAAGAGCCGCGAGAAGCUGCAGUACUCCAUCCCCAUG

>random_seq_from_cds__NO_7767

CGUGUGACAUGGGCCCGCGCCGCCGGAGCCGAAAGCCCGAGGCCCCGAGGAGGCGCAGCCCGAGCCCGACCCCGA

>random_seq_from_cds__NO_7839

UCGGAGGAAGGACUUGAGGUCUCCCCCCGCCAUGAGCUCCAGCAGGAUGAACCGGGGCAGGGAUUGCAGGCUCACCCCAAUGCA

>random_seq_from_cds__NO_7843

CAGCUUGUACUCAGGGCUCUGCAGCUCCAUCUGCAUGGCUUGCAGCUCCUGGUGCUUCCGGCGGUACACCAA

>random_seq_from_cds__NO_7845

GUGGCUCCGGGGUGGGUGACACCAAUGCAGGAGACGCCAUCCUCAGCCAGCACCGUCCCGUGGUCACAGAAGCAGAUGACCUUG

>random_seq_from_cds__NO_7864

AGUCCUGGUGGAAGUCACAGGCCUGCCCAAGCUGGAGGACUGUCCCAUUCCAACAAGUGAAGGAGCUCUGCA

>random_seq_from_cds__NO_7866

UCCACUGGAGAUGUAUUCCAGGGCCACUCGAAAUGGGUUGUCUGGACGCCCGAUUCUUCCCUGGAGCACUGUCCAACCCAUGCUU

>random_seq_from_cds__NO_7869

UGCUUGGAGUCAGCUGAGGUGUUGAGAAGGAGAAAGGAGCCCUCUGGGCAUCUCCUUAGAACGCUCUGCCCCAGGCCCAUC

>random_seq_from_cds__NO_7873

CCCGAAGAUCUGGAAGAGAAGGCGGGGCUGGGAGGCGCGAAUUGCCGCGGACAGCCUUCCCUCUCUGCCCACUUCCGACGCCUUCU

>random_seq_from_cds__NO_7881

AGGGUUUGCAGUUUUCUGGGACACCAGUGACACACCAGGGUGGUGAACUUGUCCACGCAGGCAUUGCGGAUCUUCUCCGGGGUG

>random_seq_from_cds__NO_7890

GAGCUCUACUGGCCACCCUGGACCAUUUUGGUAUGAAGGCCUUGGCCCAUCUCAGUCCCCCCGUGGGUCAGCAGCACA

>random_seq_from_cds__NO_7893

GAUACUGAGAGCUUGCUAGGCAUUCUUCCCAGCAUCUGGGCAAGGUGAAACCCUCAAGCUUCUGGUUGAAG

>random_seq_from_cds__NO_7901

UUGGAACAGCAAUGGUGCAGUGAGUCUCCAGGUAGAAGUGCUCUUGGCCACCGAUGUAUAUCUCCCCCUGACACAACAUUAUCUGCUUCGGAAAAC

>random_seq_from_cds__NO_7929

UUUUUUUCUCAUAAGGAAUGACUGUAGUCAAAUACUGUGCCGGGCAUAUGACUUCUCUCAUUCUUC

>random_seq_from_cds__NO_7932

GCACCAGCUCCUCAGAGUACUCCUUAAUGGCAGUCUCAAUUUCUUCCAGGAUUUCAUUUAACUCAGACACUGAGAGCCUUUUCACUCCU

>random_seq_from_cds__NO_7933

CUCUUCAUAACUGCCGGUACUAGACCUCUUGAGAGUUUGAAUUUCCUGGGAAAGCAUUGAAAGCCGAUCUGACUGUGUAGGGGUUUC

>random_seq_from_cds__NO_7934

AUCAUCUUCUGGGUCCGGUGAUUCCUGCAUCAUUUCUUCAAUUUCUUCAAUAACCCUGGUCUGCCGUGAAGAGGGGUUCAUCAUUAACACAGGAGACGAUGAUUGAGUG

>random_seq_from_cds__NO_7948

GGGGGGCGGCCCCGGGCCGAAGGCUUUCGCAGUGCCUCAAGAACUUGUCCAGGAUGAAGUUGCUGAAGUUGCGGUUGAGCUGCUCGAAUUCGCG

>random_seq_from_cds__NO_7951

GGCGCCGUCCGCGCUGCCGCGCACCAGCAGCGCCACCAGCUCGCGCGCCUCGCUCAGCACGUGGCCCUCGAGGACUUGGCGGCUGCGCGGCUGGC

>random_seq_from_cds__NO_7955

CCGCCUGGCCCACCGCCGCCGCGUUUCCGAUCAGUGGCCACGCAAACGGGCCCGGGGGCGCGGACCGGAGCUGCCGCCUC

>random_seq_from_cds__NO_7958

GCAGGCGGCGGCCAGGGUCGAGGCUGGGAGACUCAGAGCCGCUGAGGCUGCCGGAGCUCAGGGAGCCGCUUAGGUAGCUGUCGCGGUC

>random_seq_from_cds__NO_7963

GUCCUCGGCGCCCCCGGUGCCGUACAGCAGAGCGGCCGCAGCCGCGGCCGCCGCGGAGGCGCAGCAUGUCGGGGCGCCCG

>random_seq_from_cds__NO_7969

GAAGGGCCGGCACAGCUCGGUCUUGUAGCGCGUGGAGUUGAUCUGGGAGCCGCCGCCCCCCUUCUGCUGCU

>random_seq_from_cds__NO_7976

UGGGCAGGUUUUCUCAAUGAAUUGAAUUCCUUGAGUGAAGGCACACAUUGGAUUAGUUGUCACAGAAACAUUUGAGCUGCCCACA

>random_seq_from_cds__NO_7986

UUCAAAGUUUUAUGACAAAUUGCUGAUUUCUUGUAGGCAGAUUCUAUCAUCUGGACUCUCUUGGAGGUUUCUAUUUCCCGUUCCUUGCUUUGGGUAUC

>random_seq_from_cds__NO_7997

GGAGCUCUGGGAGCCUCUGUUUACUUGGAGACCCAUGGACCCUCCGGGGGUCAAAGAUGAGAGGUCACCCAUCAUGGCGCCCCCGCAGG

>random_seq_from_cds__NO_7998

UCCUCGCGUUCGGGCUUCUGCUUGCCGCGGCGACGGCGACUUUUGCCGCAGCUCAGGAAGGAAUGUGUCUGUGAAAAC

>random_seq_from_cds__NO_8009

AGGAGACGCUGCAGUUGGAGAGCGCGGCCGAGGUCGGCUUCGUGCGCUUCUUUCAGGGCAUGCCGGAGAAG

>random_seq_from_cds__NO_8034

GUCAGCAGAAGUGUCCAUUGUGGACUGCAUCUUAGCCCGAGUAGGGGCUGGUGACAGUCAAUUGAAAGGAGUCUCCACGUU

>random_seq_from_cds__NO_8038

UAACUAUGCUUUAUCAGGUGAAGAAAGGGUGUCUGUGAUCAAAGUUUUGGGAUUCAUGUUGCAGAGCUUGCUAAUUUCCCUAAGCAUGUAAUAGAGUGUGCUAAACAGAA

>random_seq_from_cds__NO_8049

GGUACACCGAGGGCUUCCAGCCCGCCAGGCUGUCGGCCUCCGAGAGCGCGGAGCCGCGGCGGAAGGUGGCGGGCAGCAGGCCGCUGCGGCGCAG

>random_seq_from_cds__NO_8051

AGCCCGUAGGCGAUGAGGAAGGCCUUCCCGCCCACCGUCGCGGGGGUGGUCAUGCCGAAACCCUAUGGU

>random_seq_from_cds__NO_8076

UGCCGGUUUGAGCUGAUGCGUUUCAAGACUUUGUAUAAUGGGGAUAAUCUUCCCUUUUCCUUGAAGUCUGUAGUGGUUGUCCAGGG

>random_seq_from_cds__NO_8079

UGUCUGGGGAGUUUACAGGAACUUGAAUCUGAACCUGUCAUUCAAGUCACUGUGGGGUCAGCA

>random_seq_from_cds__NO_8096

GAAGGUACCUGAAGAAGAAGCUGACAGUAUUUCAAAUGAGGAUUCAGCCACAAACAGUAGUGAUAAUGAAGACCCUCAAGUAAACAUUGUAGAAGAGGGACCCUUUAAAU

>random_seq_from_cds__NO_8115

AAAUUGUGGACAACUUUUGGGGUAAAGUCACAAAAGAAGGAAAAUUUGUUGAAAGUGCUGUGAUAACUCAAAUUUAUUGCCUCUGCUUUGUGAAUGGGAACCUGGAAUGC

>random_seq_from_cds__NO_8134

GCAUAAUCAGAAUGGCAUGUCUUAAUCGCAGCUUUUGGUCCAGGUGAAUAGCAUAGGUGAUGGUGUGCCAUCUUUCUAGAGU

>random_seq_from_cds__NO_8143

CCAUAGCUCGGCAGGGCCUGCAAUUUGGUGGAAGAAAUAUCCCAAGGUUUUCGGCCCUGUGGCCCCACGG

>random_seq_from_cds__NO_8150

CAGCGCAGGGCGCCGUCGGGCACGCAGUUGCAGGGCUCAGGGCAGAGCGCCUCGCGCAGCGCUCGUG

>random_seq_from_cds__NO_8151

GCAGCGGCGGCUGCAGCAGCAGCAGCAGCUUCAGCAGCUGCAGCGCCGAGAACCGCUGCUUCAUCAUGUC

>random_seq_from_cds__NO_8197

GAGAACUGCAUCCAACAGAGGGGGAACAAAAUUUUCAGCGACCCAUCUGUGGAUCAUUGGAUCGGCUCACCCAACCAGAUAUUAAC

>random_seq_from_cds__NO_8201

AUUAUACUAUCCCACACUUGAUUAGGGAGUAACAUGUACUUUUCUAUCAAGUGUUCUUGUACUGUUUGAUCUGUUUG

>random_seq_from_cds__NO_8237

UUCCCCUCCUGAAAGGAUGUCAUCGCAACAGAUAAAGAAGACGUUGCCUUCAAAGACCUGGAUGUGGCCAUUCUUGUGGGCUCCAUG

>random_seq_from_cds__NO_8247

AGCCAGCAACUGUAAAGGGAUCACGCUGAGAAUGCCCUGCAAGCAGUCCACUGAGUGGGGCACCUUGAUCGUUCUUUUUGUG

>random_seq_from_cds__NO_8258

GCCCCUUCAUGAUCUGCUGGAGUUCCAUCUGGAGUGUUUGCACAGCUCGUCCGGGGUGAUCUCCUGCAGUUCGUUUAAUUCGAUGGAUAGAAAGACG

>random_seq_from_cds__NO_8264

GUUUCAGAUUCGAAGUCAUAGCCUUUGCUUUCCCAAAAACUUUUUCAAGUCUUUGUAGUUGGUGAUGAUUCCAUUGUGAAUAACGAUAAA

>random_seq_from_cds__NO_8265

UUCCAUUAUUUUUAUCAGAGCGCUGGGGGUGGCUAUUGACAGGACUGGGUUCUCCAUGUGUUGCCCAACGGGUAUGAG

>random_seq_from_cds__NO_8293

CUCCGAGGCCUUUGCCACCUCCAACAUUCUGCGGCUGCUGGAGCAGGGCCGGCUGCUCUCUGUGCCCAGGGCCCCUAGCCUCC

>random_seq_from_cds__NO_8298

CCGAGAACACAUGCAGGCGGUCACCCGAAACUACAUCACCCACCCCCGUGUCAGCCUACAGGACUGUGUGCAGCGUGAACGGGCC

>random_seq_from_cds__NO_8300

UGCUUGAGGUGGCUGGCACCAAGGCGAUUGUUCAGGGUGUUUGAAGGGACAUCAGGGAUCGAUGCCAGGAAGACCACUUGCGAAUUU

>random_seq_from_cds__NO_8375

GCCCGAAGACCCUUCUACUUUAGUUACUGUAGAUGAAAUACAAGAUGACAGCAGUGAUUUGCAUUUAGUGACUUUGGAUGAAGUAACUG

>random_seq_from_cds__NO_8376

AAGAGGAUGAAGACUCUCUGGCGGAUUUUAACAACCUUAAAGAAGAGCUUAAUUUUGUUACUGUUGAUGAAGUUGGAGAGGAGGAAGAUGGAGAUAAUGAUUUAAAAGUU

>random_seq_from_cds__NO_8403

AGUCCCAGAGAGUUCACAGGCUUCCCAGACCCCUACACAGAGCUCAACACAGGCAAGGGGGGAAGGUGUGGCUUAUCGUGGCCGGCUUCUGCUCUCCCU

>random_seq_from_cds__NO_8409

ACCAGUACCUGUACCAGCUGCGCACCCAUCACCUGAGCCAAAUCACUGAGGCUGCCCUGGCCCUGAAGCUCGG

>random_seq_from_cds__NO_8414

AGUUUGCUGAGGGGAAGCUGUCUGUCUUUGCUGAAACCGUAUGAGAACGAGACUAAGUUGGCCCUUGUUGGGAACUGGGGCACAA

>random_seq_from_cds__NO_8425

GAACACCCUUAACCCCACCUGGGACCAGACGCUCAUCUUCUACGAGAUCGAGAUCUUUGGCGAGCCGGCCACAGUUGC

>random_seq_from_cds__NO_8435

AGCCCCUCAUCCCCAUCCAGGGAGGAAGAGUUCAUCGAUUGGUGGAGCAAAUUCUUUGCCUCCAUAGGGGAGAGGGAAAAGUGCGGCU

>random_seq_from_cds__NO_8436

CCUACCUGGAGAAGGAUUUUGACACCCUGAAGGGUCUAUGACACACAGCUGGAGAAUGUGGAGGCCUUUGAGGGCCUGUCUGACUUUUGUAACACCU

>random_seq_from_cds__NO_8438

UCCCAGAAGACCCAGCCAUCCCCAUGCCCCCAAGACAGUUCCACCAGCUGGCCGCCCAGGGACCCCAGGAGUGCUUGGUCCGUAU

>random_seq_from_cds__NO_8445

CUGGUCCCGGAGCACGUGGAGUCACGGCCCCUCUACAGCCCCCUGCAGCCAGACAUCGAGCAGGGGGAAGCUGCAGAUGUGGGUCGACCUAUUUCCGAAGGCCCUGGGGC

>random_seq_from_cds__NO_8473

AAUGUUGAGGAUGUCUCCUGACACUGUGCCAAAGGCUCCUAAACAUUUAAAAGCAGGGAGACACUUCUAAAGGAGGCAUAGCUAAA

>random_seq_from_cds__NO_8500

ACCUGAAGAGGCACAGAAAGUUUCACCUGUUCUUGGACCAGCUGACCAGAAGACUGGGACACCAACUCCAACCUCUGCUUCUUACUCA

>random_seq_from_cds__NO_8509

CUGUAACCUCCCCUUCCAGCUCAUUUGGAGAGAAGCCCAUUGUUAUCUACAAACAGGCCUUUCCAGAGGGUCA

>random_seq_from_cds__NO_8565

AAAUACAAGUGAACAUUUCAGAUUUCGAAGGACAUUCCAAUCCAGAGGGGACCCCAGUAUUUGCAGAUCGGAUUACCAGAGAAGAUGAAGACCCCACUUUCUGCUUUCUC

>random_seq_from_cds__NO_8582

UUCUUCAGAAUCCAAUCACACAUUCUCUCCAGGUCUCAGAAAGUACACAUGAUGAUAGCAGAGGGGAACGAAGUGUGAAGGAAUGGAGUGGUAGACAACAGCAGAGAAAU

>random_seq_from_cds__NO_8593

UGAGGACAGAAAGUUAAAAAAGAACAAGAAGAAUUCCCAUGAAGGGAGUUUCCUGGUUUGUUCCUGUGGAAAAUGUGGAG

>random_seq_from_cds__NO_8597

GAUAAAGCGCCUGAAGUUAAUAGUCCAGGAGAGGAAGCUGCAGAGCAUGUUACAGACCGAGCGGGAUGCACUAUUCAACAUUGACAGGGAACGGCAGGGCCACCAGAAUC

>random_seq_from_cds__NO_8609

AAUGAGAUGGCCACAGCAGCUUCCUCUUCCUCCCUGGAGAAGAGCUAUGAGCUGCCAGAUGGGCAGG

>random_seq_from_cds__NO_8651

UCCAGCCGUGCUACGCGGCCCGCCGCCCCGGCCGUCCCGUCGCCCGGGGCCUCCCCGAGCCGCCCUCUCGGC

>random_seq_from_cds__NO_8653

UGCAGAGUCAGCGCUUUGGGACCAAGGAGGUGGAGGAAGACCUGGGCCGUGCUCUACCCGGCCAGUCCCCACGGCGUAGCGCGGCUC

>random_seq_from_cds__NO_8663

CAUGCCCUUCCCAGGACUCCCUAUACUCAGACCCCUUGGACAGCACGUCUGCUCAGGCAGGAGAGGGAGUACAACGGAAGA

>random_seq_from_cds__NO_8666

CAACCCUGCCACUGAUGACUACGCUGUGCCACCCCCUCGGAGCACAAAGCCCCUCCUUGCUCCCAAGCCCCAGGGCCCAGCCUUCCCUGAACCUGGUAC

>random_seq_from_cds__NO_8668

GUAGGGACUGACAAGACUGGGGUCAAGUCAGAGGGCUCUACCUGAUCUAGUUUUUGAACUUGCAAACAA

>random_seq_from_cds__NO_8674

CAGGAACAUCAGGGAGGAGAUCAGCAUGAAGAACGAGUUGGUCUGAGCCAUCAUGGCUCAGACCAGCUCAUACUUCAUGCU

>random_seq_from_cds__NO_8679

AGCAGUGGGUCCCUGGUCUCCUACAAGUCCUGGGGCAUUGGAGCCCCAAGCAGUGUUAAUCCUGGCUACUGUG

>random_seq_from_cds__NO_8702

AGGCGUCGGACGGCAGCCUCCUGGGGGACCUCGGGCACACACCACUUAGCAAGAAGGAGGGUAUCAAGUGGCAGAGGCCGAGGCUCAGCCGCCAGGCUUUGAUGAGAUGC

>random_seq_from_cds__NO_8706

AACGCCUUUGAUGCGGACGGGAACGGGGCCAUCCACUUUGAGGGACUUUGUGGUUGGCCUCUCCAUCCUGCUGCGGGGCACAGUCCACGAGAAGCUC

>random_seq_from_cds__NO_8718

AGCACAUGAGCCGGGCUGUCGGGCCGGAGCUCCGCCACCGAGGCACUGCCCUCGUCCAGCACCACGGCCCGCGCCAGCUCC

>random_seq_from_cds__NO_8741

CUCUGGUCCAGCUUCACCUCAAACCAGGCACCAGGGUUACUUAAGAUGGCCCAGGGCCAUAGGGUAGAGACUGAGCAUUAUGAAGAA

>random_seq_from_cds__NO_8743

GUGAUGAUGAAGAUUUGGAUGACUUAUUUGUGGGACCUGUUGGGAACUCUGACCUCUCACCUUAUCCUUGCCAUCCACCUA

>random_seq_from_cds__NO_8746

UGAAGCAGAGCAUGUGGAGUCUGCUGACAGCGCUCUCCGGAAAGGAGGCAGAUGCAGAGGGCAAACCACAGGGAAGCUGGAA

>random_seq_from_cds__NO_8750

ACUUCGCGGAGCUGGAACGCUGGCCGCGCAGGCCCUGAGGGCUCGCGGCCCCAGUGGCGCGGCCGCGAUGCGCUCCAUGGCAUCUGGAGGGUGGU

>random_seq_from_cds__NO_8764

AGGAGCACAGGAUGCCUCCUCCGAGAAGGUCUGCAGCUGAUCCUUGGAGUAGACGUACUGCCAGAUGCGUUCCAUGUCGUUC

>random_seq_from_cds__NO_8767

UCUCCUGCAAAGCCAGCUUUAAUCACCCCCGAACCCGUUGUCGAUGACCACAGGCUGGUUGGCGAUGAUGUCGUAGGACUCCAUAUCAGUUGGUGG

>random_seq_from_cds__NO_8770

CUUCACUCCUUAAAAGGCAAAACUUCCACAGUUGUGUUUAGCAGAAUAUCUCCAGGAUGUUCUUGGUUGCCGCUAUGGAACAAAUAACCUUUCUACAUU

>random_seq_from_cds__NO_8775

UCAGAGUAAGAUCCGGCGCUUGAAACAUUUUACCCAAUGAAGCCCAGCUGGGAAAACUCUAGAAUCAUCCAU

>random_seq_from_cds__NO_8788

AGAAUUGUCUUUGGAAGCUCAAGGCAUCAAAGCUGACAGAGAAACUGUUGCAGUGAAGGCCAACAGAAAACAAUGAAGAGGAAU

>random_seq_from_cds__NO_8816

GAUGUCAUCUUGAUCAAUGGGCGUUUGAAGGAAGGAGAUACAAUCAUUGUUCCUGGAGUAGAAGGGCCCAUUGUAACUCAG

>random_seq_from_cds__NO_8818

GCUCAGGGGGUAAAGAUUCUUGGAAAAGACCUGGAGAAAACAUUGGCUGGUUUACCCCUCCUUGUGGCUUAUAAAGAAGAUGAAAUCCCUGUUCUUAAAGGAUGAAUUGA

>random_seq_from_cds__NO_8828

GGUGGGCGCUGUUCCGCAGCCAGUGCAGGCCCUGUUGGGAGUACUGGACCAGGUGCUCCAUGCUGCUGUG

>random_seq_from_cds__NO_8830

GUAGUCGUAGCUGUGCAGGAUGCUGUUGGUGAUGCUGACGUGGUUGGCCGCCAUCUGGUGGAUGCGCUGUGGGAUGCUGACGAUGGUCGA

>random_seq_from_cds__NO_8833

AAUAGUCGAUUAGUGCUUUUGAAUACUUUACAGCGUGGUCCCUUUUGAGUCGAAACAUCCGCCAGUACAGGAGGGCCAGGCAUCGGUAACCAUAAUGCAGCCAGUUGUUUGUCUUCUGG

>random_seq_from_cds__NO_8837

UCUGCCUCUUGCAGUCCCUGUGGCCGUUGGAGCCUGGAGACCACGGCUUCGUCUGCGGCCGUGACUUGUGGAGGGGAAUGUUUUCA

>random_seq_from_cds__NO_8840

UUUAUUGGUAUUGCCACACCUGUUGAUGUUCAUGUUGCAGUGGUUUGCAGACAAAGUAUUACUGGUGGAGGUGGCCAGUCUUGAAGAGCU

>random_seq_from_cds__NO_8842

UUGGAUUUUGGCAAAGCCUUUUCUGCAGGUGUGUCCGAGGUGUGGCUGGGCGGUGCGCUCUCAGAGUCCUUGGUG

>random_seq_from_cds__NO_8844

CCUGAUCUCAUCACUGUCCUUUAGAGGGGAGAGAAGUUCGUUCCGGCCAAAGGGGACCAGUGUGUAGAACUGCUCCUC

>random_seq_from_cds__NO_8848

ACGGAGGAGCGCAGCUCCUUGCGGUGGCUCGCUCUGUUGUUGCCACAGGGCCUGGUUUUGGUGGGCUCCGG

>random_seq_from_cds__NO_8850

GUCCUCUCGGUGCGCCUGGUGGGCUUCUUGCCCGCGGACCUCCGGGCAGGCGCGGGCGCGUUCUCCGCGGGCGCACAGGGCACUGCG

>random_seq_from_cds__NO_8872

UGGACAAAACCACCGUGUUGGAAAAGGUCAUCGGAUUUUUGCAGAAACACAAUGGAAGUCUCAGCGCAAACGGAAAUCUGUGACAUUCAGCAAGACUGGAAGCCUUCAUU

>random_seq_from_cds__NO_8874

UCAUCUAUGUCUCUGACAGUAUCACGCCUCUCCUUGGGCAUUUACCGGUCGGAUGUCAUGGAUCAGAAUUUGUUA

>random_seq_from_cds__NO_8883

UCCGGGUGGAAAGGAGGCAGGAGCUGGCUCUGGAAGACCCGCCAUCCGAGGCCCUCCACUCCUCAGCACUAA

>random_seq_from_cds__NO_8901

CUACAGAACAAGCCUCCAGGAUUCAUCAACACAAAGAGAAACUUUGGUUUGUUCCUGCUAAGGUGGAGGAUUCAGGACAUUACUAUUG

>random_seq_from_cds__NO_8929

GCCUUCCUUUCAUGUGCCACGCUGGAGUGUCCACAGCAUACAUUAUAUUACAGCUCCCAGGCUCCGGAUUUUCGAGCUUACUUGAUAGGAGG

>random_seq_from_cds__NO_8934

UCCCCGAAUCGCUGGGCUUUGGCCUGUUGAAGAACCUGUCAGAAGAACAAAUCGCGGUCUACAGUGCCCUGAUCCAGGACGGGAUGA

>random_seq_from_cds__NO_8946

AAUAGUGGAAGGAUCGCAGUAAUAUAGUUCCGGUUCUUCUUGGACCAAAGCUUAACCAUGUUGCAGUGGA

>random_seq_from_cds__NO_8951

UGGUGGCAGUAGUGUGCCUAGUGACUGUGUGUGUCAUUUAUAGAGUUGACUUGGUUCUAUUUUAUAGACAUUUAACGAGAAGA

>random_seq_from_cds__NO_8972

CUUUGUAAAAAUGAUGUUACUGAUGGAAGAGCAAAAUACUGGCUUGAAAGAGCAGCCAAACUUUUCCCAGGAAGUCCUGCAAUU

>random_seq_from_cds__NO_8976

UUUACAGUGUUUGGAGUCUGAUAAAAGUGACUGGCGAGCAACCAAUACAGACUUACUGCUGGCCUAUGCUAAUCUUAUGCUUCUUACGCUUUCCA

>random_seq_from_cds__NO_8982

CUUGGUAGCGAUGAUAUUGGAAACAUUGAUGUACGAGAACCAGAGCUUGAAGAUUUGACUAGAUACGAUGUUGGGUGCUAUUCGAG

>random_seq_from_cds__NO_8997

CCUCAUCGUUGGCCCACAGAGAAUUAUGGACCAGACUCAGUGCCUGAUGGAUAUCAGGGGUCACAGACAUUUCAUGGGGCUCCACUAAC

>random_seq_from_cds__NO_9026

AAGGAAGGACAGUGGGAUUGCAGUGUGUGCUUAGUAAGAAAUGAAGCCAGUGCUACCAAAUGUAUUGCUUGUCAGUGUCCAAGUAAACA

>random_seq_from_cds__NO_9046

AGAUGGACAGUACUUUGAACCUGUUGUUCCUUUACCUGAUCUAGUUGAAGUAUCCAGUGGUGAGGAAAAUGAACAAGUUGUUUUUAGU

>random_seq_from_cds__NO_9063

UAAAUCUGAAGAACCUGAUUCUAUUACCAAAUCCAUUAGUUCACCAUCUGUUUCCUCUGAAACUAUGGACAAACCUGUAGAUUUGUCAACUAGAAAGGAAAUUGAUACAG

>random_seq_from_cds__NO_9090

CUUUUCCAUCUUCUUCUUUGGGUAAUUUUUGGGAUCUACACUCCUCCAGCUGUAGAGUGGGCUUAUCAUCUUUCAACACGCAGGACAGGUACAGAUUCUUUUCCUUGAGG

>random_seq_from_cds__NO_9111

GCGCCCGGGUGCCCAACGGGUGGACAGGGUCUCUACCUGCGGCAGUCUGGCCUCUGUCACGCUCGACCCGGCCUCCUCCUCCUGCGCCUCCUCUGG

>random_seq_from_cds__NO_9113

UGAAAAAGUUGGUGGUGCGGUGCAGCUGGGCCGCUGGCUGCGGCUGGUGAGCAGGCGCCGCGAGAUGCUGAGGCG

>random_seq_from_cds__NO_9115

GACACCGGCACGCUGUCUCCAUCGCUGCCGCUGCCGCUGCUGCCGCUGGCGCCCGGACUGAGGCUCAGGCUGAGGCCGCCCGGAG

>random_seq_from_cds__NO_9117

GAUGAUGCUGGUCCUGCAGGUGCCCUGGCUCUGCUCCAAGUGGCUGGCCUUGGUGCUGUUGCUGAAGGAGGCCACGGGGUGCAGUGGGAACUCACGGAGGUGCCAUUGCU

>random_seq_from_cds__NO_9124

CCAGCAGCCAGGAGUAGUUGGCCAUGAUGCAGUACUGGAACAGCACCAUGACCAGCUUGCAGCCCGC

>random_seq_from_cds__NO_9126

GGACACGAACAGGUGCAUGUGGAUGUAGUUGCGAGUGCAGUGGAGCCUCCCGGAAAGCACAGAGGAUGCCAAGGGCGACCAGGAGCAUGACC

>random_seq_from_cds__NO_9160

GUACGAAACAAGAGGGCUGGCAAGGAAGAACCCGUUGUUGUCCAUUGCAGGUGCUGGAAUCGGAAGAACUGGGGUUCUUAUUACUAUGG

>random_seq_from_cds__NO_9161

AAACAGCCAUGUGUCUCAUUGAAUGCAAUCAGCCAGUUUAUCCACUAGAUAUUGUAAGAACAAUGAGAGAUCAGCGA

>random_seq_from_cds__NO_9170

AUGGACGGGCUGCCCGGUCGGGCGCUGGGGGCCGCCUGCCUUCUGCUGCUGGCGGCCGGCUGGCUGGGGCCUGAGGC

>random_seq_from_cds__NO_9200

UGUCAUUCCUCCUUUCAUGAAACUUGAUCAGAAACUGGCAAGCUCUAAAUUUGUUGGGGUUCAUGGUGUACAGCAAGAUUCGUUUC

>random_seq_from_cds__NO_9211

CAUUUGCUCAUAGAAGUCAAACAGGUCCAUGGGGAUGUCAGAUUUACCCUGUGGAUCUGUCACUCGGGAAGUGGAGGG

>random_seq_from_cds__NO_9212

CCCACCACUGCUUUCAGCAGUCUUAGAAAUCGGCAGAUUUGCUUGUGAAAGUCUCUGUGAUGAGCUCAGUGGCCUCCCCUU

>random_seq_from_cds__NO_9215

CUGAGCUUCCUGAGGUACUCGGUGAUGUCACUGGUUUGCAGCCCAACGCUGACAGCUGCAUACAAGGAGUAGGCAGUUAGU

>random_seq_from_cds__NO_9234

CCCCUCUGCACAUGCCGCCACUCGCUCAGGUAGCGGGACACGGGGUCUCGUAGCAGGGUGAUGUAGUAGAACUUCCCUGGGCGUG

>random_seq_from_cds__NO_9235

CGCAGCGCGGCGGAGUCGCGGCGGUCCAGCACGCCGGGCACGCAGUUGGUGAGCUCGGUCCAGUCGGCGUGCAGCCCGCAGCUCCAGCCGGUGGAGAAG

>random_seq_from_cds__NO_9237

GCCGAAGGUGGUGCCGCCCGUCUUCUGGAUGUGCAGGAAGACGAUCACGUCGUCGCCCUUCAUGUCGAAGCGCAGCGAGCGCUCCA

>random_seq_from_cds__NO_9248

GAAGAAUUCCGGUGGAUGAGACUACGGAUCCGGCGAAUGGCUGACGCAUGGAUCCAAGCAAUCAAGUCCCUGGCAGAAAAGCAGAAC

>random_seq_from_cds__NO_9263

AACAAGGACAAGGACAUGCUGAAGGUACAAGGUGACCUGCCAAAGCUCAGAGCUGGCCAAGGACAUCCUGGUGCCCUC

>random_seq_from_cds__NO_9270

UUACAACAUCCUGCUCUAUUCCUUGGUGCCACUUAUGUUAAUUGCGGGGAUUGUCAUUUGUGCAUUUUGGGUGUACAGGC

>random_seq_from_cds__NO_9272

GUUAUUAGAAGUGAAAGCAAGGGGAAGAUUUGGUUGUGUCUGGAAAGCCCAGUUGCUUAACGAAUAUGUGGCUGUCAAAAUAUUUC

>random_seq_from_cds__NO_9285

CAUCCAUUUGAAUGCCUUCCAUUCCUGUCAGAAGCACUGUCCUCUUAGAUGUUCCUGAAAAUAUCCUGAUACUUUUUCAAGUGUAUUU

>random_seq_from_cds__NO_9291

CGAGCUCACUUGAAACGAACAGGAGAUAUUUGACAACUGGCUGUCAUUCUCAGGAGUUUCAACUGAUAAGAAUUUCA

>random_seq_from_cds__NO_9296

CGCAGAAGCUAAGGCGGUGUGUGAAUUUGAAGGCGGCCAUCUCGCAACUUACAAGCAGCUAGAGGCAGCCAGAAAAAUUGGGAUUUCAUGUCUGUGCUGCUGGAUGGAUG

>random_seq_from_cds__NO_9311

AUACCUCGGUAGUUAAUGUCACUGAAGUCCUGCAUGUUCUUCUUUACUCGUUCAGUGAUAGGAUCUGUCACCACUGGUGUGA

>random_seq_from_cds__NO_9316

UGUAUAACACCCGAGCUAAUGUUUUCUUGAUUGCGUUUGACUCUCUGCAUCUCAGGAGUGACAGGGGUUGCGGUGGCUUUCCCCAC

>random_seq_from_cds__NO_9323

ACACGCAGUAUUUCUGGCGUGUCUUGAACAACUGUAAUUUUUCCUUUACUGUUUUUAAGGUCACACUGGUAU

>random_seq_from_cds__NO_9332

UCUUUGUAUUUCACCUAUACUCUGUAAUUCUGUGGCCUCUUUUAGGUGAAGCUGCUCCAGAUUAUCGGGUAUGGUGUGGUAGUGGCCUU

>random_seq_from_cds__NO_9333

UACUCUUCUCAUACUUCUUCUUGUACUGGUACAGAACUGGCAAGUUGGCUUGUAUUCAGGACAUGAUUCAUGAUCAGAGAC

>random_seq_from_cds__NO_9337

GGCCUGUUUGGCUGCCUGUGUGGCCUUCUUGAUGUCUGGUCGAUCAGCCACUGUGGUGUAAUGCAGGUUCUCUUUGGCAUCUU

>random_seq_from_cds__NO_9341

CAGGAUCUGAGGCGUGUCAGGUACGGCAUGGCAGGUUCCUCUUUCCUUGACAUGUUUCUCUUUGUAUUUCAGCAUCACUUUC

>random_seq_from_cds__NO_9342

UAUUAAAGUAUUCCUGAGGGCGAGCACCGUGUUUUUGUCAUCAGUGACAGAAAGCUUGCAACCCUUGAGGAACUCCCGGUCCAGCUUAUAUUCAAAC

>random_seq_from_cds__NO_9343

AUCGCUUUGCUGCAGGGAUGACUUGGCAGCCUGGAGGAAGUCCGGUCGGUCAGGAGUCCACUUCCAGUGGGCUUUGUUGG

>random_seq_from_cds__NO_9356

CCAUGUCCUUCACGUCUUUAGCAUGCUUCAAGGCUGUGGUCUGGUUUCCAGCGUGAUGAUGGGGUCUCUCUUUGGUGGC

>random_seq_from_cds__NO_9357

AAGUUCAACAUACAGAUACCUGACUCUGAAGCUUCUGCCCUCGCUUGGCUCUUAAAAGAUCAGGAGUAUCAGGAACUGAAGUAA

>random_seq_from_cds__NO_9361

ACAUAGCGGCAAUGGAUCACUUGGGGUGUGUAUGGCAGAGAGAUCAUGUGGCCCUGCAUCUUGAAGAAGUCUGAUUUGUAGAUC

>random_seq_from_cds__NO_9362

AACCUCACUGACAGCCUCUUGUGUCUUCUUGACUUGGCGGAUCUCAGGGGUAUCGGGAGUUGUAUGGAU

>random_seq_from_cds__NO_9368

GACAAGCUUGUAGUCAUUCCUUGUUUUCAACAUGUGAGCUUUAUACUUGAUCAUCAUCACGUAGAUCAUAAGCAUGCUUGGCAUGGAGGAUUUCAGGAGUGUCCCAGACG

>random_seq_from_cds__NO_9370

UUCCUGACGUGAUCAUUGACUUGCAAGUCGGGGUGGCAAAUCCAUUCGUGGAGGCGCAGGCGGUAAUCAAUCUCACUGACU

>random_seq_from_cds__NO_9374

GACAAGGUGCUGGAAGGCAGGAUGUAGCUGGUGGCUUUCACUCUAUCCCAGGCCUCCUUGUACAGGUUCACUGCUGUAAAGAUUCUU

>random_seq_from_cds__NO_9378

AGGUCAUCUUUAUACACAUUCAUCACUCAAGAUCUCCUGGGCGUUUCGGACGCGUAUAACAUUGGGUUCUUCCAGAAGAGACGUCCACUGGUGGAAAUAGUGUCGAUACU

>random_seq_from_cds__NO_9386

AGUGGGAUCCAGCCAAUGCCUCGGAGCCACUCCAGGUCAGCUCUGUAGACAUUCAUCACUCUGUAGGUCAUAGGCCUUUCU

>random_seq_from_cds__NO_9390

UCAGCUUUGUAUUUCAGCCUCACUCUGAACGUCAUUGCAGUGAUGGGCAUGAACAAAUGGCACAGCAUCUGG

>random_seq_from_cds__NO_9392

UGUGCAGCAGGGGGGUUUCUGUGAGGGUGUACUUUGAUUUGGUGGCAUUCCAGUCUUUCCGGUAUUUAAUCAUCAUCGAGGAUCUCGCCACUU

>random_seq_from_cds__NO_9395

AACAUUCAUCGCUCUGCAGUUCGUAGGCCUUCUUGGCCUGAAUCACAUCGUUCUGGUCGGGCAUGCAGGUCCACUGGUGCAGGUA

>random_seq_from_cds__NO_9410

UGAAUUUGAGCUGGUCUGCAGGCUGGCGAUACUUCCUGUCACUCAGGAUUUCUCCAGCUCUCUUCACUUUCUCGACCUCUAC

>random_seq_from_cds__NO_9420

GUCUGACUUAUACAAAUUCGUCACUCUGGAGGUCAUAGGCUUUCCGUGCAUGAAUGAUGUCAUUCUGGUCGGGCAGGCAGAUCCA

>random_seq_from_cds__NO_9422

UGUACCUUGUCUUGUAUUUCUCAAAAUCUUUCUUGUACUCACGGUCACUCUGCACUUUGGCAAUGUGGAGGGACCACAUUAUCUUGGGGUCAUCGUGUACU

>random_seq_from_cds__NO_9442

AAAAUCUGGGUGGCUCGUUUAUUUUUCUCAUCCUCGAGAGAACCACUAGUCAUCCAGCCAAUGCCUUUUAGCCACUGA

>random_seq_from_cds__NO_9448

GCUUUAGCCAGAACAAUGUCAGGUGUAUCAGGCAUGAUGUGGAUCUGAGUCUUGUCUUUGUCCCAAGCUUCUCUAUAAAGUCUCAU

>random_seq_from_cds__NO_9454

CUUGUAUUCUCUUUCACUCUGCAUUUUGGCUACAUUCAUGGACAACACAAGUUUUGGGUCAUCUUGCAGACUCCGGAAUCCAACAUGGU

>random_seq_from_cds__NO_9457

AAAUAUCUGGUGUAUCAGGCAUCACAUGAAUCUUGGUCUUAUCUUUAUUCCAAUCAAUGGUGUAUAAAUGCCUUGUUCAUGGUAUG

>random_seq_from_cds__NO_9460

UGGUCAGCUCAAUGUUCAUUGCAUCUGGAAGGAGGAUGUACUUGUGAAUCAGGUGCUUGUAGUUAGUGUUGGU

>random_seq_from_cds__NO_9465

AAUCUGGGGGAUAUCAGGCAUGAUGUGGACUUUGGUUUUGUCAGCCUCCCAUGCUUGUUUGUAGAGAUGCUUCGUUCAUAAU

>random_seq_from_cds__NO_9470

GUCCACCGGGGUGUGGAAGGAGGUCUUGGAUUUCUCAUAUCCCUUCUUGUAUUCCCGGUCUGACUGCA

>random_seq_from_cds__NO_9475

CAGUUUGUUACUCUUGUUAAGUGCCUGUUCCAUUGUGUCCAUGGCGUAAGUGAACUUCAGCUUCUCGGGGUGCUGGCGAUA

>random_seq_from_cds__NO_9486

ACUCGUCUUUAUACACAUUCAUCACUCUGAAUUUGAUUGACAUUCCUCGUAUGCUCAAGACUCAUGGCGUCAGGUAGGUAUGUGUAA

>random_seq_from_cds__NO_9487

UGAUGCAAUGGCUGUUUGUAGUUGACAUUGGUAGCGACAUCCUGGGCCAUCUUUGCAGCUGUGAUGCUAACCAUGUCCCCA

>random_seq_from_cds__NO_9490

AUGUUUCUCGAUGCCUUGGCUGCAGUGAUGGGAAUAGCAUCGCCCAGCACAUUGUUGCCCUUGGCUAUUAAGUCAUAC

>random_seq_from_cds__NO_9500

GAUGAACUGGGGCAGGUCUGGUGGCAGGUUGUAUUUGUGAAUAAUUUCCUCUCCUUUGGCUUUGUAAGCGAUCAUCACUCCUCUGGGCCUGGUUAAUUUU

>random_seq_from_cds__NO_9503

UUUGUAUUCAACCAUCGCUCUGCAGCGCAUAUGCCUUCUUGGCAAGGUCCACAUUGAUGCUAUCAGGGGGGUAGCUGUAACUGUGUAAG

>random_seq_from_cds__NO_9519

ACUUGGGCUUGUAGCAGAACAGGAGAGUCUGUAACUUGGGUGAAUUUUGUCUUAUCUGGAUGGACUUUGUAGGUGUGCGUCUUU

>random_seq_from_cds__NO_9521

GUAUUCUGCUUUGUAGUUUUUCAUCACUGUUUUGAGCUGUGACUUUCAUGCAGUGUGAAUGGUAUGGAUCCUCGAAGCUGCCUACAUAAUGUCCCAAAA

>random_seq_from_cds__NO_9523

CUUUGCUUUUGUCUUUUCAUAGUUUUCCUUGUAUAGUUUCGUCACUUAGGGCAUCUCCUGCUGCCUUCAGCUGCCUAAGCUGUGGGUUCUCUGAA

>random_seq_from_cds__NO_9526

UGUGCUAAUGUUAUCAGCAUUCAUUCUGGCAUUUGCAACUUCAAAGCAAGGUGUCUCACUCCAUUUGCCUUUGAUUUUAUUUUCAUAG

>random_seq_from_cds__NO_9528

AGCCAGAGGCGUGAAUUGAGCUUGCUGUUCAGCGAGACCUUUUUUGUAGGCAACCAUCACUGAGAGCUUUCUGGGCCUGGGCAACUCUCCUCAGUUCCGGGC

>random_seq_from_cds__NO_9530

CGGCCUGGACAAGUUCAGGGGCAUCAGGAGGAAGCAGGUACUUAUCCUUGGUGUCUUCCCAGUUCUGCUUGUAUAAAACCCUUAC

>random_seq_from_cds__NO_9552

UGUUCCUGUUACACCAGUCCCCUUGGCAGCACUCCACGGCUUGGCCAGGGGACGGCGGGGUCUUACAGGUCAUCUUUCCCU

>random_seq_from_cds__NO_9559

ACUGGUGUAAGCAAACCUAGAGUUUCUGACGCUGUCCAGCCCAACAACUAUCUCAUCAGGACAGAGCCAGAACAAGGAACCCUCUAUUCACCAGAACAGACAUCUCUCCA

>random_seq_from_cds__NO_9583

UAAAACCAUAUCUGACUCUUCUAGCAGAAAGUUCCAACCCAGCCACCUUGGAAGGCUCUGCAGGGUCUCUCCAGAACCUCUCUGCUGGCA

>random_seq_from_cds__NO_9585

GAUAACGAUAGAGUUGUUUCUUCCGUGGCAACAGCCUUGAGGAAUAUGGCACUAGAUGUUCGCAACAA

>random_seq_from_cds__NO_9589

GCAAUAUCGGGACCUCCGGAGCAUUUAUAAAAAGGGAUGGGUGGAAUCAGAACCAUUUUAUUACACCUGUGUCGACAUUGGAGCGAGACCGAUUCAAAU

>random_seq_from_cds__NO_9606

CACUGGAUGCGCCAAGAAAUCUCUUUUGGAAAAUUAAAACUUACGAACAACAAAGGAGCUUCAAAUAACAAUGGGCAGGAUGGUGGU

>random_seq_from_cds__NO_9644

CUUUGGCUUGCUUCGUGGCCCCUCCAACACCAGGUGAAGCUGUUAGUCCCAGUAUCUGAGGAAGGGGAAUCACUGGUUUGUUUUCUUU

>random_seq_from_cds__NO_9647

CAGGACUUGACAACUUCUGGAAAUGAUAUUUUCAGUUGGGUAUCACCACUUAAUCCAAUAACACGAUACCAUUUCUUCAAAAAUGGUUGGAACU

>random_seq_from_cds__NO_9658

CAGACUCCCUUCUCCAAGGUGCUCAGCAGCAGUUCAACUGCCUGCAUGUUCCCGGAGGUGGCGAC

>random_seq_from_cds__NO_9664

AUGGAACUUACUCUCGCCUUCAGUUUGGAAACCUGCAGAAUUAUGAUUGCCAUGUUGGAUGAGAGAUCACACAGGAAAAAUGGGAUUUAAUGCAUUCAAAGAGCUAUGGG

>random_seq_from_cds__NO_9673

ACACUGAAGGCGGUGUUUUAUUUCAAAUCUUUUUGAAAGAUCACCAAAUGCUUUCUUGUUUAACAAUUUUUGCUGCAUCUG

>random_seq_from_cds__NO_9709

AUAUUGGCUUUCUGAAUGGCUGUCUUGAAGGGCUUCUCCAGCUCAGUCUCAAGUGCUUCAAUGAACCGCCUCUCCUUUCCAAUUC

>random_seq_from_cds__NO_9721

GAUGAGCAACUGAAAUGAUUGUGUGCCCAUGCUGAAUCCUUACUCAGCACUUCUUGCACCAUGGCUUCACUCUCAUUGUCCAGA

>random_seq_from_cds__NO_9723

UUCUGGCCACCACUCAUCUGGCCUCCUCCUUCUCCAACAAGGGUGUCAAAUUGCCUGUGGCAGGUCCAU

>random_seq_from_cds__NO_9727

UUUAAUGACCAUGUUGAGGUCAUUUAGAAUCCUUCACCUCUGGUCUGGAAGGAUAAUGGAAGGUCACAUUAUGGAAUUCAAUUU

>random_seq_from_cds__NO_9744

AGAUCCCGGGCGGGGGCACCGAGGGCUACCACGUUCUGCGGGGUACAAGAAAAUUCCCCAGGACACAGAGCUGGUUUGGAGCCUUUCUUUGAUUUUAUUGUUUCUAUUAA

>random_seq_from_cds__NO_9758

ACUCACUGUGGAUGUGACGCCCCCCACUGCCAAGGCCCCCACCACCGUUGAGGACAGAGUCGGCGACUCCACCCCAGUCAGC

>random_seq_from_cds__NO_9761

UGACAUUAGAACUUUCUUUCCUGAGUAUACCCAUCAACUCUUUGGGGAUGAGUGAAACUGCUUUUGGUUACAAGGGUCUAAAGAU

>random_seq_from_cds__NO_9774

CCGCCGUGAUGGUGGUGGUGGUGAUGCGGCUGCGGGGUCUGAGUGGGGUGCAGCAGCGGCGCCGUGGCCUGCAGGUGUGAG

>random_seq_from_cds__NO_9779

CUGGAAACUGGAGUAGAUGGUGCGGGGUUUCCGGACUUUCUUUGGCUUCCCGUUCACUAUCCGAAUUUCAGGCUCAAGGUCC

>random_seq_from_cds__NO_9780

UCCUUCUCCAGGCUCGUUGUUGGCUGGGGACGAACUGGUUCCAUAGGGAGCGUAGGAGGUGUAGGCGG

>random_seq_from_cds__NO_9781

CGGUGUAGCCCAGGUCAUAGCUGCUCUUGGCGGAGUAAGGGACGUUGUUGAGGCCGCUGGCUUGGUACUGGUAGGAACCCA

>random_seq_from_cds__NO_9783

ACCGGAAGGGUGGGCGACUCCUGGGGCUUGUGGAGGCUGCUGCUGCUGCUGCUGUUGCCACCCGGGCC

>random_seq_from_cds__NO_9793

GAAGAUGGGCCUUAUGAAGUUGGUGGAGAGACUGAGCAUGAUGAAAGUCUCGUUCCUGUUCCUGCUAACAGUUACUUAGGGUUUUUCUUUGGACUCA

>random_seq_from_cds__NO_9811

UUGCUAAACCUUCCCAGGUGUAUUUUGGAGGUACAGUUGUUGGCGAGCAAGCUAUGAAAUCUGAAGAUGAAGUGGGAAGUUUAA

>random_seq_from_cds__NO_9812

UAGAGUAUGAAUUCAGGGGUAAUAAACUUAGGUAAACCUCUUACAAACCUCGGCACAGCAACCUUGAACAUUCAGUGGCCAAAAG

>random_seq_from_cds__NO_9817

CUGCCGAAAAUAUCAGGCUGCCAAAUGCAGGCACUCAGGGUUCGAGUGACUGUGUUUCCCUCAAAGACUGUAGCUCAGUAUUCGGGAGU

>random_seq_from_cds__NO_9824

UGGGAUAGUGUCGAUAAAAACCCUUCCGCACCCAGUUGGGCAUGACAUGGGUGCUGGGUGAGCGGUGGUGUGUGUUGAUGACGA

>random_seq_from_cds__NO_9825

UGACAGUGAUGAUGAUGGAGGCAAUGACGAACACCAUGGUGAACAGCAUGUAUUUUCCAAUCAAGGGCACAGCACUGGACGUGG

>random_seq_from_cds__NO_9831

CUCACAGUAGCUUUUAAAGAUGGCUGGAGGUGUCCACGUGAUGUGGCCAGUGUACUGCAGGAGCACUU

>random_seq_from_cds__NO_9838

CGGGGCGGGCGGCGGCGGCGGCAGCGGCGGCUGCGGCGGCGGCGGCGGCAGCCUCCGGCUUUGCGUACCCCGGGACCUCUGAGCG

>random_seq_from_cds__NO_9841

CCGUAUGUCGCACGGCGUGGGCUUACAGCAGAAUGCGCUCAAGUCAUCGCCGCACGCCUCGCUGGGAGG

>random_seq_from_cds__NO_9853

ACGCCGGGCUCCACGACCCUGCUCCAGGGGGCUCCCUGCGCCCCUGGCUUCAAGGACGACACCAAGGGCCCGCUCAACUUG

>random_seq_from_cds__NO_9858

AACGACUUUGACGAGUGCGGCCAGAGCGCAGCCAGCAUGUACCUGCCGGGCUGCGCCUACUAUGUGGCCCCGUCUGACUUCGCU

>random_seq_from_cds__NO_9860

CGGCCUGGAGCGCGCCAAGUGGCCGUACCGCGGCGGCGGCGGCGGCGGCAGCGCGGGGGGCGGCAGCAGCGGGGGCGGCCCCGGCGG

>random_seq_from_cds__NO_9885

GCCUCUGCCUCCGAGCCCGGCCGCUACGUGCGCUCCUGGAUGGAGCCGCUGCCCGGCUUCCCGGGCGGUGCGGGCGGUGGC

>random_seq_from_cds__NO_9903

CCAAGAGCUGGAGGAAGACAGAGCCGAAGGCCUGACAAAUUAAAAUGGUCAUGAGUUCGUAUAUGGUGAACUCCAAGUAUGUGG

>random_seq_from_cds__NO_9908

GCCCUCCGGGACGGCACUCAAGCAGCCGGCCGUGGUCUACCCCUGGAUGAAGAAGGUGCACGUGAAUUCGGGUGA

>random_seq_from_cds__NO_9910

AAAAAGAAUUUCAUUUUAACAGGUAUCUGACAAGGCGCCGUCGGAUUGAAAUCGCUCACACCCUGUGUCUGUCGGAGCGCCAGAUC

>random_seq_from_cds__NO_9931

GGGCCUACGAACCUGGUGCCGCACCUGCCGCGGCAGCUGGGGGCGCGGACUACGGCUUCCUGGGGUCC

>random_seq_from_cds__NO_9947

UGUAGUUAAGAUUGUGAAUCUAGCUUGCAAAUAUAAUCUUUGUAUCAUACCAAUUGGUGGGAGGAACAAGUGUUUCAUAUGGCCUGAUG

>random_seq_from_cds__NO_9973

AAGGCUUGGAUCGUACUUUGGAGCUUCUGUCUGUGCUGUGGACCUCAAUGCAGAUGGCUUCUCAGAUCUGCUCGU

>random_seq_from_cds__NO_9980

GUUGAAAAUGGAUGGCCUUCUGUGUGCAUAGAUCUAACACUUUGUUUCUCAUAUAAGGGCAAGGAAGUUCCAGGUUACAUUGGUUUUGUUUUAUAACAUGAGUUUGGAUG

>random_seq_from_cds__NO_9992

UUUUAGCCCCCAAACUGAUAAGCUGUUCAACAUUUUGGAUGUCCAGGACUACUACUGGAGAAUGCCACUUUGAAAAUUAUCAAAGAGU

>random_seq_from_cds__NO_10003

CCCUCCUGGUAUUCCUGGGAGAAAUGGUGACCCUGGUAUUCCAGGACAACCAGGGUCCCCUGGUUCUCCUGGCCCCCCUGGAAUCUGUG

>random_seq_from_cds__NO_10004

AAUCAUGCCCUACUGGUCCUCAGGAACUAUUCUCCCCAGUAUGAUUCAUAUGAUGUCAAGUCUGGAGUAGCAGUAGGAGGACUCGCAGGCUAUC

>random_seq_from_cds__NO_10019

CCAUCUGGUCCCCGAGGUCAGCCUGGUGUCAUGGGCUUCCCCGGUCCUAAAGGAAAUGAUGGGUGCUCCUGGUAAGAAUGGAGAACGAGGUGGCCCUGGAGGACCUGGCC

>random_seq_from_cds__NO_10029

UCCCUGGUGCUCGUGGUCUUCCUGGUCCUCCUGGUAGUAAUGGGUAACCCAGGACCCCCAGGUCCCAGCGGUUCUCCAGG

>random_seq_from_cds__NO_10038

GGCCCCAGAGGGACCUGUUGGACCCAGUGGACCUCCUGGCAAAGAUGGAACCAGUGGACAUCCAGGUCCC

>random_seq_from_cds__NO_10055

UUUACGUGGUACACUGGAUGGGUUUGCUGAAAUACAUGUUUCUCCUGUUUCCAUGUUGCAGUAAACUUUGAUUGCAUCUUC

>random_seq_from_cds__NO_10059

UGGCCAGGGGGACCCGGAGGGCCAGGUGGGCCAGGCUCACCAGGAGGGCCCCUCAGGUCCUGCUUCUCCUACACUGCCUCGUACA

>random_seq_from_cds__NO_10060

CCUGGAGGUCCAAUUGGCCCAAGUGGCCCAGGGUUUCCUUCUUUACCUGAAGGACCAACUGGGCCUGGAGGACCCUCUUGG

>random_seq_from_cds__NO_10063

GAACCCCGGAUCUCCUCUUUGUCCUGCAUCUCCUGGAGCACCCACAGGGCCAGGAGUUCCAGGGGCACCCU

>random_seq_from_cds__NO_10065

GUUCCCCUACAGGACCAUUGGAGCCUGGGGGCCCCACAGGUCCAGGUGGACCUUUAUCUCCUGUUGCACCAGUUGGUCCUACUU

>random_seq_from_cds__NO_10066

UUCCUGGUGUUCCCCGCUGGGCCUGGUAGGCCGGGCAUGCCUCUCUCUCCACGUUGCCCAGGCAUGCCAACAAUUCCUCUCUGCCCGGUCGUUCCAGCU

>random_seq_from_cds__NO_10073

UUUUCUCCAGUAGGACCUGCCGGACCUGGAGGGCCCAAAGGACCUGGAAGACCCUCUUGCACCAUCAUUUCCAGCUGUGCC

>random_seq_from_cds__NO_10085

ACCCUGAGGUCCUGGAGAUCCAGGAGGCCCUGCUGAGCCAGGAGGACCAGAGGUACCUGGAGAGCCCCGUUGGGCCUUU

>random_seq_from_cds__NO_10133

GGGUUUGCUUGGUGUACCAGAUGAGUAUGAGGAUAUUUUUGUAAAAAUACAAAUUCACACUCUCCAGAGCAGUAAUUGGCCUUAUAUCUUUUAGG

>random_seq_from_cds__NO_10137

GGAUUCAGGUUGUUUGAGCCAAUUUUGCAACACUGUCUUCACAUCAAUGCUCUGCCAAAUACCAGUGCCUGGGUUCAUGUCAAGUU

>random_seq_from_cds__NO_10140

UCCAUCCACUUGCAUUAGAAAAUCAGCACUCUGUAGGCAUGGUAAUGAUUGUUUCCGUUGUAGCGUGAUAAUCGUCAUCUUCCAAAGAGCCAUCG

>random_seq_from_cds__NO_10144

GUCAGAUAUCCUCCGGGAGCUGCUCUGUGUCUCUGAGAAGGCUGCUAACAUUGCCCGGGCGUGCAGACAGCAGGAAGCCC

>random_seq_from_cds__NO_10164

UUCAGAAUGGUCCACCCAGGUGAAAGUUAUUCCUCCGAGAUGGCUUUCACUGAAUCUUAAUAAAAAGGUGCCAGGCAUUUUAUCCUUUAGCAA

>random_seq_from_cds__NO_10182

GAGUAGGUUUUUCUCUUUGGAAACACGACCUAACUGUUCAUCCAGUUGUAUUAACAAGUUUUGAAGAAGAAUCGUUGCCAUGGUUUC

>random_seq_from_cds__NO_10201

UGGAGGCGUUGGUCCAGCCACCAUCUGUGCCAAGAAUUCCUGCCAGAAACUCAUUGGAAGGGCACUGGAACGAACAGAUGCUGGAUAUAGCCUGCAGGCUUA

>random_seq_from_cds__NO_10202

UUCUGAAUGAAGUCUCCCUUUUGGGCUCGGCGCCAGGUGGGAAAGUGGAGUUCAAGAGGACUCUCAUCAUCAGCUUCCUC

>random_seq_from_cds__NO_10214

AGGCUGGAUUCAUGAACGAUGGCAGAAUCUUGGCCCUGGACAUGGAGCAUUACAGCAAUGCAGGCGCCUCCUUGGAUGAAUCAUUAUUCGGUGAUAGAAAUG

>random_seq_from_cds__NO_10223

UUCAGCUGUUGGAUACUUCAGGAGGUUAUGAGUCAGACAUGAACUGGGAGAAAGGCGAAGGCCAGCCCUUCGAAUACUUUGUUUAUGGAGCUGCCUGUUCCGAGGUUGAA

>random_seq_from_cds__NO_10243

UCAUCUCGGCGGGUGACAUUUCACUAUAAGAGAAGCAGUCUGAGUUACUUUGGAGGUGUCUGUUCUCGCACAAGAGGAGU

>random_seq_from_cds__NO_10251

AACUGCGGGAAGGAUGGAGACCGGUGGAUUCAGUGCAGCAAACAGUGAUGUGUUCUGUGGAUUCUUACUCUGUACCAAUCUUACUCGAGCUCCACGUAUUGGUCAACUUC

>random_seq_from_cds__NO_10254

GGCCAUGGGGGUGUGUAGUAAUGAAGCCACCUGCAUUUGUGAUUUCACCUGGGCAGGGACAGAUUGCAGUAUCCGGGAUCCAGU

>random_seq_from_cds__NO_10266

ACUGUCUUGGGGGAUGAGACAACAGGAGCGGAUGGAGUCGCUGAGGCCCAUCCAUUGCUGGUAGUCGGGGUACUCCCCUCGCCGCAGCAAGUAUUGU

>random_seq_from_cds__NO_10270

GGAUCCAGCUGCCCUCCAGCACAUUGAGGGAGUGAAUUUCAGUGAGGUGGAAGCGGUCCUGAAC

>random_seq_from_cds__NO_10296

CCAUUCAGCAAACCUUACAGGCCCUAUACAGGUACUUCAUGUCCUGUGUGAGGUUGUAGAGUGU

>random_seq_from_cds__NO_10311

GAAGGUUCCCCACAAAUAGAAGUAGCAUCCAUCAAACAGCUUUGGCAACCAGCUGUUCUCUGUUGAGCCUGCUUCUGCGUGGACCUUCAGGAAUUUCAU

>random_seq_from_cds__NO_10317

UCUUGGCUGCAUCGUGAAGUGGUGAGUCAUUUUGAUACCCGGUGGUGUUCACCAAUGCCUUAUGCUGGAGCAAUAAUU

>random_seq_from_cds__NO_10334

GAUCCUCGGCUGCCGGUUCCUCGGCUGCCGAUUAUCCGGCAUCAUGGUGCGGUCGGGGAAUAAGGGCAGCUGUUGUGCUGUGUAU

>random_seq_from_cds__NO_10342

GCAUUCCAUUCACUGGCCCUGCCGACUGACCAUUGGCUCCAAUUUGUCUAUAAGGAUUGCAGCCUAUAAAUCGGAUUCUACAGGAGAGAGUUAAAA

>random_seq_from_cds__NO_10385

CGGCUGACCUGGUGGUGUUUGCUGAGCUGUUUUGGAACCCAGGGGGUGCUGAUCCAGGCUGAGGACCGCGUGCACCGCAUUGGACAGA

>random_seq_from_cds__NO_10391

GUAACAUGGGCCAAACGUACCAAGAAAGUCGGGAUCGUCGGUAAAUACGGGACCCGCUAUGGGGCCUCCC

>random_seq_from_cds__NO_10393

GAAGAGACGAGCUGUGGGGAUCUGGCACUGUGGUUCCUGCAUGAAGACAGUGGCUGGCGGUGCCUGGACGUACAAGUACCACUU

>random_seq_from_cds__NO_10400

GGCAGGGCCAAUGGGAUGAUGGAGUGCUGGUAGACCAGGGCAGACAGCGAUCCCGAAGUUUGGCUCAUUG

>random_seq_from_cds__NO_10402

CCCUUUUUUAUUCUGCUGCAUGAUGGUUGGAGGUGGCGAAGACACCUUCAUGGCCAGCCCGUACGCGCCUCGGAAGGAGUGACUGUC

>random_seq_from_cds__NO_10406

GUCUCCCACUGACAUCCUUCGCUUCUCUGGCAAGGCUGGUGUUGGGCUCCCUUGCCGGAAGGCUGCGGAGUCUGGUGACGGGGAGGUGGCAGGGCUGCUCAGGCCAGGGU

>random_seq_from_cds__NO_10409

CCCAGGCUGGGGCUUCCAGGGCUGGCUAUUGCAUUGCUAUGAAGACCGGAGGCCAGGUUGCCUUGGUGAGCCCCAGGGUGCCGGCCCAG

>random_seq_from_cds__NO_10422

CUGGACAGGGGAGCGAACCCCAGGGCUACGAGGGGAUGUGGCUUCUGGAGACUGGUUCUCAUACUGUCCAU

>random_seq_from_cds__NO_10424

CUGCUACCCUGUGGGCCACCAGCCCUUCUAAAUUCAAUGGCUCCUCCUCUGGAGUCCGGGAGGGGUCUGAAGUCGCUUCCUUUGGUGAGAGC

>random_seq_from_cds__NO_10429

UGUGUAGUGGGGCAGCAGCGGAGGCUGGCUCCAGGUCCAGCAUCAGCAUAUUGAGUGUUUCGAUGGACUGUUCAAUCUCCUGCUGGGAGGCUGCUCGCGGGAACUCAGGG

>random_seq_from_cds__NO_10430

AGACUGGGGAUGGGGGUCUCUGCCAAUGGCUGAGGGCUGGGCCUGCUGGCUACAAGACUCUCCAAGUGGGC

>random_seq_from_cds__NO_10433

GGAGGUGGUCACUGGCUGUGGCCAGGCUGGGUGGGGCCCCUCCCUCUGGUAGCCAGCUAAACCCUCCUGUGC

>random_seq_from_cds__NO_10435

ACUUGUCCAGACCGUUGGUCAGUGGGGACAGGGCCUCUGGGUAGCCCCCCUCACUGGUGUUGGUGAC

>random_seq_from_cds__NO_10442

CCUCCCUCCAUGCCGUCAUCUCGAUGCCCACUGAAGUUGUCGUAGGAGUCCCAGCGGAUGAGGGGGUCGGA

>random_seq_from_cds__NO_10446

CAAGAGCAGUCCUGGCUCGAUGGUGAUGCAGACGCUAGUCUGGCUGUCUCCUGGGAUGUUGCUAGAUGCCAG

>random_seq_from_cds__NO_10452

GCCAAAUUCCAGUACCCUUGGCAUGGAGCUUCGUGAUGUCAGGUCUCCGCUCAGAGAGGUUGAACAGCCAGGUAGUUGCCUCCAUGU

>random_seq_from_cds__NO_10465

CAGACCUCCUCCUUAGUGUCCACCUGCCACACUUUCAAGAGGCCACUCAUGUCCCCUGUGGCCACUAGAGU

>random_seq_from_cds__NO_10469

CGUUGCCCUCUUCCUCCUCUUCUUCCUCAAAGUCCACAUCUUCCAUCUCCUGGGCCAGGUCAUCCUGGGUCCGGCGGACCGGGAUCAAGUUCUACCACCUCG

>random_seq_from_cds__NO_10484

AAAACCCACACUGUGGGCUCCGUGGGCCAAAGUGGAACAGGUGAAGUUCGAUGCCACAUCCAUGCAUGUCAAGCCUCAGGUGGCUGC

>random_seq_from_cds__NO_10499

CCUGUCCAUUGAAGAUUUCACUCAGGCCUUUGGGAUGACUCCAGCUGCCUUCUCUGCUCUGCCUCGAUGGAAGCAACAAAACCUCAAGAAAGAAAAAGG

>random_seq_from_cds__NO_10500

ACUAUUUUGACAUGGCUGCGCUGGGCUGCGCGAGGCUGAGGUGGGCGCUGCGAGGGGCCGGCCGUGGCCUCUGCCCCCACGGGGCCAGAG

>random_seq_from_cds__NO_10506

GGUGAUUGAUGACUUUAUGACUCGACUGGACCAGCUGCGGGCAGAGAGUGCUUCGGGGAACCAGGUGUCG

>random_seq_from_cds__NO_10509

CCCGUGCUGCCUUUCUGGAAGCGAUACCUGGAUGGUUGGAAUGCCAUCUUUUCCUUUGGGGAAGAAGCUGAUUGAUGAGAAGCU

>random_seq_from_cds__NO_10526

UGCGGAGCCACACGCGCACCGAGUGCAAAUGCCACGGGCUGUCGGGAUCAUGCGCGCUGCGCACCUGCUGGCAGAAGCUGCC

>random_seq_from_cds__NO_10529

ACGCGCGGUCGCGCCUGCAAUAGCAGCGCCCCGGACCUCAGCGGCUGCGACCUGCUGUGCUGCGGCCGCGGGCACCGCCAGGAGAGCGUGCAGCUCG

>random_seq_from_cds__NO_10543

GGCAGCAUGUGCUGCGGCCGCGGCCACAACAUCCUGCGCCAGACGCGCAGCGAGCGCUGCCACUGCCGCUUCCACUGGUGCUGU

>random_seq_from_cds__NO_10545

CAGAGGUUUCUUCCUGUCCCUGCUGCAGCUGCCACAUGUCAGCAUACACCCCACCUCGGGACAACAGAGCCUCGUGUCCGUCCCCUCUCC

>random_seq_from_cds__NO_10550

UCAUUCCCAGCUGUGACACGGCCGUAACGGAUAUUGUCGGCGAUGGUGUCAUUAAAGAGGACAGUGUCUUGGG

>random_seq_from_cds__NO_10553

AAGACACGUCCUGCAGAGUCUCCCGCCCCAUCGGCAUAGCUGAAGUGCACGUUCUCAAACUCAAUACGGCCCUUCUGAAAGCGAAGGGGCCCUGCUCCAGGAAGGUCCUU

>random_seq_from_cds__NO_10554

CACCUUCUGUCUCCUCUUUCAGCAAGUCAAACAUGUUCUCCAUGUCAAUGAAGUUGGUCUGGAUCAUCCCUGUAGUAGGUGCCAA

>random_seq_from_cds__NO_10580

UGACGAUGACGGUGCAGCCGCUCUCCCACACCAUCCUGCCAGAAGUCUGCGAUGGUAUGGGACAGCGGGCCCUGCGUGGCUAUGU

>random_seq_from_cds__NO_10585

GGACACACUGCUCACCCGUGAAGGCUCCGGUGGACCCUCUGCCCGGUUGAACAAGGACUUCGUGGCCAUGUGCUGGCGGCACAGGUCCCUGGUACUCAAAGGUAGUGUCA

>random_seq_from_cds__NO_10590

CUCCAGCAGCUUCACUCCUGCAGCCAGGCUCAGGGGCCUUCUGAUCAGUGACGAUGUAGCCAUAUUCCUCUGCUGCUGGGCGGGCUGAGGGCUGU

>random_seq_from_cds__NO_10594

CAACCCCUCCCCGGAUUUCUUCCUGCACCCUUGGGCAGUAGCUGCAGCAGGGUCAGGAGUGUGGAGAGCUGCUCAGGG

>random_seq_from_cds__NO_10599

GGCAGGGGCUUCAGCCUUGGGCAGGGGGCCGACACUGACCAUCCCUGGGGAGCCCUCUGAGACCCUGGAGCCAUCACGGGAG

>random_seq_from_cds__NO_10602

GGGCAGCAGGGGCGGAGCCAGUGGGGAUGUCCUGUAAAAGCAGCUCUCCAGCAGGACCAGGUCUCUUGGGUGCCAAGC

>random_seq_from_cds__NO_10621

CAAAAGCUAUGAUGGUGGAGGAGUUCCUGGUCAUGAAGUACCUUGCUCUCGGGCUUAAUAUUCCAUUUCUCAGUCUCCUUG

>random_seq_from_cds__NO_10633

GGACAUCCGGGCUCAGUAUGAGACCAUCGCGGCUAAGAACAUUUCUGAAGCUGAGGAGUGGUACAAGUCGAAGGGUGUCAGACCUGACCCAGGCA

>random_seq_from_cds__NO_10644

CAAUAGCUCUGAGCCCCUGCUAGGCCUGCUGGCACUGUCACCGGGAGGACCCGUGGCUGUGCCCAUGUCUUUGGGCCAUGC

>random_seq_from_cds__NO_10664

ACAGCGGCUGACAUAGGGGCGGAUCGCCUCUUCAGAGAGUGGCAUCAUGGGGAGGGGCGCAGCGCUGGCCAGCCAGUAGGAUCUGU

>random_seq_from_cds__NO_10677

GCCAGGUGAUCCGGAGGGACCCUUUUAUUCCCGGAGGACCUGGUAUCCCUGGAUCCCCCUGGAGGCCUCUUGG

>random_seq_from_cds__NO_10683

CUUGCAUCCCGGGAGUUCCUUUAUCACCCUCUUUCUCCUGGGAAUCCAUCAUCUCCAGGAGGUCCAGGUUCCCCAGGUGUUCCCUUUUGUGAAAUGAU

>random_seq_from_cds__NO_10686

ACCAGGCAGCCCCCGGGGUCCCCUUUGGACCUGGAGGACCAGGUAGCCCAUCAUCUCCAAAGGGACCUGGGAUUCCUGGGAGGC

>random_seq_from_cds__NO_10715

UGAAAGCCCAAUGGGUCCUGGGGCUCCCAGGGGUCCAAUUGGACCCUGUGGCCCUGGUGGUCCUGGUGGACCCCCGAGACCCCUUUUCAGGAACACAGUGGCAAACAGAG

>random_seq_from_cds__NO_10728

CCCCUAUUGCUUCUGUGCCUGGAUCCACUAAUACGGGUACUGUUCCGGGAUCAGAGAAAGACAGGUGACUCGAU

>random_seq_from_cds__NO_10734

AUCAGGAAGGUGGAGGUCUCUAUGCACUAGGUCUUAUUCAUGCCAAUCAUGGUGGUGAUAUAAUUGACUAUCUGCUUAAUCAGCUU

>random_seq_from_cds__NO_10756

UUAUUUCCAUCAAUUUCACCGUCUUCCAUGGCCUCCUUUGGCAUCCUCCUCACUGUUGAAGUCUACAAAACCAAACCCCUUUGGAGGACCCAGUUUCCCGGUCAGUAACU

>random_seq_from_cds__NO_10773

UGUGGCUCCCUUCUUGCCAGGUGUGGUAACUGCUUUGGCUGGUGUAACUGUCUUCUUGGCAGGUGUUGCUGCUGCCUUUUUGCC

>random_seq_from_cds__NO_10778

AUCCCAGGUUGAGGAGGAGAACCCGGACUUCUGGAACCGCGAGGCAGCCGAGGCCCUGGGUGCCGCCAAGAAGCUGCAGCCUGC

>random_seq_from_cds__NO_10788

UGGAGGGGUGGUCGCAUCGACCAUGGUCAUCAUGAAAGCAGGGCUUACCGGGCACUGACUGAGACGAUCAUGUUCGACGACGCCAU

>random_seq_from_cds__NO_10794

GCGUUGCUUCCUCUGCUGGCCGGGACCCUGCUGCUGCUGGAGACGGCCACUGCUCCCUGACAUGCAGGGGCC

>random_seq_from_cds__NO_10823

CCCUGAUGGAGAUGACAGAGGCUGCCCUGCGCCUGCUGAGCAGGAACCCCCGCGGCUUCUACCUCUUUGUGGAGGGGCGGCC

>random_seq_from_cds__NO_10825

CCAGCUCACCAGCGAGGAGGACACGCUGACCCUCGUCACCGCUGACCACUCCCAUGUCUUCUCCUUUGGUGGCUACACCUUGCGAG

>random_seq_from_cds__NO_10833

UGUGUGUACUUGAGCCGGGGAAGUGGGUGCUCUGGGCCGUGCUCCCGCACCCACUUCUGAUAGGCCGUGGUAGGCCA

>random_seq_from_cds__NO_10835

GAAGUUGUCAUAGAGACGGACGAUGCACUCAGCCUUUCGCAGGAAGCGGCUGUAGGAGGCCUCCGUCCACCAGUGCAGCAGGUUCCCUGA

>random_seq_from_cds__NO_10842

AGAGGGCGCCAAGCGCCAUGCCAAAGUGGCGAUUGGCCUGGCCCAAGCAGACCCGGGCCAGCUCCUGUGGCUUGUCGCUGCCCUCCAUCUCCUGUGCCAGCUCGUG

>random_seq_from_cds__NO_10844

AUGUAGUCUGUCGCCAGCAGCACCACCUCCUCUUCCUCUGAGAAGUCCUCCUGGAAGAUCUGGUCUAGCAGCCACUUCCACCGCAACGU

>random_seq_from_cds__NO_10863

AGGAGAACCGCACCUACCCCGUGGAGUGGAUCAUCAUUGAUCCUGAAGGCUUCACAGGAGAACGGGGAGUGGGAGAUAGUCCACC

>random_seq_from_cds__NO_10866

AGGUGGUGAGAAGACAUCAGUGGCCAUCUCGGUGCUCCUGGCUCAGUCUGUCUUCCUGCUGCUCAUCUCCA

>random_seq_from_cds__NO_10869

CUGCCGGAGCUCCUGCACAUGUCCCGCCCAGCAGAGGAUGGACCCAGCCCUGGGGCCCUGGUGCGGAGGAGCAGCUCCCUG

>random_seq_from_cds__NO_10888

CUGAAGCAAGCCUUCCCAGCUGAGUACCUGCCUCAGUGACAUGGCAGCCAGCGGGAAGACCAGCAAGUCCGAACCGA

>random_seq_from_cds__NO_10891

UCACUCUGACCUGCGCCUUCCGCUAUGGCCAAGAGGACAUUGACGUGAUCGGCUUGACCUUCCGCAGGGACCUGUACUU

>random_seq_from_cds__NO_10898

UAACACAGAGAAGACCGUGAAGAAGAUUAAAGCAUUCGGUGGAACAGGUGGCCAAUGUGGUUCUCUACUCGAGUGAUUAUUACGUCAAGCCCGU

>random_seq_from_cds__NO_10899

GGCUAUGGAGGAAGCGCAGAGAAAAAGUGCCACCAAACAGCACUUUGACCAAGACGCUGACGCUGCUGCCCUUGCUGGCUAACAAU

>random_seq_from_cds__NO_10904

GACGUUGAUGAGUGACAUGGCCCGGGCCCUGUGCCGCCUCCCGCGGCGCGGCCUCUGGCUGCUCCUGGGC

>random_seq_from_cds__NO_10906

CAGUUCCAGGUAGACAUGGAGGCCGUCGGGGAGACGCUGUGGUGUGACUGGGGCAGGACCAUCAGGGAGCUACAGGGAGCUG

>random_seq_from_cds__NO_10907

GCCGACUGCACCUGGCACAUGGCGGAGAAGCUGGGCUGCUUCUGGCCCAAUGCAGAGGUGGACAGGUU

>random_seq_from_cds__NO_10910

GCACUGAGGGCAUUGUGUAGCAUGGCGGAGGGCGGCAGCCCAGACGGGCGGGCAGGGCCGGGCUCCGCAGGGUCGUAAUCUGAAGGAGUGGCUG

>random_seq_from_cds__NO_10924

AUCUCCAUGACUUUCUCCAUGGAACGGACAGCGUUUGCAUUGGGUCUUUGCUGUAAAACCUUUUCUGGGAGAGGAUCA

>random_seq_from_cds__NO_10928

GGGGGGGUCCAGGCCGCCGGUGAAAGCCAUGUUGACGUUGAAACCCACGCCGGGCCCUGUGCCCACCCUCAUCAGGA

>random_seq_from_cds__NO_10934

CGGUUGAGGGGGUUCGUGCCAUACAGGAGGGUGUGGGCUUCCGAGUGCACCGUCUGUAGCUCCUCCAG

>random_seq_from_cds__NO_10945

GUGCUUGUGCCGGCGGCUGCUCCAGUAAGACCAUGUGCUGCAGAAGAGGGCUGUGCGCUGCCCCUCCGU

>random_seq_from_cds__NO_10953

CAGUAGCGAGGGUCGCUGGAAAUGCAGUGGUUCAGAUUCCGGUGGGCCAGCGCCUUCUUUUUAUUGAGGAC

>random_seq_from_cds__NO_10976

ACGGGAUGGCCAGAGGCCGGGUACCUCCCCGAGGUCAUGGGUGACGGCCUGGCCAACCAGAUCAACAACCCC

>random_seq_from_cds__NO_10982

UCCUGCUGGGGCCUGGUCCUUCCAACCUGCCUCCUCGCAUCAUGGCAGCCGGGGGGCUGCAGAUGAUCGGGUCCAUGAGCAAGGAUAU

>random_seq_from_cds__NO_10984

CCUGGAGGCCGCCCUGGUCAAUGUGCUGGAGCCUGGGGACUCCUUCCUGGUUGGGGCCAAUGGCAUUUGGGGGCAGCGAGCCGUGGACAUCG

>random_seq_from_cds__NO_10986

CCUGGCCCAGCACAAGCCAGUGCUGCUGUUCUUAACCCACGGGGAGUCGUCCACCGGCGUGCUGCAGCCCCUUGA

>random_seq_from_cds__NO_10987

UGGCUUCGGGGAACUCUGCCACAGGGUACAAGUGCCUGCUCCUGGUGGAUUCGGUGGCAUCCCUGGGCGGGACCCCCCUUUACAUGGA

>random_seq_from_cds__NO_10991

GCACCGCGAGGCCGCGGCGUAUCUGCAUGGGCGCCUGCAGGCACUGGGGCUGCAGCUCUUCGUGAAGGACCCGGGCGCUCCGGCUUCCC

>random_seq_from_cds__NO_10999

UGGGCCACACGCCUGAGGCCGUGCAGAUCGCCACGGGAAGGAACGCCGCGCGCCUCUGCUGUGUGGCC

>random_seq_from_cds__NO_11000

AGCCGUGACGCCGGCCUCUCGUGGGGCAGCGCCCGGGACCUCACCGAGGAGGCCAUCGGUGGUGCCGUGCAGGGACUGGGCCAC

>random_seq_from_cds__NO_11006

GUCCACGAACCCCCAGAGGAGGCUGCUGUAGACCCCCGUGGAGGCCAGGUGCCUGGUGGGCCCUUCAGCCGUCUGCAGCCUCGG

>random_seq_from_cds__NO_11009

UGGACCCGCGCAGCUGGACAGAGCCCUGGGUGAUCUACGAGGGCCCCAGCGGCUACUCCGACCUGGCGUCCAUCGGGCCG

>random_seq_from_cds__NO_11010

GCUCCUGAGGGGGGCCUGGUUUUUGCCUGCCUGUACGAGAGCGGGGCCAGGACCUCCUAUGAUGAGAUUUC

>random_seq_from_cds__NO_11019

UCUCAGACAUGGUGGUCCCUCGAUCAACCAAGACUCAUUACUGAGGACAAGGAAGGGGGCCUUUUCACUGUGACUCUGUUUC

>random_seq_from_cds__NO_11030

UGUUUGUAGGAGCCACUGAUUCAGCCGUGCCAUGUGCAAUGAUGUUGGAACUUGCUCGUGCCUUAGACAAGAAACUCCUUUCCUUAA

>random_seq_from_cds__NO_11045

GGGAUUUUUGCUGAUACAGAAAAUUUCCCUUGGCAGUAUUCUGUCAGGGAGUUUGUGUGCUGGAAUAGCAACAGG

>random_seq_from_cds__NO_11059

ACUUUAUCCAACAGCUCGAUUGCCAAAGACCGUGUCAAAUGUUGUGUUACAAAAGGAUGCCUGUAAUAAUUUUUCAGCAGUAGGUCUUUUUUUCGGAUUUUUGGUAAGUG

>random_seq_from_cds__NO_11065

GCAAUUUGCAGUUCUGACAGAGGUCCAGUUACCGUGAUAAAUAUCCUGUAAAGAACCACCUCCACAAAACUCCAUGCAAA

>random_seq_from_cds__NO_11068

CCGAUGCGCUGAAUCAGCUCGAAGUCCUCCUGCGGGUUCCGGCGGGACAAAUCGAAGCCGGGGUUCAUCUCAUUUGUUUUUGGGCU

>random_seq_from_cds__NO_11071

GAAUCGGAGGUCAGUUUAGUUGGUGUGGCAAAUAUGAUAGGUGGUGCUUCUGUGGAAACCACAGGCUUUAAUCCCCUGCGGGCUAUAGGCCU

>random_seq_from_cds__NO_11074

ACCAGGAGGCUGUCAUCAGGGGCCAGGCUCAGGCGCCGUCCCCCCAUUGUCACCACCGAGGAGCCCUCCCAGCUGCCACAGCCACACGUC

>random_seq_from_cds__NO_11087

ACAUCCUUGGGCACCACAUCUCCACGGUACAGCAGGCAGCAGGCCAUGUACUUGCCGUGCCGGGGAUCACACUUUACCAUCUGGUUG

>random_seq_from_cds__NO_11089

UGACUGGUGCAUAGGUGGCCAGGGGGAAGUGGAUGCGAGGGUAGGGCACCAGGUUGGUCUGGAACUCUGUCAGGUCC

>random_seq_from_cds__NO_11102

GAAAUUUCUGAACGAAUUGUACAGUGGCCUGCAAUAGACGUACAAACCUCUGUUGGACAAAGCUGGUUUGGGAUCCAUAACU

>random_seq_from_cds__NO_11108

CUACAACAGUUUUUAAGGAGAUACUGCUUGGCUGUACUGCGGCAACUCCACCUAGUAAGGACCCAAGACAGCAAAGUACUCCCCAGG

>random_seq_from_cds__NO_11116

AUCGUGGCACAGUUGCCUAAAUGCCGAGAGUGUCGCUUGGACAGUCUCCGCAAGGAUAAGGAGCAACAGAAG

>random_seq_from_cds__NO_11121

ACUGGGUGUGUCCUCGGUGUGGGUUUGGAGUAUGUGUGGACUGCUACCGGAUGAAGAGAAAGAAUUGCCAACAGGGGUGCUGCUU

>random_seq_from_cds__NO_11125

GAAAAACCGACUCUUGGUGCAGUGCUCCAGCAGAAUCCCUCAGUGUUGGAGCCAGCAGCUGUGGGUGGGGAAGCAGCCUCCAAG

>random_seq_from_cds__NO_11140

UCAUUAAGAAAACGUCUUCAUCAAGAGUAUGGAGUUCAAGGCUGGGCUAUUGUACAGUUUCUUGGGGAUGUGGUGUU

>random_seq_from_cds__NO_11146

AAAAGGGGCCUUCUGUGGAUUGGGGAAAAAUCCAGAGACCCCCUGAAGAUUCGGAUUCAACCCUAUGAAAAGAUAAAGGCCAGG

>random_seq_from_cds__NO_11190

CCUGCUGCUGUAAUUGUGGAGAAGCCACUGAGUGUACCACCAGCCCAAGGACUUUCCAUUCCAGUGAUUGGCGCAAGAAGUUGA

>random_seq_from_cds__NO_11205

UUUCAGCCUCACUUGAUCAAUGUUGAAGGAGUUGGUGUUGCUGAAUUGCUUUUUAACACAAUUCAGGCAGCUGACAUUGAU

>random_seq_from_cds__NO_11208

GGUAUUCCUGGGUGGUGCAGUUCUAGCGGAUAUCAUGAAAGACAAAGACAACUUUUGGAUGACCCGACAAGAGUACCAAGAAAAGGG

>random_seq_from_cds__NO_11209

UGUCCGUGUGCUAGAGAAACUUGGUGUGACUGUUCGAUAAGAUGAAGUUCACAGAUUGUCUUUGCUGGACUUC

>random_seq_from_cds__NO_11217

UCCUGGGCUGCCCGACUGGCAUUGGGCUUCACAUUCCAAGAAUACCUCCUGUGUGAAUAGUCCUCUCCAGGGGGACCAGGAGGAGGGA

>random_seq_from_cds__NO_11226

AAACGAGUCCCAGGCAUCCGCUGUCCAACUGGGAGAUAGUCUUUCCACCCUUUCGGGGAUGUGGUUUCCGCCCUUCUUCUUGGC

>random_seq_from_cds__NO_11258

GUUAUAGAAGGUGAUGGUGGUCUCUGCUCUUAAUAAGGCCUGGUGCGUGAACUGCUUUGCCUGUUCUACCUGCAACACUAAAUU

>random_seq_from_cds__NO_11261

UCAGUUUGGCCUCUACAAAAAUCCGUUUGAGGAAAUCUCGAGUAUAGUCUGAGACAUGAGGUGGCAGCUUUGGGUUUGUUGGCU

>random_seq_from_cds__NO_11291

AUUCGCACUGAGUUUGACCAGGAGAUUGACAUGGGCUCACUGAACCCGGGAAAGCAACUGGUUUGAGAAGAUGAUCAGUGGGAUGUAC

>random_seq_from_cds__NO_11294

GGAGGACUGCGUGGCCACUCACCGGAUCUGCCAGAUCGUGUCCACACGCUCCGCCAGCCUGUGCGCAGCCACCCUGGCCG

>random_seq_from_cds__NO_11299

CAAGGAGACUCAUGCCAGUGCCCCCGUCAAGAUGCUGCCCACCUACGUGUGUGCUACCCCGGACGGCACAGGAGAAAGGGGA

>random_seq_from_cds__NO_11312

CAGCUUUGCCAAAGUCAUGCAUGAGACAGUGAAGGACCUGGCUCCGAAAUGUGAUGUGUCUUUCCUGCAGUCAGAGGAUGGCAG

>random_seq_from_cds__NO_11314

AGCGGCUGGAAGCGCGGGGCGGCCGGGAGGGGGCAGUCCUCGCCGGCGAGUUCAGCGGACAUCCAGGC

>random_seq_from_cds__NO_11318

UGGGCGGGAAAAGGUGUGAGCGGUACUGGGCCCAGGAGCAGGAGCCACUGCAGACUGGGCUUUUCUGCAUCACUCUGGAUAAAGGAG

>random_seq_from_cds__NO_11322

GGCAGCUGCUCCUGACCCAGGAUGAUCCCACCUGACUUCAGUCUCUUUGAUGUGGUCCUUAAGAUGAGGAAGCAGCGGC

>random_seq_from_cds__NO_11342

UCAAAUCCUCCCAUUUUGUUGGUCUGUUCCUUGCAUGCUAGUUUAGAGGAUCUUUAAGCAAGCGACAAUGAAGGCAUUGCGGAAAAAAUCUGU

>random_seq_from_cds__NO_11354

CUUGGGAGUCUGGUUUUGAAUCUGUUGGAUCUAUACUCUGAAGUACCUGCAGGGCUCUAUCCAUCUUAUCCCUCAUCUAUA

>random_seq_from_cds__NO_11384

AACUGCAUUUGUUAAUAGCUGCUAUAUUAAAAGUGAGGAGGCCAAACGCCAACAACUUGGGAUAGAGAAAGAAGCUGUUCUUC

>random_seq_from_cds__NO_11387

ACGUGGCUAGAAACUUGGCUGUGGAGCAGUUAACACUGAGUGAAGAAUUCCCAGUGCCCCCAGCUGUGUUACAGCAGACUUUCU

>random_seq_from_cds__NO_11394

GUCCGGCCUUUAGAGCGCUCCCAGCGAACACAGUCCCGAGUCCUGCGGGGUGGGGGCCCCUGCCAGCUGCCAGGCCCCUU

>random_seq_from_cds__NO_11396

UGGCAGUUCCUGGGUGUCACGGGUACCUUUAGGGGCGUGGCACUCCCCUCCCUUUGGGUGCCUCCGUUCGGGCUGUCGCC

>random_seq_from_cds__NO_11397

AGGGACCUCGACUGGGCUUGGGGGGAUCUAGCAUAGCUUUCUGGGUUUCGCCCAACCUUUGCUGAUUUGACCUGGUCCCUGGAA

>random_seq_from_cds__NO_11407

CAUGGGGAUACCUCUGCUGGGACCUGAAGCACCAGAGUGAAGCAGACAUACUGUGAAUCCUGGUCCUCUGCAGUGGCAGGAUGCAAAGUGAUGUAGAUCU

>random_seq_from_cds__NO_11421

GAUUUUCUGUAGCUGCCACUGACAUUAAUGGAGAUGAGUUAUGCAGAUGUGUUUAUUGGAGCACCUCUCUUCAUGGAUCGUGGCUCUGA

>random_seq_from_cds__NO_11441

UUGGGGUUGUGGAGUUGCUCAGUGCUUGAAGAUUGUCUGCCAAGUUGGGAGAUUAGACAGAGGAAAGAGUGCAAUCUUGUAC

>random_seq_from_cds__NO_11449

CUGAAGGGAGCCAGAUGGGGGGCUGGUUGUGCUGAGUGGAGGGGGCACCUCUGGCCGGAUGGCAUUCCUCAUGUCGGGUGUCCUUUAAU

>random_seq_from_cds__NO_11450

CAGCUGAUGAAAGGUCUGGGACAGAAACCUCUUUACACCUACCUCAUUGCAGGUGGUGACAGGGUCUGUGGUGGCCUCUAGGGAG

>random_seq_from_cds__NO_11464

GGACAGUCCAAGGCUCGAUGCAUCGAGAGCCUCCUCCGAGCGAUCCACUUUCCCCAGCCACUGUCAGAU

>random_seq_from_cds__NO_11481

AAGAAACUCAUCCUUCAUGCUCUGGAGCAGUGUGUUAAAGUCGACACCGUCUUGCUCUGAGAAAACAUCACCAAAGAGGGA

>random_seq_from_cds__NO_11515

UGUUUUCCUUCAUGAAAGGUCGCUAGAGAAGUCCGACUCUUCAUCUUUGAUGGAGUCGUAACAAGAAUCACAAACCCG

>random_seq_from_cds__NO_11537

UUUUUUUGAUCCAGUUGAAAGUGAUGAAGACAUAACAAAUGUUCAUGAUGAUGAGCUGGAUUCAAACAAAGAAGAUGAUGAAAUUGCUG

>random_seq_from_cds__NO_11546

AAAGUUGUGUCAAAUCUGCCAGCCAUAACCAUGGAGGAAGUAGCCCCAGUGAGUGUUAGUGAUGCAGC

>random_seq_from_cds__NO_11560

AACAUGCCCCACAAGUUCGGUAUCCACAACUACAAGGUCCCUACCUUCUGCGAUCACUGUGGGUCCCUGCUCUGGGGACUCUUGCGGC

>random_seq_from_cds__NO_11565

UGGGCCUGGAUGAGUUCAACUUCAUCAAGGUGUUGGGCAAAGGCAGCUUUGGCAAGGGUCAUGUUGGCAGAACUCAAGGGCAAAGAUGAAGUAUAUGCUGUGAAGGUCUU

>random_seq_from_cds__NO_11577

CGAUGAAGGCCAGGGAUAAAUUUCAAGAGUUGCUUUCGGACACUUCCCUUUCUGCUCUUCCGGGGCAGGGUCCCAAACAUGCUUGAUAAGUUGCCCU

>random_seq_from_cds__NO_11585

GCUUGCAGGCCAGCACAGUGAGCUGGGCUGUCCUCCUGUAUUUUGCAUAUCAAAGUGAACAUUUCCGAGGAGCAGGCAUUCUGAUUCUGGGGCCUGUAAGACCUGAAUUU

>random_seq_from_cds__NO_11595

UCUUCAUGGUCAUGAUGUCCCCAUAGCAGCCGUGGUACAGGCGUGGAUGUAGGUGAGGCUUCAUUGAGUGACUGAAGG

>random_seq_from_cds__NO_11607

UCCCUAUGAUCCAACACCAUAUUGAAGCCUGUGCCCAGGGUCCCUGGAAUGCAGGAGAUUCUGCUCAUUGGCUUCUACCAACCUGAUGAG

>random_seq_from_cds__NO_11610

UGCUCCGACUUCCCCUUGAGUGCUAUGUUGGAAGCCCACCGACGCCAGCGUCACCCUUUCUUACUCCUUGGCACUACGGGCUAACAGG

>random_seq_from_cds__NO_11613

AAUCAGCAGGAUGGGCAAUUGGGAGGACUCACCAGGCUUGUGGCCAGGGGCAGGUACCAUCCGCCUAGAGCA

>random_seq_from_cds__NO_11616

UCCGAGGGGAAUGUGUACAUCCACCCGACCGCCAAGGUGGCCCCCUCGGCUGUGGCUGGGCCCCAACGUCUCCAUCGGG

>random_seq_from_cds__NO_11637

ACAUACGCAGGGUAAGUGGUAUCGAUAAUAAAUAUCCGAACAACGGGAUUCUUAGCUCCAGCCCUUUGGGUAUGGAAUAUUUAUUGUUCUAGGAUAUUGUUCAUCGCCAU

>random_seq_from_cds__NO_11639

UUCAUAAACCCAGUCUGGGAUUCCAUUAAAUAUUUUAUUUUCUCUUCCAUUAAAUGUUAUUUGAAAAGGUGGAUCUCCUGGUCUUUGUUUCAAA

>random_seq_from_cds__NO_11642

CUAGAUAUACAAAUUGCCGAUCAGGUGAUAAGCCGUAAUUUGAAGCAUUCACACUUUUCCAUGGUUCUAUUACUCAAAAUGGUAUAUGAUUGUCC

>random_seq_from_cds__NO_11648

GACGAGGAAUGCCUCCCGACACUGCUGGUAAAAAUCUUGAAAAGAAAGGCCCAAUGUAAGAAGGGUUAUCCUGGUUGUCCAG

>random_seq_from_cds__NO_11659

UUUUAUCUCUUUUCAUUCUCUUAGAAGGUGUUUCUUUGCCAACAGGAGCUGAAACUACUCUAUUCUGAGCUUGAAUCUUUU

>random_seq_from_cds__NO_11667

GGUUUACUCAUGAUGGCGGCCUUCUCCGGAGAUGGGUGUAAUGCCUGAGAUUGCACAAGCUGUGGAAGAGAUGGAUUGGCUGCC

>random_seq_from_cds__NO_11679

UAAGAAAUACAUUGAUAAUCCUAAAUUAAGGGGAGCUUCUGAUAAUUGGAGGUGUUGCAGCCCGGGAUCAGCUCUCUGUUUUGGAAAA

>random_seq_from_cds__NO_11681

AUCUCAAGUUAGAUUCCUGGUCCUGGAUGAAGCUGGAUGGGCUUCUUUCUCAAGGUUAUUCUGAUUUUAUAAAUAGGAUGCACA

>random_seq_from_cds__NO_11697

CAAGCGUGAGGCCCUGUAGCGGGGUCAGGGAAGCCAUUGCAUCGGACCACAGAGGCGUAGAACUUCGCUGAUGCUUUGGGGAUCCUUGGCAGAGAAGGGUCACUGUAGUU

>random_seq_from_cds__NO_11702

AUAGUUGAGGUUGUAGGCGAGGACAGUGGUGUAGUGAUUGAACCCAAAAAAGUCAUAGGUGCCGUUGAUCCUCCUCUUCUCACUCUCUGUAAAUUCUGG

>random_seq_from_cds__NO_11713

UCAGGAAACCGUCCGUACAGAAACUCAUCCUCCCUGGCCAGUGGCAUGCCGUUGUUGGUAAUGACCUCUGUGUAGUACCUGGCGGAGGCU

>random_seq_from_cds__NO_11716

GGUUUUGUGGUAAAAUAUCCUAUCAGUAUCCUCCGUGUUCGGAUUGGUCAGCCCCACUCCGUUUUCGGUGAUGUAAAUGGGGAUGUCA

>random_seq_from_cds__NO_11721

CAUUCGGUCAGCGGCUUCCACAUCUCUGGGGACCCCUGGUGACUUGGGCUCUGCCCAGUGUGUACUGAGGCUCAGCGAGAUGAC

>random_seq_from_cds__NO_11731

AAAGUGAAGGCUCUGACUUUGGAGGGGAGGUUUACUGUAUUAGGUGGUAGCAGUCUUUUUGCCCCCUUGGUGAGGAAACC

>random_seq_from_cds__NO_11732

GUUCUUUUCUAUGAUGCUAGUGAAAAAGUAGGCAGAUUUCCUGGGAGUCCUUGACUUGCUGCUGUCGCUGAAGUUGAC

>random_seq_from_cds__NO_11742

GGACCAAGUGAGCCACCCUUAAAAGAGGCCACUCCUGGGUCAGAGAUGCCGGGAGGGUGCUGGCCGGUGCC

>random_seq_from_cds__NO_11744

CGCUCUCAUUCUGCCAUCCACCAUGAUCCUGCAGGGCCUGAGGCAGGUCCCAGUGGAACAGCGUGGCCAUGGGC

>random_seq_from_cds__NO_11757

UGGUGCAGGAUGACCAUGGGCUGAAGCCGUGCAGUCUUGAGGGCCUUGAGGAGUCGCCGGUAGCACUGCACUGUUUUCUCGUCUGGAUUCUGGGUGC

>random_seq_from_cds__NO_11758

UUCCUGCUGGGAGGAGCUGUGCCCAUGACAGAAAUACCUUAUAAUGGGUGAUCUGACUGGCAUGGAGACUGCUGAAGUAUUCUGGCAGG

>random_seq_from_cds__NO_11767

UGUUGUAGUCUGCAUUUUGGGAUGCAUACUUAUUUGUUGGCUUACAAAAAAGGAAGUAUUCAUCCAGUGUG

>random_seq_from_cds__NO_11773

GCGGGAACAGGGGUCUCUGCUGGGGAAGCUGGUCCAGAAUGGAACAGAACCUUCAUCCCUGCCAUUCUUGGACCCCAAUGCCCGCCCCCUGG

>random_seq_from_cds__NO_11774

UACCAGAGGUCUCCAUUAAGGACUCCACGGGUAUUCAAUACAGGGGGUGCCCCUCGGAUCCUUGCUUUGGACUGUGGCCUCAAGU

>random_seq_from_cds__NO_11775

AUAAUCAGAUCCGAUGCCUCUGCCAGCGUGGGGCUGAGGUCACUGUGGUACCCUGGGACCAUGCACUAGACAGCCAAGGAGU

>random_seq_from_cds__NO_11783

AGACGUUGCUGAUCAACCCCAAUAUUGCCACAGUGCAGACCUCCCAGGGGCUGGCCGACAAGGUCUAUUUU

>random_seq_from_cds__NO_11796

UGGACCGCCUGUAUGAGCUCACACGCAUCGACCGCUGGUUCCUGCACCGAAUGAAGCGUAUCAUCGCACAUGCCCAGCUGCUAGAAC

>random_seq_from_cds__NO_11806

CCCUCCUAUGUGCUGAGCGGUGCUGCUAUGAAUGUGGCCUACACGGAUGGAGACCUGGAGCGCUUCCUGAGCAGCGCAGCAGCC

>random_seq_from_cds__NO_11811

GGGUGUGGACCUAGUAGCCUUGGCCACGCGGGUCAUCAUGGGGGAAGAAGUGGAACCUGUGGGGCUAAUGACUGGUUCUGGAGUC

>random_seq_from_cds__NO_11813

GAGGUGGCCGGCUUUGGGGAGAGCCGCUGUGAGGCAUACCUCAAGGCCAUGCUAAGCACUGGCUUUAAGAUCCCCAAGAAG

>random_seq_from_cds__NO_11820

GCCCUGGCUGGGGGUAUCACCAUGGUGUGUGCCAUGCCUAAUACCCGGCCCCCCAUCAUUGACGCCCCUGCUCUGGCCCUGGCCCAGAAG

>random_seq_from_cds__NO_11829

CCUAUGUGGAGGGUGGAUCUGGAGCAUGAGUGGACAAUUCCCAGCCACAUGCCCUUCUCCAAGGCCCACUGG

>random_seq_from_cds__NO_11834

AGGUAGCCGAGCCAGGAGCUGAUGGGAACCCCUGAUGGCACCUGCUACCCUCCACCACCAGUACCGAGACAGGCAUCUCCCCAG

>random_seq_from_cds__NO_11835

AACCUGGGGACCCCUGGCUUGCUGCACCCCCAGACCUCACCCCUGCUGCACUCAUUAGUGGGCCAACAUAUCCUGUCCGUCCAG

>random_seq_from_cds__NO_11838

UGUGCUCAGCUUCUCGGAAGCCACAUCGUCCGUCCAGAAGGGCGAAUCCCUGGCUGACUCCGUGCAGACCAU

>random_seq_from_cds__NO_11839

GAGCUGCUAUGCCGACGUCGUCGUGCUCCGGCACCCCCAGCCUGGAGCAGUGGAGGCUGGCCGCCAAGCACUGC

>random_seq_from_cds__NO_11847

GGAAGCAGGAGGAGAAGCACCUGAAGAUGCUGCGGGACAUGACCGGCCUCCCGCACAACCGAAAGUGCUUCGACUGCGACCAGC

>random_seq_from_cds__NO_11889

GUCACCGAGGACGAGGUCGAGUGGAUGAGACAAGGCUGGACUACUUACUACUGAUCCACGUGUGAGGGAACGGAAGAA

>random_seq_from_cds__NO_11890

GCCAGGCCAAGAGGGAGCCCGCAGAAAGUUUACGUGGAAGAAACGCUAAGUUAGACUGUGGCGCUCAGCAUGGCCUCCGUCUC

>random_seq_from_cds__NO_11897

GCAGCUUCUCCAGCACAGUGGGGUGCUUCAGCAGCUGCAUGAUGAGUGAGGUGCUGGCGCUGGCCGUGGUGGCAUAGGCCGCAAAG

>random_seq_from_cds__NO_11902

GGCCUCGGGGUGGCUGCUCCAGGCGCGCAGUGUGUCCUGGAUCACCAGCUGGAUCUUGGGCAGGUAACUCU

>random_seq_from_cds__NO_11906

UGGCCGGUCUCUCCGAUGAGCGGGAAGCCCAUGGAUCCCUUGGGGAUGGGCAGCUUGCAGCUCUUGUCGCGAGUGGCGGCCCAGCGCAG

>random_seq_from_cds__NO_11911

AUGAUAGCCAGAUUUCUUCUGCAUGGCGGUUACAGGGCAAUCUUUAUGAGCCAGAAGAAGCUGUUUCAGCUGUGCCACUUCAUUU

>random_seq_from_cds__NO_11914

GUUAGCUGCUCUUCUCCGACGACCACUUGUACUUUGGGUCUGUGGAGUUGUGUGAGCUGGAGAAGCCCGGAG

>random_seq_from_cds__NO_11915

UUUCUGUAGUGGAUGUGGCUGGCUGUUGUAAUGACUGCGGUCGAGAUUCCUCUGACUGAGUCCUAACCAAUCCGCUACCAUGACCUUUGACAGUAUC

>random_seq_from_cds__NO_11916

ACCAUUGGUAACUGGAGGAUGUUGCUGGGUCAAAGCAGCUUUUAAUCUCCAUUUUUGCUUCUGACUGUACU

>random_seq_from_cds__NO_11917

GGUUGGGGAGAGGAAGGACCUGGGAUUCCUGGAACACUAGGCACCAUGGUGACUGGUCGAACGAGCUG

>random_seq_from_cds__NO_11924

AAUCUUGUUGGUGUUGGGGUCUGAUCCAGCCACAAUGACACUGUCAUUACGUGCUGGACCAAAUUUCAGUGUCAUCUCAUGU

>random_seq_from_cds__NO_11937

AAGCACAGAAACUUGGUAAUGGGAUCAACAUCAUUGUGGCCACACCAGGCCGUCUGCUGGACCAUAUGC

>random_seq_from_cds__NO_11940

UUGAAGACCUGGCAAGGAUUUCUCUGAAAAAGGAGCCAUUGUAUGUUGGCGUUGAUGAUGAUAAAGCGAAUGCAACAGUGGAUGGUCUUGAACAGGGGAUAUGUUGUUUG

>random_seq_from_cds__NO_11950

AUCAUUCCAGGCCUGAAGUGAGGGAGCCUUUAUAUUGCCACAGUGUUCAUUGUUCCCAGCAUUGUUGGGCUCACCUGGAAUCCAGAACCC

>random_seq_from_cds__NO_11956

CCCAACUUCUUGUUAAGAUCUGGAUCUGUGCGUUGGCCUUCUCCACACUGGUUUUUAACUUCAGGAACUGAGAACGCACAUAACCCAGGCUCUCAUUCA

>random_seq_from_cds__NO_11964

GAAAAGCUAUCCUCACAUCAUUCAGGACAAAUGUACCCUUCACUUUUUGACCCCAGGGGUGAAUAAUUCUGGGUCAUAUAUUUGUAG

>random_seq_from_cds__NO_11983

CGGACCUCUUCACCACGCUGGUGGACCUCAAGUGGCGCUGGAACCUCUUCAUCUUCAUUCUCACCUACACCGUGGCCUGGCUUUUCAUG

>random_seq_from_cds__NO_11997

CCACCAAGAUGUUAUCUGAUCCCAUGAGCCAGUCUGUGGCUGAUUUGCCACCAAAGCUUCAAAAGAUGGCUGGAGGAGCAGCUAGGAUG

>random_seq_from_cds__NO_12001

GUUAACAAGCUCGCUCCUCUUUAAUGCUGACUCGUCCUCUUCCUUCUUCCACCUUUCUGAGGUGAAGCACAAUAAGGUUAGAGAUUCGGCAGUACUCAGAG

>random_seq_from_cds__NO_12009

UCGGGACAUGAUGGGAGCUGACAAAUUUAUAUUCUGUUUCAAUGAUUUUGAUCUGUCAUAGUGUCCACUGAUUGGGUUUGCUGCUGCCAAAAUGG

>random_seq_from_cds__NO_12018

UCGAAUGCCUUCUGUCUCAUAUCCAUCAACACCACUGACACGGGAAUUAGUUUCUGCACGUGCUCCCUGGUGUGCUAAGCUUGGAGAC

>random_seq_from_cds__NO_12027

UUCUGGCACUUCUCGGCCACCUCGUCGCGGACCUCCAGGUGCUGGCUGCCGGCGCCCGGCUCCGCUGCCGCCGCGAGGUCCAUUUCAGCAUAUGG

>random_seq_from_cds__NO_12047

UAUCAUUUCCAAACAUCAUGGACUGGUCCCUGCAGAUCACAUUCACCUCCCUGGUCACAAAUAGGACAGUCCAAUGGGUGAUUU

>random_seq_from_cds__NO_12061

GGUGGCGAGGGGCUUCCCAUCUCCUGUGGUACUGUCUGGAUAAUGAAUCCCCGCUUCAGGAAAGUGCUUGAAGCCCUAGUUUCU

>random_seq_from_cds__NO_12073

ACGUGGCGCGUCAGCUGCACAUCUCCCUGCAGUCUGAGCCUGUGGUGACCGAUGCGUUCCUGGCCGUGGC

>random_seq_from_cds__NO_12075

CAGGCCCAGCCUGCCAUGGUCCACGCCCUCGUGGACUGCCUGGGGGAGUUCGUGCGCAAGACCCUGGCAACCUGGCUGCGGAGAC

>random_seq_from_cds__NO_12096

CUUCCUGCUGGUGUGGCUGGUGGUCUUCUGUUGCAGGAUUAUGGCCCUGGCCGCCGCGGCCCUGCUCCCCACCUUCCACAUGGCCUC

>random_seq_from_cds__NO_12112

ACGUCCUCUCUACAAGGAAUAUUGUCCUCUCUGGAGGUUCAACCAUGUUCAGGGACUUUGGACGUCGCUUGCAAAGAGAUUUGA

>random_seq_from_cds__NO_12137

CUUAGUGCAGAGAAGAAAGCUGGGGGCUGGUCAAGGUUUGCAAAUAGGUAUAUUUUUAACCAAUACUGAUCACUUUUAUUCCAUUCUUCUUU

>random_seq_from_cds__NO_12146

CGGAGCCGCCUUGGCCGGCCCGGGCCCGGGGCUGCGCGCCGCCGGCUUCAGCCGCAGCUUCAGCUCGGACUCGGGCUCCAGCCCGGCGUCCG

>random_seq_from_cds__NO_12181

CUGCUUUCCCACUCAUCCUUCACAUGGAGAGUCUUGGAAGCCAUUUCUAAUAGAUGUUGAUGGUGGAGGUGGGGGAGGUGC

>random_seq_from_cds__NO_12184

CGGGGGAGGAAGAGGACCUGAACGUCCUGGCGAAGGUAACGGGGGCGUGGACGAACUGAGGGACAGAUUCCGCUGUGGGAGU

>random_seq_from_cds__NO_12186

GGAAGGCACUGGAGGCUUGUUGUUCUGAGGAGGAGGAGGGGGAACCGCUUCCCUGUGGAUGGAGGGCCUGUUGCCCACUGG

>random_seq_from_cds__NO_12189

GGCUGCCUGGGGCCUCCGGGCACUGGUGGGGACCCCCGGUUGUGCGGACUUGAUUGAAUGGGUCUUGGAGUACUAGGUACUGGAGGAGGAAUGCUAUCAGGCUUUGAGCC

>random_seq_from_cds__NO_12191

CCUGGGCCACUUGGGGGUGAAAAGGGUUUCGCAGAUGUGGAUCUUCCUCCCGGUGGCAACAAUGGUGGUCGGCUUCCUCCAGAAUCCAUUAUCCCUGUUGGCCGUGGAUC

>random_seq_from_cds__NO_12196

GCUGGGGGCGUCGCUCCACGUCUGGGGUUGGCUAAUGCUCGGCAGCUGCCUCCUGGCCAGAGCCCAGGCUGGAUUCUGAUGGCACCA

>random_seq_from_cds__NO_12200

ACAUGGGCCAAUUAUUCAGACUGCCUUCGCUUUCUGCAGCCAGAUAUCAGCAUAGGAAAGGCAAGAAUUCUUUGAACGCCUCUAUGUAAUGUAUA

>random_seq_from_cds__NO_12204

GUUGUGAUGUUUAUUUACUUCCUGGCUACAAAUUAUUAUUGGAUCCUGGUGGAAGGUCUCUACCUGCAUAAUCUCAUCUUUGUGGCUUUCUUUUCGGACACCAAAUACCU

>random_seq_from_cds__NO_12208

GUCCUGGUCCUAGUCUUUGGAGUGCAUUACAUCGUGUUCGUAUGCCUGCCUCACUCCUUCACUGGGCUCGGGU

>random_seq_from_cds__NO_12227

CAUGGUGGCCCGUGUCUCUGUGUCAUGGGAGUGCAGCAAGGAGGCACUCAAGCGCCCAUUAAUCAGCU

>random_seq_from_cds__NO_12234

CGCUGGGGCUCCUUGUCUGUCUCUGUUGCCCGGGCAGGGGAAGGGAGCUUCUUGGAGUCGGCCUCUGGCA

>random_seq_from_cds__NO_12244

UCGAUAUAAGCGGAGAGUGUCAUGUAGAUUUUCUCUCGAUAAGGGGUGACCCGGUUCAGCAGGAGAGAGUUGUGCAUGGAGC

>random_seq_from_cds__NO_12265

CCUCCAGCUUGCUCUCAUAGUCCCAGCCGCUGCUGCUCCAGCAGGUAGGUGGCCUCCUCCCGCUCGCGGCGGUACUGGUCCUCCAGUUCCUGGAGCCCU

>random_seq_from_cds__NO_12297

GUCCCACGCCUGCGGCACAUCAUCACUGUUGAUGGAAAGCCACCGACCUGGUCCGAGUUCCCCAAGGGCAU

>random_seq_from_cds__NO_12344

GAGGUCUUAGAGUAAAUGGUAUUCUCUUUUCAUGGUCAUCAGGCUCACUCUCAGGGUCAUCUUCGCUCUCUCUCUUCA

>random_seq_from_cds__NO_12347

AAACCCUGAUCAUCCUUACUUUCUGAGGAGUGAUGUAUUUUAUCCUCUUCAUCUGUGGACACAUCAAGGGUUCUGGAUUCCU

>random_seq_from_cds__NO_12348

GAGCCUUCGUCCGCGCGGGGAGCCGCUUUGGUGGCACGCCGGGAGCUCGCCCAGACCCGGCCCCGGCCACGAGGGCCCCGAACCCGGUGGGGCAGUCC

>random_seq_from_cds__NO_12351

CAAAGGGUACGACGAUCUACCCCAUUACGGGGGCAUGGAUGGAGUAGGCAUCCCCUCCACGAUGUAUGGGGACCCGC

>random_seq_from_cds__NO_12367

UAAUGGCCUGGAGCCAAUCGCCCACUGAUACCACAGCAGAGAAUUCAGGGGAGCUUGGAUCCAACAAGGCAGUGUUAGGUCCU

>random_seq_from_cds__NO_12376

CUCUCUCUUGCCAGGCACUUUGAGACGCCCACUGCAUACCUCACCAAAUUCACCCAACUCCUAUAACUUUUUCAAUCUUAAUGCAGGAUGCGUCAAUUUCUUUGGCAAAC

>random_seq_from_cds__NO_12392

GUAAAACCCCUUUUUGCUUAAUGGCCCUACAUCCCGGAUCUCGGUGUUCAGCUUCAUGAUUCUGUCACCAAUGUCCACUUGGGUGAAGCUCUCAUCAGCAGCAAUGGUGU

>random_seq_from_cds__NO_12410

UACUUCUCCAGCUUUCUCCCAUCUCUGAGUGAUGAGGAAUCACUUCUUCUUGGAAAAACUUCCUUACACUUUUCCGGAAAAUGUCAUGCUCUGGAGAAAAGA

>random_seq_from_cds__NO_12426

CAAGAUCUCAAAUCAAGGCCGGAGUCUGUGGAAUGUAUCAGGGUAUAAUUUCCGUGGGUUUCGCUGGUUACAGGCUAUGAUAUU

>random_seq_from_cds__NO_12433

CUCUGAUGAGGAAGAGAUCCAGCAUGUGGUAGAGGUGAUUCAAAAUUCCACGGCCAAAGUCAUCGUGGUUUUCUCCAGUGGC

>random_seq_from_cds__NO_12439

ACUUAGCAGUCUACUCCAUUGCCCACGCCUUGCAAGAUAUAUAUACCUGCUUACCUGGGAGAGGGCUCUUCACCAAUGGCUCCUGUGCAGACAUCAA

>random_seq_from_cds__NO_12470

GGGGAGUGGGAGCUCUUGGGCAUCAACAAGGCCACCCCAAAGAUGUCCAUGGGCAACAACCUAUAUGACCAGA

>random_seq_from_cds__NO_12473

CUUCCUGCUCAUGAUGAAUGACUUGCUCCCUGCCAGUGGCACCCCCCUCAUCAGGUGUCUACUUCGCCCUGUGCCUGUCCCUGAUGGUGG

>random_seq_from_cds__NO_12478

UCCUCUGGAACACCUAGAAUGGCUUCUGUUUGGCAGAGAUUGGGUUUUUAUGCCUCUCUUCUGAAAAGACAGCUAAAUGGUGGGC

>random_seq_from_cds__NO_12482

AUCCUUCUGGUAAGCUGCACAUGGGCCAUGUGCGUGUCUACACCAUCAGCGACACCAUAGCACGGUUCCAGAAGAUGAGAGGGAU

>random_seq_from_cds__NO_12486

ACUGGGACCCAGUGGAUCAAACAGUGCUUGCCAAUGAGCAGGUGGAUGAACAUGGCUGUUCAUGGCGUUCUGGAGCAAAGGUGGAACAGA

>random_seq_from_cds__NO_12494

CACACCAAUCCCCAUUGUCCACUGCCCAGUCUGUGGCCCCACACCUGUGCCCCUGGAGGACUUGCCUGUGACC

>random_seq_from_cds__NO_12559

GGGAUGUCCGUCCAUUGAUACAAACCAAAGAUGGAGAAAAUUACGCUACAGUGGUUGGGUACAGUGCUUUCUUACAUUGCGAGU

>random_seq_from_cds__NO_12562

AUAGGAAAAACUGCAGUCACAGCCAAUUUGGAUAUUAGAAGAUGCUACAAAACUUAGAGUUUCUCCUAAGAAUCCUCGUAUCCCCAAAUUGCAUAUGCUUGAAUUACAUU

>random_seq_from_cds__NO_12567

AACAAAGAAGAGCCUGGAAGGUGGGAGGAACUGACCAGAGUCCAAGGAAAGAAAACCACAGUUAUCUUACCUUUGGCUCCAUUUGUGA

>random_seq_from_cds__NO_12585

UAUUGGUGCCUACGCUGGAUCUAAGGAGAAGGGAUCUGUUGAAAGCAAUGGAAGUUCUACAGCAACUUUUCCCCUUCGGGCA

>random_seq_from_cds__NO_12591

AGAAGGUCAAGGUGUGGUGCUUCUCUGUGGCCCACCGCCACAUUUUGGAGGAUUUAUCUUAUGCAUGGACCUUCAAUGAUAACCCCUUAU

>random_seq_from_cds__NO_12611

UGAACUCGUCAUUACGUGGGAGGUCAAUUCCAGAAGAACUGCAGAAUGGGGAGGGAUUUGGAUAUAUCAUCAUGUUCCGGCCAGU

>random_seq_from_cds__NO_12619

CAUCAGCUGAGCUUCUGGUUCCAUUUGAAGAAGACUACUUAAUUGAAAUAAGAACAGUCAGUGAUGGUGGAGAUGGAAGCAG

>random_seq_from_cds__NO_12620

CAGUGAGGAAAUUAGGAUUCCAAAAAUGUCAAGGUUUGAGUUCCAGAGGAAUUCAAUUCUUAGAACCUAGCACCCAUUUUC

>random_seq_from_cds__NO_12627

CUCGCGCUGCCGUGGACUUUUGGAGAAUGAGUUGAAACUGAUGGAAGAAUUUGUCAAGCAAUAUAAGAGCGAGGCCCUCGGCGUGG

>random_seq_from_cds__NO_12630

CCCACCUGCUUUGUGUGUGCCAAGUGCUCCGAGCCGCUGGUGGACCUCAUCUACUUCUGGAAGGAUGGUGCACCCUGGUGCGGCCGCCAU

>random_seq_from_cds__NO_12649

CUGAUGGCUCCCAGUACUAUGUUCUGCUCAUCAUCACUGAUGGGGUCAUCUCUGACAUGACGCAGACCAAGGAGGCCAUCGUCAGCGG

>random_seq_from_cds__NO_12650

CCUCCUCAUUGCCCAUGUCUAUCAUUAUCGUCGGUGUAGGACCAGCCAUGUUUGAGGGCAAUGGAAGAGUUGGACGGUGAUGAUGU

>random_seq_from_cds__NO_12665

CCGUUGCCUGCAGUCACCCUCUCGUGCUGUGGAUUGUGCCCUGUGCCCCAACAAGGGCGGUGCCUUCAAGCAGACAGAUGACGGGCGC

>random_seq_from_cds__NO_12671

GAUCCUGGCAGAGAAGCGGGCAGCAGCACCUGUGGUGUCAGUGCCCUGCAUCCCACCACACAGGGCUUAGUAA

>random_seq_from_cds__NO_12678

ACACCAUCUUCUACCGGGCAGCAGUGCGGCUUCGUGAGCAGGGUGGUGCUGUGCUCCGCCAGGCCCGGCGCCAGGCAGAAAA

>random_seq_from_cds__NO_12696

GGGCUCUGCAGCACCGCAGCAAGGUGCAAGGCGAGCAGAGCAGUGAGACCAGCGAUAGUGAUUGAGAUGGACUGCCACUCUCCGCCCCUACCUGAGUGCCGUGCGGGCCA

>random_seq_from_cds__NO_12699

UGUGAAACAGGGCUGAUGAGAUCGAGAAGAUUUUGUGCCACAAGUUCAUGCGCUUCAUGAUGAUGCGAGCAGAGAACUUCUUUAUCCU

>random_seq_from_cds__NO_12714

AGUACCUGCAUGGUCUGGAGAUCAUCCACAGCAACGUCAAGAGGCUCUAAUGUCUUGCUGGACCAAAAUCUCACCCCCAAACUUGCU

>random_seq_from_cds__NO_12731

AGCUGGAACAUCUGAAACUUGGUCUCCUGGACAUGCAGGUGUUUGUAGAGGCUGCUGCCCUCGCACCACUGGGUCACAAUUGCCAGGUU

>random_seq_from_cds__NO_12737

UGUGACUUCGAAUUGCAUCCCUCAAUCAUCCUGCUGUCCACAGGCAGGGUGGUGCUGACCAUGUGGACAUUAGGUGUGGAUGUCGACC

>random_seq_from_cds__NO_12747

CCUGUAUGUGCUCCAUCCUACUCAUUUUCUUCACUGCGCAGCCUGGCAUUGGGGUUGGUGACUCUGAUGGCCAGUUGGGCAGCUCUUUCCACGAUGGCUUUGCGGUUCUU

>random_seq_from_cds__NO_12755

AGAUCAGCUGCCAGUUCAUGCUGUGCCCGGAGCUGCCGCCCAACUGCAUCGAGGCUGUAGUGGUGGCUGACAGCUGCCCACAGUGCGG

>random_seq_from_cds__NO_12762

CUGGAUGGGCUGCCCACUACAGCCCCAGCCUGGACCCAGUCUUCCUAUCCAGGAGGAGAGGGCAGAAGCUGGGGCAAGGGCAGAAGCUGG

>random_seq_from_cds__NO_12764

UGGGCAAGGCUGCUCUCGUCCCAACUCAGGCCGUGCCUGGCUCUCCCAGGGACCCAGUCAAGCCCAGCCCCCACAACAUCCUGU

>random_seq_from_cds__NO_12772

CACCACGGAGAGGUUUCAGAGGCAGAGAUGGCGGGCCGAGAGGCCCUGUCACUGGGCACAGAGGCCGAGCUGCCGAAC

>random_seq_from_cds__NO_12781

AACCCGCUGAUCUGCGCGCGCGGCUACCACGCCAGCGAUGAUGGGACCAAGUGUGUGGGACGUGAAUGAGUGUGAGACAGGUGU

>random_seq_from_cds__NO_12783

UUUGGCCGGGGCUGCAUCGGACGUGAAUGAGUGCUGGGCCUCGCCAGGCCGCCUGUGCCAGCACACGUGUGAGAACACACUCGGCUCCUACCGCUGUU

>random_seq_from_cds__NO_12796

ACCUUGACAUCUAUGAACAGCAAGUGAUGACUGCAGCCCAAAAGGGAUGUACAGAUUAUAGUGUUUCCAGAAGAUGGCAUUCAU

>random_seq_from_cds__NO_12817

GAUGACCUGAACUGGUGAAUUUGGGUAAGUAGGAUAUGCCCUGAACAUACUGAGUUAUAGGAUUCAUCGUGGUUGUGGGC

>random_seq_from_cds__NO_12824

CUUGCCAAUGCCCAGGUGGCUAAGGGUUUCCCCAUAGUCUACUGUUCCGAUGGCUUCUGCGAGC

>random_seq_from_cds__NO_12825

UUGCUGGAUUUGCCCGAACUGAAGUCAUGCAGAAGAGUUGUAGCUGCAAGUUCUUAUUUGGGGUUGAAACCAAUGAGCAACUGAU

>random_seq_from_cds__NO_12837

GGGAAGACAACAGCCUUCUGAAGUGGGAAGUUGGGUUGGCUUCAUGAGUUGGGAAAGAGACUGGAAUCUCCAUACUAUGGCA

>random_seq_from_cds__NO_12838

ACAAUACCUUGGGGGGCCCGUCGAUCCGAAGUGCCUAUAUUGCCGCUCUGUACUUCACGCUGAGCAGCCUCACCAGCGUGG

>random_seq_from_cds__NO_12862

AAUCAACAUCAUCUUCUUCUUCAUCAGACUCUGCAAAAUAUUUUACUUCUUUCCUAGCCCGACCGGUUCGUGGCAGAGAAGGUGGCUCAGUAGGGAAGUCUGAGGGGAAG

>random_seq_from_cds__NO_12874

GCAUCUGCCUUCAUAGCUGUAAUUUCAGGAAUUAUUCUUCUGCCAUAAGGUGAGGGCAUUGUCUCUUCCAACUGG

>random_seq_from_cds__NO_12890

UGACCAAUAGGCUGAAGCAAGUUAAUGUUGUUACUUCCCACAAAGUUCUGAGCCAAAUUCACAAUAGUCAUCAUCAAUGCUUGCUUCUCCAUGAUGAU

>random_seq_from_cds__NO_12917

CAGCAUUGUUCCACCCAGGUCAGUGCCCCGUUGCCGCCGCCCACGCCGGCUCCCGCGCCGCAGCCACCCGACUUGGCCAUUU

>random_seq_from_cds__NO_12926

GACAAUCUGAUGGGAUCGAAUCAUAGUUGUCUCUGAAGCCUUUUGCAAAGGGGUUAUGAUCAAUCUUUAGUUGAGUAAUCAUCGGUG

>random_seq_from_cds__NO_12930

AGUAUUAGGAGACUCUGGGUGAACAUACAUUUUGUUGCCCCUGCAUGUUAUUGUCGGCUUUGCCACAGGUCACCCAUUUGCCCCCCUG

>random_seq_from_cds__NO_12935

CCGCUGCUACCGCCCGCGCCACUGCCCGCACCGGCUCCUGGGCCGAACUGCGCCCUCCCGGGUGGGCACACAGCCGCGGGGAAGC

>random_seq_from_cds__NO_12937

ACGGGAAGAGUGAGCAGGGCGCAGCCAGCUCCGACCCCUGAGGACCGGGGGACUGGAGGUAGUACCGCUCGGAGCUCAGGCUGUC

>random_seq_from_cds__NO_12940

GCAUGGCCGCGGGGGCCCCUGCGCUGGCGGCUGCGGGCUCCCCGCUCACCGCCUCGCAGGAGAGACUGCCGGAAAACUUC

>random_seq_from_cds__NO_12955

AUGAAUCACCUUUCGUUGGGCACAACAGGAACGGUAUAUGACUGCUGCUGCUCCUAUGCAAGGGACCUACAUUCCUCAGUA

>random_seq_from_cds__NO_12972

CACUUUUUUCAUCUUCAGACAAAACCAAUUCAGAGCCUGAGUUUGGCCUCUGGCUCUGGCUGUGAGGUUUGUUCGUCUUCAGCAG

>random_seq_from_cds__NO_12977

UGAUUGUGACAACACCGGGGGCCCCCACACUGAGGUGUCUCUGGCUACAGGGAGACGCAGAAUUGGGUGUUCCAUGUGGGAGCAAAGUG

>random_seq_from_cds__NO_12980

AGAGUACACCACCAGCAUGUGGGGAGCUUCAUCGCUCAGGGACACUAUGGCUCCAGAUAAAGACAGGACUCCUCGAGGCUUCUGGUGU

>random_seq_from_cds__NO_13004

GGAGCAAGUUACUCAGAUGGAAAACUGAAAGCCCCUCCUAAACCAUGUGCUGGCAAUCAAGGGACCCAGAUCACGGGUGGAGGACCUUUUUUACAACAUAGCCACGAGGA

>random_seq_from_cds__NO_13013

CCCUGUCCAGUCAGCCCCAGGCCAUUGUCACAGAGGAUAAGACAGAUAUUUCUAGUGGCAGGGCUAGGCAGCAAGAUGAGGA

>random_seq_from_cds__NO_13014

GAUGCUUGAACUCCCAGCCCCUGCUGAAGUGGCUGCCAAAAAUCAGAGCUUGGAGGGGGAUACAACAAAGGGGACUUCAG

>random_seq_from_cds__NO_13019

CGGGAGCCAGCACCGCUCUUUGACCUUGCCAUGCUUGCCUUAGAUAGUCCAGAGAGUGGCUGGACAGAGGAAGAUGGUCCCAAA

>random_seq_from_cds__NO_13020

GAAGGACUUGCUGAAUACAUUGUUGAGUUUCUGAAGAAGAAGGCUGAGAUGCUUGCAGACUAUUUCUCUUUGGAAAUUGAUGAG

>random_seq_from_cds__NO_13022

UCAUUCUUCGACUAGCCACUGAGGGUGAAUUGGGACGAAGAAAAGGAAUGUUUUGAAAGCCUCAGUAAAGAAUGCGCUAUGU

>random_seq_from_cds__NO_13032

CGGCGGGAGGGGAUAAAUCGAUUCAUGUAAAGCUGGACGUGGGCAAGCUGCACACCCAGCCUAAGUUAGCGGCCCAGCUCAGGAUGGU

>random_seq_from_cds__NO_13038

UGCCAGCCCGUGCCUCAUCCCUCAACUCCAGUGACAUCUUCUUGCUGGUCACAGCCAGCGUCUGCUACCUCUGGUUUGGGAAGGGGCUGUAAUGGUG

>random_seq_from_cds__NO_13040

UUCUGGGAGGCCCUGGGAGGCCGGGCCCCCUACCCCAGCAACAAGAGGGCUCCCUGAGGAGGUCCCCAGC

>random_seq_from_cds__NO_13045

GCGGCUAUCCAGAUGGCCGGGCAAUGGCAGGGCAGGUGCCGUGGCCCUGCAGGCCCUCAAGGGCUCCCAGGACAGCUCAGAGAAUGA

>random_seq_from_cds__NO_13052

CAGCCCUGGCUUGCAAAGGCCUCAUUGAUCUCGAAGAUGUCCACGUCACUCACUGUCAGCCCCUGCUUUUUGCAAAGCUACUGGGAUGGCAU

>random_seq_from_cds__NO_13072

CUCCUUAUCAUAAAUAUCCACCAAUGAAGGGUUUUAAUGCUGACACUGCAGAACGAUCCUCCUUCUUUGGAAACUGGUGUUCAAGAUAAAGAAAUGCUGAAAAAAUAUGG

>random_seq_from_cds__NO_13085

CUGAACAGCUGGUACUGAGCGUGCCCCUGGACACUGCAGGUCACCCAGAGCCCUGCCUCAUGU

>random_seq_from_cds__NO_13087

UAUCCUGAGAACAGGCUCCCAUCCCUGAAGAAUGAGGUUCAACCUGGUUUGUGAUCGGAAGCACCUGAAGGACACCACACAGUC

>random_seq_from_cds__NO_13098

UGCCCGUGGUGGUCACCAUGCUGGCUGUGGUGGGGAAGAUGGCCACAGCUGCUGCCUUUACCAUCUCCUAUGUGUACUCUGC

>random_seq_from_cds__NO_13112

UAUCCUGGGACACUGCUUUUUCGCUGUUGGGGCCGUGUUGCUGACAGGGAUCGCCUACAGUCUUCCCCACUGGCAGCUGCUGUUU

>random_seq_from_cds__NO_13115

UGCCCAGAAAGAAGGUGACUCGGGCCUCUGUCCUGGACUUCUGUAAGAAUAGGCAGCUCUGCAAGGUGACCUUGG

>random_seq_from_cds__NO_13125

UGCACAAGGAUGGGCUGACGGUCACUUCUCCAGUACUAAUGUGGGUCCAGGGCACUGGAUAUCAUCUUGGAGAAGAUGAAGGCUUCGGGC

>random_seq_from_cds__NO_13129

AAAUUGCAAAAAUUUACCAGCAGAACCCCGAGGCCUACUCACAUACGGAGGAGAAUUUCUUUGGUCAGUAGCUUUGCUGCUUCCC

>random_seq_from_cds__NO_13131

CUGGUCCCAGGCUUGCCUUGGUGCCUGUGCACCUCAUUUAGAGGAGAAGCUUAGCCCACCAGUACCAUCAUGCUCAGU

>random_seq_from_cds__NO_13133

CUGGCAGGCAUGAGACUGGAGGAAGGUGACAUUGCGGGUCAGCCUGGGCACCAGUGACACCCUGUUUCUCUGGCUCCAAGAGCCCAUG

>random_seq_from_cds__NO_13134

CCUGCCCUGGAAGGCCACAUCUUCUGCAACCCGGUUGACUCCCAGCACUACAUGGCACUCCUGUGGCUUUAAAAAUGGCUCC

>random_seq_from_cds__NO_13142

GAAUCUUGUCUCAGACCCGGGGGCCUCCGGAGUGACAUGACGGCGCCCUGGGUGGCCCUCGCCCUCCUCUGGGGAUCGCUGUGC

>random_seq_from_cds__NO_13149

GGUGGGCCUGAAGCCACUGCAGCUGCUGGAGAUCAAGGCUCGGGGGCGCUUUGGCUGUGUCUGGAAGGCCCAGCUCAUGAAUGA

>random_seq_from_cds__NO_13160

CAUCUAAGUUAUUCUGUGGUAGAGAUCUGGGCCUGGCUGUCCAGCCCCUCAGCCUCCGUCCCAGUAUCUAGGCCCUCUGUGCUUGCUGGU

>random_seq_from_cds__NO_13173

UUCUCCACAUUGGCUUUCAGUAGUGCCUUCAGGGUGUCAUUCUCCUUGUUCCAGCCUCGAGUGCCCAAUGGUGAUGAUGAA

>random_seq_from_cds__NO_13175

GGCUGCUCAGCGCUGACGCCCAGGCCCAUCUCACACCGGCACAGGCUCCAUUAGAGAUGCUGGGACAGUGUCCUCAA

>random_seq_from_cds__NO_13181

CACCAGGGUCACUAGUACCAAAGAUGUCAGCUGGAUGGAAGCAGCUGAGGUUGUCCAGGUUCCCCACGCUCCCUUCCUCCUCUUCCU

>random_seq_from_cds__NO_13194

GACAGGGCGGGUGAAACUUCUGGGGCCGCAGAAGUCACGGUCAGGCAGGGGCAUCUGAUCCCAGGGGCCUUCCUCCUCUGAGUCCCA

>random_seq_from_cds__NO_13206

CACGGAAGACUGGUCUGCAGCUCCCACUGCUCAGGCCACUGAAUGGGUAGGAGCAACCACUGACUGGUCUUAAGAUGUUCACUGUGCUGA

>random_seq_from_cds__NO_13209

UGGUAUUGUGCUGGAUGCCGGGUCUUCAAGAACCACAGUCUACGUGUAUCAAUGGCCAGCAGAAAAAGAGAAUAAUACCGGA

>random_seq_from_cds__NO_13214

UCCUGGAGGAAGAACCUGUGGCACAUGUGGGUGCACCCGCAUGGAGUGGAAACCACGGGUGCCCUGGACUU

>random_seq_from_cds__NO_13220

CAAAGAUUAAAGGGCCAUUUGUGGGCUUUUGCAGGAUUCUACUACACAGCCAGUGCUUUAAAUCUUUCAGGUAGCUUUUCCCUGGACACCUU

>random_seq_from_cds__NO_13237

CCGCCCGCUGGCUAUGCGUGCUGGCAGGCGCCCUCGCCUGGGCCCUUGGGCCGGCGGGGCGGCCAGGCGGCCAGGCUGCAGGAGG

>random_seq_from_cds__NO_13244

AGUGCUCCGAGGGCUCGGGUGGGCUGUAAGGCAGCCAUGGUCUUUUUCCAAUAUUGUGUCAUGGCUAACUUCUUCUGGCUGCUGGUGGA

>random_seq_from_cds__NO_13245

GGGCCUCUACCUGUACACCCUGCUUGCCGUCUCCUUCUUCUCUGAGCGGAAGUACUUCUGGGGGUACAUACUCAUCGGCUGGGGGG

>random_seq_from_cds__NO_13258

AUUUGCCCGCAUCCUCCUACUGACCUAGUGGCAUUCAACCUCCAGGAUCCUCAGCAUGGAUUCUCCUGCCCCUGAAGCUUCUGCCCUUUCCCAG

>random_seq_from_cds__NO_13260

GGAGGUGUCAGUAUGCUUCACUUCAGAGGAAUGGGCAUGUCUGGGCCCAAUCCAGAGGGCCUUGUACUGGGAUGUGAUGCU

>random_seq_from_cds__NO_13261

GGAGAAUUAUGGAAAUGUGACCUCCCUAGGAAUGGGAGACCAUGACCGAGAAUGAGGAGGUGACAUCAAAGCCAAGUAGUUCUCAA

>random_seq_from_cds__NO_13286

AACCCUAUGGGUGUAAUGAUUGUAGUAAAGUUUUUAGGCAAAGAAAAAACCUUACUGUACAUCAGAAAAUCCACACAGAUGAAAAA

>random_seq_from_cds__NO_13294

ACCUCCUUAGGGCGCUCAGGCUCAGGGGGAGGUGCGGGAGCUGGAGCUGCCUUGGGGGCUGCCUUGGCAUCAUCCUUCUUGGGCUCUGGCUUUUUGGGGGCCAUGCUAAG

>random_seq_from_cds__NO_13298

UGGUAUCUGACCCUGGCUGGGGUGGAUGAGGUGGUGGCAUCUGGUGGUGCAUCAGAGGGUAGGGAGGGGGCU

>random_seq_from_cds__NO_13315

AUACUCUUGGGGGUUUAGACGAUACGUGUCAAUCAUAAAAUUUCGAUAUGCCAAGUAUCAUUUCUGGAGUCUUGGAUUUGU

>random_seq_from_cds__NO_13339

CAGGGACCCCAGGAGCCCCCACCACUCUCUCUCCGGGGGGACCUCGCUCACCCCUUCUGGCCCUGAAGUCCU

>random_seq_from_cds__NO_13345

UCGGAUACCAGGCACUCCAUCCUUUCCUGGGGAUCCCCGGGUCACCAGGGAUCCCUGCUGCACCAGGUUGACCCCUGGGGUCCC

>random_seq_from_cds__NO_13349

CGCUCUCCCCUGCUGCCAGGGGGCCCCCGUGAGUCCUCGGGGUCCCUCCUGGCCGGGGCGGCCAUCUUCA

>random_seq_from_cds__NO_13360

AGACCCCGGGGCACCAGGUGGUCCAGGGUCUCCAUGACCACCCCACUGGGCCAGGGGGGCCUCUUGGACCCUGCAGACCCCGGCUUCCCU

>random_seq_from_cds__NO_13373

GGUUUGCCAUCCUCGCCUGGCUUUCCCCGGUAAUCCAGGGGGACCAGAGGGGCCAGGGAGGCCCUGUUCUCCACG

>random_seq_from_cds__NO_13375

CAGUCCGCUCCGGCCAUCCAGCCCAGGGGGACCCCGGUCACCCCUUUUCUCCUGCUGGGCCUCGGACACCUGGGU

>random_seq_from_cds__NO_13384

UGGGCCUGGGGGUCCACGUUCGCCCCUUCUCUCCAGCCUCACCCAGGGGCCCUGGAAAGCCCCGGUCA

>random_seq_from_cds__NO_13386

CCCUUUUUCUCCUUUCUUUCCAGGGGGGCCAACGGGGCCUUGGGGUCCAGGGCUUCCGGGAAGACCCCGGCAG

>random_seq_from_cds__NO_13392

CCGGGAGGCCAGGGUCGCCAGGAGGCCCAACUUGUCCUCUCAGGCCCCAUCUCUCCAGGUUCCCCCUUCUGGCCCCUUUGGACA

>random_seq_from_cds__NO_13393

AUACACUGGGCAGGGCUCUGGCCGGGGCCUGAGUAGUGAAGGAUGCCUGACACAGGGCUGUGGCCAGACCACUGACUG

>random_seq_from_cds__NO_13415

GAGUUUCCGGCUCCCCGGCGGACAGUGAGGACACUGGCACUGCCCUCACGGGGACCCACUCGAGCAGACAC

>random_seq_from_cds__NO_13417

AGGGUCCGCUCAACCCCCCUGGGUGCUGCGCACAAUGAUGCGGUACUGGGUGGCACCAGGGACUGGGCUCCAGGACA

>random_seq_from_cds__NO_13428

GCUUGGCUCAGACAGCACCAGGUCUCGUGGAGCAGAGGUCGAGUCAUCCGCGAGGUCGGGUCACAGGCACGCCACCAGCAGUCGU

>random_seq_from_cds__NO_13433

AUCACAUCACCCCCAGAGCCAAGUGCAUCCAGGCCGAACUCUGUCCCGUGGGUCAUCGCUGUACUGCACUGUGGCAAAGCGCAC

>random_seq_from_cds__NO_13439

ACUGUUCUGUGGAGGGGCCAUGGCUGGGGCCACUGACCGAGUCCUCCCUAGCUCUGAGCAGCUGCGGG

>random_seq_from_cds__NO_13455

GAGAGCCGUGUUGUUGUAGGCCCCAGGUCCCCAGCCCAGGUGCUGGCCUUGCCUCCUCAGGCGCUGCCUUCCU

>random_seq_from_cds__NO_13474

GAUUCCUCAUUGAAGUGGGCAUCCUGUGUGGCUGUCAGCCCGAAGCCCUGCUGAUGGCUCUCGAAGGCCAGCAGGUGGGCCAGC

>random_seq_from_cds__NO_13477

CCAGCCAACCCUGGGCCUCAUCACACAGCCGCACAGCAGCACCCCAGGGCCCCCCGGGGACAGGGCACUGUGGCCAGGACGCCAAACUUUGUCUG

>random_seq_from_cds__NO_13479

CCUCUGCGAAGGGACUGUCACAGCUGUUGCACUGGCGGCCAAGGGCUCCUGGGCGACAGGGGCACUGCCCGCUGU

>random_seq_from_cds__NO_13500

CGAAAGCCGCCGUUGGGCGCGUCGGUGCAGGUGCCCCCGUUGCGGCAGACGCCCGGCACGCAGCGGCCGGCCUCGGUGUCCAGCUCGCAGUCCUCUCCC

>random_seq_from_cds__NO_13505

CCCUGCAGCGCCCGCCCCGGCCCCACGUGGAGCUAGCGCCGAGAAACUCACAUUGAGCACGGUGCCCCCUACGUCUGUGUCGUUCUGGAUGUUG

>random_seq_from_cds__NO_13508

UGCGCCGUCACGCUGUGCAGGCCAUCCUGUGACAGUCACCAACAUGGAGGCCACCAGUGGGCGGUUAU

>random_seq_from_cds__NO_13513

GUUCCCCUCCACGAUCUGGUACAUUAUAUGGGCAUUGGGGCCUUCGUCAGGGUCCACUGCAGUGAUCUGGGCCACCACUGAGCCCACAAUGCUAUUCUCUUUCACCCGC

>random_seq_from_cds__NO_13523

CCCCCCUGGGUGCUGAUGGCAAAGCGAUUCCGGGUGUUGCCGCCUGUGAUCUGGUAGCUGAUGGCACUGUUG

>random_seq_from_cds__NO_13533

CGCACAGUGGCCGAGCGCGGCCCGGGUUCCUGGCCCUGGUCGCUGGCUUCCACCACCAGCUCAUAGCUUUCC

>random_seq_from_cds__NO_13540

CAACCACGCGUAGCACCGCGGUGCCUGCUGCCUCAUUCUCCGGCACCAGCGUCUGGUAGUUGUACUGCGGAAACUGCG

>random_seq_from_cds__NO_13545

GCGGUGCCCACUCUUUUGCGGGAGCCUGUCCCAGCGUUCCGCUGGGAGGACACCGGCUUGGGACCGUGGUGCCGAAUCAAAAAGUC

>random_seq_from_cds__NO_13550

CAAAGAGCUAAGGCUCCGCCACCGAUAUGCGCCCUUGGCCCCGUAGUGGCAGCUAAGCCUGGGUCCCAGC

>random_seq_from_cds__NO_13551

CCUGGUGCCCACCGCCCCCCAGCUCCUCCUGGCUGAGGGGGAACAAAGAGAGGAGAAGGAGCAGGAGUAUGGGGGUCGACCGUC

>random_seq_from_cds__NO_13567

GGCAUCGGGCCCUGGCGGGGUAGUAGCUUGCCUAGAGAUCUCAGCCGCACCCUCUGGCAAGGCCAUCUUUGUCAA

>random_seq_from_cds__NO_13574

UGGGGAUGUCAUGCUGUACAGGGCUCUGGGUGGCUGCACCCGGCCACACGUGAUUCUCCGGGAGGGUCUGC

>random_seq_from_cds__NO_13577

CACAGGCUCAGCCCACGCACUCACAGCUGUUUGUAACCAUAUCUCCUCGGUACGUGCUGUGGCUGUGUGGG

>random_seq_from_cds__NO_13583

CAGCCUGGCUUUCUGGGAUCUCACCACCAUGCUAGACCAUGACUCCACUGUCCUGGAGCCUCCAGUGGAUCCUGGGCUUCCCUACCGGGCUUGGCACCCCCUCCCUGACU

>random_seq_from_cds__NO_13589

CCUGUGAGCCCUGAGUUUGGCCACCGUUGUGCCCUUGGGGGUCAGGGGCUUGAGGUUUACAACUGGUAUGAC

>random_seq_from_cds__NO_13597

AGCAGGAAAGUUGGUGAUGAUGACCCGUAGUGACUCCAGCACAGCCAUGGCUCGUGGGGCUGUGUCAUUCAGCACAUCACGCA

>random_seq_from_cds__NO_13608

AGGUGCUGCUUUAGUAGAUUCAUGGUGUGUGGAGUGACCACAUAGCCUGGGGUCUUGUAGUUCUCACCC

>random_seq_from_cds__NO_13614

GCCGCUUAGCUGGGGCUCAGUGUGGAUCUUCUUACUGGCUAUGUAGCUUACAAGGAAGGAGAGACGCCGGGUAUCCCUGAGUCGGGAGGCCAAGCCAU

>random_seq_from_cds__NO_13616

GCUGCGCGCUCAGAGCCGAGUUCUUGAGCGUCUCGCGGGCCUUCUGCUCGCUCAGGCCGAGGCUAGUGAAGAGCGACAGG

>random_seq_from_cds__NO_13620

CUGGGCACGACCCUGGGCACUGCCUGCCGUUUCUUCUGCUGUAGAGGCUGCCAGACUAUUUCCUGCCCGUU

>random_seq_from_cds__NO_13622

GUACCCUGGUACAGGGUCUGCUCUGUGUCCCGUGUGUCAGCCACUGCCCCCCGGAUGGCACCCUGGGCAAUACCCUGUGCCCGCU

>random_seq_from_cds__NO_13627

GCUGCCUGGGCCCGCUGCUGUGCCUCGCUUGCCUGCCGACGAGUCUCAGCCACUCUGCUGAGGAUGCUACCACCUUCUGCCA

>random_seq_from_cds__NO_13629

UCUCGACAGCCGGCACCCCCACAAGGGCUUGUAGCACAGGGUGCAUCCCCUGGUGCCCCACACACCCAGCUCAUUUAUGUCUGUCAGGCUCAGGG

>random_seq_from_cds__NO_13634

UGCUCAGUGGCCUCCCCAAUUUCACGCCCGCAGCUCCUCUGUGGCCUCCACAAGCUGUGCAGUGGAGGCGGCUGAGGUGUUGCGGGCACCUACGAUGCCCUGCACAAUGC

>random_seq_from_cds__NO_13640

GCCACUGGUGAGGUUCCAGAAGUUGGGGGCACAGCGGUCACAGCUAGGGCCCUGGACAUUGGGGAGGCAUGGGCA

>random_seq_from_cds__NO_13642

UCGGGCAGCCUGCCCAUGGAAGCCAGGCUUGCAGUGGGCACAGUGUGGACCCUCUGUGUGGUGUAAACAGCGCAGGCAUUGCCCCGUG

>random_seq_from_cds__NO_13649

CACUGGCAGGCCUUGACAGCCUGUGGGGCCAAAGCCAUAGUAGCCAGGGGCACAGAGGUCACAGCGGCGCCCAACCACUCCAGGCUUGCACAGGCACUGACCACCAUGAG

>random_seq_from_cds__NO_13655

UCAGUUCCAACUCUGCCCAUUGCUCAGGGACCCUGGGGCUCUAAGCGCAGCAGCAGGUCAUAGUCCAUAGCCUUCGGCACAGAG

>random_seq_from_cds__NO_13656

GCCACCAGGAACUCCAGGGUCUGACCUUCCUGUAGCCGCACGAAGCCUGAGCCAGUCCAGGAUGGAG

>random_seq_from_cds__NO_13667

GAUGCAAGCUCCGUGCACCCAUGCCCUCAGCAUGGGCUGGUGCCCCUGGGGCGGGUGCACACUCUGAGGCGUGUCCGUAG

>random_seq_from_cds__NO_13670

GAGCUGUAGGGGUCUGGGAUAGGGAUGGCAGGGUCCAGCACACGAUAGAUGACCCUCGCCUUCAGUGGAUGGCUCAAUCUCUGA

>random_seq_from_cds__NO_13674

CCAUUCUCUGACUGCCACCAGGCUGCCCGCCGCUGUGGUGCAAAGCUGGUGACUACAUUCUGGAUGCGAUGGCUG

>random_seq_from_cds__NO_13682

CAGCGGCAGGGAAGGAGGCUUUGCCAUCUUGGCUGCACUGGGACUCACAGAGCCACACCCUGGAGGGCCUCCCCC

>random_seq_from_cds__NO_13687

GGAAAUGCAAAAAAGGUGGUGGAGAAUGGGGCCCUUCUCUCCUGGAAGCUGGGCUGCUCCCUGAACCAGAACAGUGUGCCUGACAUUCAUGGUGUAGAGGCCCCUGCCAG

>random_seq_from_cds__NO_13688

GGAGGGCGCAAUGUCUGCUCAGCUUGGCUACCCUGUGGUGGGUUGGCACAUCGCCAAUAAGAAGCCCCCU

>random_seq_from_cds__NO_13692

GAAGCUGGCACCACAGUUCCUGGCCAGAUUCGCCCAACGAUGACCAUUCCUGGCUAUGUGGAGCCUACUGCAGUUGCUACCCCUCCCAC

>random_seq_from_cds__NO_13695

AUCACCAGAUUGGAAACUGCCUCACCGCCUACUCGUAUUCGCACCACCACCAGUGGAGUGCCCCGUGGCGGAGAACCCAACCAGCGCC

>random_seq_from_cds__NO_13700

CUGGCCUUCGCCUUUGGAGACCGAAACUGUAGCACCAUCACCCUGCAGAAUAUCACCCGGGGCUCCAUCGUGGUGGAAUGGACCA

>random_seq_from_cds__NO_13702

UCGGCCUGCCUUCUCCAACGCCCUAGAGCCUGACUUUAAGGCCACAAGCAUCACUGUGACGGGCUCUGGCAGUUGUCGGCACCUAC

>random_seq_from_cds__NO_13703

AGUUUAUCCCUGUGGUACCACCCAGGAGAGUGCCCUCAGAGGCGCCGCCCACAGAAGUGCCUGACAGGGACC

>random_seq_from_cds__NO_13704

CUGAGAAGAGCAGUGAGGAUGAUGUCUACCUGCACACAGUCAUUCCGGCCGUGGUGGUCGCAGCCAUCCUGCUCAUUGCUG

>random_seq_from_cds__NO_13706

AUCUUUGCAGACGAACUGGACGACUCCAAGCCCCCACCCUCCUCCAGCAUGCCACUCAUUCUGCAGGAGGAGAAGGCUCCCCUACCCCCUCCUGAGUACCCCAACCAGAG

>random_seq_from_cds__NO_13715

ACACACUUGUUGUAUGUGGCAGAGAAGAAGCGCCCCAAGGCCGAGUCCUUCUUUCAGACCAAAGCCUUGGACGUCAGUGCCAGCGAUGA

>random_seq_from_cds__NO_13722

GAGAACUUCUCUGGGAUCUACUGCAGCCUUCUGCCUUUGGGAUGCUGGUCAGCUGACAGCCAGAGAGUGGUCUUUGACUCGG

>random_seq_from_cds__NO_13726

ACAGCCACCCCCAGAGCAAGAGAAUGUGCAGUAUGGCUGGCCUUGACUUUGAAGCAAUCCUGCUGCAGCCUGGCAGCCCUCCAGA

>random_seq_from_cds__NO_13730

CAUGUGGCCCUUAUGGGUGGUUCCCAUGGUGGCUUCAUUUCCUGCCACUUGAUUGGUCAGUACCCAGAGACCUACAGG

>random_seq_from_cds__NO_13734

AGACCCGGAAUGUGCCUGUUCGGGCUCCUGCUCUAUCCCAAAAGCACCCACGCAUUAUCAGAGGUGGAGGUGGAGUCAGACAGC

>random_seq_from_cds__NO_13747

CCACGGUUGGCUAUGAUGCCCAGAGCAUUGAGUGGCAGGUGCACACCAAAGCGGGCCUCACAGUGUGC

>random_seq_from_cds__NO_13750

GGUGAUCCAGCUUCACGAUGCCCCUGAGGAAUCAGAUUCUUAUGCACUCGCCUCAGCUUGGCCCGCUUCUGCCCGUUCUUG

>random_seq_from_cds__NO_13754

CGGCCGGGACUUGGAGGCGGUGCGGCGCGGCGGGUGCGGUUCAGUCGGUCGGCGGCGGCAGCGGAGGAGGAGGAGGAGGAGGAGGAGGAGGAGGAUGAG

>random_seq_from_cds__NO_13760

AGAGCUGAAGAAGAUUCUGGAUGACUGGGGGGAGACAUGCAAAGGCUGUGCAGAAAAGUCUGACUACAUCCGGAAGAUAAAUGAACUGAUGCCUAAAUA

>random_seq_from_cds__NO_13771

GGGGGCCCUGGAGAGUGUGGUGGCAGGCAGUGAGGGGGCUGCUGAGGGUGCUAUCACCAUCGAGCUGGCCUCCUACCCCAUCAGUGACUUUGCC

>random_seq_from_cds__NO_13775

AUGCGGCCAGUUAACGGGCGCCGCCUCUACAAGGACUUUGUGCUCAACGUCAAGUUUGAUGGCCCCCUUUCGCCCAGCUGACACCCACA

>random_seq_from_cds__NO_13776

AUGAGGUCCGCUUUGACCGCUUUGGUGAUGGUAUUGGCCGCUACAACAUCUUCACCUAUCUGCGUGCAGGCAGUGGGCGCU

>random_seq_from_cds__NO_13784

CGGCCACGCUUCAUCAGUCCUGCCUCACAGGUGGCCAUCUGCCUGGCACUUAUCUCGGGCCAGCUGCUCAUCGUGGUCGC

>random_seq_from_cds__NO_13793

GCCUGGGCUGUCAGAAAGUAGAGGGUGGCACCUGGCUAUGUGGUGACCGUGUUGACCUGGCCAGGCACCAACCCUACACUCUCCUCCAUCUUGCUCAAC

>random_seq_from_cds__NO_13796

CCAGAACCAUCCACAUGACCUUUGUGCCUGGAUGAGGAGGUUGGGGGUCACCAAGGCAUGGAGCUGUU

>random_seq_from_cds__NO_13798

CUGUCUUUUAUAGUGAGCGGAGUCCCUGGUGGGGUGCGGGUUACCAGCACUGGGAGGCCAGGCCAUGCCUCACGCUUCAUG

>random_seq_from_cds__NO_13801

CAGCUUUGACUUCCGUGUGGCACCGGAUGUGGACUUCAAGGGCUUUUGAGGAGCAGCUGCAGAGCUGGUGCCAGGCAGCUGGCGAG

>random_seq_from_cds__NO_13804

CUCACCCAUGAACCGCACACCUGUGCUGCUGCACGACCACGAUGAACGGCUGCAUGAGGCUGUGUUCCUCC

>random_seq_from_cds__NO_13807

UUUGGCCGGCACAGCCUGAGCCCCUUGGCAAUACGGGCACGAGCACGCUUCCCAAGCUUGGGGUGGGCAAUGUAGGCAAGUCGAUCG

>random_seq_from_cds__NO_13811

AGAAGUUCCGGUUAUAGAAGUGGUGGUUGUCCCUGGUCAGGGCCAUGCCCAGCUGGGCCCAGAAGCUGCGCUGACCACUGG

>random_seq_from_cds__NO_13814

CACUGACCCGGUCCGUGUGGGCCAGCACAAACAGCGUCUUGCGGCUGCCAUAGACCGAGGCCCACAG

>random_seq_from_cds__NO_13819

GGUCCUGUGCAAAGAUGCUGAGGCCCUGGAGCUGGCCCGGACUGCCACACUUCACCCGGCUGGGCAGACCGG

>random_seq_from_cds__NO_13835

AUUGAAGGACAGGUUAAGCUUGCGCAGCUGUGUUAGGCCCUGGAAGGCCUUGGUUUUAGUGAUGCAUUUGUAGAGGAAGUUCUC

>random_seq_from_cds__NO_13837

CGGCUCAGGUGGCUGAAGGUAUCGGGAUGUAGCUGGGGGAAGUGACGAGGGCACUCCAUGCAGGGGUUGGGAGCGUGG

>random_seq_from_cds__NO_13840

GAGGUUGCCCAGGCCAAGGAGGGCACCCGGGGCCACCUCCAGUGCCUGCCUGCAGGGGUUCU

>random_seq_from_cds__NO_13842

AUGGCUGAGGGACAGGGAUAUGAGGGAUUUGGGCAGCGCAGGCACAGUCAUGAUGUUGUUGUAGCUCAGGU

>random_seq_from_cds__NO_13844

GGGCAGUUCCACUUGAGGUUGAGAUGCCGCAGGCUGGGCAGGUGGGCAAAGUCAGAAUCAUGGAGGUGGUGGAU

>random_seq_from_cds__NO_13862

CCUGUGUGGUUGCCCUCAGAGGCUGCAGGGGCCCUGUUUGCUUCCAGCACCAGCGGGGACUUGUUGCUGGCUGACUUGGACUCCUCAG

>random_seq_from_cds__NO_13865

GCGAGGCCGAUACGCUCCAUGAUGACCCGCCGGGCCUUGUCUGUCCACUCCUCGUCCUCCCCCCAGGGCCCCAUGGUCAAUGGGGU

>random_seq_from_cds__NO_13892

AGGUGUAUUUGAAGAAGAUGCCACAGCUAUUUCCAACUAUAUGAACCAGUUGUAUCAAGCUAUGCAUCGGAUUUAUGAUGCACAGGAAUGAAUUAAG

>random_seq_from_cds__NO_13894

CUACAUUGCAACAGUUUUCAAAAGUUAUAGAUGAGGCUUAGCUCUUGUCAUGCAGUGCUUUCAACUCAACUUGCUGAUGCCAUGAUGUUCCCCAUUACC

>random_seq_from_cds__NO_13901

AGUAAAACAGGCUUGGUGUCAUCUACCUGGGACAGACAGUUUUACUUCACGCAGGGUGGAAAUUUAAUGAGUCAGGCCCGUGGG

>random_seq_from_cds__NO_13902

GAUGUAGCAGGAGGCCUGGCCAUGGACAUAGACAACUGUUCAGUGAUGGCUGUGGACUGUGAAGACAG

>random_seq_from_cds__NO_13912

GGGAGAAGUGAAAGUAAUCUGUCAUCAGUCUGCUAUAUAUUUGAGUCAAACAAUGAGGGGGAAAAGGAUAUGUGAUUCUGUUGGACUGGCAAAACAGAUAGCUUUGCAUG

>random_seq_from_cds__NO_13918

UUUAGGCGGCACAAAUGUUUUAGGGAGCGAGGGUUUGCUUAAGAUAAAAUGUAUUUCUGACCAGAUCCCCUGUUUUUGGAGCACAGCUUUCAACUUUGAACAGAUCCGAA

>random_seq_from_cds__NO_13928

GGCAAAUCCUGUUUGUUUGCAAAAAGUAGCAGCACUGCAUCUCUCAAUUCAUCUACCAGAAGCCAUUUUCUGCAGCUCAUCUGCUACUUCCUGAAUUCUUUCACGAUCGUUGCUAUCUA

>random_seq_from_cds__NO_13934

UAACAGGAUGGCAUCCUUCCCGGAUCAGGCGGAGCAGGUCCAGGUAGGCUGUUCUUGCCAUGUCCACUUGGGCACCAGACAGGA

>random_seq_from_cds__NO_13937

UAGCAGUGCACCCUUAGCAGCCUGGAGGUGUAUGACAGUGGUCUGGUUCCAAGCCUCGUGCUGGUCAGCUCCGGAUUGCGUCAGGGUCUG

>random_seq_from_cds__NO_13943

GAAGUCUUGGUUCAGAAUGGCAGUGUAGGAGUGCUGGAAGAACUCCAAGAGGCUGACUGCCAGGAAAUGGA

>random_seq_from_cds__NO_13957

CCAGGUAUCCCCCAAUGACACUCGGUGCACUGGGCUGCCCAUCAUGCGGAGGUUACUGGAACCG

>random_seq_from_cds__NO_13967

CUUACCAUCAGGCUUGAGGCUUUUUAUUCCAUCUUCACCACGGAGCAGCAAGACCAUGUCAAGUCGGUGGAGUAUCUGAGAAAUAA

>random_seq_from_cds__NO_13975

GCUCGCCGGCCUGGGGUUCGGCGGUGGUGGCAUCUCCUCUUUCCCCAGCACUGUGUGGCCCACGCGCCUCCCGACGGCCGCCUCAGCCA

>random_seq_from_cds__NO_14006

GAAAAUGGAAAUGGUCCCAUGACAGUAGACAAAAAUGGUGCUGUUCUUAUUGCAGAUGAAUCAGACCCUGCUGAGAGCAUGGAG

>random_seq_from_cds__NO_14008

GGCGGCGGGCGGAGCAGCGGCCGCGGCCGCCCGGCAGCAGCAGCAGCAGCAGCAGCAGCAGCAGCCGCCGCCUCCGCAGCCCCAGCGGCAGCAGCACCCGCCACCGCCGC

>random_seq_from_cds__NO_14009

CACGGCGCACACGGCCGGAGGACGGCGGGCCCGGCGCCGCCUCCACCUCGGCCGCCGCAAUGGCGACGG

>random_seq_from_cds__NO_14010

UCGGGGAGCGCAGGCCUCUGCCCAGUCCUGAAGUGAUGCUGGGACAGUCGUGGAAUCUGUGGGUUGAGGCUUCCAAA

>random_seq_from_cds__NO_14017

UCUGCGGUGGGGCCAACCUGUCCUGCUACUGUGAGUUCCUUAGUCAAGCCUGGCCUUAACUGCCCCUCAAUACCAAAGCCAACCUUGCCU

>random_seq_from_cds__NO_14023

CUCCACACCCUCCCCUGCCUGCCACUGAGCCAGCUUCUCGGUUAUCCAGUGAGGAGGGCGAAGGCGAUG

>random_seq_from_cds__NO_14044

GAGGGUAACUGUGUUUCCCGCUUCCUUGAUUAGGUUCACAAUGUCUGAAUGGGAUUUGUUGGUGAUGGAACAUCCAUUUACUGCC

>random_seq_from_cds__NO_14048

CACCGUGUUCAGCGAGUUCUGGCUGCCCUGCGGAGUGCGCUUCUCUUCUGUCAGCGAGGCCGGCUGGUUGCUACUGUGAUGAGAGG

>random_seq_from_cds__NO_14056

UGAGAACUAUUCUGGCUGUCUUUCCUUUCCAGUGGUUGCCGACUUUGGGCUCUUCUUGGGAACUGGCAGCCCCUCCUCGUU

>random_seq_from_cds__NO_14066

GAAGUGGAACUUCUCUGGCUGGCUCUGGAUUGCUUGGAGGGUGGUUUGGAAUAACAGGAGGCACAAGGGCUGAGUGAUC

>random_seq_from_cds__NO_14067

UUCUGUCCAUUCCUUCUGUCUGCUGCUGCUGCUGCUGCUGCUGCUGCUGUUGCUGCUGCUGUUGCUGCUGCUGCUGCUGCUCAA

>random_seq_from_cds__NO_14069

AGCAGGCAGUUCCUAGUUCACUGUCCAGCUCCUCGGUGUGUACCCCUUCCAUCAUCUUCACACUCUUCCAGUGGCUUCUGCUGC

>random_seq_from_cds__NO_14071

CCCAGUUUUCAGGUAGAGGACCUAAAUUAUCCUCUGCAGAAAGAGGUAGGUAUUGAGGGAACUUCUGAGAAG

>random_seq_from_cds__NO_14072

GGUCCGUGAUGGGAGCAGCGAUGAUGCUACUAUUCACAGGUGGUAAUGCUGUUUCUUGGAGAGUGUGCUCCUCUUGUUCACCAGAAUCGGCCUG

>random_seq_from_cds__NO_14074

UUGGUUCGCUUCGGGGUCGACUGCUUAGAGCCAGACUGAAGGCUGUGCAAGGCAUCCGUCGUGAUCACUU

>random_seq_from_cds__NO_14078

AUGUCGCAGGUCCUUGUUCAGCCUUCCUCCUUUUAGCGUUUUCCACCAACUCGGGGAGCUGAAACUUUCACAGGCUUCACAAUCUUUUGCUUAGGUGCU

>random_seq_from_cds__NO_14096

ACGGCUCGCUGAAAGCUCAAAGGAUGAAAGAGAUUCAUGCUUCAAGUCAUCGCCCAAAAGCCUGAUUUUCUG

>random_seq_from_cds__NO_14102

CAAUGUUAAAUUUCUAUUAUCUGAUGCCGGUGUCCUUACAGUACUGGACCAAGUACUGUCCCAUGACUUUUAGUAGAUGAUUAUAC

>random_seq_from_cds__NO_14120

CCUUCAGCGAGAACCAUGGGGUGGUGAUCCAGCCUGCCUAUAAGGACAAGAUAAACAUUACCCAGCUGGGACUCCAAAACUCAACC

>random_seq_from_cds__NO_14124

AUAUCAAAGACCCUAAGAAUCAGGUGGGGAAGGAGGUGAUCUGCCAGGUGCUGCACCUGGGGACUGUGACCGACUUUAAGCAAACCGUCAACAAAGGGCUAUUGGU

>random_seq_from_cds__NO_14130

AAACUCCUUCCGAGGAGAAGAAGGGGGAGGGUGAUGCUGCCACAGAGCAGGCAGCCCCCCAGGCUCCUGCAUCCUCAGAGG

>random_seq_from_cds__NO_14137

ACAACGAUGACAUCUAUGGGGCUGCCUGGAUCGGCAUAUUUGUGGGCAUCUGCCUCUUCUGCCUGUCUGUUCUAGGCAUUGUAGGCAUCAUGAAGUCC

>random_seq_from_cds__NO_14142

ACUAGGCGUGCCUGGUUUUUAUCACAAUCAGGGGCUGCUAUGAACUGAUCUCUGGUCCAAUGAACCGACACGCCU

>random_seq_from_cds__NO_14144

UGAAUAUUAACUCAAUUAGGUUCUGCUGGGUUCCUGGAGUUGGGAGUGAAGUGGCUCUUGAGUGGCUCCCAGCACUUGUGGUAGUUC

>random_seq_from_cds__NO_14153

UAGCCCCUGGUCUCCUCAAAGACAUCUAUGCUGAACCGCAUUCCUCUCCUGAAUGACGCAGAUCUCAUUGGGCUGUACAAGCAUC

>random_seq_from_cds__NO_14159

UUUAACUCAGCCAUAAUGAUACCUGGAGGCUUAUCUGAGGCCAAACCCGCCACUCCAGAAAUCCAGGAGAUUGUUGAUAAGGGUUAAACCACAGCUUGAAGAAAAAACAA

>random_seq_from_cds__NO_14173

AGGUGGACUUUUCCCUCUACAGAGAUUAGACAAAGCCCAUACUGCAUUCCGGGUCAUGGUCAGGCGGUUUUGCUUUGAAAAUAACCUGCAAAAGAGG

>random_seq_from_cds__NO_14191

AAACACUGGAUGCUACCUCGCUUUUUGGCAUCUUCCAGGAACAGUGAAAUGUUUCUCCAAUGAUAGGAUUGUAUGGUUUUUUAGCAAUGGCUCCCUUA

>random_seq_from_cds__NO_14212

ACGAGAUUAAAAGGUGACCGAGGAGGUGUCCAUUUCUUAAAGGCUUUACGUCGUGGGGGGCUAAGAGCCUUCUUUGUUAUAUU

>random_seq_from_cds__NO_14214

AGUGAAGUCUUUCCUGGUUGGUGAGCAGUUGUUGUCCAUUUCAGAGCCACGUUUUAAAAUGUCAGUAUGCAAAUGUUCUUUCCUUUCCACAACUUCUACUUU

>random_seq_from_cds__NO_14222

AGAUAAUUAGCAAGUGAACUUUUGGAUCUGAACUUCAGUCCUUGUGGGCCUGAUAAAGUACACAUCAAAUCUUCCUGCUGUCUUCCCAA

>random_seq_from_cds__NO_14230

CCACCCUGGGCGGGUGAAAUUGCCCUGUGGUCCUUGGUGGUCCUGGCCAUCGAGCGGUACGUGGUGGUGUGUAAGCCCAUGAGCAACUUC

>random_seq_from_cds__NO_14231

CGCUUCGGGGAGAACCAUGCCAUCAUGGGCGUUGCCUUCACCUGGGUCAUGGCGCUGGCCUGCGCCGCACCCCCACUCGCCGGCUGGUCCAGGGUACAUCCCCGAGGGC

>random_seq_from_cds__NO_14233

CACUUCACCAUCCCCAUGAUUAUCAUCUUUUUCUGCUAUGGGCAGCUCGUCUUCACCGUCAAGGAGGGCCGCUGCCCAGCAG

>random_seq_from_cds__NO_14272

UUGGCACUGGUCUGGACGACAUCCUUGAGACGAUCAGAAUUCAGUGGGAGAGAGAUGUUGAAAAGAACCGGGUGGAG

>random_seq_from_cds__NO_14274

GCCAAGUCCAGAGCCUCCAGGCUGAGACAGAAUCCUUACGUGCCCUGGAAACGAGGCCUGGAGAAC

>random_seq_from_cds__NO_14278

CCGUCCUGGGGGCUGUGUCUGGCUGUCCCUGAUAAAACUGUGAGAUGGUGUGCAGUGUCGGAGCAUGAGGCCAC

>random_seq_from_cds__NO_14285

CUGAAGGAUGGUGCUGGGGAUGUGGCCUUUGUCAAGCACUCGACUAUAUUUGGAGAACUUGGCAAACAAGGCUGACAGGGACCAGUAUG

>random_seq_from_cds__NO_14288

CUCAUGGGAAGGACCUGCUGUUUAAGGACUCUGCCCACGGGUUUUUAAAAGUCCCCCCCAGGAUGGAUGCC

>random_seq_from_cds__NO_14290

CCCCAACAGAUGAAUGCAAGCCUGUGAAGUGGUGUGCGCUGAGCCACCACGAGAGGCUCAAGUGUGAUGAGUGGAGUGUUA
